# Electronic and Steric Tuning of Molecular Acidity toward Unified Models for Excited State Proton Transfer

**DOI:** 10.1002/advs.202517140

**Published:** 2025-10-13

**Authors:** Cheng Chen, Ivan N. Myasnyanko, Mikhail S. Baranov, Chong Fang

**Affiliations:** ^1^ Department of Chemistry Oregon State University 153 Gilbert Hall Corvallis Oregon 97331 USA; ^2^ Institute of Bioorganic Chemistry Russian Academy of Sciences Miklukho‐Maklaya 16/10 Moscow 117997 Russia; ^3^ Pirogov Russian National Research Medical University Ostrovitianov 1 Moscow 117997 Russia

**Keywords:** excited‐state proton transfer, photophysics and photochemistry, reaction mechanism, structure‐photoacidity relationship, thermodynamics‐kinetics relationship, ultrafast spectroscopy

## Abstract

Photoinduced proton transfer powers a myriad of functional processes from bioimaging to photocatalysis. However, the elusive structure‐photoacidity and thermodynamics‐kinetics relationships remain the hurdle for developing such useful tools. Herein, these problems are tackled by systematically investigating photoacids with varied strengths via substitutions on the archetypal green fluorescent protein chromophore. This study quantitatively demonstrates that the thermodynamic driving force of excited‐state proton transfer (ESPT) in water is governed by electronic and steric effects exerted by the substituent. Importantly, two different treatments are proposed in calculating ESPT driving force for the fluorescent and nonfluorescent photoacids. In the latter case, the unusually fast ESPT kinetics result from the extra driving force due to the Franck‐Condon excess vibrational energy besides the free energy difference, thus providing the missing link in current ESPT theory. Furthermore, the thermodynamics‐kinetics relationship for ESPT is unveiled to follow the Bell‐Evans‐Polanyi principle. The work offers the highly desirable predictive power to engineer photoacids with strategic substituents for targeted properties.

## Graphical abstract

Conventional excited‐state proton transfer (ESPT) theories explain the kinetics of fluorescent photoacids, but face challenges for weakly or nonfluorescent ones. This missing link is bridged by making and dissecting series of green fluorescent protein chromophore derivatives with varied photoacidities via strategic substitutions. Structure‐photoacidity and thermodynamics‐kinetics relationships are revealed with a uniform treatment of ESPT driving force.

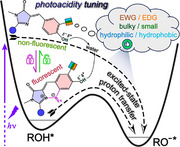

## 1 Introduction

**Figure 1:**
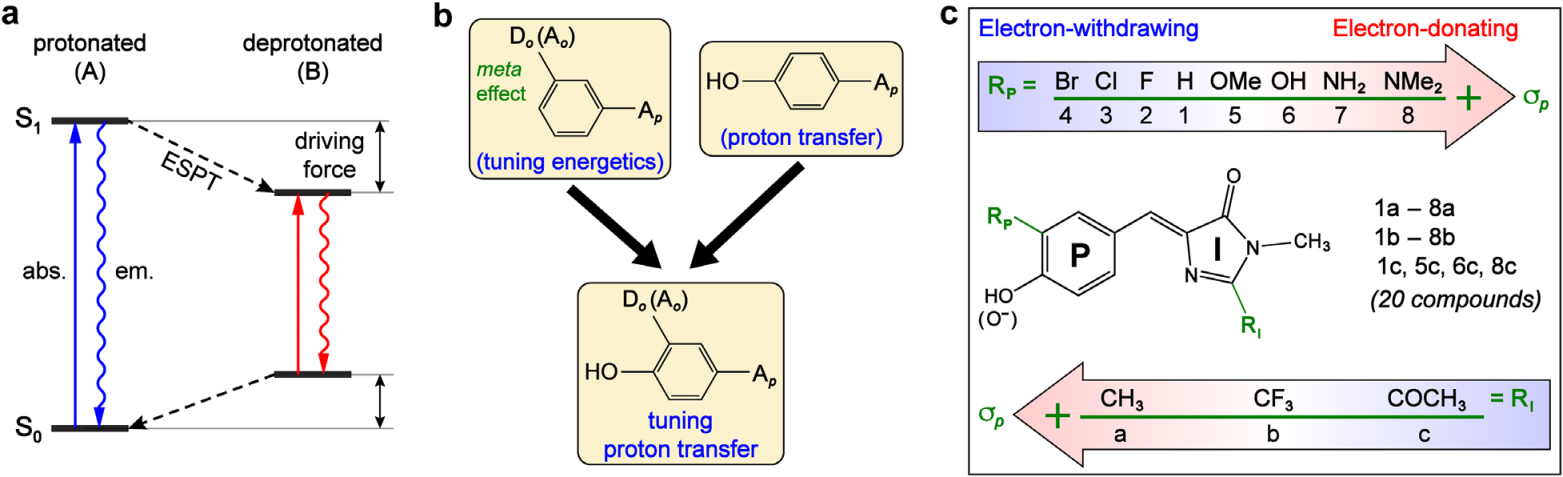
Energy diagram and strategic tuning of the excited state proton transfer (ESPT) driving force. a) Förster cycle as the general mechanism for ESPT with driving force determined by the free energy difference between the relaxed excited states of acid and conjugate base. b) Tuning of ESPT driving force for a phenol‐based photoacid. The *meta* effect (between D_o_ and A_p_) inhibits ground‐ but not excited‐state electron delocalization and was reported for tuning spectral color.^[^ ^35^, ^36^ ^]^ c) Structural modifications of GFP chromophore to tune photoacidity. The donor (R_P_)/acceptor (R_I_) moieties are modified by electron‐donating and ‐withdrawing groups of different strengths corresponding to Scheme in b.

Photoinduced or excited‐state proton transfer (ESPT) is pivotal to numerous natural and artificial processes with direct relevance for chemical, energy, biological, and health disciplines. Stemming from bioimaging, biosensing to enzyme activities, acid‐catalyzed reactions, and photoacid‐sensitized photovoltaic devices, the light‐triggered proton release plays a central role in modulating fluorescence, regulating pH, catalyzing reactions, and driving ion transport.^[^ ^1^, ^2^, ^3^, ^4^, ^5^, ^6^, ^7^, ^8^, ^9^, ^10^, ^11^, ^12^, ^13^, ^14^, ^15^ ^]^ For example, ESPT results in a huge absorption‐emission energy gap in popular large Stokes shift fluorescent proteins and allows multicolor microscopy to visualize multiple cellular events.^[^ ^16^ ^]^ ESPT is also utilized in generating protons to catalyze series of chemical synthesis such as polymerization and self‐assembly of nanoparticles.^[^ ^7^, ^17^ ^]^ The reaction center is typically a photoacid that undergoes a p*K*_a_ drop upon photoexcitation.^[^ ^18^ ^]^ The mechanism is commonly described by a Förster cycle where ESPT becomes exergonic due to the altered energetics of acid (A form) and conjugate base (B form) in the electronic excited state (**Figure** **1**a).^[^ ^19^ ^]^ The ESPT driving force is conventionally measured by p*K*_a_^*^ (asterisk represents the excited state), reflective of free energy difference calculable by the Förster equation p*K*_a_^*^  =  p*K*_a_  −  ($E_{0-0}^{A}-E_{0-0}^{B}$)/(*k*_B_ *T*ln10), where $E_{0-0}^{A}$ and $E_{0-0}^{B}$ are the 0−0 transition gaps of acid and conjugate base, respectively.^[^ ^20^ ^]^ Although these definitions have qualitatively explained the kinetics for many photoacids, the lack of numerical precision and substantial sampling has led to considerable theoretical challenges.^[^ ^21^ ^]^ Current discrepancies between thermodynamics as estimated by Förster equation and the overly fast ESPT kinetics are remarkable for strong photoacids with short excited‐state lifetimes such as the green fluorescent protein (GFP) chromophore derivatives in solution, thus demanding more rigorous and fundamental insights.^[^ ^22^, ^23^ ^]^

Meanwhile, the intrinsic structure‐photoacidity correlations remain unclear and underexplored, although the photoacids of various frameworks have been investigated^[^ ^24^, ^25^, ^26^, ^27^, ^28^ ^]^ to reach a general understanding of photoacidity arising from the light‐induced intramolecular charge transfer (ICT).^[^ ^29^ ^]^ In our earlier work of the GFP chromophore‐based photoacids as prototypical functional systems with the ESPT reaction, we incorporated substituents to increase photoacidity, but no explicit relationships were formulated.^[^ ^27^ ^]^ In similar photobasic molecules, p*K*_a_ and p*K*_a_^*^ of quinoline derivatives were correlated to the electron‐withdrawing ability of a substituent using Hammett parameters,^[^ ^30^ ^]^ but a sole consideration of such intramolecular electronic effect resulted in a nonideal linear correlation for p*K*_a_^*^.^[^ ^31^ ^]^ Similar qualities of linear correlations have also been observed in the color tuning and isomerization barrier of chromophores, indicating the inadequacy of Hammett parameter alone in describing the substituent‐induced energetics in condensed phases.^[^ ^32^, ^33^, ^34^ ^]^

To provide deeper insights into ESPT, a systematic tuning of ESPT driving force is needed. For a typical phenol‐based photoacid, a *para*‐site electron acceptor (A*_p_*) ensures excited‐state ICT and hence photoacidity. The driving force can be varied by introducing an electron‐donating (D*_o_*) or accepting (A*_o_*) group *ortho* to the hydroxy group, or changing the electron‐accepting strength of A*_p_* (Figure 1b). In this work, we based our investigation on the model GFP chromophore, *p*‐hydroxybenzylidene‐dimethylimidazolinone (*p*‐HBDI), wherein the methine‐bridged imidazolinone (I‐)ring acts as A*_p_*. We synthesized new *p*‐HBDI derivatives with substituted phenol/phenolate (P‐) and I‐rings by electron‐donating (EDG) and electron‐withdrawing (EWG) groups (Figure 1c). These substitutions lead to absorption and emission shifts for the acid and conjugate base,^[^ ^34^ ^]^ thus tuning the ESPT driving force. We introduce an additive model to linearly correlate the 0−0 transition gap, p*K*_a_, Δp*K*_a_, and p*K*_a_^*^ of photoacid to substituent properties describing intramolecular electronic and steric as well as intermolecular H‐bonding effects. We found that the p*K*_a_^*^ calculated by free energy difference via the conventional Förster equation contradicts the observed ESPT kinetics due to a significantly underestimated driving force. This inconsistency results from an overlook of Franck‐Condon (FC) excess energy upon photoexcitation when ESPT is substantially fast. In contrast, the free energy difference remains valid as the ESPT driving force for fluorescent photoacids. Moreover, the thermodynamics‐kinetics relationship for ESPT follows the Bell‐Evans‐Polanyi principle.^[^ ^37^, ^38^ ^]^ Our findings crucially provide the power for predicting ESPT likelihood/rate to enable the rational design of functional photoacids across chemical, energy, and biological disciplines.

## 2 Results and Discussion

### 2.1 Origin of Photoacidity

**Figure 2:**
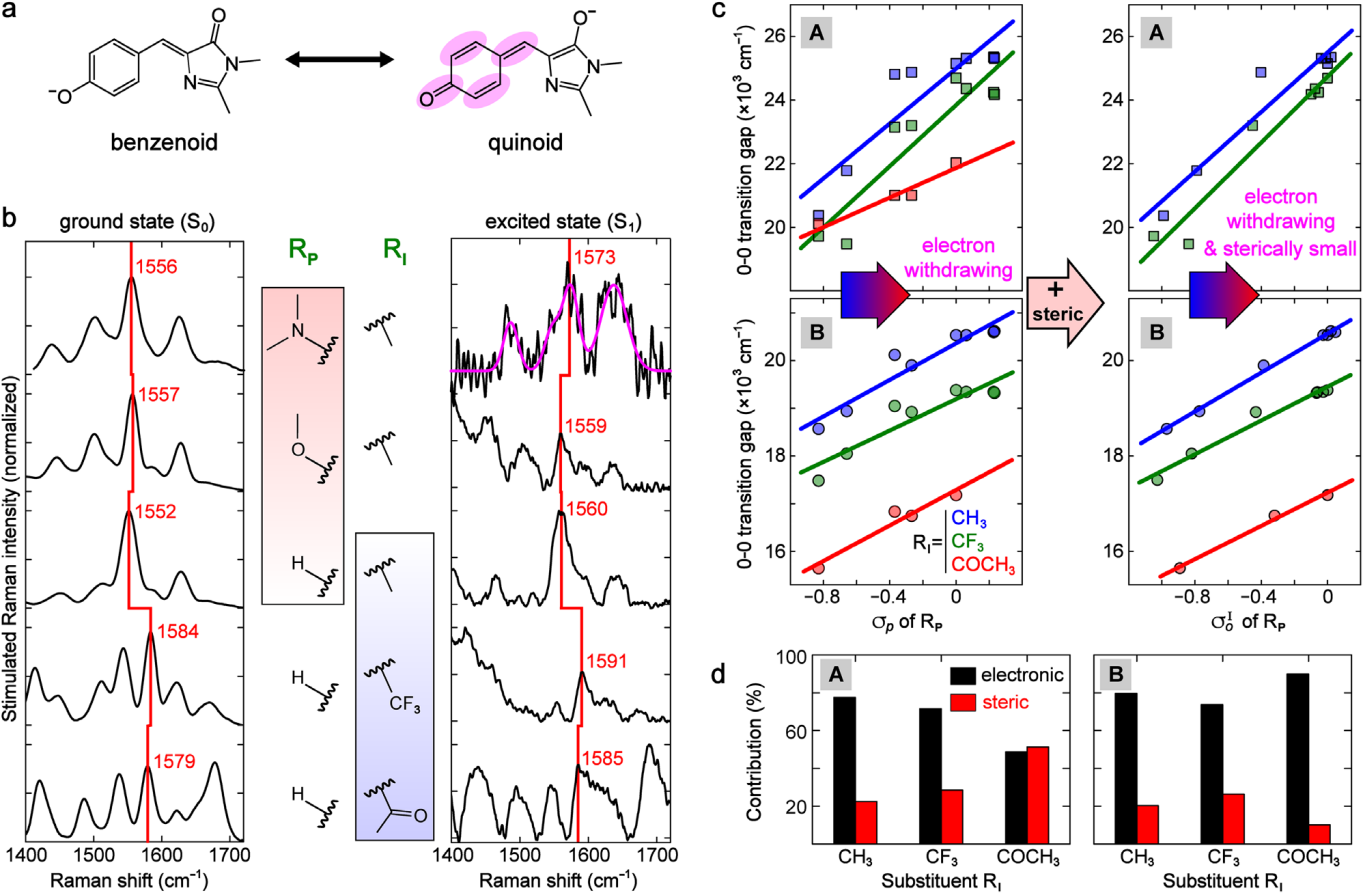
Color and energetics tuning mechanism of the GFP chromophore. a) Resonance structures of deprotonated *p*‐HBDI, whose alternating double‐bond stretching mode at ≈1552 cm^−1^ is marked by magenta ellipses. b) Ground‐ and excited‐state FSRS of the deprotonated *p*‐HBDI derivatives in acetonitrile. Red lines highlight frequency changes of the C=O/C=C stretching mode as the R_P_ and R_I_ substitutions red‐shift the chromophore color. The experimental conditions are provided in Section S3.3 and Table S1 (Supporting Information). The excited‐state FSRS spectra were chosen around time zero with maximal mode intensity (Figure S7, Supporting Information). The spectrum of **8a** (R_P_ =−NMe_2_, R_I_ = −CH_3_) was shown with the fitted trace in magenta due to low signal‐to‐noise ratio. c) Correlations between 0−0 transition gap in acetonitrile and substituent for the acid (A) and conjugate base (B). The R_P_ substituent −OH is excluded from linear regression due to its H‐bonding interaction with the solvent upon adding steric effects (right panels). The acid form of the R_I_ = −COCH_3_ series yields a drastically different slope (Table S6, Supporting Information) and is not shown in the upper right panel. d) Contributions by percentage of electronic (black) and steric (red) effects of R_P_ substituents to the change of 0−0 transition gaps with three R_I_ substituents in acetonitrile.

Typically, photoacidity originates from the excited‐state ICT that causes different energetics for the acid and conjugate base from ground states.^[^ ^29^, ^39^ ^]^ The photoacidity tuning is thus accompanied by color change. Besides computational insights (Figures S1 and S2, Supporting Information), we provided the experimental evidence of ICT using wavelength‐tunable femtosecond stimulated Raman spectroscopy (FSRS, experimental conditions in Table S1, Supporting Information) that can probe the ground‐to‐excited‐state electron redistribution with vibrational marker bands.^[^ ^40^ ^]^ The intense mode of the deprotonated *p*‐HBDI at 1552 cm^−1^ due to alternating C═O/C═C stretches (Figure S3, Supporting Information) displays correlated frequency patterns with the benzenoid and quinoid resonance structures^[^ ^41^, ^42^ ^]^ (**Figure** **2**a) due to the photoexcitation‐ or substitution‐induced charge redistribution. Alternating double‐bond stretches usually exhibit strong Raman intensity and have been used as indicators for the electron delocalization and color tuning of polyenes and visual pigments.^[^ ^43^, ^44^ ^]^ The blueshifts of this mode in *p*‐HBDI derivatives from electronic ground to excited states (Figure 2b; Figure S7, Supporting Information) directly evince the photoinduced ICT, reflecting a shift from the benzenoid to quinoid resonance structure. This key insight agrees with theoretical calculations (Figures S1 and S2, Supporting Information). Meanwhile, R_P_ and R_I_ substitutions shift the mode frequency to different extents in the ground and excited states, which implies their different energetics (Figures S4–S8, Supporting Information). These results support our assertion that ground‐ and excited‐state acidities can be tuned by the combination of R_P_ and R_I_ substituents on the molecular framework (Figure 1b). Notably, a similar indicator mode tracking charge redistribution has not been found for the protonated form, which may involve more complex resonance structures.^[^ ^45^ ^]^

### 2.2 Tuning Photoacid Energetics by Substituents

To quantify the substituent‐induced energetics, we first found that the 0−0 transition energy^[^ ^46^ ^]^ in acetonitrile and water (Figures S9–S14 and Tables S2 and S3, Supporting Information) generally exhibits a linear correlation to the *para* Hammett parameter σ*_p_* of R_P_ substituent (Figure 2c), indicative of dominant electronic effects. Notably, the lowering of 0−0 transition energy upon the acceptor substitution is not in line with σ*_p_* of R_I_ (−CF_3_ versus −COCH_3_) because −COCH_3_ expands the π‐conjugation upon excited‐state ICT and stabilizes the excited state more than −CF_3_ (Figures S1 and S2, Supporting Information).^[^ ^47^ ^]^ Discussions of the chromophore acidity appear below after the multivariable analysis (Figures S15‒S18 and Table S4, Supporting Information).

To delineate a more precise correlation, steric effect also needs to be considered for an *ortho* substituent.^[^ ^48^ ^]^ We linearly linked the 0−0 transition energy (Δ*E*_0−0_) to two variables, *para* Hammett parameter σ*_p_* and Charton parameter *V* (see Section S6 and Table S5, Supporting Information),^[^ ^30^, ^49^ ^]^ to quantitatively describe the electronic and steric effects exerted by the substituent, respectively:

(1) $$\Delta E_{0-0}=\rho \sigma_{p}+\mathrm{sV}+\Delta E_{0-0,H}$$

We consider that σ*_p_* and *V* describe intrinsic substituent properties regardless of the molecular framework; therefore, the respective coefficient informs the difference between two structures or states caused by the effect each parameter represents. Similar additive models were developed to explain reactivity‐substituent relationships for organic reactions, and later spawned the quantitative structure‐activity relationships (QSAR) with popular uses in biochemical and pharmaceutical fields (see Section S6, Supporting Information, for details).^[^ ^30^, ^50^ ^]^

With this model, we extracted R_P_ substituent's electronic and steric contributions to energetics of the acid and conjugate base via linear regression. The positive ρ and negative *s* indicate that electron‐donating and sterically large R_P_ substituent red‐shifts the photoacid: the larger magnitude (ρ) for electronic effect confirms its major role (see Figure 2d; Table S6, Supporting Information). The correlation can also be demonstrated via a single *ortho* parameter $\sigma_{o}^{I}$ combining two effects ($\sigma_{o}^{I}=\sigma_{p}+\frac{s}{\rho }V$). The improved linearity between Δ*E*_0−0_ and $\sigma_{o}^{I}$ justifies the role of *ortho* substituent's steric effect, albeit weaker than the electronic effect, in tuning the energetics of photoacids in various solvents (Figure 2c; Figure S20, Supporting Information). Meanwhile, the acid has larger‐magnitude coefficients than the conjugate base (Table S6, Supporting Information), so the acid is more susceptible to energy tuning,^[^ ^45^ ^]^ which contrasts the common belief that photoacidity arises from a stronger ICT and more stabilization in the conjugate base than the acid^[^ ^29^, ^39^ ^]^ and could inspire further experimental and theoretical investigations.

### 2.3 Tuning p*K*_a_, Δp*K*_a_, and p*K*_a_^*^ by Substituents

**Figure 3:**
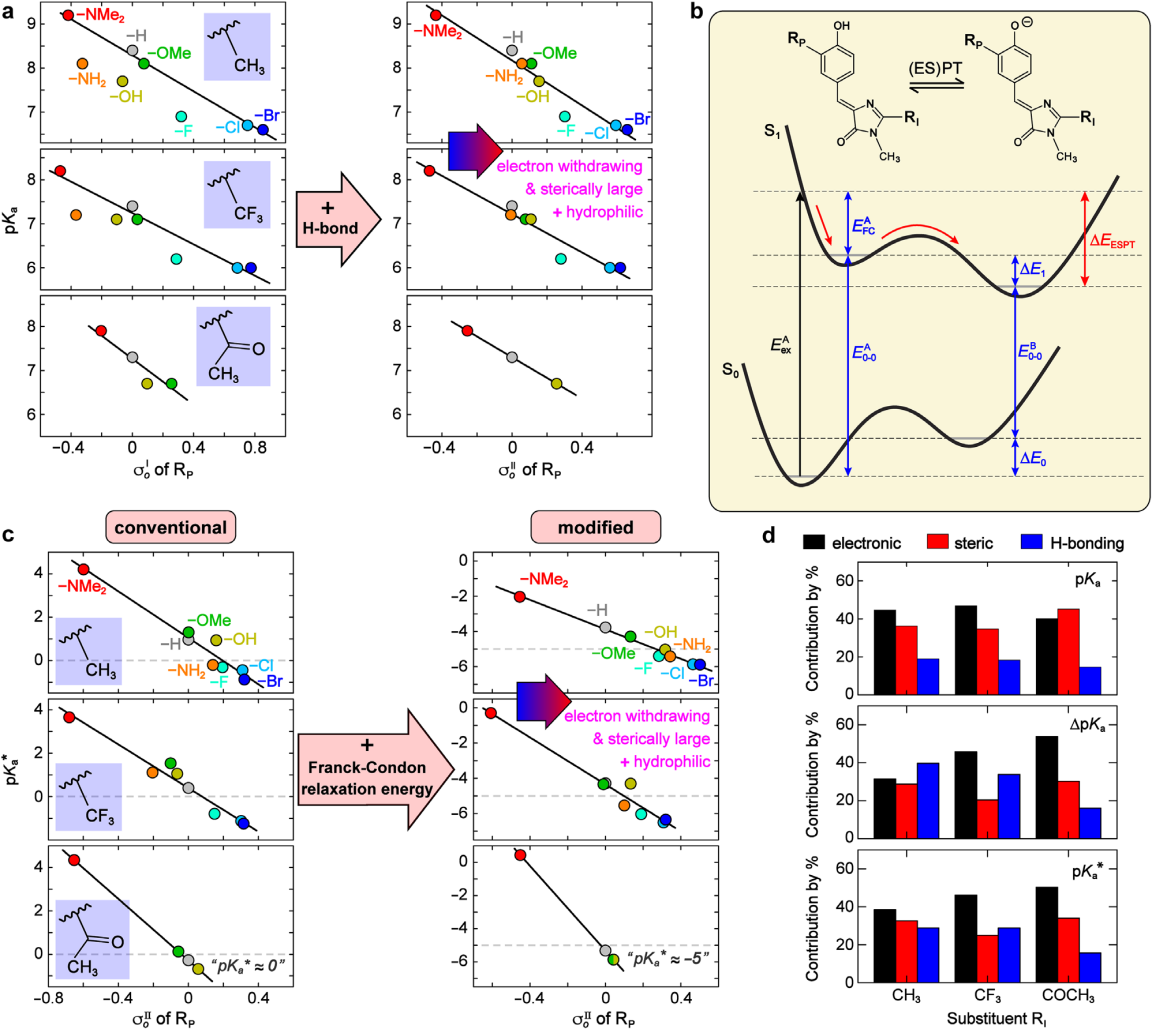
Ground‐ and excited‐state acidity tuning mechanisms of GFP chromophore in water. a) Correlations between p*K*_a_ and newly defined *ortho* parameters $\sigma_{o}^{I}$ and $\sigma_{o}^{\mathrm{II}}$. b) Energy diagram for ground‐state acid‐base equilibrium and ESPT. Energy abbreviations: $E_{\mathrm{ex}}^{A}$ > 0 (excitation), $E_{\mathrm{FC}}^{A}$ > 0 (A‐form Franck‐Condon excess), $E_{0-0}^{A}$ > 0 (A‐form 0−0 transition), $E_{0-0}^{B}$ > 0 (B‐form 0−0 transition), Δ*E*_0_ (ground‐state free energy difference: conjugate base minus acid), Δ*E*_1_ (excited‐state free energy difference: conjugate base minus acid), Δ*E*_ESPT_<0 (ESPT driving force: final minus initial state). c) Correlations between p*K*_a_^*^, calculated without and with the Franck‐Condon excess energy, and the *ortho* parameter $\sigma_{o}^{\mathrm{II}}$ of R_P_. The dashed line marks a threshold p*K*_a_^*^ value, above which ESPT was not observed. d) Contributions by percentage of electronic (black), steric (red), and intermolecular H‐bonding (blue) effects of R_P_ substituent to the changes of p*K*_a_, Δp*K*_a_, and p*K*_a_^*^.

We translate the same insights to p*K*_a_ and p*K*_a_^*^, assuming minimal entropy change (Δ*S*°) that contributes to free energy difference (Δ*G*°).^[^ ^51^ ^]^ Therefore, the tuning of p*K*_a_ and p*K*_a_^*^, calculated by Δ*G*°/(*RT*ln10) ≈ Δ*H*°/(*RT*ln10), is directly connected to energy changes by the R_P_ substitutions. By applying a similar equation, p*K_a_* =  ρσ_*p*_ + *sV* + p*K*_*a*,*H*_ or $pK_{a}=\rho \sigma_{o}^{I}+pK_{a,H}$ (Figures S16–S18, Supporting Information), an unsatisfactory linear correlation was observed with a few substituents (−NH_2_, −OH, −F) clearly deviating from the trend (**Figure** **3**a). We ascribe such deviations to ignoring intermolecular solute‐solvent interactions besides electronic (σ_*p*_) and steric (*V*) parameters that only characterize intramolecular effects. The “outlier” substituents like −NH_2_ and −OH can establish strong H‐bonds with the solvent (water) particularly as H‐bond donors, constituting intermolecular interactions. Consequently, the substituent hydrophobicity parameter (π, Table S5, Supporting Information)^[^ ^52^ ^]^ can be used to describe this interaction (Section S6, Supporting Information). The acidity‐substituent correlation is then given by:

(2) $$pK_{a}=\rho \sigma_{p}+\mathrm{sV}+p\pi +pK_{a,H}$$

Notably, this analysis yields an excellent linearity (Table S7, Supporting Information) and can be illustrated with the single parameter $\sigma_{o}^{\mathrm{II}}=\sigma_{p}+\frac{s}{\rho }V+\frac{p}{\rho }\pi$, hence $pK_{a}=\rho \sigma_{o}^{\mathrm{II}}+pK_{a,H}$ (Figure 3a). The greatly improved linear correlation for all substituents substantiates the pronounced role of intermolecular interactions in tuning the energetics for the ground‐state acid‐base equilibrium.

To correlate p*K*_a_^*^ to substituent properties, we first used the 0−0 transition gaps of the acid and conjugate base to compute Δp*K*_a_ and p*K*_a_^*^ by the Förster equation. Despite a good linearity, the resultant p*K*_a_^*^ values (from −1 to 4, Figure 3c) are too high to rationalize the kinetics: the GFP chromophore‐derived photoacids have miniscule fluorescence quantum yield (FQY≈10^−4^) due to facile nonradiative ring‐twisting pathways;^[^ ^53^ ^]^ therefore, the ESPT occurrence (for R_P_ substituents −F, −Cl, −Br, −NH_2_ in −CH_3_ and −CF_3_ series, and −OMe, −OH in −COCH_3_ series), corroborated by their fluorescence spectra in water (Figures S12–S15, Supporting Information), infers a faster or comparable ESPT rate versus ring‐twists on the sub‐picosecond timescale.^[^ ^23^, ^54^ ^]^ Such ultrafast ESPT, likely barrierless, is typically associated with p*K*_a_^*^ below −4 as predicted by Förster equation.^[^ ^21^ ^]^ These results insinuate that using the free energy difference as ESPT driving force for the unlocked, nonfluorescent photoacids is problematic. In the conventional scheme, ESPT is treated as a quasi‐equilibrium reaction, and the thermodynamic relation p*K_a_* =  Δ*G* ^○^/(*k_B_T·*ln10) is applicable. The quasi‐equilibrium assumption qualitatively fits fluorescent photoacids when ESPT is faster than excited‐state deactivation, but fails for nonfluorescent photoacids when other nonradiative pathways (such as ring‐twists) are substantially fast.^[^ ^23^, ^53^ ^]^

We note that thermal or quasi‐equilibrium signifies Boltzmann distribution wherein reactant and product species are populated predominantly at their lowest vibrational energy levels; however, photoacids are exclusively populated at FC region of the acid upon photoexcitation. For essentially nonfluorescent photoacids in this work, ESPT may occur faster than FC relaxation (typically hundreds of femtoseconds) and proceed directly from the FC region instead of from the lowest vibrational energy level, which would not be “thermally” populated before ESPT or act as the reactant state. Therefore, the ESPT driving force should be the energy difference between the FC vibrational levels and energy minimum of the conjugate base, thereby increasing the conventional free energy difference by the FC relaxation/excess energy ($E_{\mathrm{FC}}^{A}$) of the acid. This energy term has been omitted in previous ESPT models that worked well for strongly or moderately fluorescent photoacids.^[^ ^21^ ^]^ We hereby revise the ESPT driving force (Δ*E*_ESPT_<0) by including $E_{\mathrm{FC}}^{A}$ (positive) and the free energy difference (Δ*E*_1_, conjugate base minus acid) via $\Delta E_{\mathrm{ESPT}}=-E_{\mathrm{FC}}^{A}+\Delta E_{1}=-E_{\mathrm{ex}}^{A}+E_{0-0}^{B}+\Delta E_{0}$ (Figure 3b). The resultant p*K*_a_^*^ values calculated by Δ*E*_ESPT_/(*k_B_T*·ln10) can nicely predict the ESPT occurrence. For example, the large $E_{\mathrm{FC}}^{A}$ values of ESPT‐capable derivatives (R_P_ = −NH_2_, R_I_ = −CH_3_ or −CF_3_; R_P_ = −OMe and −OH, R_I_ = −COCH_3_) reveal the significant underestimation of their p*K*_a_^*^ values computed by conventional Δ*E*_1_ that suggest no ESPT reaction (Figure 3c; Table S4, Supporting Information). The p*K*_a_^*^ values calculated by the Stokes shift exhibit a similar trend (except for R_P_ = −NMe_2_) due to the “intrinsic” inclusion of $E_{\mathrm{FC}}^{A}$ (Table S4, Supporting Information). Notably, the p*K*_a_^*^ is used as a proxy of the excited‐state energy difference that essentially drives the ESPT reaction, which should not be taken literally since the ground‐state p*K*_a_ has conventionally been used by the community to describe an equilibrium reaction.

Under this new definition, Δp*K*_a_ and p*K*_a_^*^ also demonstrate linear correlations (Equation (2)) to substituent properties (Figure 3c). The slopes for Δp*K*_a_ and p*K*_a_^*^ become larger with R_I_ substituent from −CH_3_ to −CF_3_ to −COCH_3_, in contrast to p*K*_a_ whose slope remains similar regardless of the R_I_ substituent. This finding indicates that the acceptor plays a more important role in the excited state due to photoinduced ICT, while in the ground state its effect is weaker. It also implies that p*K*_a_ can be effectively tuned by substitution at the donor moiety near a dissociable −OH group, while p*K*_a_^*^ is sensitive to both donor and acceptor substitutions.^[^ ^27^ ^]^ For p*K*_a_ and p*K*_a_^*^, the parameters (σ_*p*_, *V*, π) show (−,−,+) signs, respectively (Table S7, Supporting Information), implying that the electron‐withdrawing, sterically large, and hydrophilic R_P_ substituents can enhance the ground‐ and excited‐state acidities (Figure 3a,c). The prominence of each factor depends on the R_I_ substituent and varies between p*K*_a_ and p*K*_a_^*^ (Figure 3d). In general, all three factors make comparable contributions, while the intramolecular effects add up to a larger impact (σ_*p*_ + *V* > π). The intermolecular effect (π) plays a minor role in p*K*_a_ tuning for all three R_I_ series as well as p*K*_a_^*^ tuning for −COCH_3_ series.

### 2.4 Thermodynamics‐Kinetics Relationship for ESPT

**Figure 4:**
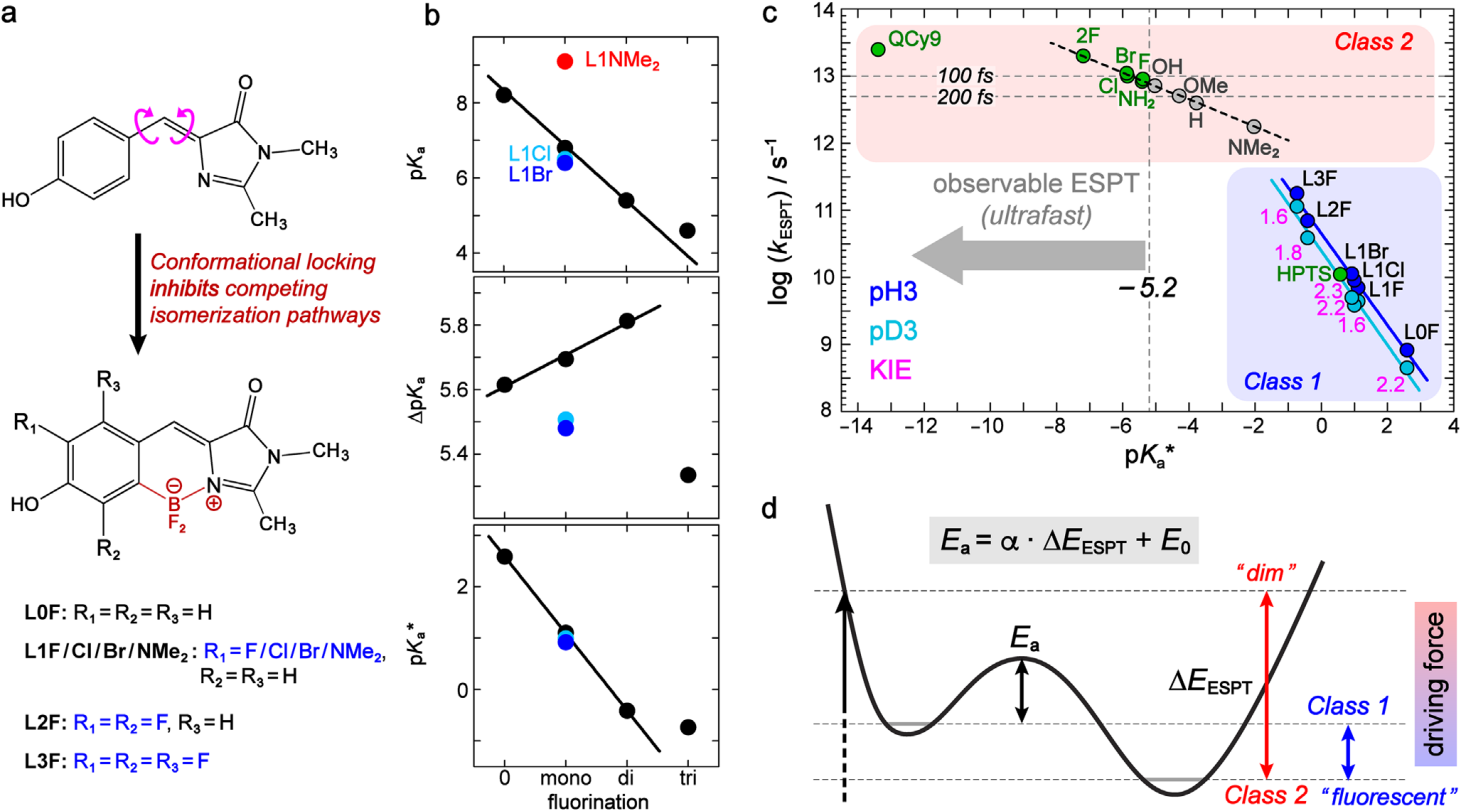
Thermodynamics‐kinetics relationships for ESPT. a) Modifications of GFP chromophore into fluorescent photoacids (L*n*F, *n* = 0–3; L1Cl, L1Br, L1NMe_2_) by conformational locking and fluorination. b) Correlations between p*K*_a_, Δp*K*_a_, p*K*_a_^*^, and fluorination. The tuning of p*K*_a_, Δp*K*_a_, and p*K*_a_^*^ exhibits additivity by fluorination and linear trends for mono‐ and di‐fluorination, indicating that the two *ortho* sites to the electron‐donating −OH/−O^−^ group are electronically similar. The accumulative fluorination changes the p*K*_a_ and p*K*_a_^*^ more than the single substitution by different halogens (−F, −Cl, −Br). The Δp*K*_a_ and p*K*_a_^*^ of L1NMe_2_ are not shown here due to the absence of ESPT (Table S9, Supporting Information). c) Correlations between base‐10 logarithmic ESPT rate and p*K*_a_^*^ value. The ESPT rates of L*n*F/L1Cl/L1Br are verified by the isotopic kinetic effect (KIE). Aqueous solutions under the same pH(= 3) and pD(= 3) conditions are measured to keep the ground‐state protonated form populated and ensure the identical proton‐accepting capability for all photoacids. Three reported photoacids (QCy9, 2F, HPTS) are plotted (green circles) with their ESPT rates taken from literature. The p*K*_a_^*^ values of the nonfluorescent QCy9 and 2F are calculated with FC excess energy, while that of the highly fluorescent HPTS is estimated by 0–0 transition gaps (Table S10, Supporting Information). The abbreviated NH_2_ is the photoacid (R_P_ = −NH_2_, R_I_ = −CH_3_; green circle). The abbreviated −F, −Cl, −Br are photoacids with −CH_2_COOH group added at the N‐3 position to increase solubility in acidic water (R_P_ = −F, −Cl, −Br, R_I_ = −CH_3_; green circles) for transient absorption measurements. The other substituents in −CH_3_ series with log(*k*_ESPT_) deduced from Bell‐Evans‐Polanyi principle are shown by gray circles. Their p*K*_a_^*^ values were calculated by including the FC excess energy (Tables S4 and S10, Supporting Information). The horizontal dashed lines denote ESPT time constants (100 and 200 fs) for the unlocked *p*‐HBDI derivatives as references to predict the ESPT occurrence (*k*_ESPT_ in the unit of s^−1^). d) Illustration of the BEP principle in an ESPT reaction.

Our new understanding of ESPT explains the unusually fast ESPT kinetics of previously reported GFP chromophore‐derived nonfluorescent superphotoacids due to an underestimated driving force. These photoacids undergo barrierless or nearly barrierless ESPT, which is faster than FC relaxation, hence necessitating the inclusion of excess vibrational energy upon photoexcitation in driving ESPT reaction. When ESPT is slower than FC relaxation, the photoacid would be predominantly populated at the lowest vibrational level. The knowledge about ESPT in this case falls into the conventional scheme with the driving force dictated by the free energy difference; this class of photoacids usually has a relatively long excited‐state lifetime with moderate or strong fluorescence, constituting the photoacids in literature with rather small driving forces and slow ESPT rates.^[^ ^18^, ^21^ ^]^ We thus investigated a series of fluorescent photoacids L*n*F (*n*  =  0–3), L1Br, L1Cl, and L1NMe_2_ by conformationally locking the GFP chromophore to inhibit nonradiative ring‐twisting pathways (**Figure** **4**a).^[^ ^27^ ^]^ The acidity (Figure S19, Supporting Information) and photoacidity are varied greatly by accumulative fluorination but slightly between different haolgens (Figure 4b), while the Δp*K*_a_ (5.3 to 5.8) and p*K*_a_^*^ (−1 to 3, Table S9, Supporting Information) are estimated with free energy difference via 0–0 transition gaps to correlate with the observed ESPT kinetics. Notably, we were unable to synthesize certain locked compounds with identical R_P_ and R_I_ substituents to the unlocked series due to the synthetic challenges (Section S1.3, Supporting Information). Nevertheless, the five singly substituted (−H, −F, −Cl, −Br, −NMe_2_) locked chromophores still enable us to perform the multivariable analysis and reveal similar insights into the acidity/photoacidity‐substituent relationships as the unlocked series (Figure S21 and Table S8, Supporting Information).

We implemented femtosecond transient absorption (fs‐TA) spectroscopy to obtain the ESPT rate constants (*k*_ESPT_) for L*n*F, L1Br, and L1Cl in water (pH = 3) as confirmed by the slowdown of ESPT in deuterated water (Figures S22–S24 and Table S9, Supporting Information). L1NMe2 as previously reported^[^ ^28^ ^]^ is incapable of ESPT (Figure S24, Supporting Information). The picosecond (ps) to nanosecond (ns) ESPT time constants from L3F to L0F (5.6 ps to 1.2 ns) correspond to p*K*_a_^*^ of −1 to 3. Other fluorescent photoacids in literature also fall into this range such as HPTS (p*K*_a_^*^ ≈ 0.6 and τ_ESPT_ ≈ 90 ps, Table S10, Supporting Information).^[^ ^21^ ^]^ Notably, the logarithmic *k*_ESPT_ demonstrates a linear correlation with p*K*_a_^*^ (Figure 4c), reflective of the Bell‐Evans‐Polanyi (BEP) principle that states a linear correlation between the activation energy and enthalpy change within the same family, *E*_a_ = *E*_0_  + αΔ*H*. The α coefficient (0 ≤ α ≤ 1) characterizes the transition state position along a reaction coordinate.^[^ ^37^, ^38^, ^55^, ^56^ ^]^ The linearity between log(*k*_ESPT_) and p*K*_a_^*^ validates free energy difference as the ESPT driving force for fluorescent photoacids, which also indicates that BEP principle applies to ESPT reactions: *E*_a_ = *E*_0_  + α · Δ*E*_ESPT_ or $\log\left(k_{\mathrm{ESPT}}\right)=\;\log\left(k_{0}\right)-\alpha \cdot pK_{a}^{*}$ (Figure 4d). The fitted α value of ≈0.68 for the locked photoacids suggests a product‐like transition state. To examine if the BEP principle also applies to the nonfluorescent photoacids, we had to slightly modify the structures due to their poor solubilities in acididic water (i.e., insufficient sample concentrations for fs‐TA measurements). The −CH_2_COOH group was thus added to the N‐3 position of the three halogenated (−F, −Cl, −Br) molecules of the −CH_3_ series, and their ESPT rate constants were then obtained by fs‐TA data (Figure S25 and Table S10, Supporting Information). As a result, a clear linear correlation between the logarithmic *k*_ESPT_ and p*K*_a_^*^ was also retrieved for the nonfluorescent photoacids (Figure 4c), supporting the BEP principle. Notably, the linear BEP relationship challenges current ESPT formalism based on Hynes’ and Marcus’ proton transfer theories, which establish the nonlinear thermodynamics‐kinetics relationships (Section S7.3, Supporting Information).^[^ ^21^ ^]^

Our results reveal that the driving force for fluorescent (Class 1) and nonfluorescent (Class 2) photoacids should be treated differently in calculating the p*K*_a_^*^ to account for their kinetics. For corroboration, we plotted a number of other reported photoacids in Figure 4c: HPTS, QCy9, and 2F (Figure S26, Supporting Information).^[^ ^22^, ^25^, ^57^ ^]^ For Class 1 photoacids, the thermodynamics‐kinetics relationship of HPTS aligns with the L*n*F series, supporting the p*K*_a_^*^ values that can be estimated by the free energy difference. For Class 2 photoacids, especially those undergoing faster ESPT than FC relaxation (e.g., QCy9 and 2F), the FC excess energy adds an extra driving force to ESPT and results in much lower p*K*_a_^*^ values, thereby satisfactorily rationalizing their ultrafast ESPT. Furthermore, their deviations from the trend of L*n*F confirm that structural similarity of molecules is prerequisite for applying the BEP principle.

Within the same family of photoacids, the BEP principle can be used to estimate ESPT rate by structural modification and predict ESPT occurrence. For example, the photoacids 2F (difluorinated *p*‐HBDI), NH_2_ (R_P_ = −NH_2_, R_I_ = −CH_3_), and F/Cl/Br (R_P_ = −F/−Cl/−Br, R_I_ = −CH_3_ with −CH_2_COOH at the N‐3 position; Figure S25, Supporting Information) presumably meet BEP principle to derive the slope and intercept for these unlocked *p*‐HBDI derivatives, and the “intrinsic” log(*k*_ESPT_) for various substituents can then be deduced for the −CH_3_ series (Figure 4c). The protonated *p*‐HBDI is known to have an excited‐state lifetime below 100 fs in water,^[^ ^53^ ^]^ which should hold for its derivatives with R_P_ substituents and extremely weak fluorescence. This property results in a high threshold for substantial ESPT due to the kinetic ultrafast competition between ESPT and ring twists.^[^ ^23^, ^54^ ^]^ The ESPT occurrence for substituents (−F, −Cl, −Br, −NH_2_) infers that the thermodynamic and kinetic thresholds for an observable ESPT reaction are −5.2 (p*K*_a_^*^) and 5–10 ps^−1^ (*k*_ESPT_), respectively (i.e., the excited‐state lifetime is 100–200 fs, marked by dashed lines in Figure 4c). Therefore, the accurate kinetic prediction of ESPT using the BEP relationship should be system‐dependent and requires a general fundamental understanding of the excited‐state decay dynamics within the same family of photoacids (as demonstrated above).

## 3 Conclusion

In summary, the acidity and photoacidity of GFP chromophore as an archetypal and versatile photoacid were strategically tuned by comparative series of chemical substitutions to unveil the controlling factors. The photoacidity arises from the excited‐state ICT causing different energetics for the acid and conjugate base, tracked by frequency shifts of vibrational marker bands in FSRS. We devised a linear relationship correlating the chromophore energetics and photoacidity to substituent's electronic and steric effects, thus providing key insights into the photoacidity‐structure relationships. We further showed that ESPT driving force or p*K*_a_^*^ for fluorescent and nonfluorescent photoacids should be treated or calculated differently. In the latter case, ESPT may be faster than FC relaxation and occur directly from FC region; we revised the p*K*_a_^*^ definition by adding the FC excess energy of the acid to the free energy difference, which rationalizes the observed ESPT kinetics for nonfluorescent photoacids in this work and literature. Meanwhile, the conventional Förster equation based on the free energy difference remains valid for fluorescent photoacids with ESPT rates slower than FC relaxation. The thermodynamics‐kinetics relationship is unveiled to follow the Bell‐Evans‐Polanyi principle, which enables the ESPT reaction prediction.

Notably, “FC excess energy” is a complicated term due to the multidimensional excited‐state potential energy surface while we used the apparent (inhomogeneously broadened) electronic absorption maximum to calculate it as a holistic representation. Although it is shown to be an effective approximation to model nonfluorescent photoacids, our current findings have certain limitations. For instance, these unlocked photoacids exhibit an excitation‐depedent ESPT but the trend is not as simply expected (Figure S15, Supporting Information). The discrepancy may be rationalizable by steady‐state spectral measurements implying the excitation‐dependent competing pathways such as ring twists or internal conversion (Figure S27, Supporting Information), and a precise determination of ESPT rate requires more sophisticated studies with analytical precision. Our work thus focuses on the conceptual development with crucial fundamental insights into the ESPT theory covering structure‐energy, structure‐acidity, and thermodynamics‐kinetics relationships, which can effectively guide the rational design of functional photoacid systems (e.g., enzymes, pumps, catalysts, dyes, probes, sensors) for a myriad of chemical and biological advances, as well as current hot areas from photolithography, photocatalysis, to carbon capture.^[^ ^58^, ^59^, ^60^, ^61^ ^]^

## Conflict of Interest

The authors declare no conflict of interest.

## Supporting information



Supporting Information

## Data Availability

The data that support the findings of this study are available in the supplementary material of this article.
