# Supplementary material for: Electronic and Steric Tuning of Molecular Acidity toward Unified Models for Excited State Proton Transfer
Source: Adv Sci (Weinh). 2025 Oct 13;13(4):e17140. doi: 10.1002/advs.202517140 (PMC12822464; doi:10.1002/advs.202517140)
Supplement: Supplementary file 1 — Supporting Information [file ADVS-13-e17140-s001.pdf]

Supporting Information (SI) for:

Electronic and Steric Tuning of Molecular Acidity toward Unified Models for Excited State Proton Transfer

Cheng Chen,[1] Ivan N. Myasnyanko,[2] Mikhail S. Baranov,[2,3] and Chong Fang[1]*

[1]Department of Chemistry, Oregon State University, 153 Gilbert Hall, Corvallis, Oregon 97331, United States

[2]Institute of Bioorganic Chemistry, Russian Academy of Sciences, Miklukho-Maklaya 16/10, Moscow 117997, Russia

[3]Pirogov Russian National Research Medical University, Ostrovitianov 1, Moscow 117997, Russia

*To whom correspondence should be addressed. E-mail: Chong.Fang@oregonstate.edu

Table of Contents

S1. Synthesis of photoacid compounds S4

S2. DFT calculations and color tuning of GFP chromophores S23

Figure S1. Calculated energetics for photoacids in the protonated form S25

Figure S2. Calculated energetics for photoacids in the deprotonated form S26

S3. FSRS reveals mechanistic insights into photoacid energetics S27

Figure S3. Calculated vibrational motions for the alternating C=O/C=C stretch mode S30

Figure S4. Ground-state FSRS and calculated spectra for $–CH_3$ series S31

Figure S5. Ground-state FSRS and calculated spectra for $–CF_3$ series S32

Figure S6. Ground-state FSRS and calculated spectra for $–COCH_3$ series S34

Figure S7. Excited-state FSRS spectra for photoacids with various R_P and R_I substitutions in acetonitrile S35

Figure S8. Correlation between Raman peak frequency and R_P/R_I substituents S36

Table S1. Experimental conditions for FSRS measurements. S37

S4. Electronic absorption, emission, and 0–0 transition gap S39

Figure S9. Absorption and emission spectra for the $–CH_3$ series in acetonitrile S41

Figure S10. Absorption and emission spectra for the $–CF_3$ series in acetonitrile S42

Figure S11. Absorption and emission spectra for the $–COCH_3$ series in acetonitrile S43

Figure S12. Absorption and emission spectra for the $–CH_3$ series in water S44

Figure S13. Absorption and emission spectra for the $–CF_3$ series in water S45

Figure S14. Absorption and emission spectra for the $–COCH_3$ series in water S46

Figure S15. Excitation-dependent steady-state fluorescence spectra of several series of photoacids with spectral decomposition S47

Table S2. Absorption/emission maxima and 0-0 transition gaps of the protonated neutral and deprotonated anionic *p*-HBDI chromophore derivatives in acetonitrile. S49

Table S3. Absorption/emission maxima and 0-0 transition gaps of the protonated neutral and deprotonated anionic *p*-HBDI chromophore derivatives in water. S50

S5. Determination of pK_a, ΔpK_a and pK_a^* S51

Figure S16. pK_a titration for the $–CH_3$ series in water S54

Figure S17. pK_a titration for the $–CF_3$ series in water S55

Figure S18. pK_a titration for the $–COCH_3$ series in water S56

Figure S19. pK_a titration for the locked photoacids in water S57

Table S4. pK_a, ΔpK_a, pK_a^* values using different methods for unlocked photoacids in water. ... S58

S6. Multivariable linear models S59

Figure S20. Correlations between optical properties and substituent parameters for the unlocked (nonfluorescent) photoacids in water S62

Figure S21. Correlations between optical properties and substituent parameters for the locked (fluorescent) photoacids in water S63

Table S5. Electronic, steric, and hydrophobic parameters for multivariable linear analysis. S64

Table S6. Multivariable linear regression for the 0-0 transition energy gaps of the unlocked *p*-HBDI derivatives. S65
Table S7. Multivariable linear regression for pK_a, ΔpK_a, and pK_a^* of the unlocked *p*-HBDI derivatives in water. S67
Table S8. Multivariable linear regression for pK_a, ΔpK_a, and pK_a^* of the locked photoacids[a] in water S69
S7. ESPT kinetics and Bell-Evans-Polanyi principle S70
Figure S22. Fs-TA spectra and global analysis for the fluorescent (locked) photoacids in pH 3 aqueous solutions (H_2O) S76
Figure S23. Fs-TA spectra and global analysis for the fluorescent (locked) photoacids in pD 3 aqueous solutions (D_2O) S78
Figure S24. Electronic absorption and emission spectra of the fluorescent (locked) photoacids in H_2O and D_2O S79
Figure S25. Fs-TA spectra and global analysis for the nonfluorescent (unlocked) photoacids in aqueous solution S82
Figure S26. Electronic absorption and emission spectra of some other reported photoacids S83
Figure S27. Comparison of the electronic absorption and excitation spectra of the nonfluorescent photoacids in methanol. S84
Table S9. Acidities and kinetics of the locked fluorescent photoacids. S85
Table S10. Acidities and kinetics of several nonfluorescent and reported photoacids in aqueous solution S87
S8. References S88
S9. Appendix (^{1}H and ^{13}C NMR spectra of new compounds) S94

S1. Synthesis of photoacid compounds

The commercially available reagents were used without additional purification. E. Merck Kieselgel 60 was used for column chromatography. Thin layer chromatography (TLC) was performed on silica gel 60 F254 glass-backed plates (MERCK). Visualization was effected by UV light (254 or 312 nm) and staining with $KMnO_4$.

The NMR spectra were recorded on a 700 MHz Bruker Avance III NMR spectrometer at 303 K, Bruker Fourier 300 spectrometer, and a 800 MHz Bruker Avance III NMR spectrometer at 303 K. Chemical shifts were reported relative to residue peaks of DMSO-d_6 (2.50–2.51 ppm for ^{1}H, and 39.5 ppm for ^{13}C) or $CDCl_3$ (77.0 ppm for ^{13}C). Melting points were measured on a SMP 30 apparatus (Cole-Parmer Stuart). High-resolution mass spectra (HRMS) were recorded on a LTQ Orbitrap Elite (ThermoScientific) using electrospray ionization (ESI). The measurements were conducted in a positive ion mode (interface capillary voltage at –5000 V) or in a negative ion mode (3500 V); the interface temperature was set at 275 °C.

S1.1 Synthesis of the –CH_3 and –$COCH_3$ series.

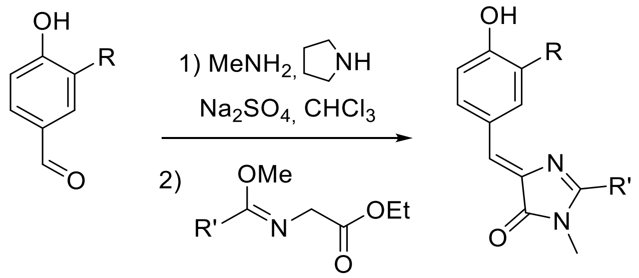

Typical synthetic procedure for imidazolones with methyl or ethyl groups

The corresponding aromatic aldehyde (10 mmol) was dissolved in $CHCl_3$ (50 mL) and mixed with methylamine solution (40% aqueous, 2.5 mL), pyrrolidine (7 mg, 0.1 mmol) and anhydrous Na_2SO_4 (10 g). The mixture was stirred for 48 h at room temperature, filtered and dried over the additional Na_2SO_4. The solvent was evaporated and ethyl((1-methoxypropylidene)amino)acetate or ethyl((1-methoxyethylidene)amino)acetate (20 mmol) was added to the residue (5–10 mL of methanol was also added if it would not mix well). The mixture was then stirred for 24 h at room temperature, solvents were evaporated, and the product was purified by column chromatography (using the eluent of $CHCl_3$-EtOH at v/v of 95/5). The syntheses for the compounds with R group of –H, –F, –Cl, –Br, –OH, –OMe, and –NH_2 and R' group of –CH_3, as well as the compound with R group of –H and R' group of –CH_2CH_3, were previously described (see below).

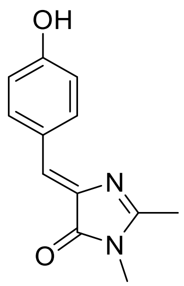

(*Z*)-5-(4-hydroxybenzylidene)-2,3-dimethyl-3,5-dihydro-4*H*-imidazol-4-one (1a)

The synthetic route has been previously described.[1]

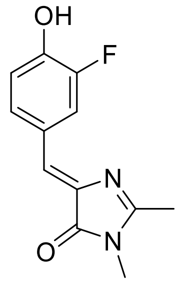

(*Z*)-5-(3-fluoro-4-hydroxybenzylidene)-2,3-dimethyl-3,5-dihydro-4*H*-imidazol-4-one (2a)

The synthetic route has been previously described by us.[2]

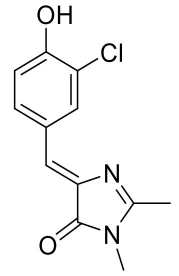

(*Z*)-5-(3-chloro-4-hydroxybenzylidene)-2,3-dimethyl-3,5-dihydro-4*H*-imidazol-4-one (3a)

The synthetic route has been previously described by us.[3]

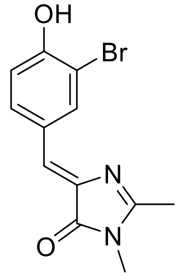

(*Z*)-5-(3-bromo-4-hydroxybenzylidene)-2,3-dimethyl-3,5-dihydro-4*H*-imidazol-4-one (4a)

The synthetic route has been previously described.[4]

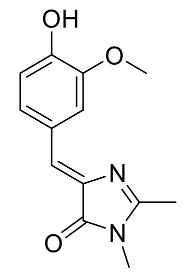

(*Z*)-5-(4-hydroxy-3-methoxybenzylidene)-2,3-dimethyl-3,5-dihydro-4*H*-imidazol-4-one (5a)

The synthetic route has been previously described.[5]

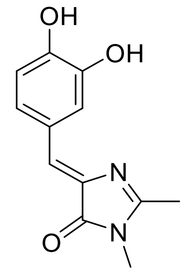

(*Z*)-5-(3,4-dihydroxybenzylidene)-2,3-dimethyl-3,5-dihydro-4*H*-imidazol-4-one (6a)

The synthetic route has been previously described by us.[6]

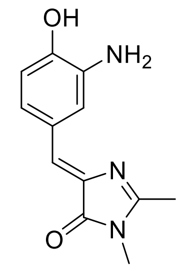

(*Z*)-5-(3-amino-4-hydroxybenzylidene)-2,3-dimethyl-3,5-dihydro-4*H*-imidazol-4-one (7a)

The synthetic route has been previously described.[6]

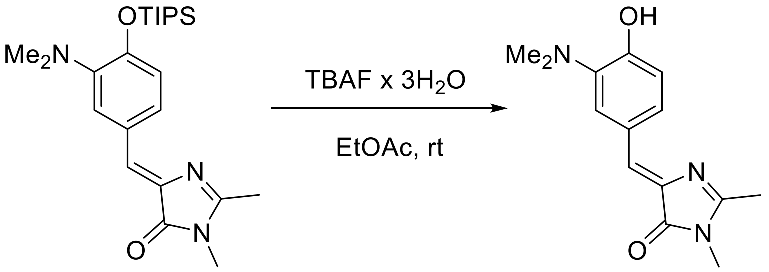

(*Z*)-5-(3-(dimethylamino)-4-hydroxybenzylidene)-2,3-dimethyl-3,5-dihydro-4*H*-imidazol-4-one (8a)

The precursor compound (*Z*)-5-(3-(dimethylamino)-4-((triisopropylsilyl)oxy)benzylidene)-2,3-dimethyl-3,5-dihydro-4*H*-imidazol-4-one as reported previously[7] (100 mg, 0.24 mmol) and tributylammonium fluoride (TBAF) trihydrate (190 mg, 0.60 mmol, 2.5 eq) was dissolved in EtOAc (10 mL). The mixture was stirred for 1 h at room temperature, neutralized with 50 μL AcOH, washed with brine (5 × 5 mL) and dried over Na_2SO_4. The solvent was evaporated and the residue was then purified by column chromatography (eluent $CHCl_3$-EtOH at v/v of 10/1).

Dark yellow solid (55 mg, 89% yield); m.p. = 136–138 °C; ^{1}H NMR (700 MHz, 303 K, DMSO-d_6) δ (ppm) = 9.79 (br. s., 1 H), 7.85 (d, J = 1.4 Hz, 1 H), 7.68 (dd, J = 8.3, 1.4 Hz, 1 H), 6.86 (s, 1 H), 6.81 (d, J = 8.3 Hz, 1 H), 3.08 (s, 3 H), 2.70 (s, 6 H), 2.32 (s, 3 H) (see Appendix S1); ^{13}C NMR (75 MHz, 303 K, DMSO-d_6) δ (ppm) = 169.9, 161.9, 152.5, 141.0, 136.1, 127.5, 126.2, 125.8, 122.2, 115.8, 42.6 (2C), 26.2, 15.4 (see Appendix S2); HRMS (ESI) m/z: 260.1389 was found (calculated for $C_{14}H_{18}N_3O_2^+$, $[M+H]^+$ 260.1394).

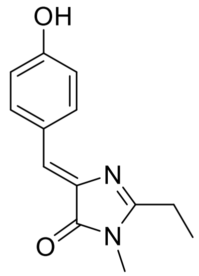

(*Z*)-5-(4-hydroxybenzylidene)-2-ethyl-3-methyl-3,5-dihydro-4*H*-imidazol-4-one

The synthetic route has been previously described.[8]

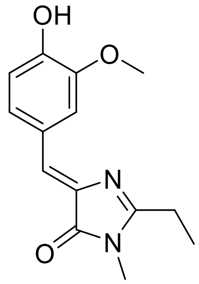

(*Z*)-5-(4-hydroxy-3-methoxybenzylidene)-2-ethyl-3-methyl-3,5-dihydro-4*H*-imidazol-4-one

Yellow solid (2.45 g, 94% yield); m.p. = 149–152 °C; ^{1}H NMR (700 MHz, 303 K, DMSO-d_6) δ (ppm) = 9.8–9.6 (bs, 1H), 8.17 (d, J = 1.3 Hz, 1H), 7.56 (dd, J = 8.2, 1.3 Hz, 1H), 6.90 (s, 1H), 6.83 (d, J = 8.2 Hz, 1H), 3.81 (s, 3H), 3.08 (s, 3H), 2.67 (q, J = 7.4 Hz, 2H), 1.27 (t, J = 7.4 Hz, 3H) (see Appendix S3); ^{13}C NMR (176 MHz, 303 K, $CDCl_3$) δ (ppm) = 170.0, 165.6, 149.1, 147.5,

136.3, 126.8, 125.9, 125.8, 115.5, 115.2, 55.3, 25.9, 21.2, 8.8 (see Appendix S4); HRMS (ESI) m/z: 261.1234 was found (calculated for $C_{14}H_{17}N_2O_3^+$, $[M+H]^+$ 261.1239).

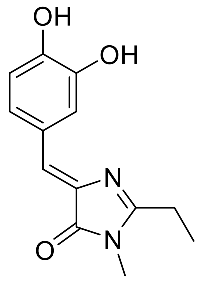

(*Z*)-5-(3,4-dihydroxybenzylidene)-2-ethyl-3-methyl-3,5-dihydro-4*H*-imidazol-4-one

Red solid (540 mg, 22% yield); m.p. — over 250 °C with decomposition; ^{1}H NMR (700 MHz, 303 K, DMSO-d_6) δ (ppm) = 9.7–9.3 (bs, 1H), 9.3–8.9 (bs, 1H), 7.85 (d, J = 1.7 Hz, 1H), 7.40 (dd, J = 8.2, 1.7 Hz, 1H), 6.80 (s, 1H), 6.78 (d, J = 8.2 Hz, 1H), 3.08 (s, 3H), 2.67 (q, J = 7.4 Hz, 2H), 1.27 (t, J = 7.4 Hz, 3H) (see Appendix S5); ^{13}C NMR (176 MHz, 303 K, $CDCl_3$) δ (ppm) = 170.2, 165.5, 148.5, 145.3, 136.2, 126.2, 125.9, 125.8, 118.8, 115.7, 26.0, 21.4, 9.4 (see Appendix S6); HRMS (ESI) m/z: 247.1077 was found (calculated for $C_{13}H_{15}N_2O_3^+$, $[M+H]^+$ 247.1083).

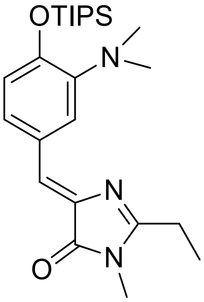

(*Z*)-5-(3-(dimethylamino)-4-((triisopropylsilyl)oxy)benzylidene)-2-ethyl-3-methyl-3,5-dihydro-4*H*-imidazol-4-one

Yellow solid (3.2 g, 75% yield); m.p. = 155–156 °C; ^{1}H NMR (700 MHz, 303 K, DMSO-d_6) δ (ppm) = 8.16 (d, J = 1.7 Hz, 1H), 7.59 (dd, J = 8.3, 1.7 Hz, 1H), 6.90 (s, 1H), 6.81 (d, J = 8.3 Hz, 1H), 3.08 (s, 3H), 2.73 (s, 6H), 2.67 (q, J = 7.3 Hz, 2H), 1.31 (quin, J = 7.5 Hz, 3H), 1.27 (t, J = 7.3 Hz, 3H), 1.08 (d, J = 7.5 Hz, 18H) (see Appendix S7); ^{13}C NMR (176 MHz, 303 K, $CDCl_3$) δ (ppm) = 171.1, 164.4, 151.4, 145.0, 136.6, 128.1, 127.9, 127.3, 122.4, 119.9, 43.0 (2C), 26.3, 22.1, 17.9 (6C), 13.0 (3C), 9.2 (see Appendix S8); HRMS (ESI) m/z: 430.2874 was found (calculated for $C_{24}H_{40}N_3O_2Si^+$, $[M+H]^+$ 430.2884).

Preparation of (*Z*)-5-benzylidene-2-acetyl-3-methyl-3,5-dihydro-4*H*-imidazol-4-ones

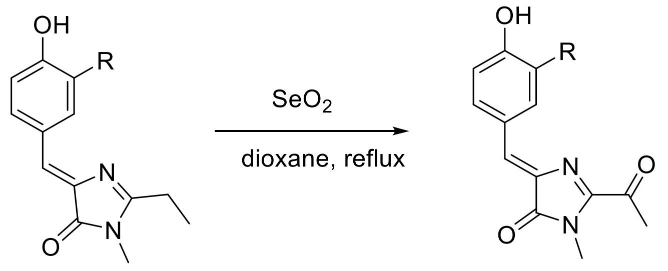

The corresponding 5-(*Z*)-benzylidene-2-ethyl-3-methyl-3,5-dihydro-4*H*-imidazol-4-one (1 mmol) and selenium dioxide (0.13 g, 1.2 mmol) were suspended in dioxane (30 mL). The mixture was refluxed for 1 h, the solvent was removed *in vacuo* and the crude product was dissolved in EtOAc (200 mL). The solution was washed by water (2 × 35 mL) and brine (2 × 35 mL), then dried over Na_2SO_4. The solvent was evaporated and crude product was purified by column chromatography (eluent $CHCl_3$-EtOH at v/v of 95/5).

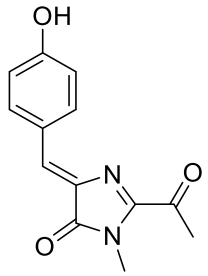

(*Z*)-5-(4-hydroxybenzylidene)-2-acetyl-3-methyl-3,5-dihydro-4*H*-imidazol-4-one (1c)

Red solid (81 mg, 33% yield); m.p. — over 200 °C with decomposition; ^{1}H NMR (700 MHz, 303 K, DMSO-d_6) δ (ppm) = 10.5 (bs, 1H), 8.23 (d, J = 8.8 Hz, 2H), 7.38 (s, 1H), 6.91 (d, J = 8.8 Hz, 2H), 3.29 (s, 3H), 2.62 (s, 3H) (see Appendix S9); ^{13}C NMR (75 MHz, 303 K, DMSO-d_6) δ (ppm) = 192.9, 169.6, 161.5, 153.6, 135.9, 135.4, 134.3, 125.0, 116.3, 28.3, 26.3 (see Appendix S10); HRMS (ESI) m/z: 243.0775 was found (calculated for $C_{13}H_{11}N_2O_3^-$, [M–H]$^-$ 243.0770).

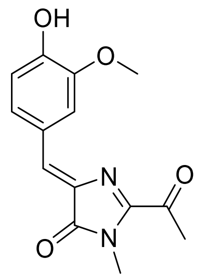

(*Z*)-5-(4-hydroxy-3-methoxybenzylidene)-2-acetyl-3-methyl-3,5-dihydro-4*H*-imidazol-4-one (5c)

Red solid (58 mg, 21% yield); m.p. — over 200 °C with decomposition; ^{1}H NMR (700 MHz, 303 K, DMSO-d_6) δ (ppm) = 10.2 (br. s., 1H), 8.24 (d, J = 1.2 Hz, 1H), 7.68 (dd, J = 8.2, 1.2 Hz, 1H), 7.39 (s, 1H), 6.91 (d, J = 8.2 Hz, 1H), 3.85 (s, 3 H), 3.30 (s, 3 H), 2.63 (s, 3H) (see Appendix S11); ^{13}C NMR (75 MHz, 303 K, DMSO-d_6) δ (ppm) = 192.8, 169.6, 153.4, 151.3, 147.9, 135.4, 134.5, 129.3, 125.4, 115.9, 115.6, 55.3, 28.3, 26.2 (see Appendix S12); HRMS (ESI) m/z: 273.0880 was found (calculated for $C_{14}H_{13}N_2O_4^-$, [M–H]$^-$ 273.0875).

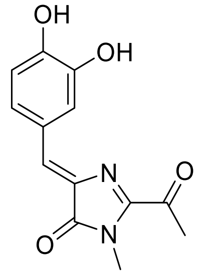

(*Z*)-5-(3,4-dihydroxybenzylidene)-2-acetyl-3-methyl-3,5-dihydro-4*H*-imidazol-4-one (6c)

Red solid (13 mg, 5% yield); m.p. — over 250 °C with decomposition; ^{1}H NMR (700 MHz, 303 K, DMSO-d_6) δ (ppm) = 10.1 (bs, 1H), 9.5 (bs, 1H), 8.03 (d, J = 1.9 Hz, 1H), 7.50 (dd, J = 8.2, 1.9 Hz, 1H), 7.28 (s, 1H), 6.85 (d, J = 8.2 Hz, 1H), 3.29 (s, 3H), 2.65 (s, 3H) (see Appendix S13); ^{13}C NMR (75 MHz, 303 K, DMSO-d_6) δ (ppm) = 192.9, 169.6, 153.3, 150.7, 145.8, 135.2, 134.8, 128.3, 125.4, 119.3, 115.9, 28.3, 26.3 (see Appendix S14); HRMS (ESI) m/z: 259.0724 was found (calculated for $C_{13}H_{11}N_2O_4^-$, [M–H]$^-$ 259.0719).

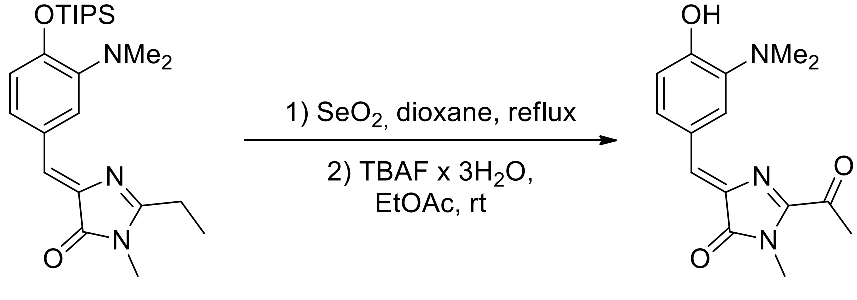

(*Z*)-5-(3-(dimethylamino)-4-hydroxybenzylidene)-2-acetyl-3-methyl-3,5-dihydro-4*H*-imidazol-4-one (8c)

The aforementioned (*Z*)-5-(3-(dimethylamino)-4-((triisopropylsilyl)oxy)benzylidene)-2-ethyl-3-methyl-3,5-dihydro-4*H*-imidazol-one (300 mg, 0.70 mmol) and selenium dioxide (93 mg, 0.84 mmol, 1.2 eq) were suspended in dioxane (30 mL). The mixture was refluxed for 1 h and then

cooled to room temperature. The mixture was filtered through a pad of Celite®, and the pad was washed with dioxane (60 mL). The solvent was then evaporated and the residue was dissolved in EtOAc (50 mL) and tributylammonium fluoride (TBAF) trihydrate (230 mg, 0.73 mmol, 2.5 eq) was added. The mixture was stirred for 1 h at room temperature. 50 µL of acetic acid was added and the mixture was washed with brine (8 × 5 mL) and dried over Na_2SO_4. The solvent was evaporated and the residue was purified by column chromatography (eluent $CHCl_3$-EtOH at v/v of 30/1).

Red solid (58 mg, 29% yield); m.p. — over 200 °C with decomposition; ^{1}H NMR (700 MHz, 303 K, DMSO-d_6) δ (ppm) = 10.4 (br. s., 1H), 8.22 (s, 1H), 7.63 (d, J = 8.4 Hz, 1H), 7.35 (s, 1H), 6.87 (d, J = 8.4 Hz, 1H), 3.30 (s, 3H), 2.75 (s, 6H), 2.62 (s, 3H) (see Appendix S15); ^{13}C NMR (176 MHz, 303 K, DMSO-d_6) δ (ppm) = 192.6, 169.5, 154.6, 153.1, 141.5, 135.0, 134.8, 130.1, 125.4, 122.4, 116.0, 42.3 (2C), 28.2, 26.0 (see Appendix S16); HRMS (ESI) m/z: 288.1346 was found (calculated for $C_{15}H_{18}N_3O_3^+$, $[M+H]^+$ 288.1348).

General synthetic procedure for arylidene-imidazolones with an ester group: (*Z*)-ethyl-2-(5-(4-hydroxybenzylidene)-2-methyl-3,5-dihydro-4*H*-imidazol-4-one-3-yl)acetate

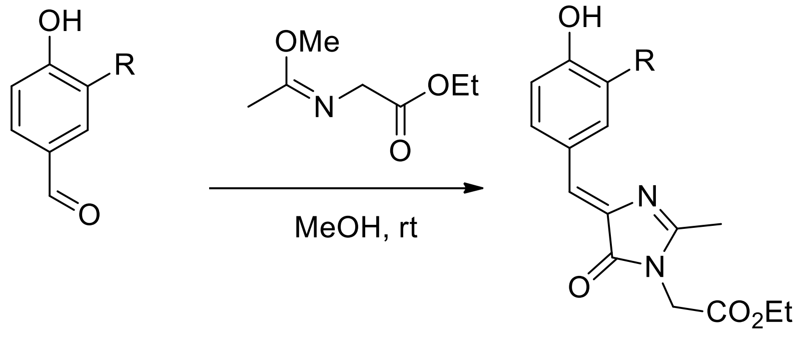

The corresponding aromatic aldehyde (2 mmol) was dissolved in MeOH (10 mL) and mixed with ethyl (*Z*)-2-((1-methoxyethylidene)amino)acetate (5 mmol). The solution was stirred for 24 h at room temperature, then the solvent was evaporated. The crude product was diluted with Et_2O solvent (5 mL), the precipitate was filtered, washed with Et_2O (3 × 3 mL), and dried under reduced pressure.

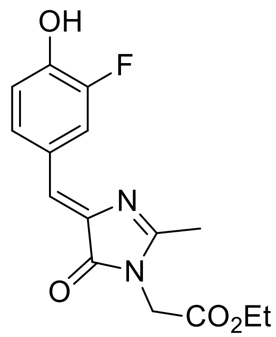

(*Z*)-ethyl-2-(5-(3-fluoro-4-hydroxybenzylidene)-2-methyl-3,5-dihydro-4*H*-imidazol-4-one-3-yl)acetate

Yellow solid (257 mg, 42% yield); m.p. = 168–169 °C; ^{1}H NMR (800 MHz, 303 K, DMSO-d_6) δ (ppm) = 10.63 (br. s., 1 H), 8.22 (dd, *J* = 12.9, 1.7 Hz, 1 H), 7.81 (dd, *J* = 8.5, 1.5 Hz, 1 H), 7.03 (t, *J* = 8.8 Hz, 1 H), 6.98 (s, 1 H), 4.51 (s, 2 H), 4.19 (q, *J* = 7.1 Hz, 2 H), 2.32 (s, 3 H), 1.24 (t, *J* = 7.1 Hz, 3 H) (see Appendix S17); ^{13}C NMR (75 MHz, 303 K, DMSO-d$_6$) δ (ppm) = 169.2, 168.1, 162.0, 150.7 (d, *J* = 241.0 Hz), 147.4 (d, *J* = 12.7 Hz), 136.4, 129.9 (d, *J* = 2.2 Hz), 125.7 (d, *J* = 7.2 Hz), 125.2 (d, *J* = 2.8 Hz), 119.0 (d, *J* = 19.3 Hz), 117.8 (d, *J* = 3.3 Hz), 61.4, 41.2, 15.1, 14.0 (see Appendix S18); HRMS (ESI) m/z: 307.1093 was found (calculated for $C_{15}H_{16}FN_2O_4^+$, $[M+H]^+$ 307.1089).

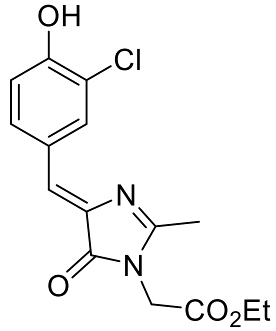

(*Z*)-ethyl-2-(5-(3-chloro-4-hydroxybenzylidene)-2-methyl-3,5-dihydro-4*H*-imidazol-4-one-3-yl)acetate

Light brown solid (406 mg, 63% yield); m.p. = 155 °C with decomposition; ^{1}H NMR (800 MHz, 303 K, DMSO-d_6) δ (ppm) = 10.93 (br. s., 1 H), 8.39 (d, *J* = 1.9 Hz, 1 H), 7.99 (dd, *J* = 8.6, 1.9 Hz, 1 H), 7.06 (d, *J* = 8.5 Hz, 1 H), 6.97 (s, 1 H), 4.52 (s, 2 H), 4.19 (q, *J* = 7.1 Hz, 2 H), 2.33 (s, 3 H), 1.24 (t, *J* = 7.1 Hz, 3 H) (see Appendix S19); ^{13}C NMR (75 MHz, 303 K, DMSO-d_6) δ (ppm) = 169.2, 168.1, 162.1, 155.1, 136.4, 133.3, 132.7, 126.3, 124.9, 120.2, 116.7, 61.4, 41.2, 15.1, 14.0

(see Appendix S20); HRMS (ESI) m/z: 323.0798 was found (calculated for $C_{15}H_{16}ClN_2O_4^+$, $[M+H]^+$ 323.0794).

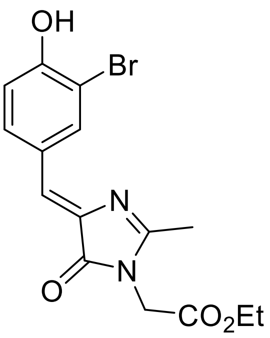

(*Z*)-ethyl-2-(5-(3-bromo-4-hydroxybenzylidene)-2-methyl-3,5-dihydro-4*H*-imidazol-4-one-3-yl)acetate

Yellow solid (404 mg, 55% yield); m.p. = 173–174 °C; ^{1}H NMR (800 MHz, 303 K, DMSO-d_6) δ (ppm) = 11.00 (s, 1 H), 8.54 (d, *J* = 1.9 Hz, 1 H), 8.04 (dd, *J* = 8.6, 1.9 Hz, 1 H), 7.04 (d, *J* = 8.5 Hz, 1 H), 6.97 (s, 1 H), 4.52 (s, 2 H), 4.20 (q, *J* = 7.1 Hz, 2 H), 1.25 (t, *J* = 7.1 Hz, 3 H) (see Appendix S21); ^{13}C NMR (75 MHz, 303 K, DMSO-d_6) δ (ppm) = 169.2, 168.1, 162.1, 156.1, 136.4 (2 C), 133.3, 126.7, 124.7, 116.4, 109.8, 61.4, 41.2, 15.1, 14.0 (see Appendix S22); HRMS (ESI) m/z: 367.0291 was found (calculated for $C_{15}H_{16}BrN_2O_4^+$, $[M+H]^+$ 367.0288).

General synthetic procedure for arylidene-imidazolyl-acetic acids: (*Z*)-2-(5-(4-hydroxybenzylidene)-2-methyl-3,5-dihydro-4*H*-imidazol-4-one-3-yl)acetic acid

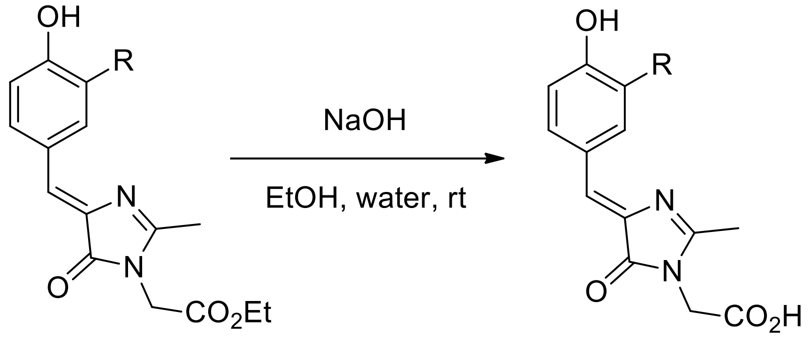

The corresponding arylidene-imidazolone with an ester group (1 mmol) was dissolved in EtOH (10 mL). The solution of NaOH (4 mmol) in water (3 mL) was then added. The resulting mixture was stirred for 1.5 h at room temperature and diluted with water (50 mL). Then, NaH_2PO_4 was added until pH 4 was reached. The aqueous layer was extracted with EtOAc (3 × 50 mL), the

organic layer was separated, dried over Na_2SO_4, and the solvent was evaporated under reduced pressure. The crude product was diluted with Et_2O (5 mL), the precipitate was filtered, washed with Et_2O (3 × 3 mL), and dried under reduced pressure.

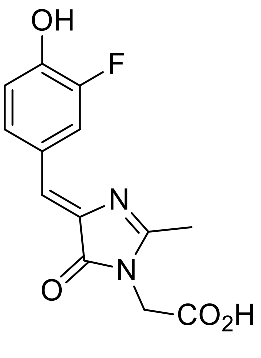

(*Z*)-2-(5-(3-fluoro-4-hydroxybenzylidene)-2-methyl-3,5-dihydro-4*H*-imidazol-4-one-3-yl)acetic acid

Light brown solid (220 mg, 79% yield); m.p. = 192 °C with decomposition; ^{1}H NMR (800 MHz, 303 K, DMSO-d_6) δ (ppm) = 13.23 (br. s., 1 H), 10.58 (br. s., 1 H), 8.20 (dd, J = 12.9, 1.7 Hz, 1 H), 7.79 (dd, J = 8.4, 1.6 Hz, 1 H), 7.01 (t, J = 8.8 Hz, 1 H), 6.95 (s, 1 H), 4.39 (s, 2 H), 2.30 (s, 3 H) (see Appendix S23); ^{13}C NMR (75 MHz, 303 K, DMSO-d_6) δ (ppm) = 169.5, 169.3, 162.4, 150.7 (d, J = 241.0 Hz), 147.3 (d, J = 12.7 Hz), 136.6, 129.8 (d, J = 2.2 Hz), 125.8 (d, J = 7.2 Hz), 124.9 (d, J = 2.2 Hz), 119.0 (d, J = 19.3 Hz), 117.7 (d, J = 3.3 Hz), 41.3, 15.2 (see Appendix S24); HRMS (ESI) m/z: 279.0779 was found (calculated for $C_{13}H_{12}FN_2O_4^+$, $[M+H]^+$ 279.0776).

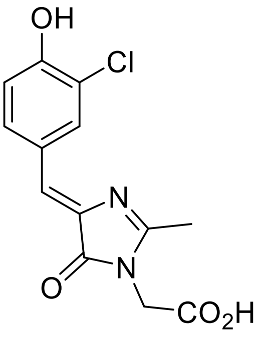

(*Z*)-2-(5-(3-chloro-4-hydroxybenzylidene)-2-methyl-3,5-dihydro-4*H*-imidazol-4-one-3-yl)acetic acid

Orange solid (274 mg, 93% yield); m.p. = 173 °C with decomposition; ^{1}H NMR (800 MHz, 303 K, DMSO-d_6) δ (ppm) = 11.03 (br. s., 1 H), 8.37 (d, J = 1.7 Hz, 1 H), 7.96 (dd, J = 8.5, 1.5 Hz, 1 H), 7.04 (d, J = 8.5 Hz, 1 H), 6.88 (s, 1 H), 4.10 (br. s., 2 H), 2.29 (s, 3 H) (see Appendix S25);

^{13}C NMR (75 MHz, 303 K, DMSO-d_6) δ (ppm) = 169.5, 169.3, 162.4, 155.0, 136.6, 133.3, 132.6, 126.4, 124.6, 120.2, 116.7, 41.3, 15.2 (see Appendix S26); HRMS (ESI) m/z: 295.0485 was found (calculated for $C_{13}H_{12}ClN_2O_4^+$, $[M+H]^+$ 295.0481).

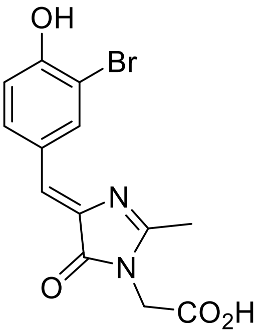

(*Z*)-2-(5-(3-bromo-4-hydroxybenzylidene)-2-methyl-3,5-dihydro-4*H*-imidazol-4-one-3-yl)acetic acid

Orange solid (325 mg, 96% yield); m.p. = 180 °C with decomposition; ^1H NMR (800 MHz, 303 K, DMSO-d_6) δ (ppm) = 13.21 (br. s., 1 H), 10.97 (s, 1 H), 8.52 (d, J = 1.9 Hz, 1 H), 8.02 (dd, J = 8.5, 1.9 Hz, 1 H). 7.03 (d, J = 8.5 Hz, 1 H), 6.94 (s, 1 H), 4.39 (s, 2 H), 2.31 (s, 3 H) (see Appendix S27); ^{13}C NMR (75 MHz, 303 K, DMSO-d_6) δ (ppm) = 169.5, 169.3, 162.4, 156.0, 136.6, 136.4, 133.2, 126.8, 124.5, 116.4, 109.8, 41.3, 15.2 (see Appendix S28); HRMS (ESI) m/z: 338.9979 was found (calculated for $C_{13}H_{12}BrN_2O_4^+$, $[M+H]^+$ 338.9975).

S1.2 Synthesis of the –CF_3 series.

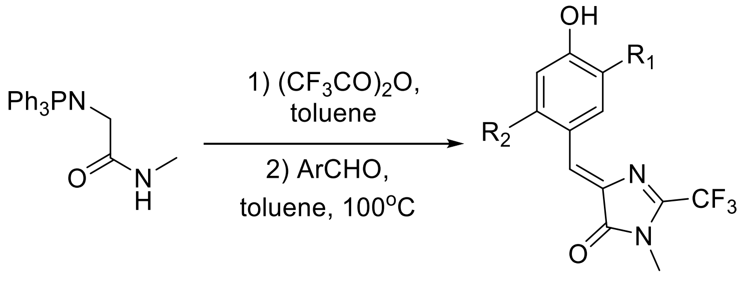

General method for the preparation of (*Z*)-5-benzylidene-3-methyl-2-(trifluoromethyl)-3,5-dihydro-4*H*-imidazol-4-ones

N-methyl-2-((triphenyl-λ^5-phosphanylidene)amino)acetamide (12.5 mmol) was degassed by three vacuum/argon cycles. Then toluene (25 mL) and trifluoroacetic anhydride (15 mmol) were added

and the mixture was stirred at room temperature until the complete dissolution (for about 40 min). Subsequently, the corresponding aldehyde (10 mmol) was added and the mixture was stirred at 100 °C for 3–6 hours. The mixture was dissolved in EtOAc (150 mL) and washed by potassium phosphate buffer (1 M, 30 mL) and brine (2 × 30 mL), followed by drying over anhydrous Na_2SO_4. The solvent was then evaporated and the remaining product was purified by column chromatography (using the $CHCl_3$-EtOH eluent at v/v of 95/5) as we reported previously.[9]

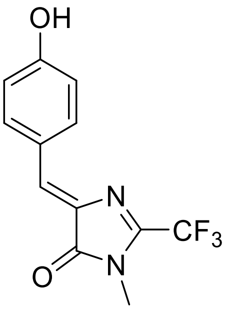

(*Z*)-5-(4-hydroxybenzylidene)-3-methyl-2-(trifluoromethyl)-3,5-dihydro-4*H*-imidazol-4-one (1b)

Yellow solid (0.50 g, 18% yield); m.p. ≈ 240 °C with decomposition; ^{1}H NMR (700 MHz, DMSO-d_6) δ (ppm) = 10.53 (s, 1H), 8.14 (d, J = 8.8 Hz, 2H), 7.43 (s, 1H), 6.91 (d, J = 8.8 Hz, 2H), 3.22 (s, 3H) (see Appendix S29); ^{13}C NMR (176 MHz, DMSO-d_6) δ (ppm) = 168.4, 161.7, 148.4 (app. d., J = 39.8 Hz), 135.7, 134.7, 133.1, 124.2, 117.7 (q, J = 273.1 Hz), 116.3, 27.0 (see Appendix S30); HRMS (ESI) m/z: 269.0576 was found (calculated for $C_{12}H_8F_3N_2O_2^-$, $[M–H]^-$ 269.0543).

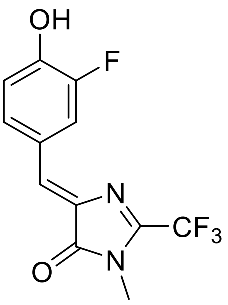

(*Z*)-5-(3-fluoro-4-hydroxybenzylidene)-3-methyl-2-(trifluoromethyl)-3,5-dihydro-4*H*-imidazol-4-one (2b)

Orange solid (0.9 g, 40% yield); m.p. = 231–233 °C; ^{1}H NMR (700 MHz, DMSO-d_6) δ (ppm) = 11.00 (s, 1 H), 8.16 (dd, J = 12.8, 1.9 Hz, 1 H), 7.90 (dd, J = 8.6, 1.7 Hz, 1 H), 7.44 (s, 1 H), 7.07 (t, J = 8.8 Hz, 1 H), 3.22 (s, 3 H) (see Appendix S31); ^{13}C NMR (201 MHz, DMSO-d_6) δ (ppm) = 168.3, 150.8 (d, J = 242.0 Hz), 149.4 (q, J = 38.1 Hz), 149.2 (d, J = 12.6 Hz), 134.2, 133.3,

131.4, 124.7 (d, $J = 7.1$ Hz), 119.9 (d, $J = 18.9$ Hz), 118.1 (d, $J = 2.4$ Hz), 117.6 (q, $J = 272.9$ Hz), 27.0 (see Appendix S32); HRMS (ESI) m/z: 287.0449 was found (calculated for $C_{12}H_7F_4N_2O_2^-$, $[M–H]^-$ 287.0449).

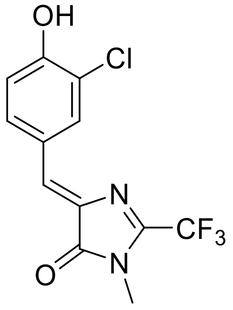

(*Z*)-5-(3-chloro-4-hydroxybenzylidene)-3-methyl-2-(trifluoromethyl)-3,5-dihydro-4*H*-imidazol-4-one (3b)

Orange solid (1.0 g, 35% yield); m.p. = 202–204 °C; ^{1}H NMR (700 MHz, DMSO-d_6) δ (ppm) = 11.30 (s, 1 H), 8.35 (d, $J = 1.9$ Hz, 1 H), 8.07 (dd, $J = 8.6$, 1.9 Hz, 1 H), 7.43 (s, 1 H), 7.09 (d, $J = 8.6$ Hz, 1 H), 3.22 (s, 3 H) (see Appendix S33);; ^{13}C NMR (176 MHz, DMSO-d_6) δ (ppm) = 168.4, 156.8, 149.5 (q, $J = 38.2$ Hz), 134.6, 134.3, 133.9, 133.0, 125.4, 120.6, 117.6 (q, $J = 273.1$ Hz), 117.1, 27.0 (see Appendix S34);; HRMS (ESI) m/z: 303.0154 was found (calculated for $C_{12}H_7ClF_3N_2O_2^-$, $[M–H]^-$ 303.0154).

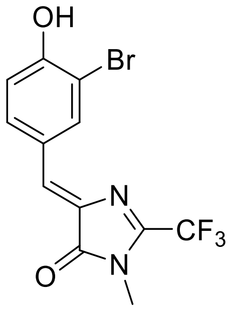

(*Z*)-5-(3-bromo-4-hydroxybenzylidene)-3-methyl-2-(trifluoromethyl)-3,5-dihydro-4*H*-imidazol-4-one (4b)

Orange solid (1.1 g, 31% yield); m.p. = 196–198 °C; ^{1}H NMR (700 MHz, DMSO-d_6) δ (ppm) = 11.36 (s, 1 H), 8.50 (d, $J = 1.9$ Hz, 1 H), 8.11 (dd, $J = 8.6$, 1.9 Hz, 1 H), 7.43 (s, 1 H), 7.08 (d, $J = 8.6$ Hz, 1 H), 3.21 (s, 3 H) (see Appendix S35); ^{13}C NMR (176 MHz, DMSO-d_6) δ (ppm) = 168.3, 157.7, 149.4 (q, $J = 38.5$ Hz), 137.8, 134.4, 134.1, 132.9, 125.8, 117.6 (q, $J = 273.1$ Hz),

116.7, 110.1, 27.0 (see Appendix S36); HRMS (ESI) m/z: 346.9649 was found (calculated for $C_{12}H_7BrF_3N_2O_2^-$, $[M–H]^-$ 346.9648).

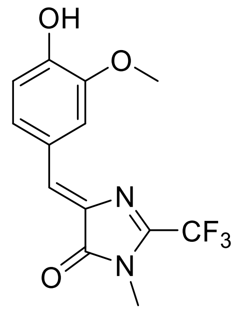

(*Z*)-5-(4-hydroxy-3-methoxybenzylidene)-3-methyl-2-(trifluoromethyl)-3,5-dihydro-4*H*-imidazol-4-one (5b)

Yellow solid (0.74 g, 25% yield); m.p. = 186–188 °C; ^{1}H NMR (700 MHz, DMSO-d_6) δ (ppm) = 10.21 (s, 1 H), 8.00 (d, J = 1.7 Hz, 1 H), 7.74 (dd, J = 8.4, 1.7 Hz, 1 H), 7.42 (s, 1 H), 6.91 (d, J = 8.2 Hz, 1 H), 3.81 (s, 3 H), 3.22 (s, 3 H) (see Appendix S37); ^{13}C NMR (176 MHz, DMSO-d_6) δ (ppm) = 168.6, 151.6, 148.5 (q, J = 38.2 Hz), 148.0, 135.1, 133.4, 129.0, 124.8, 117.7 (q, J = 273.1 Hz), 116.3, 116.1, 55.6, 27.1 (see Appendix S38); HRMS (ESI) m/z: 299.0643 was found (calculated for $C_{13}H_{10}F_3N_2O_3^-$, $[M–H]^-$ 299.0649).

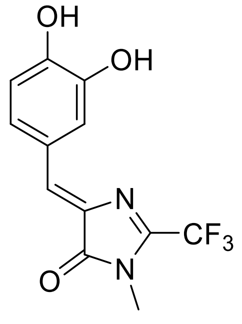

(*Z*)-5-(3,4-dihydroxybenzylidene)-3-methyl-2-(trifluoromethyl)-3,5-dihydro-4*H*-imidazol-4-one (6b)

Yellow solid (0.57 g, 20% yield); m.p. = 230–233 °C; ^{1}H NMR (700 MHz, DMSO-d_6) δ (ppm) = 10.08 (br. s., 1 H), 9.44 (br. s., 1 H), 7.87 (d, J = 1.9 Hz, 1 H), 7.49 (dd, J = 8.2, 1.9 Hz, 1 H), 7.32 (s, 1 H), 6.85 (d, J = 8.2 Hz, 1 H), 3.22 (s, 3 H) (see Appendix S39); ^{13}C NMR (176 MHz, DMSO-d_6) δ (ppm) = 168.4, 150.9, 148.1 (q, J = 38.1 Hz), 145.7, 135.2, 132.9, 128.1, 124.7, 119.1, 117.7 (q, J = 273.1 Hz), 115.9, 27.0 (see Appendix S40); HRMS (ESI) m/z: 285.0493 was found (calculated for $C_{12}H_8F_3N_2O_3^-$, $[M–H]^-$ 285.0493).

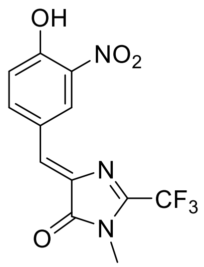

(*Z*)-5-(4-hydroxy-3-nitrobenzylidene)-3-methyl-2-(trifluoromethyl)-3,5-dihydro-4*H*-imidazol-4-one

Yellow solid (1.2 g, 38% yield); m.p. = 207–210 °C; ^{1}H NMR (700 MHz, DMSO-d_6) δ (ppm) = 11.98 (br. s., 1 H), 8.82 (d, J = 1.9 Hz, 1 H), 8.42 (dd, J = 8.9, 2.00 Hz, 1 H), 7.55 (s, 1 H), 7.26 (d, J = 8.9 Hz, 1 H), 3.22 (s, 3 H) (see Appendix S41); ^{13}C NMR (176 MHz, DMSO-d_6) δ (ppm) = 168.3, 154.7, 150.4 (q, J = 38.5 Hz), 138.7, 137.3, 135.3, 131.8, 130.1, 124.3, 119.7, 117.5 (q, J = 273.4 Hz), 27.1 (see Appendix S42); HRMS (ESI) m/z: 314.0394 was found (calculated for $C_{12}H_7F_3N_3O_4^-$, $[M–H]^-$ 314.0394).

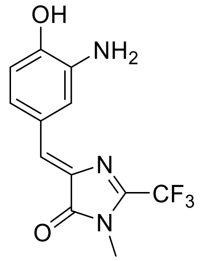

(*Z*)-5-(4-hydroxy-3-aminobenzylidene)-3-methyl-2-(trifluoromethyl)-3,5-dihydro-4*H*-imidazol-4-one (7b)

The aforementioned (*Z*)-5-(4-hydroxy-3-nitrobenzylidene)-3-methyl-2-(trifluoromethyl)-3,5-dihydro-4*H*-imidazol-4-one (0.63 g, 2 mmol) and tin (II) chloride dihydrate (3 g, 13.2 mmol) were suspended in 20 mL of THF. The mixture was refluxed for 3 h. The solvent was then evaporated and the mixture was dissolved by potassium phosphate buffer (1 M, 50 mL), extracted with $CHCl_3$ (5 × 75 mL) and dried over Na_2SO_4. The solvent was evaporated and crude product was purified by column chromatography (using the eluent $CHCl_3$-EtOH at v/v of 95/5).

Orange solid (88 mg, 15% yield); m.p. = 194–197 °C; ^{1}H NMR (700 MHz, DMSO-d_6) δ (ppm) = 7.61 (d, J = 1.9 Hz, 1 H), 7.31 (dd, J = 8.1, 2.0 Hz, 1 H), 7.25 (s, 1 H), 6.77 (d, J = 8.2 Hz, 1 H) 3.21 (s, 3 H), while the signals of –OH and $–NH_2$ groups were significantly broadened and undefined in the ^{1}H spectrum (see Appendix S43); ^{13}C NMR (176 MHz, DMSO-d_6) δ (ppm) = 168.4, 149.5, 147.6 (q, J = 38.5 Hz), 137.3, 135.9, 132.5, 125.6, 124.8, 117.8 (q, J = 272.7 Hz),

117.2, 114.4, 26.9 (see Appendix S44); HRMS (ESI) m/z: 286.0798 was found (calculated for $C_{12}H_{11}F_3N_3O_2^+$, $[M+H]^+$ 286.0798).

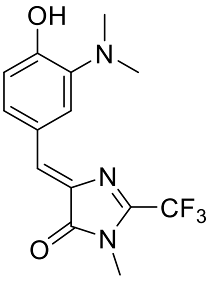

(*Z*)-5-(3-(dimethylamino)-4-hydroxybenzylidene)-3-methyl-2-(trifluoromethyl)-3,5-dihydro-4*H*-imidazol-4-one (8b)

Orange solid (0.5 g, 16% yield); m.p. – over 250°C with decomposition; ^{1}H NMR (700 MHz, DMSO-d_6) δ (ppm) = 10.34 (br. s., 1 H), 7.97 (d, J = 1.3 Hz, 1 H), 7.71 (dd, J = 8.3, 1.6 Hz, 1 H), 7.39 (s, 1 H), 6.88 (d, J = 8.4 Hz, 1 H), 3.22 (s, 3 H), 2.71 (s, 6 H) (see Appendix S45); ^{13}C NMR (201 MHz, DMSO-d_6) δ (ppm) = 168.4, 154.7, 148.0 (q, J = 38.3 Hz), 141.4, 135.3, 132.9, 129.6, 124.7, 122.9, 117.7 (q, J = 272.8 Hz), 116.1, 42.3, 26.9 (see Appendix S46); HRMS (ESI) m/z: 314.1103 was found (calculated for $C_{14}H_{15}F_3N_3O_2^+$, $[M+H]^+$ 314.1116).

S1.3 Synthesis of the locked chromophore series.

On the experimental design and execution front, we need to mitigate limitations of not having the identical substitutions on the unlocked nonfluorescent and locked fluorescent compounds for an ideal one-to-one analytical comparison. In fact, many of the locked GFP chromophore derivatives (R_P: –OMe, –OH; R_I: –CF_3, –$COCH_3$) were not synthesizable with the borylation method we proposed and used for conformational locking of the chromophores. This is because: first, the reagent boron tribromide forms insoluble complexes with *ortho*-aminophenols and *ortho*-hydroxyphenols (catechols), which does not allow borylation of the corresponding derivatives; second, this reagent easily removes the methyl group from anisoles with the formation of phenols, which does not allow borylation of derivatives with methoxy groups without their destruction.

In addition, the introduction of difluoroboryl group exerts an electron-acceptor effect on the imidazolone fragment due to the formation of a boron-nitrogen coordination bond. Therefore, the combination of this effect and the electron-acceptor effect of the trifluoromethyl (–CF_3) or ketone (–$COCH_3$) group makes such compounds unstable to the action of any nucleophiles. This intrinsic

issue makes it impossible to isolate the corresponding substances, since aqueous treatment leads to hydrolytic opening of the imidazolone cycle. In this regard, we were unable to obtain borylated derivatives of either trifluoromethylated or ketone derivatives. Nevertheless, this experimental challenge has been mitigated in the following ways. First, we carefully designed and strategically used all the available GFP chromophore derivatives that can be synthesized anew, plus some previously synthesized and characterized precursors,[7,9] to systematically derive important physical chemistry principles and mechanistic insights. Second, we newly synthesized three more BF_2-locked compounds/photoacids with R_P = –Cl, –Br, $–NMe_2$ and R_I = $–CH_3$ (termed as L1Cl, L1Br, $L1NMe_2$, respectively) with the synthesis procedures and NMR spectra presented below. Together with L0F (R_P = –H, R_I = $–CH_3$) and L1F (R_P = –F, R_I = $–CH_3$), we obtained five locked counterparts of the unlocked $–CH_3$ series of photoacids, which allows us to perform the same multivariable linear regression and kinetic analysis (see Figures S19d and S21 as well as Table S8 below) as the latter compounds for further mechanistic insights.

The synthesis routes and characterization of $\mathbf{L}\boldsymbol{n}\mathbf{F}$ compounds (n = 1, 2, 3) have been reported in our previous publications.[2,6]

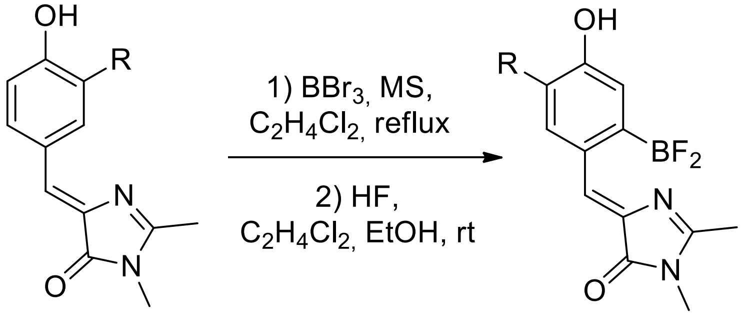

The corresponding arylidene-imidazolone (1 mmol) was dissolved in dry $C_2H_4Cl_2$ (20 mL). Molecular sieves 3 Å (5 g) and 4 Å (5 g) were added, followed by a solution of boron tribromide in dry $(CH_2Cl)_2$ (1 M, 6 mL, 6 mmol), and the reaction mixture was refluxed for 6 h under inert atmosphere. Then, the mixture was cooled and filtered; molecular sieves were washed with EtOH (20 mL). The solution was mixed with aqueous HF (20%, 6 mL) and stirred for 30 min. The mixture was diluted with EtOAc (300 mL), washed with water (3 × 100 mL), aqueous HCl (1%, 3 × 100 mL), brine (2 × 100 mL), dried over Na_2SO_4, and the solvent was evaporated under reduced pressure. The crude product was purified by column chromatography (CH_2Cl_2-iPrOH, 1:1), then washed with Et_2O (3 × 3 mL), and dried under reduced pressure.

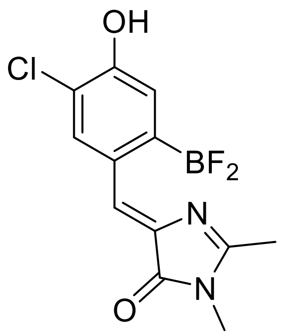

(*Z*)-5-(5-chloro-2-(difluoroboranyl)-4-hydroxybenzylidene)-2,3-dimethyl-3,5-dihydro-4*H*-imidazol-4-one

Dark green solid (69 mg, 23% yield); m.p. — over 250 °C with decomposition; ^{1}H NMR (800 MHz, 303 K, DMSO-d_6) δ (ppm) = 10.97 (s, 1 H), 7.72 (s, 1 H), 7.61 (s, 1 H), 7.21 (s, 1 H), 3.25 (s, 3 H), 2.75 (s, 3 H) (see Appendix S47); ^{13}C NMR (201 MHz, 303 K, DMSO-d_6) δ (ppm) = 166.0, 162.8, 156.5, 133.0, 127.6, 125.9, 125.2, 119.3, 119.3, 26.5, 13.0 (one signal of the C–BF_2 fragment is missing due to broadening) (see Appendix S48); HRMS (ESI) m/z: 279.0508 was found (calculated for $C_{12}H_{10}BClFN_2O_2^+$, $[M–F]^+$ 279.0503).

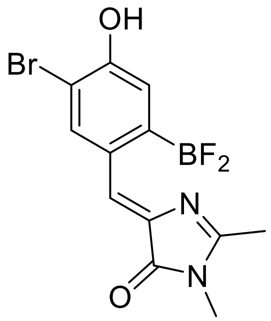

(*Z*)-5-(5-bromo-2-(difluoroboranyl)-4-hydroxybenzylidene)-2,3-dimethyl-3,5-dihydro-4*H*-imidazol-4-one

Dark green solid (106 mg, 31% yield); m.p. — over 250 °C with decomposition; ^{1}H NMR (800 MHz, 303 K, DMSO-d_6) δ (ppm) = 11.03 (br. s., 1 H), 7.86 (s, 1 H), 7.60 (s, 1 H) 7.18 (br. s., 1 H), 3.24 (s, 3 H), 2.74 (s, 3 H) (see Appendix S49); ^{13}C NMR (75 MHz, 303 K, DMSO-d_6) δ (ppm) = 166.0, 162.8, 157.5, 136.2, 127.5, 126.5, 125.1, 119.1, 108.8, 26.6, 13.0 (one signal of the C–BF_2 fragment is missing due to broadening) (see Appendix S50); HRMS (ESI) m/z: 323.0002 was found (calculated for $C_{12}H_{10}BBrFN_2O_2^+$, $[M–F]^+$ 322.9998).

S2. DFT calculations and color tuning of GFP chromophores

In our recent publications[6,10] we have used density function theory (DFT) calculations to demonstrate the color tuning mechanism in various series of conformationally locked GFP chromophore analogues. We termed it the "double-donor-one-acceptor" (DDA) strategy. The "double-donor" refers to the two electron-donating groups (EDGs) at the chromophore P-ring, i.e., original $–O^{-}$ (–OH becomes deprotonated) and the adjacent R_P site by EDG, and "one-acceptor" is the R_I site substitution by an electron-withdrawing group (EWG, see Figure 1c in main text for the atomic site designation). With time-dependent (TD-)DFT calculations, we demonstrated that the spectral redshift such as emission arises from a synergistic and additive lowering of the transition energy gap; that is, the "double-donor" structure causes an increase in the energy of the highest occupied molecular orbital (HOMO), a destabilization effect for the ground state, due to the presence of an EDG *ortho* to the $–O^{-}$ group. Meanwhile, the "one-acceptor" stabilizes the excited state and lowers energy for the lowest unoccupied molecular orbital (LUMO) due to excited-state intramolecular charge transfer (ICT).[6] The HOMO–LUMO transition is primarily responsible for the electronic absorption and emission transitions for the conformationally locked *p*-HBDI derivatives. Although the calculations were performed on locked GFP chromophores, the general mechanism on color tuning should remain the same for the unlocked GFP chromophores of the current work considering the small extent of structural modification.[11] This assertion is supported by systematic TD-DFT calculations at the same level for the unlocked GFP chromophores with representative substitutions as shown in Figures S1 and S2, as well as recent reports that demonstrate the color-tuning mechanism in GFP with an unlocked HBDI chromophore core.[12,13]

The chromophore energetics can be understood by the π-electron delocalization in HOMO and LUMO or simple particle-in-a-box quantum theory. Figures S1 (the protonated form) and S2 (the deprotonated form) present the electron density distributions (EDDs) for *p*-HBDI and its derivative compounds **8a** (R_P = $–NMe_2$) and **1c** (R_I = $–COCH_3$) to showcase the effects of electron-donating and -withdrawing groups (EDG and EWG) at R_P and R_I sites, respectively. First, EDG at R_P induces a considerable EDD change of HOMO that exhibits more localized electron density at the phenolic/phenolate and $–NMe_2$ group, hence drastically raising the HOMO energy. In comparison, the EDD of LUMO remains similar to the unsubstituted compound (*p*-HBDI, R_P = –H) that are delocalized across both rings, resulting in minimal changes in the energy due to their similar

quantum box sizes. Therefore, EDG of R_P shrinks the electronic transition gap mainly by raising energy of the lower-lying state or MO, i.e., ground state or HOMO (Figure S1, left panel). Second, EWG at R_I red-shifts the electronic transition in a very different manner, a more common mechanism as reported in literature.[14,15] As seen in *p*-HBDI, the photoexcitation promotes HOMO → LUMO transition and causes ICT from the phenolic/phenolate to the imidazolinone ring moiety. The ICT process is considered a stabilization effect for the excited state and *p*-HBDI does not exhibit pronounced ICT. The incorporation of an EWG at R_I enhances ICT and leads to a more delocalized EDD. This enlarged quantum box thereby gives rise to a significant decrease in LUMO energy. Meanwhile, EWG at R_I also lowers HOMO energy as it creates an extended π-conjugation in the ground state and hence a larger box than the $R_I = CH_3$ case (as in *p*-HBDI). Nevertheless, the extent of energy lowering for HOMO is still less than that for LUMO, which results in an overall redshift in the transition energy gap (Figure S1, right panel). From Figures S1 and S2, it is conspicuous that the donor (EDG at R_P) or acceptor (EWG at R_I) modifications exert different impacts on the energetics of the acid (protonated) and conjugated base (deprotonated). This fundamental insight supports our proposition that the photoacidity tuning is accompanied by emission color change, which can be realized through strategic modifications at R_P and/or R_I for the *p*-HBDI-based family of photoacids. Moreover, such versatile photophysical property tuning can be achieved through the modifications at donor and/or acceptor moieties for other photoacids.

We also note the applicability of Förster equation (also based on assumptions/approximations) is not the central point of our work. Our work revealed how the physical principles behind classical mechanisms (by Förster equation) and beyond (non-fluorescent photoacids) underlie the observed photoacidity of the "normal" (fluorescent photoacids in the literature and this work) and "anomalies" (*p*-HBDI derived nonfluorescent photoacids of this work). The deep insights into these overlooked/glossed over "anomalies", the widely existing yet severely underexplored dim photoacids, is a major contribution of our work with both advanced experimental and theoretical methods for characterization and generalization, which has not been addressed in the field.

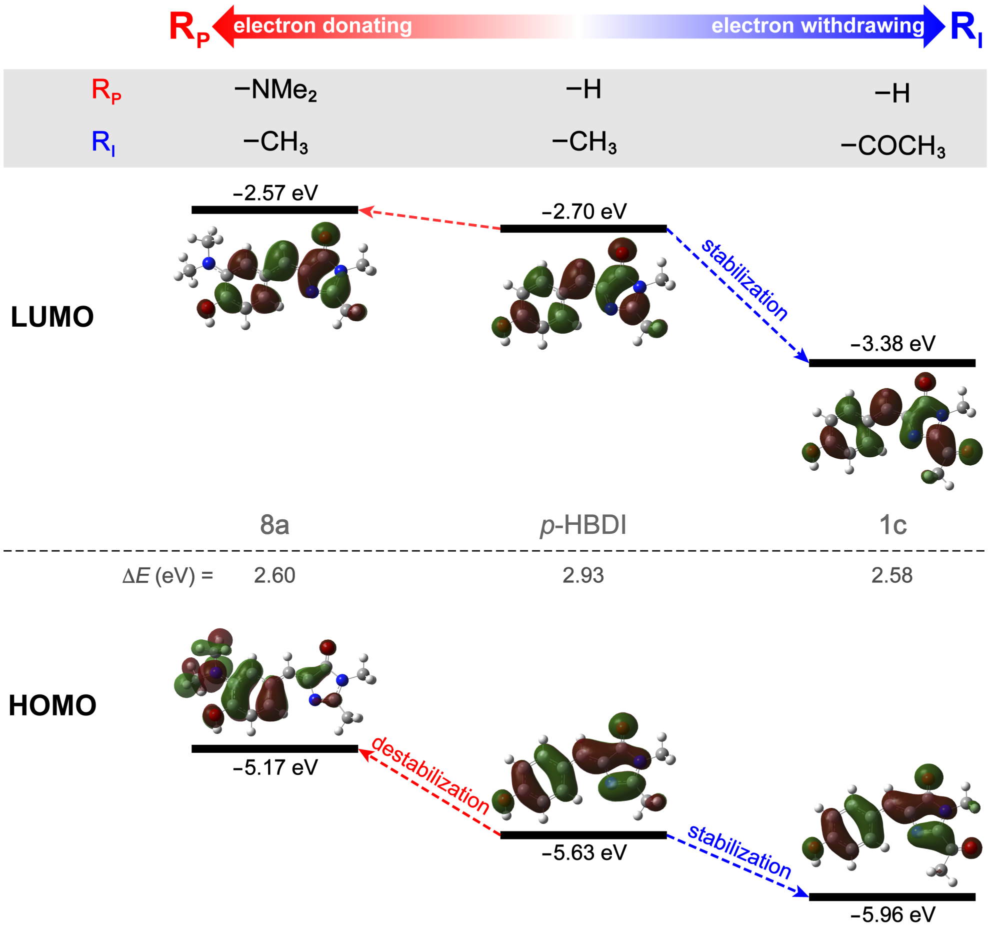

Figure S1. Calculated energetics for photoacids in the protonated form. Selected substituents are shown to illustrate the electronic effects of EDG and EWG at R_P and R_I sites, respectively. The calculations were performed at the TD-DFT/B3LYP/6-311G+(d,p) functional level/basis set. The solvent effect was accounted for by the implicit integral equation formalism variant polarizable continuum model (IEFPCM) while acetonitrile was used as the solvent. The EDG- and EWG-induced effects are highlighted by the red and blue dashed arrows, respectively, with major energy-shifting trends listed by the arrows. The GFP chromophore derivative naming scheme and the corresponding $S_0 \rightarrow S_1$ transition energy gaps are denoted above and below the central gray dashed line, respectively.

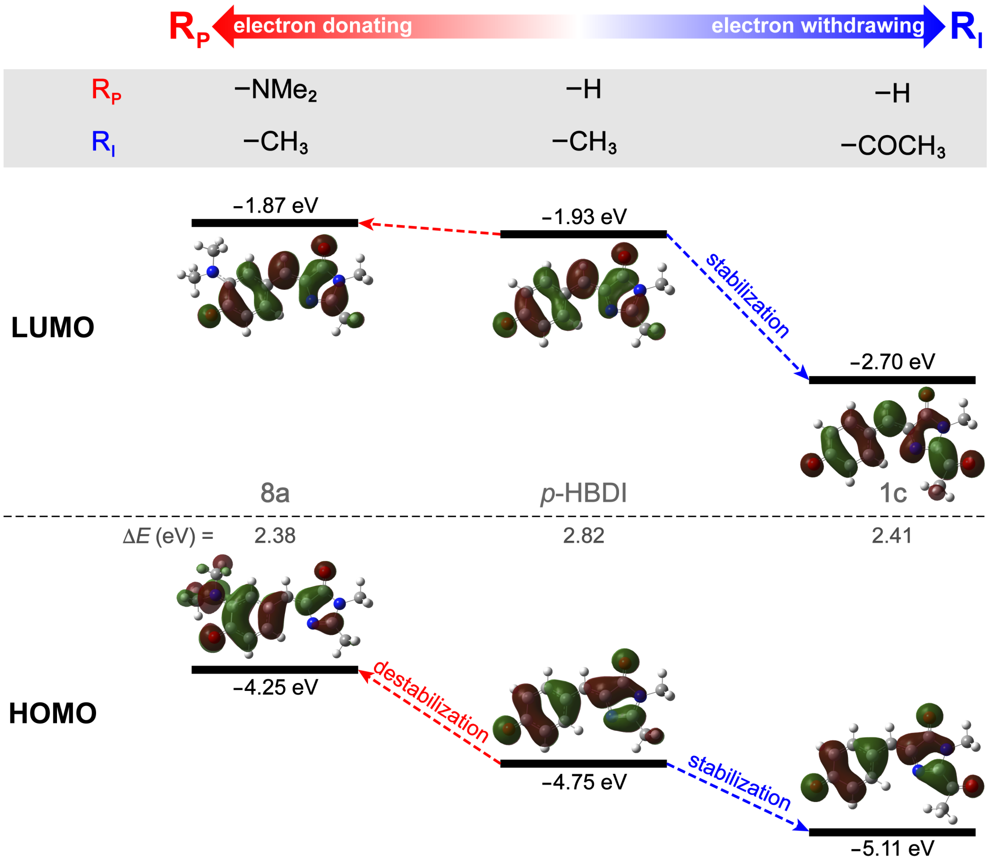

Figure S2. Calculated energetics for photoacids in the deprotonated form. Selected substituents are shown to illustrate the electronic effects of EDG and EWG at R_P and R_I sites, respectively. The calculations were performed at the TD-DFT/B3LYP/6-311G+(d,p) functional level/basis set. The solvent effect was accounted for by the implicit integral equation formalism variant polarizable continuum model (IEFPCM) while acetonitrile was used as the solvent. The EDG- and EWG-induced effects are highlighted by the red and blue dashed arrows, respectively, with major energy-shifting trends listed by the arrows. The GFP chromophore derivative naming scheme and the corresponding $S_0 \rightarrow S_1$ transition energy gaps are denoted above and below the central gray dashed line, respectively.

S3. FSRS reveals mechanistic insights into photoacid energetics

S3.1 Optical setup

The FSRS spectra were collected using a home-built setup based on a mode-locked Ti:sapphire oscillator and a laser regenerative amplifier (Coherent, Inc.) that provides an 800 nm fundamental pulse with 35 fs duration and 1 kHz repetition rate. The detailed description of the setup can be found in our previous publications.[16-18] In brief, it requires three incident pulses, actinic pump (A_{pu}), Raman pump (R_{pu}), and Raman probe (R_{pr}) to generate the excited-state FSRS signal, whereas only two pulses (R_{pu} and R_{pr}) are needed to generate the ground-state FSRS signal. The visible A_{pu} in our FSRS setup is generated through a two-stage femtosecond noncollinear optical parametric amplifier (NOPA) and then temporally compressed by a chirped mirror pair (DCM-12, 400−700 nm, Laser Quantum, Inc.). The picosecond R_{pu} is generated by a two-stage ps NOPA system. The femtosecond R_{pr} is supercontinuum white light (SCWL) that is generated by focusing a small portion of the 800 nm fundamental pulse onto a 2-mm-pathlength quartz cuvette filled with deionized water and temporally compressed by a chirped mirror pair (DCM-9, 450−950 nm or DCM-12, 400−700 nm, Laser Quantum, Inc., depending on the sample). The sample was housed in a 1-mm-pathlength quartz cuvette and stirred with a short zinc-coated steel staple that is spun by a motorized magnet. The experimental conditions for the ground- and excited-state FSRS measurements are summarized in Table S1. The FSRS spectra were shown in Figures S4–S7.

S3.2 Resonance enhancement and wavelength selections

To resolve the vibrational signatures with a high signal-to-noise ratio, the R_{pu} wavelength can be strategically chosen by taking advantage of resonance enhancement effect in FSRS.[19-21] For ground-state FSRS measurements on the Stokes side, R_{pu} was tuned to a pre-resonance wavelength at the red edge of the absorption peak where the sample absorbs minimally. This way it avoids on-resonance effects that may complicate the Raman mode frequency, intensity, and line shapes.[21,22] Likewise, for ground-state FSRS measurements on the anti-Stokes side, the R_{pu} wavelength is selected to ensure the shortest R_{pr} photon wavelength that yields the highest-frequency mode in the probing window lies at the red edge of the absorption band (i.e., minimal absorption of R_{pr}) with pre-resonance enhancement.[23-25] Since there are no bleaching or dumping issues (commonly seen in excited-state FSRS)[26] in ground-state FSRS, high R_{pu} power and sample concentration are used to maximize the signal-to-noise ratio in the collected FSRS spectra all at room temperature.

For excited-state FSRS measurements, R_{pu} is examined and chosen at the red side of the stimulated emission (SE) band. This resonance condition for either Stokes or anti-Stokes FSRS has been reported to produce good excited-state FSRS signals in the literature.[27-29] We selected the R_{pu} wavelength accordingly for compounds **1a**, **5a**, **8a**, **1b**, and **1c** at which the differential absorbance (ΔOD, measured by transient absorption spectroscopy) is less than half of the maximal SE band intensity. Combined with a moderate R_{pu} power of 2–2.5 milliwatt (mW), the bleaching and dumping issues are expected to be mitigated while the excited-state FSRS intensity of active modes can be resonantly enhanced, which is verified by the resultant spectra of these deprotonated compounds in acetonitrile (Figure S7). Anti-Stokes FSRS (with a bluer Raman probe than R_{pu}) was performed for compound **8a** (Table S1) due to its large Stokes shift and red SE (Figure S9) that require an even redder R_{pu} to achieve resonance and avoid excited-state population dumping.

S3.3 Correlation between vibrational mode frequency and color/energetics

Using the frequency of vibrational modes particularly certain stretch modes to understand electron density distribution or migration is a very useful method in various aspects of chemistry. It provides direct evidence for the electron density difference among a family of structural analogues or for the same molecule interacting with different environments and thus can be used to infer color and energetics, or other important properties. For example, the spectral tuning of the retinal chromophore in visual pigments is associated with strong dipolar interactions between the chromophore and local environment. With the same retinal chromophore, the different electrostatic environment of the binding pocket of host protein could induce hundreds of nanometer variation in absorption wavelength. Besides theoretical calculations, Raman spectroscopy offers direct evidence for the basis of spectral tuning. The retinal chromophore has a polyene structure and usually exhibits strong Raman intensity for the C=C stretch mode of alternating double bonds. This mode demonstrates frequency lowering with absorption redshift, indicating the reduction of bond alternation and increased π electron delocalization.[30,31] Similar phenomena have also been observed in other polyenes.[32,33] These results for the electronic ground state at equilibrium have been enabled by the conventional resonance Raman and FSRS.

To understand the structure-color relationship for the GFP chromophore, we in this work identified the strong mode at 1552 cm^{-1} mode in acetonitrile for the deprotonated (anionic) parent photoacid, *p*-HBDI, to be an electron density indicator mode. DFT calculations assign this mode

to stretch of alternating C=O and C=C bonds corresponding to the quinoid resonance structure (see Figure 2a in main text and Figure S3, as well as the overlaid experimental and calculated Raman spectra in Figures S4–S6 below for several series of GFP chromophore derivatives). Therefore, it can be used to indicate the structural shift between the benzenoid and quinoid resonance structures and hence the electron delocalization and energetics for the deprotonated GFP chromophore and its substituted derivatives provided that this normal mode is conserved upon substitutions, which are validated by DFT calculations (Figure S3). In the ground state, EWG substitution at R_I leads to a dramatic blueshift of this mode (see Figure 2b in main text and Figures S4–S6). This is an indication of the shift from the benzenoid to quinoid resonance structure which reflects a more delocalized ground state in accord with the computed energy lowering (Section S2, Figures S1–S2). The frequency blueshift is also largely in line with the EWG strength of R_I as a result of the chemically modified electron distribution (Figures S7–S8). In contrast, this mode barely shifts by R_P substitution regardless of EWG or EDG suggesting its marginal impact on the electron distribution of *p*-HBDI molecular backbone. The result complements the quantum calculations and indicates the different origin of redshift by an EDG at R_P from that by an EWG at R_I. In the excited state, the consistent blueshift of this mode with respect to the ground state is reflective of the photoinduced intramolecular charge transfer (ICT). The R_I substitution with an EWG enhances ICT, thus lowering the excited-state energy and blue-shifting the mode frequency (see Figure 2b in main text).

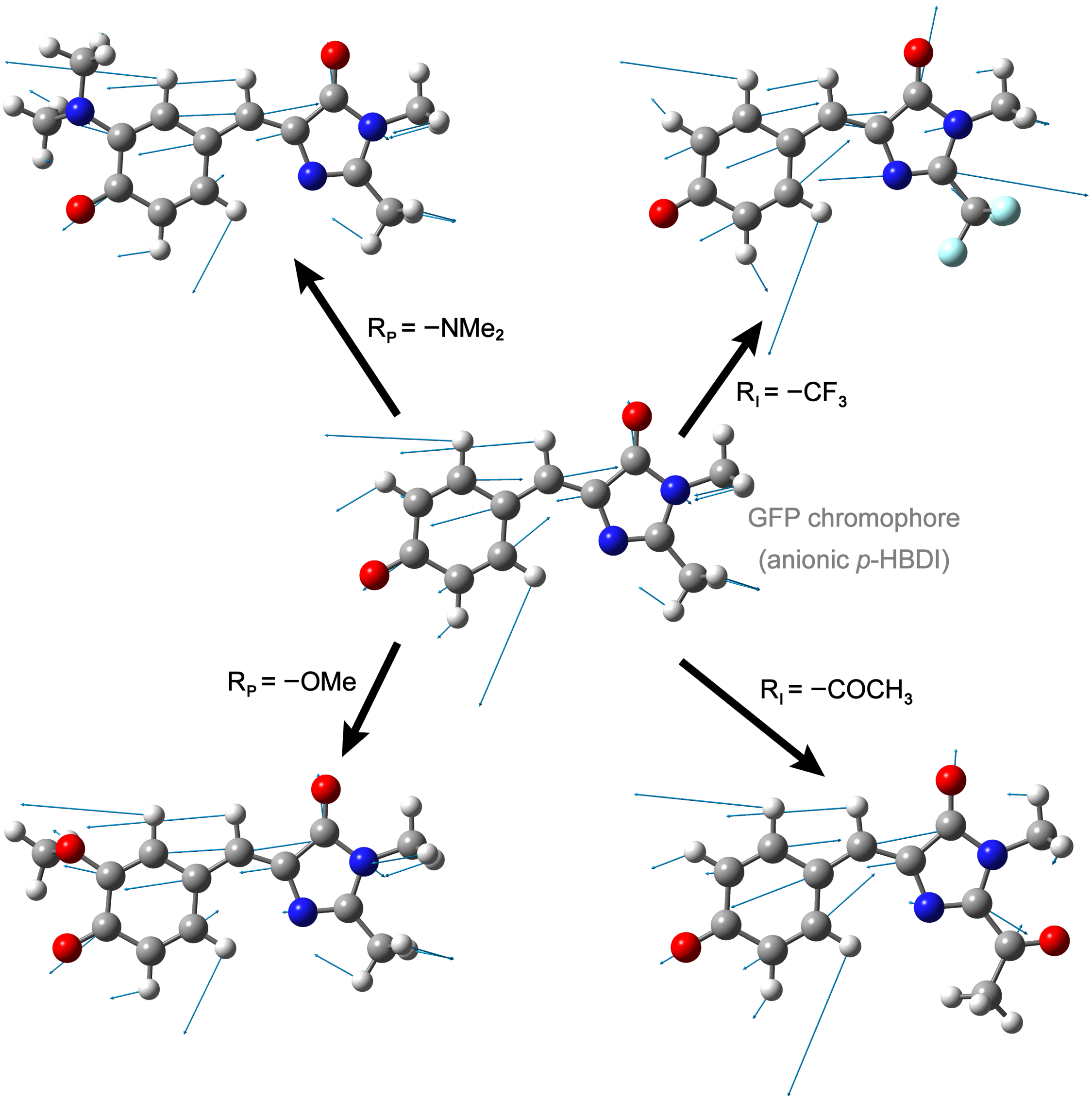

Figure S3. Calculated vibrational motions for the alternating C=O/C=C stretch mode. The ground-state Raman calculations were performed at the DFT/B3LYP/6-311G+(d,p) functional level/basis set for the deprotonated chromophore (the *p*-HBDI anionic form is shown in the middle). The solvent effect was treated by the implicit integral equation formalism variant polarizable continuum model (IEFPCM) while acetonitrile was used as the solvent. Two R_P and two R_I substituents are depicted to be consistent with ground- and excited-state FSRS measurements (see Figure 2b in main text). The atomic displacements for the pertinent vibrational normal mode of each chromophore are depicted by the cyan thin arrows.

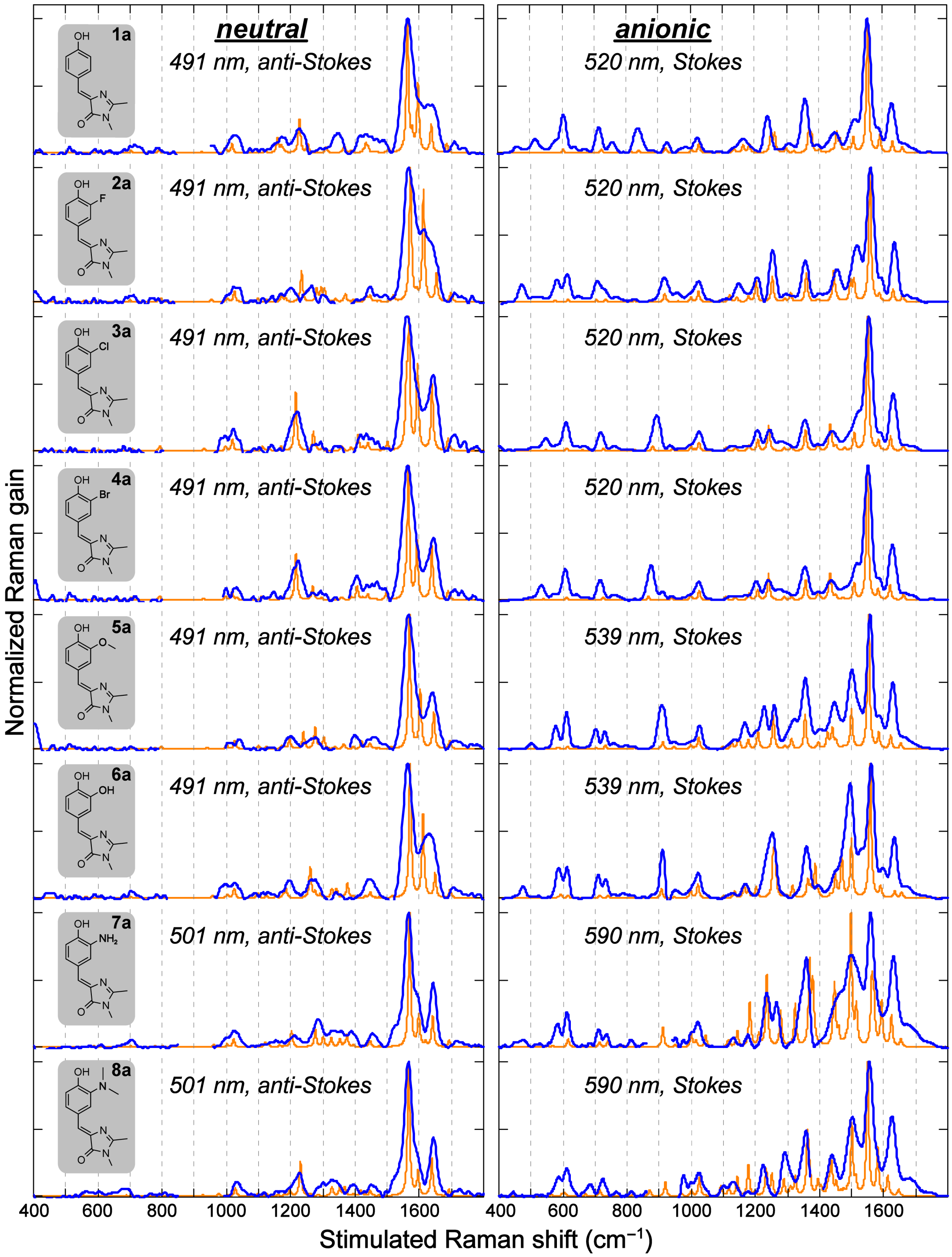

Figure S4. Ground-state FSRS and calculated spectra for $–CH_3$ series. The experimental spectra (blue) are overlaid with the calculated Raman spectra (orange; scaling factor is 0.985–0.99). The FSRS experimental conditions are also indicated. Chemical structures of the series of substituted *p*-HBDI chromophore derivatives (**1a**-**8a**) are listed in the left panel insets. The 950–1050 cm^{-1} regions for some spectra are blank due to the incomplete removal of the solvent (acetonitrile) peak.

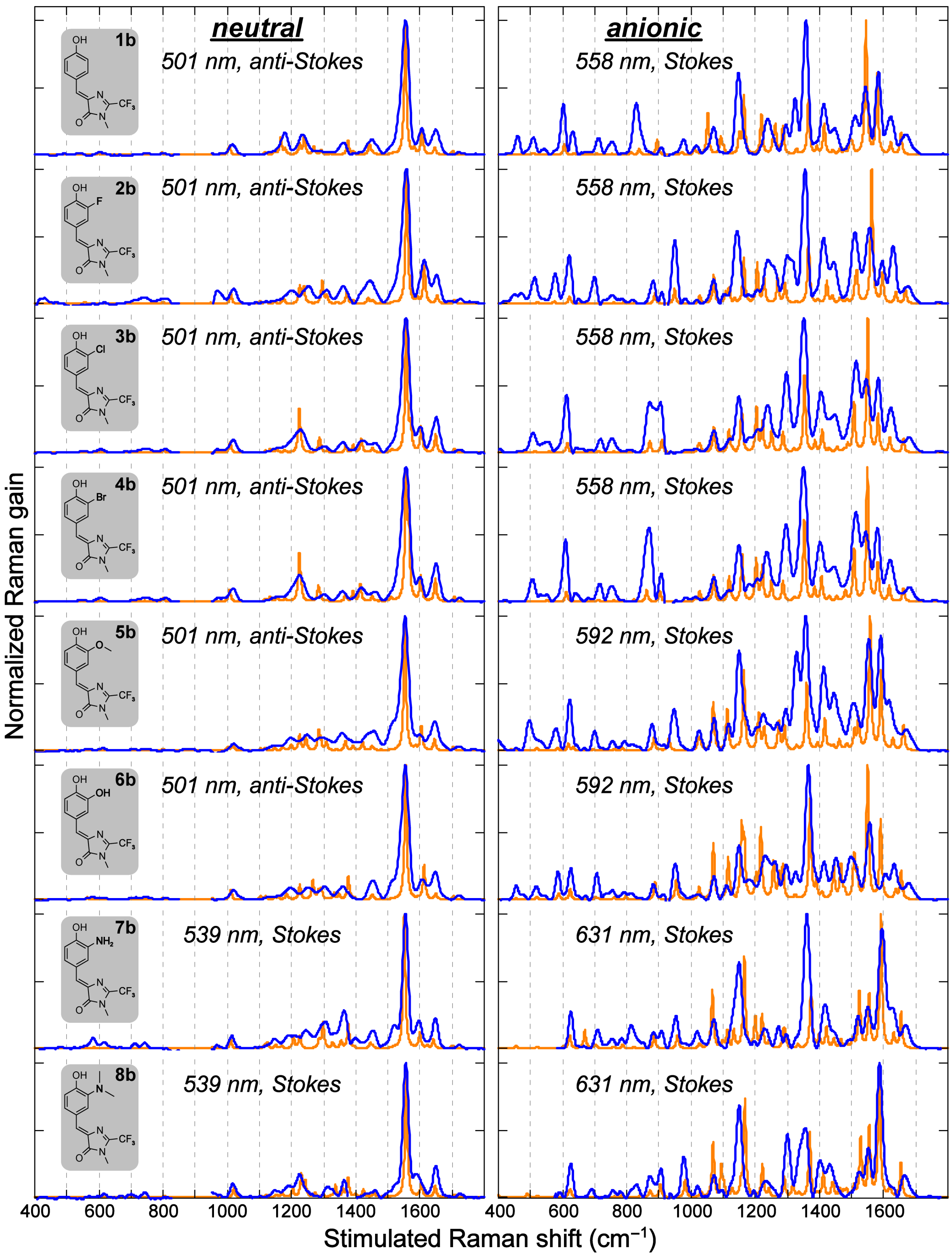

Figure S5. Ground-state FSRS and calculated spectra for $-CF_3$ series. The experimental spectra (blue) are overlaid with the calculated Raman spectra (orange; scaling factor is 0.985–0.99). The FSRS experimental conditions are also indicated. Chemical structures of the series of substituted

p-HBDI chromophore derivatives (**1b-8b**) are listed in the left panel insets. The 950–1050 cm^{-1} regions for some spectra are blank due to the incomplete removal of the solvent (acetonitrile) peak. The absence of low-frequency spectra (<600 cm^{-1}) with 631 nm R_{pu} is due to the narrower probe window in cm^{-1} (an energy unit) in the red-wavelength region (hence on the Stokes side) with the same grating used for all the samples.

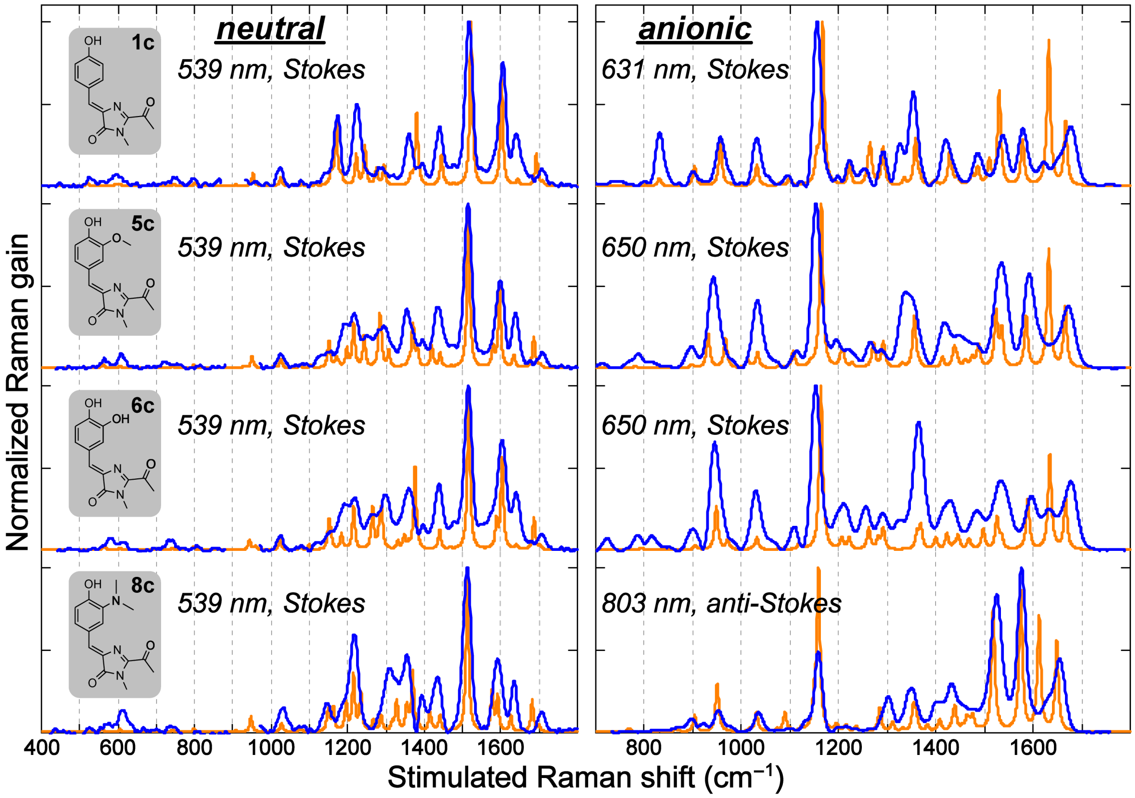

Figure S6. Ground-state FSRS and calculated spectra for –$COCH_3$ series. The experimental spectra (blue) are overlaid with the calculated Raman spectra (orange; scaling factor is 0.985–0.99). The FSRS experimental conditions are also indicated. The 950–1050 cm^{-1} regions for some spectra are blank due to the incomplete removal of the solvent (acetonitrile) peak. The low-frequency spectra (<700 cm^{-1}) for the deprotonated photoacids (anionic form) are not shown due to the narrower probe window in cm^{-1} (an energy unit) in the red-wavelength region with the same grating used for all the samples.

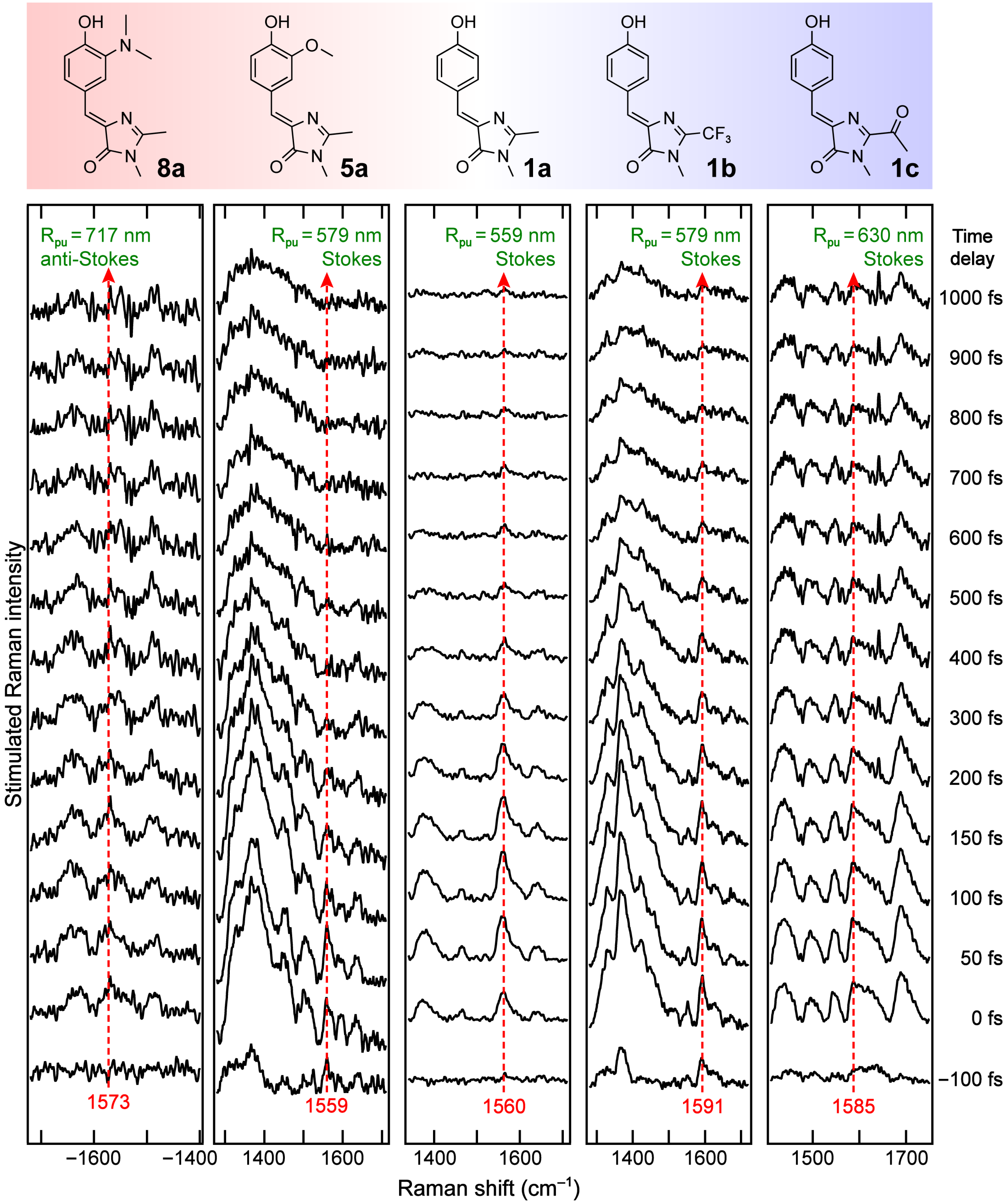

Figure S7. Excited-state FSRS spectra for photoacids with various R_P and R_I substitutions in acetonitrile. The selected photoacids with EDG- (red) and EWG-substitutions (blue shade) at R_P and R_I sites, respectively, are shown at top. The excited-state spectra at representative time delays after photoexcitation (shown on right side) are displayed to inform the Raman peak evolution. The wavenumber and upward arrow in red denotes the center frequency location and dynamics for the alternating double-bond stretching mode, respectively, in each panel. The detailed experimental conditions for measuring contrasting samples **1a**, **5a**, **8a**, **1b**, and **1c** can be found in Table S1.

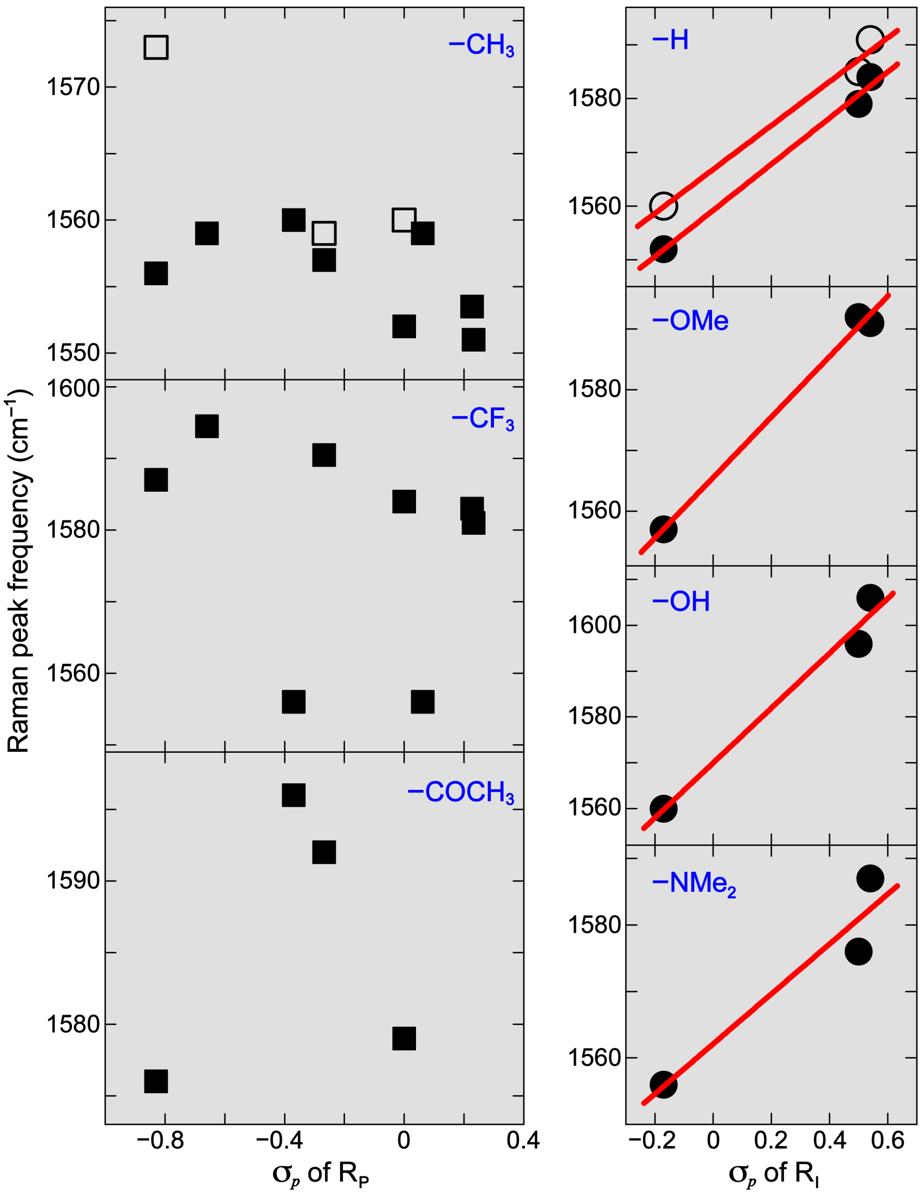

Figure S8. Correlation between Raman peak frequency and R_P/R_I substituents. The ground- (closed shape) and excited-state (open shape) frequencies for the most intense mode due to stretch of alternating double bonds (see Figure S3) are plotted against Hammett parameter σ_p of the R_P (square, left panels) and R_I (circle, right panels) substituents of the deprotonated *p*-HBDI derivatives. The various substituents (in blue color) are listed in the inset of each panel. The linear relationship for a positive correlation is highlighted by red straight lines in right panels.

Table S1. Experimental conditions for FSRS measurements.

Compound	Ground (0) or excited state (1)	Protonation state (A or B)[a]	R_P	R_I	Raman pump[b] wavelength (nm)	Stokes (S) or anti-Stokes (AS)
**1a**	0	A	–H	$-CH_3$	491	AS
**2a**	0	A	–F		491	AS
**3a**	0	A	–Cl		491	AS
**4a**	0	A	–Br		491	AS
**5a**	0	A	–OMe		491	AS
**6a**	0	A	–OH		491	AS
**7a**	0	A	$-NH_2$		501	AS
**8a**	0	A	$-NMe_2$		501	AS
**1b**	0	A	–H	$-CF_3$	501	AS
**2b**	0	A	–F		501	AS
**3b**	0	A	–Cl		501	AS
**4b**	0	A	–Br		501	AS
**5b**	0	A	–OMe		501	AS
**6b**	0	A	–OH		501	AS
**7b**	0	A	$-NH_2$		539	S
**8b**	0	A	$-NMe_2$		539	S
**1c**	0	A	–H	$-COCH_3$	539	S
**5c**	0	A	–OMe		539	S
**6c**	0	A	–OH		539	S
**8c**	0	A	$-NMe_2$		539	S
**1a**	0	B	–H	$-CH_3$	520	S
**2a**	0	B	–F		520	S
**3a**	0	B	–Cl		520	S
**4a**	0	B	–Br		520	S
**5a**	0	B	–OMe		539	S

**6a**	0	B	–OH		539	S
**7a**	0	B	$–NH_2$		590	S
**8a**	0	B	$–NMe_2$		590	S
**1b**	0	B	–H	$–CF_3$	558	S
**2b**	0	B	–F		558	S
**3b**	0	B	–Cl		558	S
**4b**	0	B	–Br		558	S
**5b**	0	B	–OMe		592	S
**6b**	0	B	–OH		592	S
**7b**	0	B	$–NH_2$		631	S
**8b**	0	B	$–NMe_2$		631	S
**1c**	0	B	–H	$–COCH_3$	631	S
**5c**	0	B	–OMe		650	S
**6c**	0	B	–OH		650	S
**8c**	0	B	$–NMe_2$		803	AS
**1a**	1[c]	B	–H	$–CH_3$	559	S
**5a**	1[c]	B	–OMe	$–CH_3$	579	S
**8a**	1[c]	B	$–NMe_2$	$–CH_3$	717	AS
**1b**	1[c]	B	–H	$–CF_3$	579	S
**1c**	1[c]	B	–H	$–COCH_3$	630	S

[a] The neutral form (A, protonated) is obtained by directly dissolving the compound in acetonitrile. For low-pK_a photoacids, ~0.01% (v:v) pH 3–5 water or 0.05%–0.2% (v:v) acetic acid were added to suppress the deprotonation. The anionic form (B, deprotonated) is obtained by adding 0.05%–0.1% (v:v) organic base, 1,8-Diazabicyclo[5.4.0]undec-7-ene (DBU), into acetonitrile. All sample solutions for ground-state FSRS measurements were prepared at high concentrations of the solute (optical density or OD>3 per mm at the solute absorption peak maxima) to increase the signal-to-noise ratio. The sample solutions for excited-state FSRS measurements were made with OD in the range of 1.5–2.5 per mm at the absorption peak maxima.

[b] The power used for R_{pu} in all the ground-state FSRS measurements was in the range of 3–4 mW.

[c] The A_{pu} wavelengths in the excited-state FSRS measurements for compounds **1a**, **5a**, **8a**, **1b**, **1c** are 487, 487, 486, 486, 542 nm, respectively; the power used was 0.2–0.25 mW. The R_{pu} power was 2–2.5 mW.

S4. Electronic absorption, emission, and 0–0 transition gap

S4.1 Instruments and measurement of electronic absorption and emission

The steady-state electronic absorption spectra were recorded with a Thermo Scientific Evolution 201 UV/Visible spectrophotometer. The fluorescence spectra were recorded with a Shimadzu RF-6000 spectrofluorophotometer. The solution sample was filled in a four-sided, 5-mm-pathlength quartz cuvette. Solvent background was pre-measured and subtracted during the collection of absorption spectrum. In fluorescence measurements, the sample concentration was kept below OD of 0.1 per 4–5 mm pathlength to minimize the inner-filter effect (re-absorption) that may red-shift the true spectrum. Due to very low fluorescence quantum yields (FQYs) of the substituted p-HBDI derivatives, the Raman scattering peak intensity from solvent might be comparable to the solute fluorescence intensity and drastically interfere with and change the solute fluorescence profile if not removed. The pure solvent spectrum under the same excitation conditions (i.e., wavelength, slit widths, etc.) is thus subtracted from the sample spectrum to retrieve the fluorescence spectrum of pure solute molecules. All the experiments were performed at room temperature (22 °C).

S4.2 Determination of the 0–0 transition gap

The purpose of obtaining the 0–0 transition gap is to determine the energy or energy difference of thermo-equilibrated states associated with the ground- and excited-state acid and conjugate base. Although in practice the 0–0 transition gap is often approximated by the energy of absorption or emission or their averaged value in the Förster equation, the presence of strong vibronic coupling in condensed phases such as solution often makes the approximations inaccurate or problematic. Instead of using hole-burning spectroscopy to rigorously measure the 0-0 transition gap,[34,35] we in this work adopted a more commonly used approach to make a close approximation based on the fundamental optical spectroscopy theory.[36] Considering a single harmonic vibrational mode and assuming the same frequency in the ground and excited electronic states (not a distorted oscillator), the vibronic absorption and emission intensity can be described by the following equations:

$$I_{Abs}(\tilde{v}) = [n(\tilde{v}) \cdot hc\tilde{v}] \cdot \sum_m \frac{S^m}{m!} e^{-S} \cdot \Gamma \cdot \delta[\tilde{v} + (\tilde{v}_{0-0} - m\tilde{v}_m)] \qquad \text{(S1)}$$

$$I_{Em}(\tilde{v}) = [n(\tilde{v}) \cdot hc\tilde{v}]^3 \cdot \sum_m \frac{S^m}{m!} e^{-S} \cdot \Gamma \cdot \delta[\tilde{v} - (\tilde{v}_{0-0} - m\tilde{v}_m)] \qquad \text{(S2)}$$

$n(\tilde{\nu})$ is the refractive index at the transition energy $hc\tilde{\nu}$ ($\tilde{\nu}$ is in cm^{-1} unit). S is the Huang-Rhys factor. Γ is the line shape function. $\tilde{\nu}_{0-0}$ is the 0–0 transition energy and $\tilde{\nu}_m$ is the vibrational energy of mth vibrational level. The 0–0 transition energy can thereby be determined from the crossing point of the normalized reduced electronic absorption and emission spectra by scaling the original spectra by factors of $1/hc\tilde{\nu}$ and $(1/hc\tilde{\nu})^3$, respectively (see Figures S9–S14 below).

This idealized model results in mirrored spectral profiles of absorption and emission where the allowed vibronic transitions of absorption and emission are symmetrically located on two sides of the 0–0 transition line/band (in the energy scale). Therefore, the mirror-image absorption and emission spectra are often required for a rather accurate determination of the 0–0 transition energy using this approach. For various series of p-HBDI-derived photoacids in this work, we note that the mirror-image law is largely obeyed by most substituents (–H, –F, –Cl, –Br, –OH, $-OCH_3$) despite that the vibronic coupling is not obvious for some molecules and the relative intensity of vibronic bands slightly differ. The substituents $-NH_2$ and $-NMe_2$, particularly the latter, lead to clearly asymmetric absorption and emission spectra for the deprotonated (anionic) chromophore, which seem to yield somewhat inaccurate 0–0 transition energy using the same approach. However, the fact that 0–0 transition energy determined this way not only yields good linear correlations of 0–0 transition energy, pK_a, ΔpK_a, and pK_a^* to the electronic, steric, and intermolecular interaction properties of substituent but also reasonably explains the kinetic ESPT behavior suggests that it is a close and reasonable approximation to the real 0–0 transition energy.

Moreover, we also examined the pK_a^* for substituents $-NH_2$ and $-NMe_2$ by approximating their 0–0 transition energies as the average energy of absorption and emission peaks. Although the resultant pK_a^* values qualitatively show consistency with the kinetics, the linear correlation to substituent properties becomes poor, implying the clear deviation of averaged absorption and emission energy from the 0–0 transition energy (see Section S5.2 and Table S4).

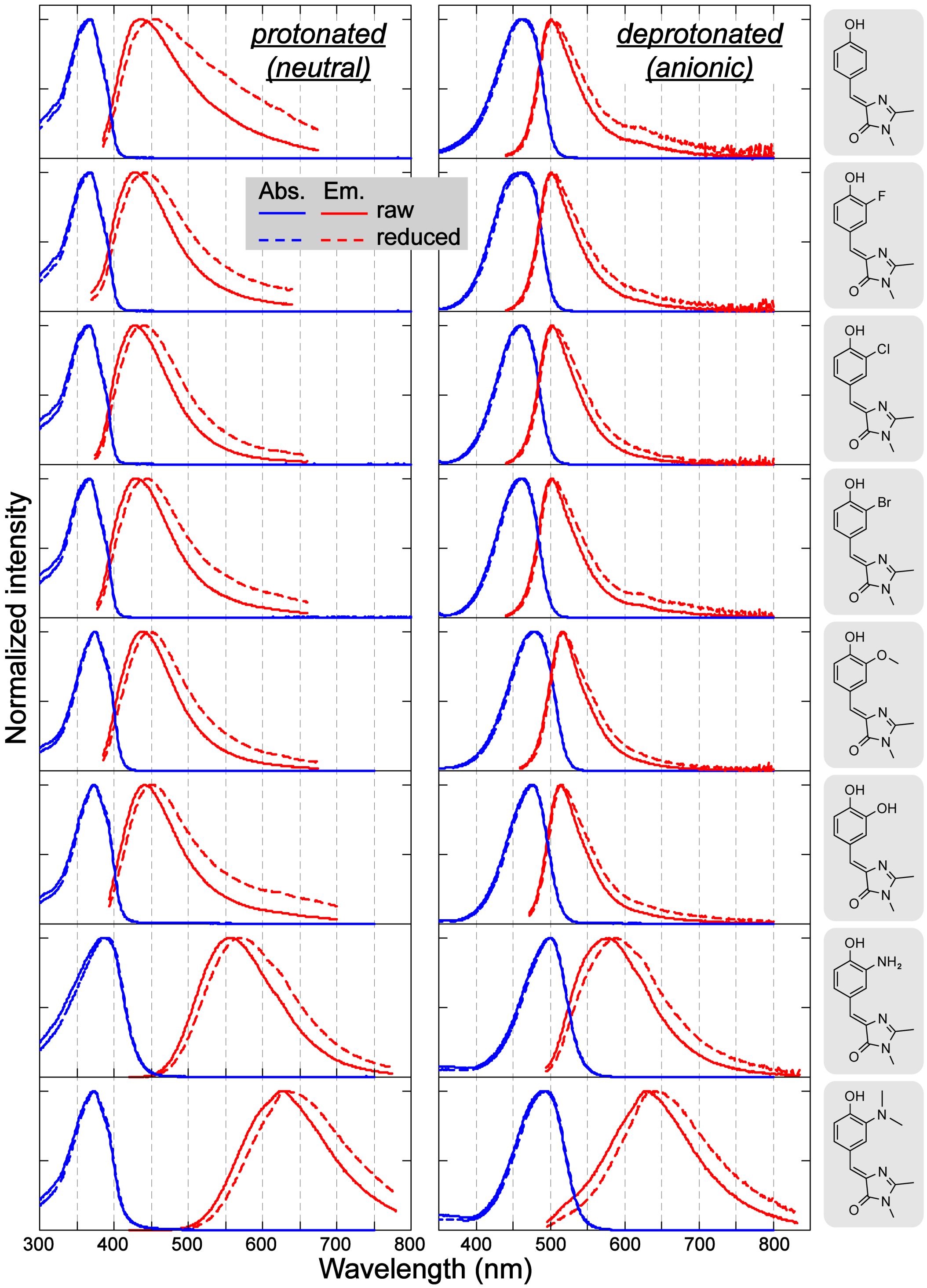

Figure S9. Absorption and emission spectra for the $-CH_3$ series in acetonitrile. The original absorption (blue) and emission (red) spectra are shown in solid lines. The corresponding reduced spectra (dashed lines) were used to determine the 0-0 transition energy gap for the protonated/neutral (left) and deprotonated/anionic (right) chromophores.

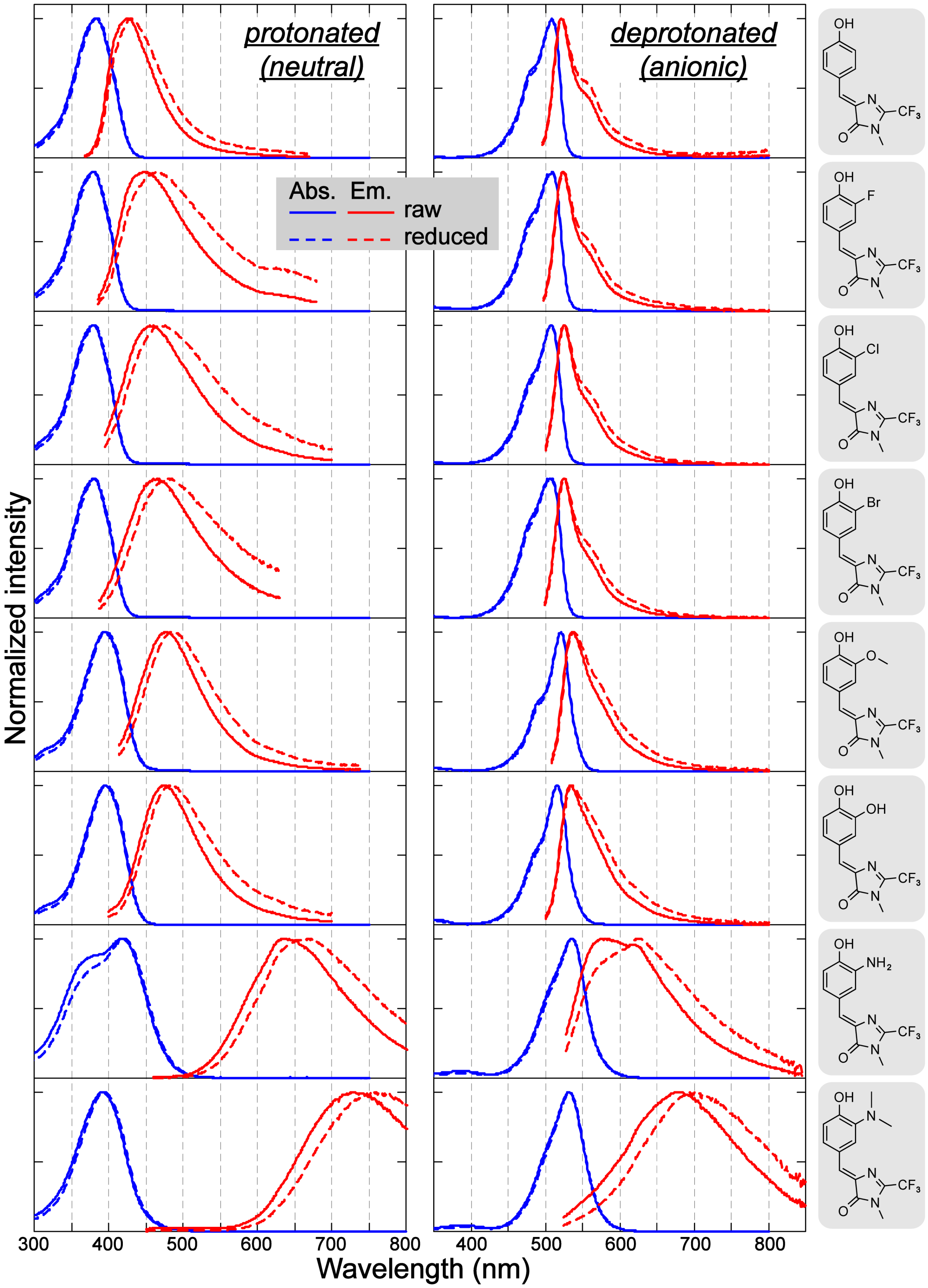

Figure S10. Absorption and emission spectra for the –CF_3 series in acetonitrile. The original absorption (blue) and emission (red) spectra are shown in solid lines. The corresponding reduced spectra (dashed lines) were used to determine the 0-0 transition energy gap for the protonated/neutral (left) and deprotonated/anionic (right) chromophores.

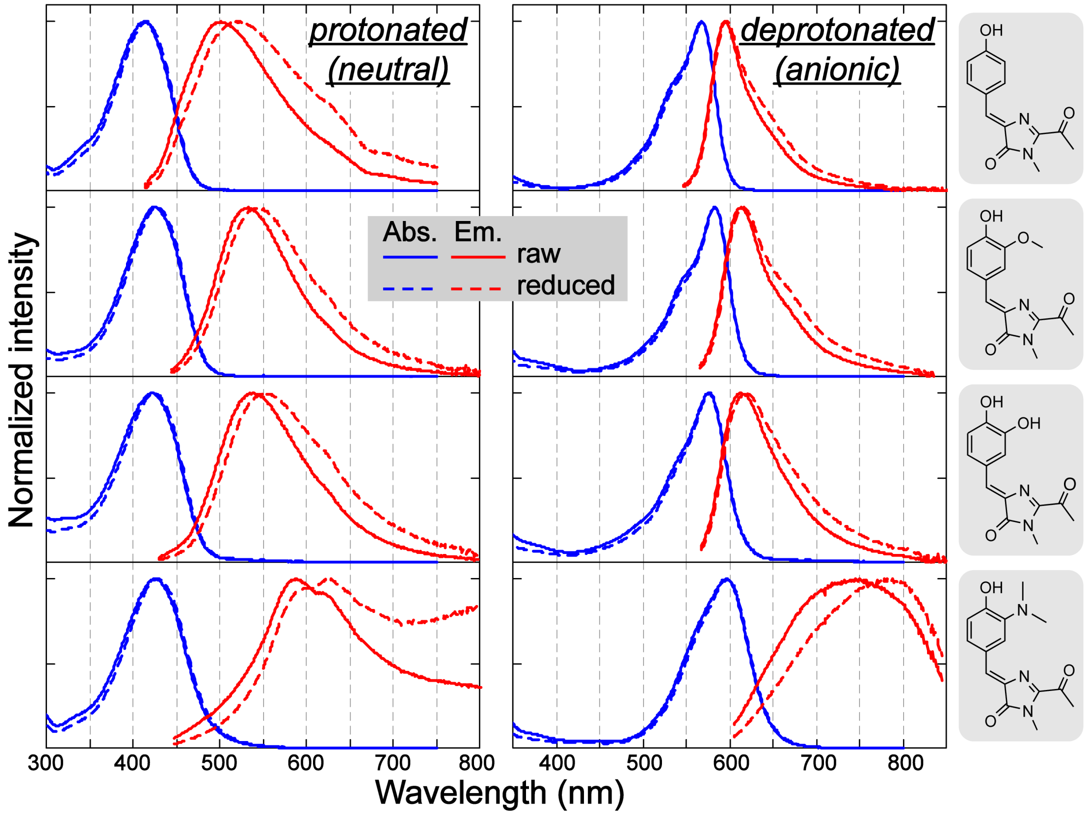

Figure S11. Absorption and emission spectra for the –$COCH_3$ series in acetonitrile. The original absorption (blue) and emission (red) spectra are shown in solid lines. The corresponding reduced spectra (dashed lines) were used to determine the 0-0 transition energy gap for the protonated/neutral (left) and deprotonated/anionic (right) chromophores.

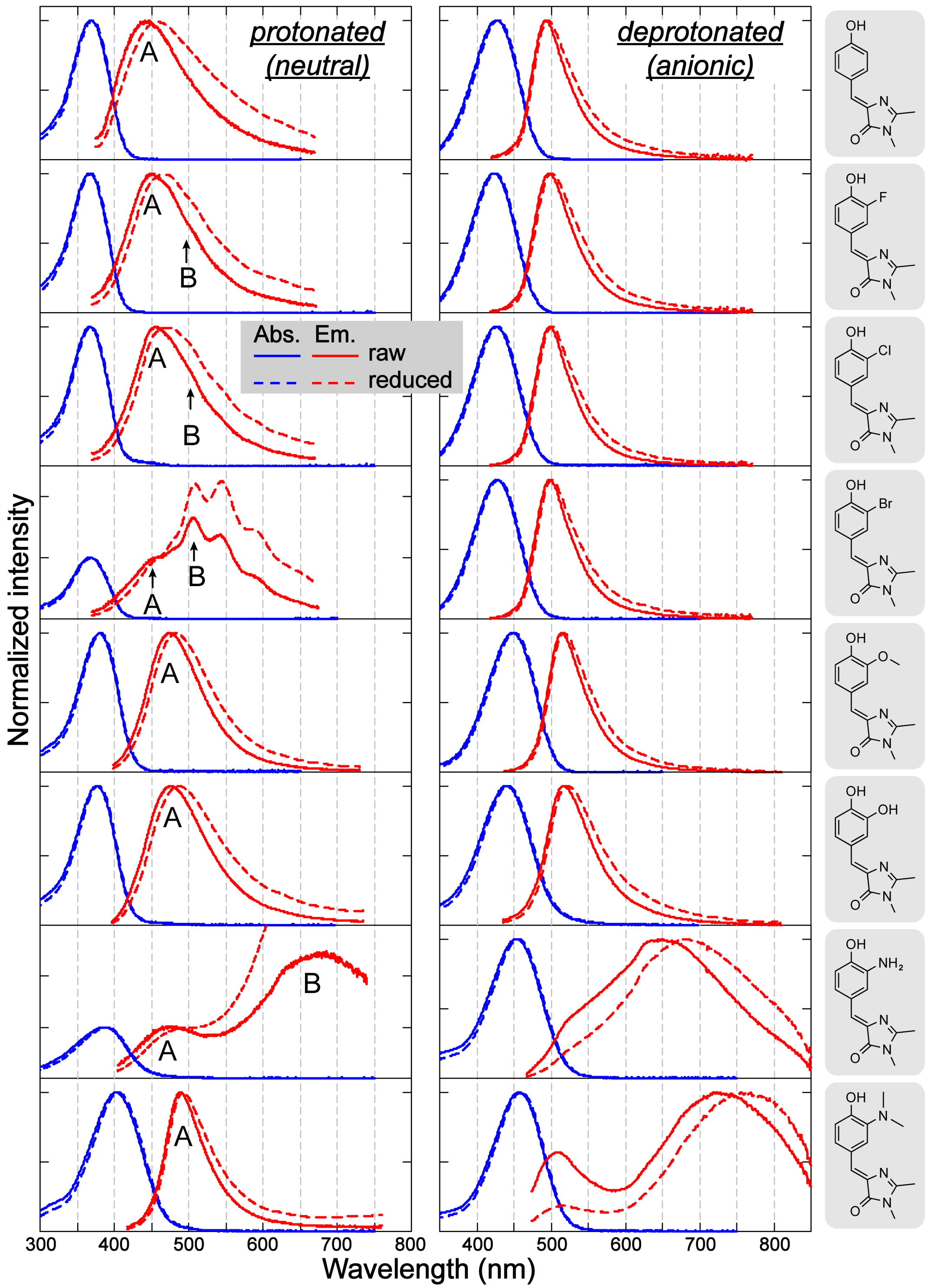

Figure S12. Absorption and emission spectra for the $-CH_3$ series in water. The original absorption (blue) and emission (red) spectra are shown in solid lines. The corresponding reduced spectra (dashed lines) were used to determine the 0-0 transition gap for the protonated/neutral (left) and deprotonated/anionic (right) chromophores. Emission labels: A, neutral; B, anionic chromophores.

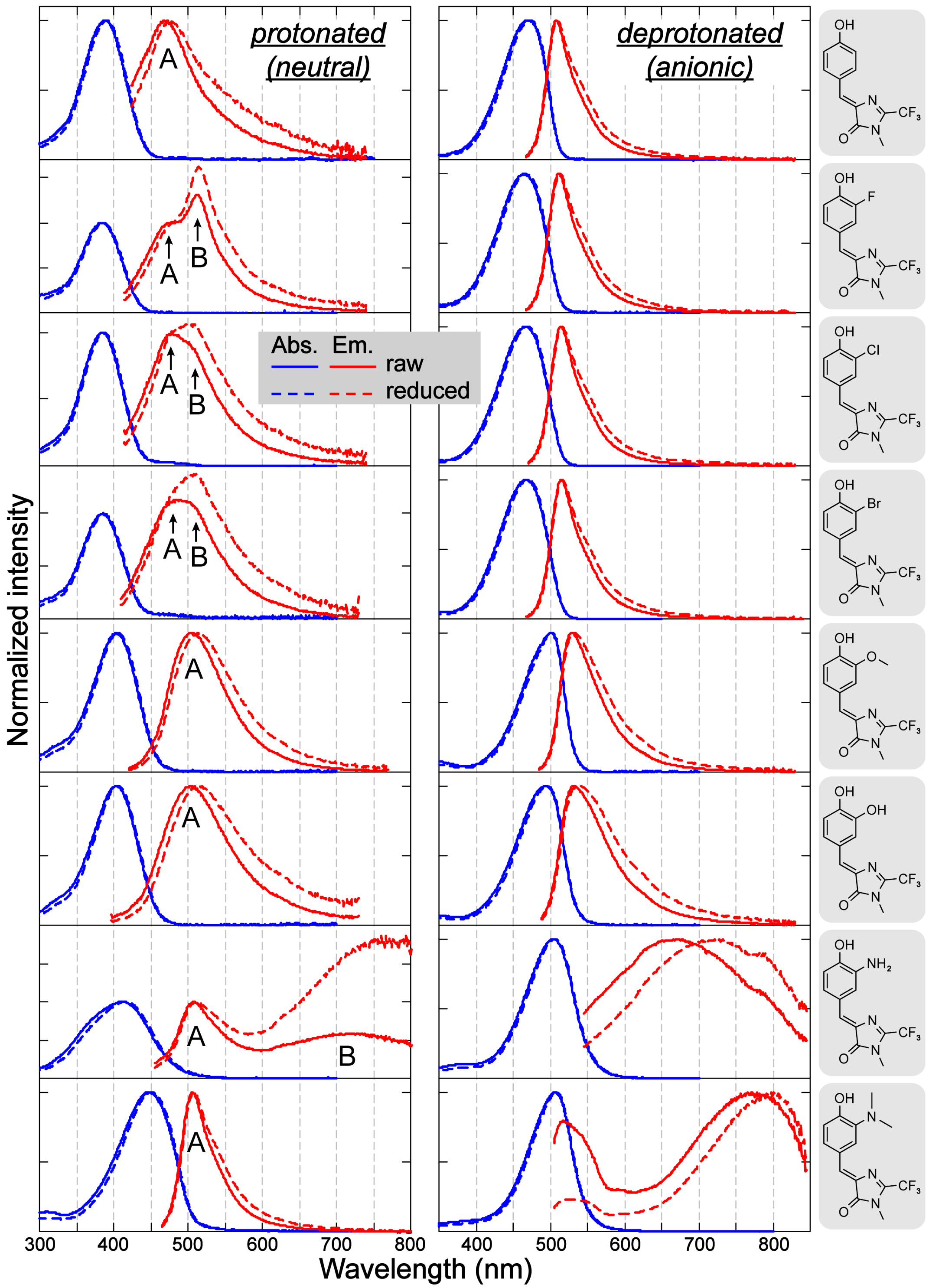

Figure S13. Absorption and emission spectra for the –CF_3 series in water. The original absorption (blue) and emission (red) spectra are shown in solid lines. The corresponding reduced spectra (dashed lines) were used to determine the 0-0 transition gap for the protonated/neutral (left) and deprotonated/anionic (right) chromophores. Emission labels: A, neutral; B, anionic chromophores.

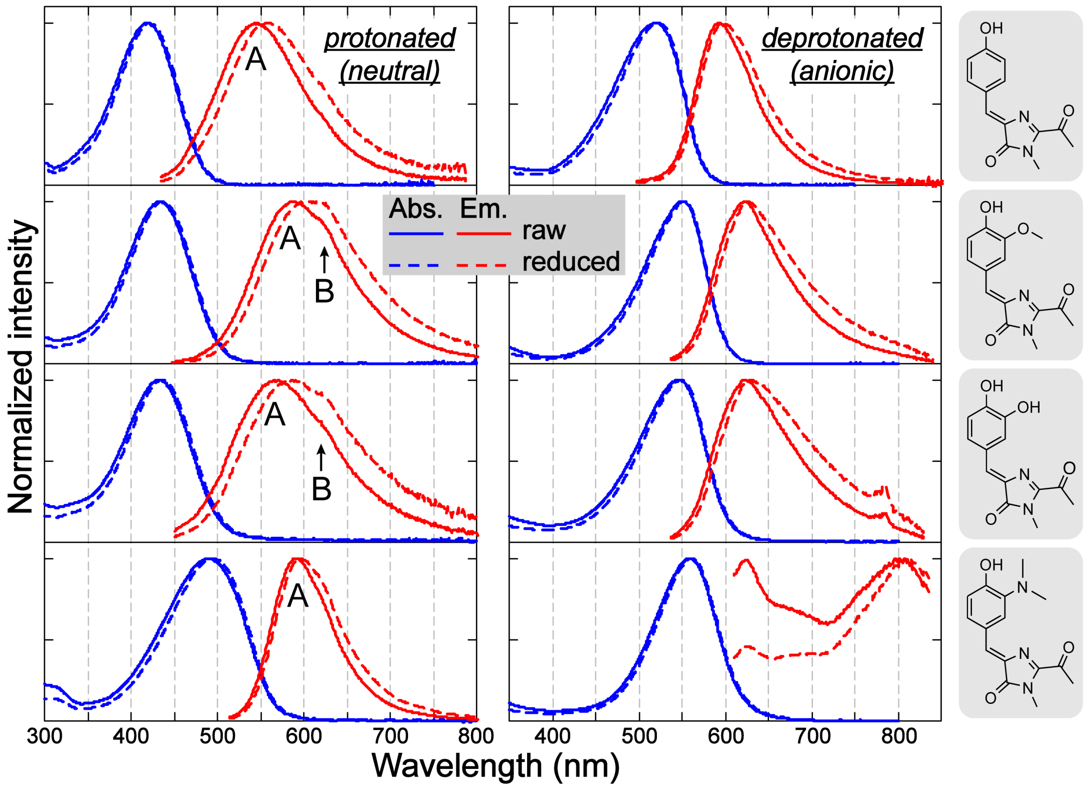

Figure S14. Absorption and emission spectra for the –$COCH_3$ series in water. The original absorption (blue) and emission (red) spectra are shown in solid lines. The corresponding reduced spectra (dashed lines) were used to determine the 0-0 transition energy gap for the protonated/neutral (left) and deprotonated/anionic (right) chromophores. Emission labels are for A, neutral; B, anionic chromophores.

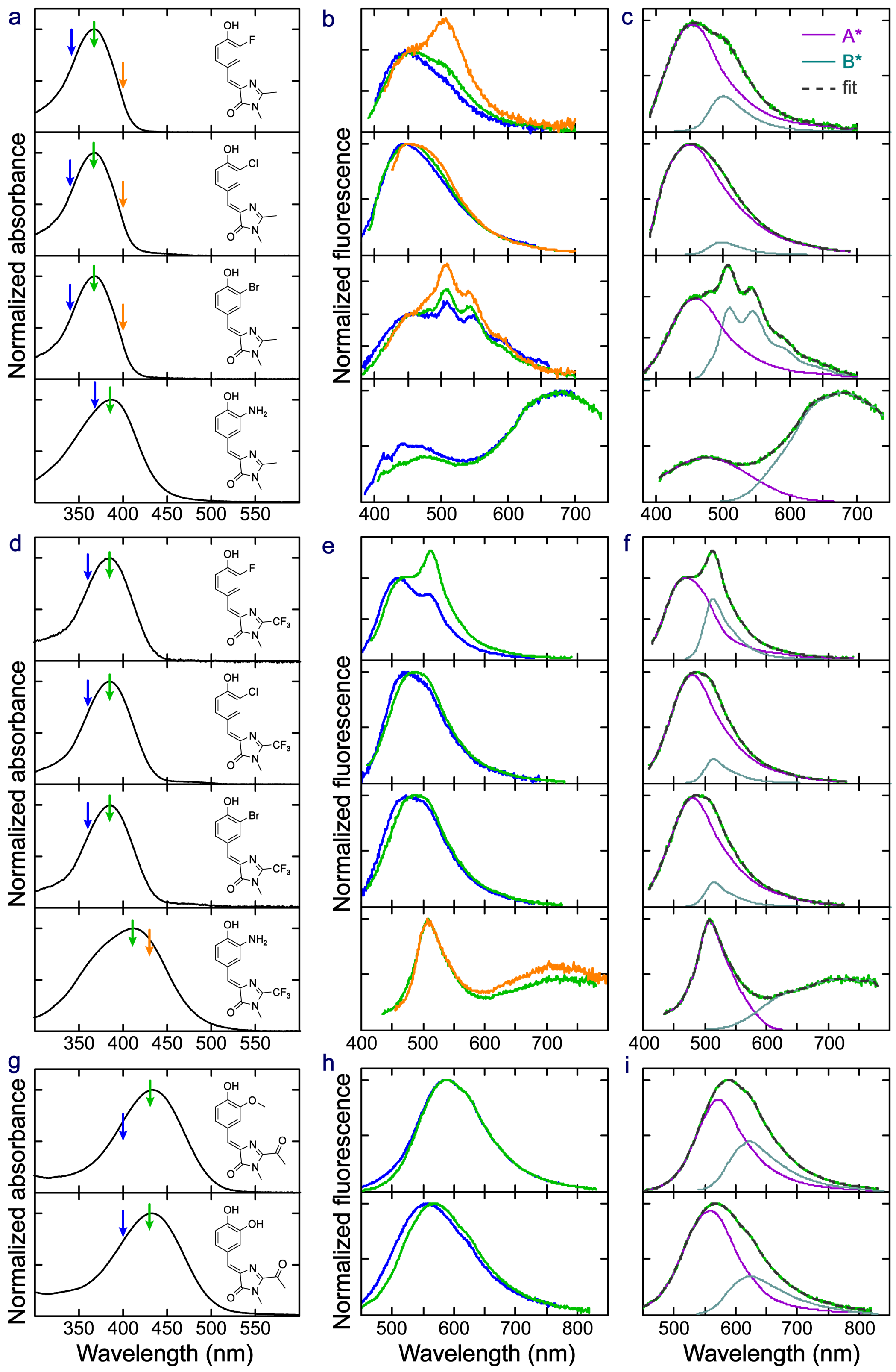

Figure S15. Excitation-dependent steady-state fluorescence spectra of several series of photoacids with spectral decomposition. (a, d, g) Normalized electronic absorption spectra of ESPT-capable

molecules (–CH_3 series, –CF_3 series, –$COCH_3$ series in water) at low pH's with the selected excitation wavelengths denoted by the downward arrows in color. Blue, green to orange colors represent higher to lower energies. (b, e, h) Normalized steady-state fluorescence spectra at different excitation wavelengths as indicated in (a, d, g), respectively. (c, f, i) Spectral decomposition of the fluorescence spectra (shown in the middle column) with excitation wavelengths at the absorption maxima (denoted by green arrows in the left column). The conjugate base spectrum (B* fluorescence) was obtained from the high-pH data wherein the chromophore is fully deprotonated. The fitted overall spectrum is shown by the dashed black line (featuring a good match with the experimental spectrum in green), while the retrieved protonated chromophore spectrum (A* fluorescence) is shown by the magenta solid line in each case.

Notably, the observed fluorescence trend is not as simply expected from excess energy, which highlights the complexity of the nonfluorescent photoacid behaviors that our current endeavors cannot fully resolve. If every compound can precisely follow a theoretical trend, then we did not need to systematically study multiple molecules with various substituents to identify an overall trend (e.g., Figures 1–4 in main text and Figure S15 above). In fact, the F-substituent on P-ring cases may deviate a bit from the general trend due to unique properties of fluorine among the halogens, well-known in organic chemistry. The overall trend remains that the photoacidity leads to ESPT in all the unlocked compounds, which exhibit emissions from both the protonated and deprotonated chromophores upon excitation around the absorption peak of A form. The intricate excitation-dependent nonradiative pathways can be examined by steady-state electronic spectral measurements for these dim, unlocked photoacids in methanol that inhibits ESPT and favors the protonated chromophore emission (i.e., the A* fluorescence, see Figure S27 below). Therefore, it is crucial to consider the effects of excitation wavelength on both the ESPT propensity and ring twists of the protonated chromophore (A*) to fully understand the multidimensional potential energy surfaces of these essentially nonfluorescent photoacids in the electronic excited state.

Furthermore, the B* emission spectrum generated by ESPT in acidic solution may differ from the B* emission by direct excitation of B in basic solution. The environment is essentially different (e.g., H^+ and OH^- concentrations) and, depending on the solute structure and solute-environment interactions, could result in slight or greater changes in spectral properties. If the excited-state lifetime for the deprotonated form is too short to relax (by structural rearrangement, solvation, etc.), the B* spectra by ESPT and direct excitation may not be the same, reminiscent of GFP.[12]

Table S2. Absorption/emission maxima and 0-0 transition gaps of the protonated neutral and deprotonated anionic *p*-HBDI chromophore derivatives in acetonitrile.

R_P	R_I	protonated neutral[a]			deprotonated anionic[a]		
		λ_{abs} / nm	λ_{em} / nm	E^{A}_{0-0} / cm^{-1}	λ_{abs} / nm	λ_{em} / nm	E^{B}_{0-0} / cm^{-1}
–H	$–CH_3$	368	436	25157	461	500	20534
–F		368	428	25316	459	501	20534
–Cl		366.5	428	25349	460	502	20597
–Br		367	430	25316	461	501	20619
–OMe		374	438	24876	477.5	517	19900
–OH		372.5	441	24814	475	513	20121
$–NH_2$		386.5	557	21786	499	575	18939
$–NMe_2$		372.5	626	20367	492	630	18570
–H	$–CF_3$	383	426	24691	508.5	521	19380
–F		379	448	24361	508.5	524	19342
–Cl		380	457	24242	508	525	19342
–Br		380	464.5	24184	506	525	19324
–OMe		395	477	23202	521	536	18921
–OH		395	475.5	23148	516	534	19048
$–NH_2$		419	636.5	19474	535	578	18051
$–NMe_2$		392	731	19724	532	679	17498
–H	$–COCH_3$	413	501	22026	568	594.5	17182
–OMe		425	533	21008	582	613	16750
–OH		422	539	21008	576	613	16835
$–NMe_2$		425	587	20121	596.5	746	15649

[a] The spectra to obtain these values listed in this table for the R_I = $–CH_3$, $–CF_3$, and $–COCH_3$ series of *p*-HBDI chromophore derivatives in acetonitrile can be found in Figures S9, S10, and S11, respectively. The fitting results with respect to various substituent properties can be found in Figure 2c (main text).

Table S3. Absorption/emission maxima and 0-0 transition gaps of the protonated neutral and deprotonated anionic *p*-HBDI chromophore derivatives in water.

R_P	R_I	protonated neutral[a]			deprotonated anionic[a]		
		λ_{abs} / nm	λ_{em} / nm	E_{0-0}^{A} / cm^{-1}	λ_{abs} / nm	λ_{em} / nm	E_{0-0}^{B} / cm^{-1}
–H	$–CH_3$	368	443	24913	426.5	493	21368
–F		367	450	24820	423	497.5	21381
–Cl		367	455	24661	425.5	498	21254
–Br		368	455	24783	427	498	21218
–OMe		380	474	23646	448	514.5	20404
–OH		376	476	23747	439.5	516.5	20521
$–NH_2$		386.5	475	23386	454	650	19425
$–NMe_2$		402.5	489	21867	457	725	19486
–H	$–CF_3$	388.5	470	23507	470	508	20165
–F		385	468	23474	464	512	20137
–Cl		384.5	471	23441	466.5	514	20040
–Br		386	467	23474	467	515	20016
–OMe		403	505	22012	501	528	19357
–OH		403	504	22257	494	531	19372
$–NH_2$		412	507	21097	504	671	18192
$–NMe_2$		447.5	506	20458	506.5	773	18292
–H	$–COCH_3$	419	544	20986	520	592	17848
–OMe		433	588	19763	551	622	17103
–OH		433	567	20145	546	623	17103
$–NMe_2$		489	591	18113	559	803	16892

[a] The spectra to obtain these values listed in this table for the R_I = $–CH_3$, $–CF_3$, and $–COCH_3$ series of *p*-HBDI chromophore derivatives in water can be found in Figures S12, S13, and S14, respectively. The fitting results with respect to various substituent properties can be found in Figure S20 (see below).

S5. Determination of pK_a, ΔpK_a and pK_a^*

S5.1 Ground-state pK_a titration

The ground-state pK_a was measured using UV/Vis absorption spectroscopy based on the Beer-Lambert law and Henderson–Hasselbalch equation,

$$\mathrm{p}K_\mathrm{a} = \mathrm{pH} + log_{10}\left(\frac{c_A}{c_B}\right) \tag{S3}$$

where c_A and c_B are the concentrations of the acid (A) and conjugate base (B), respectively. Thus, pK_a is the pH at which A and B have the same concentration. The concentration can be linearly connected to the absorbance by Beer-Lambert law, absorbance $= \varepsilon \cdot c \cdot \mathrm{l}$, where ε and l represent the wavelength-dependent extinction coefficient and pathlength of light, respectively. The absorbance is measured in optical density (OD), a unitless quantity. In practice, the solution with the same total concentration $c_A + c_B$ of solute is measured for absorption spectrum at different pH values with small increments. A clear isosbestic point should be seen between the absorption peaks of the acid and conjugated base. To detemine the pK_a value, a titration curve (A or B) or two titration curves (A and B) plotting the molar ratio (c_A and/or c_B over the total concentration) against pH should be generated. The molar ratio R is determined by

$$R = \frac{A - A_{min}}{A_{max} - A_{min}} \tag{S4}$$

where A_{max} and A_{min} are the maximal and minimal absorbance across the pH range, respectively. The values of A, A_{max}, and A_{min} are taken for acid and conjugate base at their peak wavelengths to calculate the respective R value. The molar ratios for the acid and conjugated base follow and can be fitted by

$$R_A = \frac{1}{1 + 10^{(\mathrm{pH} - \mathrm{p}K_\mathrm{a})}} \tag{S5}$$

$$R_B = 1 - \frac{1}{1 + 10^{(\mathrm{pH} - \mathrm{p}K_\mathrm{a})}} \tag{S6}$$

The pK_a is determined by the pH value at the crossing point of the two experimental titration curves (R_A and R_B), which should correspond to the molar ratio of 0.5 (i.e., $R_A = R_B = 0.5$).

S5.2 Calculations of ΔpK_a and pK_a^* by Förster equation with our revised definition

The conventional ΔpK_a and pK_a^* are calculated by Förster equation. By treating ESPT as a quasi-equilibrium reaction, pK_a^* can be calculated by $pK_a^* = \Delta G^{\circ *}/(RT \ln 10)$. Since the acid (A) and conjugated base (B) of the *p*-HBDI-derived photoacids in this work have similar coplanar structures (despite being conformatioally flexible) as suggested by calculations, it is reasonable to assume that the entropy change ($\Delta S^{\circ *}$) contributes minimally to the free energy change ($\Delta G^{\circ *}$) as compared to the enthalpy change ($\Delta H^{\circ *}$) in the electronic excited state. Therefore, the free energe change can be approximated as the zero-point energy difference between A and B. The acidity difference (ΔpK_a) between the excited and ground states can thereby be linked to the 0–0 transition energies for A (E_{0-0}^{A}) and B (E_{0-0}^{B}),

$$\Delta pK_a = pK_a - pK_a^* = \frac{\Delta G^{\circ} - \Delta G^{\circ *}}{RT \ln 10} = \frac{E_{0-0}^{\mathrm{A}} - E_{0-0}^{\mathrm{B}}}{RT \ln 10} \qquad \text{(S7)}$$

The 0–0 transition energies can be obtained by crossing the normalized reduced absorption and emission spectra as described in Section S4.2 or more rigorously by spectroscopic hole-burning experiments.[37,38] In this work, we used the former approach to acquire the 0–0 transition energies in a systematic manner. With these measurable quantities from the same setup, we can obtain ΔpK_a and pK_a^* with the experimental determination and knowledge of the titrated ground-state pK_a.

Our analysis of kinetics for the extensive series of *p*-HBDI-derived photoacids reveals the inadequacy of conventional pK_a^* by Förster equation in consistently explaining the ESPT kinetics. We argue that the inconsistency is likely due to the breakdown of the quasi-equilibrium assumption for the essentially nonfluorescent *p*-HBDI-derived photoacids.[39-41] As a result, thermodynamic principles do not apply any more as the sole contributor. We thereby propose the addition of Franck-Condon relxation energy ($E_{\mathrm{FC}}^{\mathrm{A}}$) of the acid form that can phenomenologically account for the difference between thermodynamics and kinetics. The revised pK_a^* expression is the following,

$$pK_a^* = pK_a - \Delta pK_a = pK_a - \frac{E_{\mathrm{FC}}^{\mathrm{A}} + E_{0-0}^{\mathrm{A}} - E_{0-0}^{\mathrm{B}}}{RT \ln 10} = pK_a - \frac{E_{\mathrm{ex}}^{\mathrm{A}} - E_{0-0}^{\mathrm{B}}}{RT \ln 10} \qquad \text{(S8)}$$

The new definition requires $E_{\mathrm{ex}}^{\mathrm{A}}$ (absorption energy, >0) and E_{0-0}^{B} (>0), both of which are readily accessible by steady-state electronic absorption and emission spectra. Further approximations can be made to estimate pK_a^*. For example, if the conjugate base exhibits a tiny Stokes shift, the E_{0-0}^{B} can be approximated as the emission energy ($E_{\mathrm{em}}^{\mathrm{B}}$). This way the ESPT driving force magnitude

(see ΔE_{ESPT} in Figure 3b, main text, by definition it is negative to indicate an exergonic reaction) can be calculated by replacing $(E_{\text{ex}}^{\text{A}} - E_{0-0}^{\text{B}})$ (i.e., a positive value) in the aforementiond Equation S8 with the overall Stokes shift $(E_{\text{ex}}^{\text{A}} - E_{\text{em}}^{\text{B}})$ (i.e., also a positive value) upon excitation of the acid. The $\Delta\text{p}K_{\text{a}}$ values calculated and tabulated below (Table S4) are thus positive, and $\text{p}K_{\text{a}}^{*} < \text{p}K_{\text{a}}$.

We also examined the pK_a^* values with ΔpK_a calculated by using the electronic absorption energy, emission energy, and averaging the absorption and emission energies as commonly used in the conventional Förster equation (Table S4). Among them, averaging absorption and emission energies has usually been considered more accurate than using absorption or emisison energy alone due to its proximity to the 0–0 transition energy gap. However, the pK_a^* values obtained by these three approaches all result in poor consistencies with the kinetics (e.g., R_P = –NMe_2); such inconsistencies remain for those pK_a^* values calculated by the more accurately determined 0–0 transition gaps (e.g., R_P = –NH_2 and –NMe_2). These results suggest that the 0–0 transition gaps or the approximated values by either averaging the absorption and emission energies or the absorption/emission energy alone do not accurately represent the ESPT driving force (pK_a^*) using conventional Förster equation. In contrast, the use of Stokes shift leads to better results (consistent in trend but not in absolute values), but it still fails for the –NMe_2 substituent whose predicted pK_a^* is too low in contradiction to its incapability of ESPT in water (Figures S12–S14). Using the above-mentioned new revised energy term (i.e., $E_{\text{ex}}^{\text{A}} - E_{0-0}^{\text{B}} = E_{\text{FC}}^{\text{A}} + E_{0-0}^{\text{A}} - E_{0-0}^{\text{B}} = E_{\text{FC}}^{\text{A}} + \Delta E_{0-0}$), the best match can be achieved (see listed values in Table S4, and relevant discussions in main text).

In addition, we noted that the photoacids with R_P substituent of –NH_2 and –NMe_2 in the deprotonated anionic form have asymmetric absorption and emission spectra. The deviation from the mirror-image law likely makes it less accurate in determining the 0–0 transition gap using Equations S1 and S2 directly. We thereby used the average of absorption and emission energies to approximate the 0–0 transition energy gap (E_{0-0}^{B}) for the deprotonated anionic form (see Table S4 with footnotes below). The resultant pK_a^* values under the revised definition remain largely consistent with observed kinetics, supporting our new insights and proposal that the Franck-Condon excess/relaxation energy of the acid (protonated neutral form) should be additionally included to account for the ESPT driving force and hence kinetics for this type of "dim" photoacids (i.e., with low FQY as discussed in main text with Figure 4d).

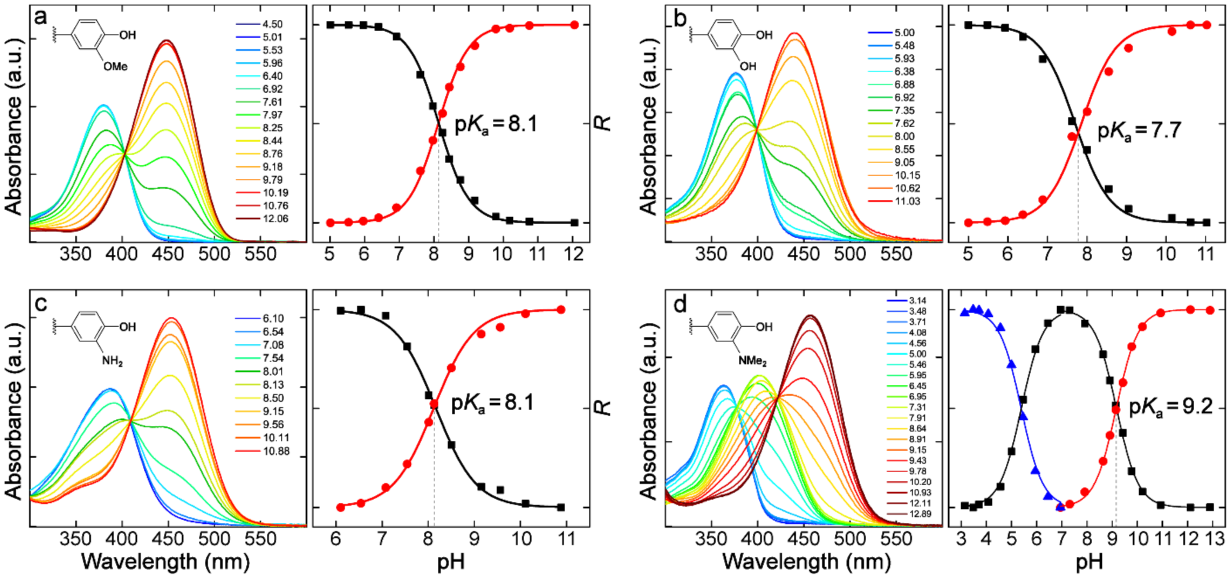

Figure S16. pK_a titration for the –CH_3 series in water. The structures of the P-ring moiety are shown in the insets with (a) –OMe, (b) –OH, (c) –NH_2, and (d) –NMe_2 substituents. Black and red titration curves denote the molar ratios for the acid (–OH) and conjugate base (–O^-) forms, respectively; their crossing point is highlighted by the gray vertical dotted line with the measured pK_a value listed nearby. The NMe_2-substituted photoacid has another lower pK_a in the titrated pH range resulting from the protonation of –NMe_2 group (blue titration curve in the bottom right panel represents the cationic form of the chromophore).

Notably, the pK_a titrations for the other four P-ring substituents (–H, –F, –Cl, –Br) have been reported in our previous publication with the experimentally determined pK_a values of 8.4, 6.9, 6.7, and 6.6, respectively.[3]

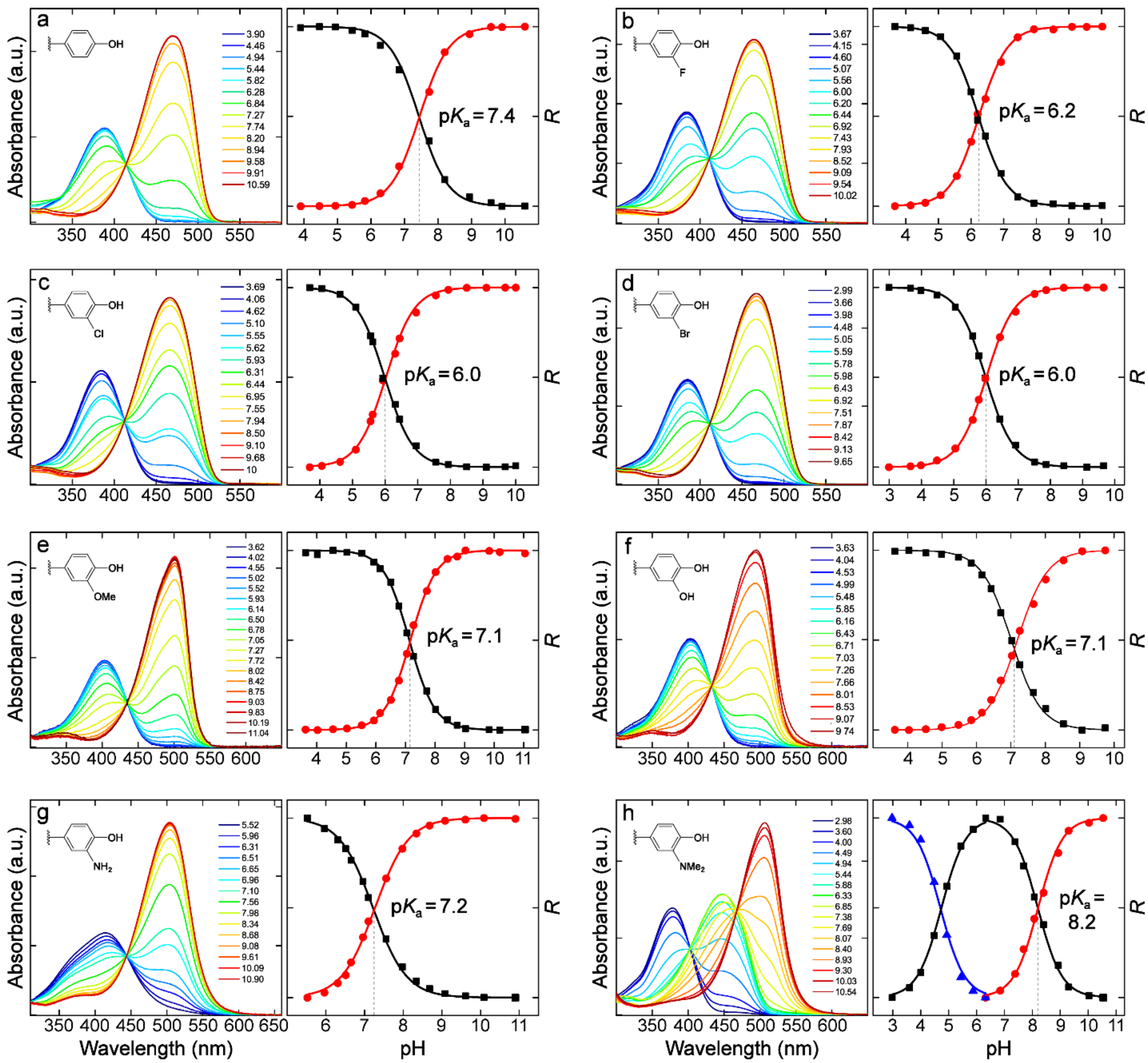

Figure S17. pK_a titration for the –CF_3 series in water. The structure of the P-ring moiety is shown in the insets with (a) –H, (b) –F, (c) –Cl, (d) –Br, (e) –OMe, (f) –OH, (g) –NH_2, and (h) –NMe_2 substituents. Black and red titration curves denote the molar ratios for the acid (–OH) and conjugate base (–O^-) forms, respectively; their crossing point is highlighted by the gray vertical dotted line with the measured pK_a value listed nearby. The NH_2- and NMe_2-substituted photoacids have another lower pK_a in the titrated pH range resulting from the protonation of amine group (titration curves for –NH_2 are not shown); blue titration curve in the bottom right panel represents the cationic form of the chromophore (panel h).

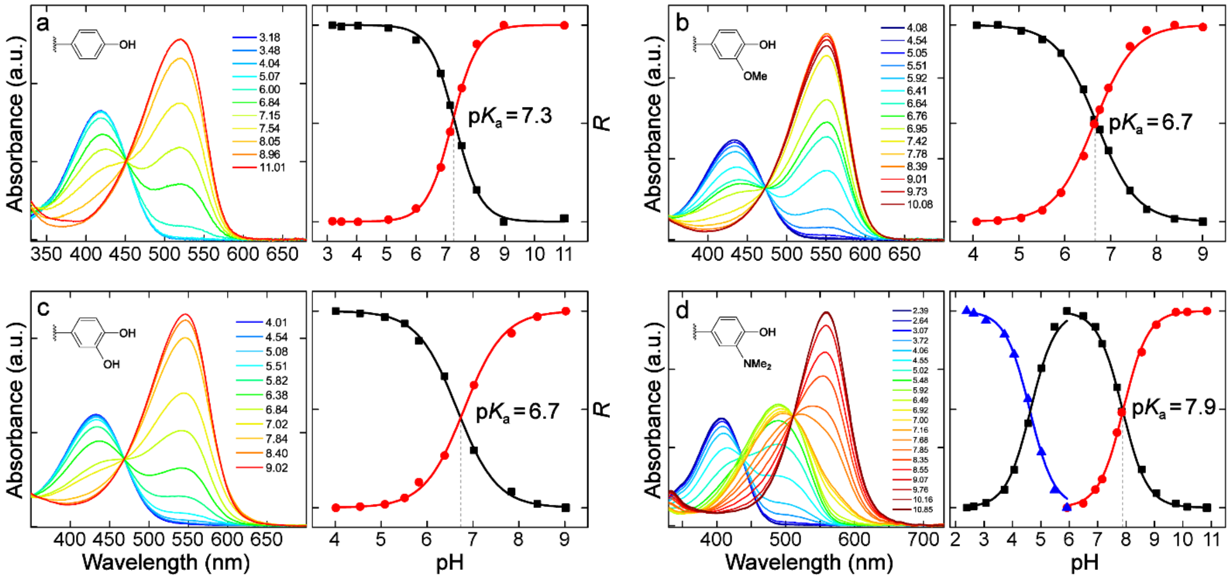

Figure S18. pK_a titration for the –$COCH_3$ series in water. The structure of the P-ring moiety is shown in the insets with (a) –H, (b) –OMe, (c) –OH, and (d) –NMe_2 substituents. Black and red titration curves denote the molar ratio for the acid (–OH) and conjugate base (–O^-) forms, respectively; their crossing point is highlighted by the gray vertical dotted line with the measured pK_a value listed nearby. The NMe_2-substituted photoacids have another lower pK_a in the titrated pH range resulting from the protonation of –NMe_2 group (blue titration curve in the bottom right panel represents the cationic form of the chromophore).

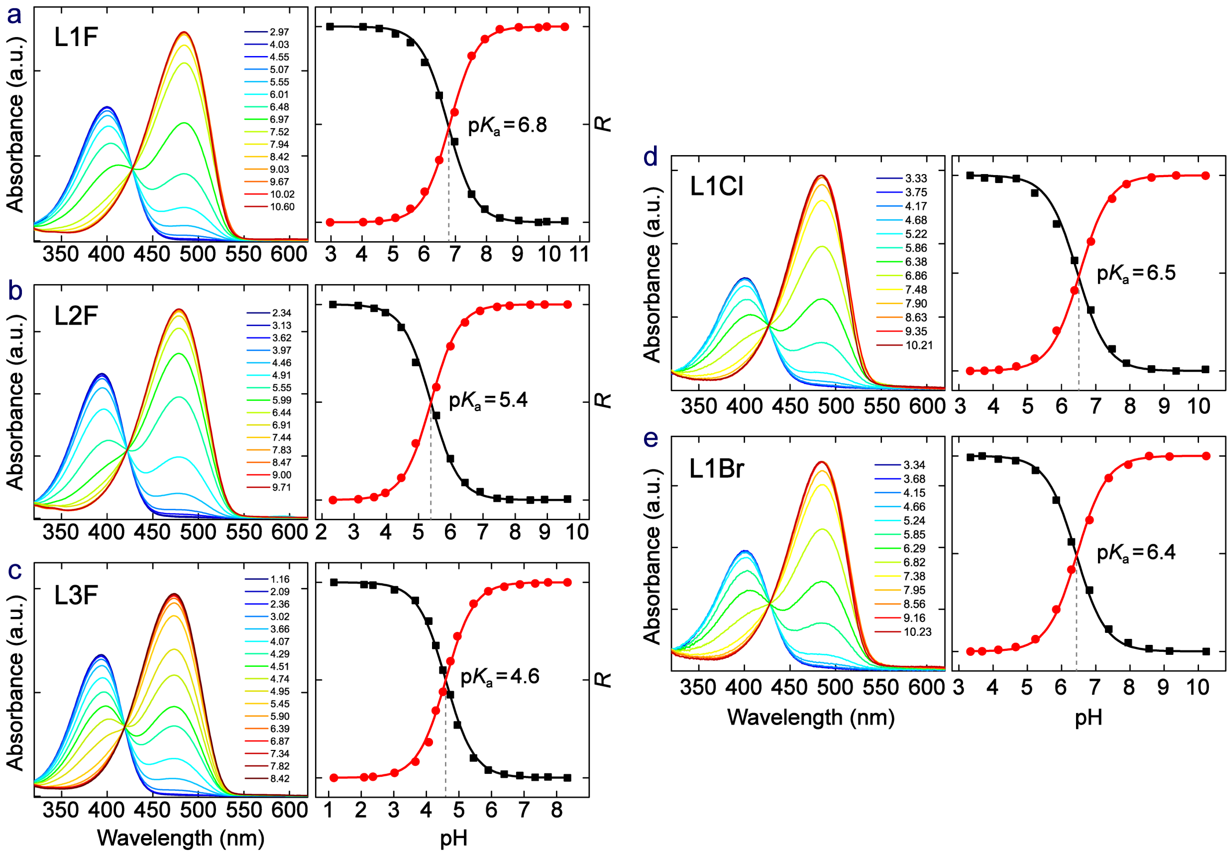

Figure S19. pK_a titration for the locked photoacids in water. Black and red titration curves denote the molar ratio for the acid (–OH) and conjugate base (–O$^-$) forms of (a) L1F, (b) L2F, (c) L3F, (d) L1Cl, and (e) L1Br, respectively; their crossing point is highlighted by the gray vertical dotted line with the measured pK_a value listed nearby in each right panel.

Table S4. pK_a, ΔpK_a, pK_a^* values using different methods for unlocked photoacids in water.

R_P	R_I	pK_a	ΔpK_a	pK_a^{*a}					
		titration	$E_{FC}^{A} + \Delta E_{0-0}$		ΔE_{0-0}	E_{SS}	E_{abs}	E_{em}	$\frac{E_{abs} + E_{em}}{2}$
–H	$-CH_3$	8.4	12.2	–3.8	1.0	–6.0	0.6	3.6	2.1
–F		6.9	12.3	–5.4	–0.3	–8.1	–0.7	2.5	0.9
–Cl		6.7	12.6	–5.9	–0.4	–8.3	–1.2	2.7	0.8
–Br		6.6	12.5	–5.9	–0.9	–8.3	–1.3	2.6	0.7
–OMe		8.1	12.4	–4.3	1.3	–6.3	–0.3	4.6	2.2
–OH		7.7	12.7	–5.0	0.9	–7.5	–0.4	4.2	2.0
$-NH_2$		8.1	13.5 (15.0)[b]	–5.4 (–6.9)[b]	–0.2	–13.9	0.0	–3.8	–1.9
$-NMe_2$		9.2	11.2 (14.7)[b]	–2.0 (–5.5)[b]	4.2	–14.0	3.0	–4.8	–0.9
–H	$-CF_3$	7.4	11.7	–4.3	0.4	–5.3	–2.0	4.1	1.1
–F		6.2	12.2	–6.0	–0.8	–7.3	–3.1	2.4	–0.4
–Cl		6.0	12.5	–6.5	–1.1	–7.7	–3.6	2.3	–0.7
–Br		6.0	12.4	–6.4	–1.2	–7.6	–3.4	1.8	–0.8
–OMe		7.1	11.4	–4.3	1.5	–5.2	–3.1	5.3	1.1
–OH		7.1	11.4	–4.3	1.1	–5.4	–2.5	5.0	1.3
$-NH_2$		7.2	12.7 (14.5)[b]	–5.5 (–7.3)[b]	1.1	–12.4	–2.1	–2.9	–2.5
$-NMe_2$		8.2	8.5 (12.6)[b]	–0.3 (–4.4)[b]	3.7	–11.5	2.7	–6.1	–1.7
–H	$-COCH_3$	7.3	12.6	–5.3	0.7	–7.3	–2.4	4.2	0.9
–OMe		6.7	12.6	–5.9	1.1	–8.0	–3.7	4.8	0.5
–OH		6.7	12.6	–5.9	0.3	–8.1	–3.3	3.4	0.0
$-NMe_2$		7.9	7.5 (11.1)[b]	0.4 (–3.2)[b]	5.3	–8.9	2.5	–1.5	0.5

[a] The pK_a^* values are calculated with ΔpK_a (defined as a positive number, see Equations S7 and S8 above) obtained using different energies including: revised transition energy ($E_{FC}^{A} + \Delta E_{0-0}$, where $\Delta E_{0-0} = E_{0-0}^{A} - E_{0-0}^{B}$); 0–0 transition energy (ΔE_{0-0}); Stokes shift (E_{SS}); absorption energy ($E_{abs}^{A} - E_{abs}^{B}$); emission energy ($E_{em}^{A} - E_{em}^{B}$); and the averaged absorption and emission energy $[\frac{(E_{abs}^{A}+E_{em}^{A})}{2} - \frac{(E_{abs}^{B}+E_{em}^{B})}{2}]$. The measured p$K_a$ values of the photoacids (A form) in water can be found in Figures S16–S18 above.

[b] The pK_a^* values for $-NH_2$ and $-NMe_2$ substituents in red are obtained by approximating E_{0-0}^{B} as $(E_{abs}^{B} + E_{em}^{B})/2$. The resultant p$K_a^*$ values are typically smaller (with higher photoacidity) than those using E_{0-0}^{B}.

S6. Multivariable linear models

S6.1 Substituent parameters and linear regression models in literature

Historically, the interest in quantitatively characterizing the substituent properties stemmed from studying the mechanisms of organic reactions. A useful strategy is to modify the structure of a parent molecule through systematic substitutions. One of the earliest and most important achievements is the Hammett equation where Louis P. Hammett defined the so-called Hammett parameter σ to quantify the electronic impact of the substituent on the rate or equilibrium constant of a chemical reaction. Since the Hammett parameter only concerns the electronic effect of a substituent, Taft developed a few other parameters characterizing steric effect E_S and the inductive and resonance effects.[42,43] The latter two can be linearly combined to give rise to the *para* (σ_p) and *meta* (σ_m) Hammett parameters. The *ortho* substituent should have the same electronic effect as its *para* counterpart as a result of the push-pull mechanism but due to the steric hindrance for an *ortho* substituent, a corresponding *ortho* Hammett parameter "σ_o" has seldom been discussed.[44] Later, the hydrophobic effects of organic molecules were found to be important when interacting with biological systems. This finding stimulated the development of pertinent parameters log P (P is the octanol/water partition coefficients) and π, which work for a whole molecule and substituent, respectively.[45] The mathematical quantifications of electronic, steric, and hydrophobic effects of substituent have facilitated the development of quantitative structure-activity relationships (QSAR) and broad applications particularly in biochemical and pharmaceutical fields such as drug design.[46] A similar term is the quantitative structure-property relationships (QSPR). The QSAR/QSPR models typically use linear relationships to correlate targeted biological activity/chemical property to substituents and are relatively easy to interpret and make quantitative predictions. Therefore, QSAR/QSPR has achieved broad success since its original formulation. The later development of QSAR/QSPR models has encompassed more substituent parameters to further improve the accuracy in the prediction of a variety of biological activities or chemical properties.[47]

S6.2 Justification of using Hammett, Charton, and hydrophobicity parameters

In this work, we aim to linearly correlate the central properties for photoacids, color and acidity (ground and excited states), to the electronic, steric, and hydrophobic effects of the substituent with the mathematical expression shown in main text. We assume no or minimal cooperativity between different effects such that they can be linearly summed using the literature values because

the numerical values of these parameters in literature were individually obtained by fixing other influencing effects for specific reactions. For the photoacids of this work, we sampled the substituent *ortho* to the conserved –OH/–O$^-$ group (i.e., part of the original GFP model *p*-HBDI chromophore) and thus the steric effect should also be considered as part of the "intramolecular" substituent effect. We used σ_p to represent the electronic effect (by push-pull mechanism) and Charton steric parameter (V) to quantify the steric effect. Taft steric parameter E_S was not used because it received concerning criticisms, although it can be related to Charton steric parameter.[48] V is defined on the basis of van der Waals radius, which is usually considered as a measure of atomic size; therefore, it has been considered as being independent of specific chemical reactions which instead have underpinned the determination of E_S. Therefore, σ_p and V primarily constitute the intramolecular effects. Meanwhile, the intermolecular effects can be represented by the hydrophobic parameter (π).[45] Though the origin of hydrophobic effect is not fully understood, it is commonly accepted that the entropic effect is the main cause that dominates the free energy change. The hydrogen (H-)bonding may play a major role for such interactions. Our observation that the substituents of $-NH_2$ and –OH which are hydrophilic exhibit dramatic deviations from the linear relationship between pK_a and σ_o^I (see Figure 3a in main text) suggests that intermolecular effects induced by the substituent are also important for proton transfer reactions in water by modifying the energetics of the acid and conjugate base in the electronic ground state (S_0). The improvement in linearity by including π in multivariable linear regression analysis supports the assertion that hydrophobic interaction is a good representation of the pertinent intermolecular effects at thermal equilibrium (the still-deviated fluorine case implies other substituent properties that are prominent for fluorine but marginally contribute for other substituents).

S6.3 Correlating 0–0 transition gap, pK_a, ΔpK_a, and pK_a^* to substituent properties

We first correlated the 0–0 transition energy gaps to the three parameters (σ_p, V, π) by linear regression for the series of photoacidic chromophores in water. The fitting yields a good linearity but shows that the intermolecular effect (π) plays a negligible role due to its tiny coefficient. It is likely due to the cancelation of solute-solvent interactions between the ground and excited states for the same molecule. For the corresponding regression analysis in the solvent acetonitrile, the π parameter is no longer appropriate as it was derived for water as solvent. We thus performed the linear correlation only to σ_p and V parameters (Figure 2c) as shown by Equation 1 in main text

assuming that intermolecular effect contributes as negligibly as that in water. However, we found a poor linearity with all the substituents included and realized that some substituents may form H-bonds with the acetonitrile solvent molecule while others cannot. We then identified the substituent –OH (an H-bond donor) to be an outlier to the linearity, suggestive of its non-negligible H-bonding interaction with acetonitrile (an H-bond acceptor at the –CN site). For comparison, the substituent $–NH_2$ is a potential H-bond donor that may form an H-bond with acetonitrile, but it does not or marginally affect the linearity (see Figure 2c in main text), indicating that an H-bond is either non-existent or too weak to be influential with the $–NH_2$ substituent.

In contrast to the 0–0 transition energy gap that exhibits negligible π dependence, pK_a, ΔpK_a, and pK_a^* demonstrate considerable contributions from the intermolecular effect (π), suggesting that the acid and conjugate base are stabilized by water to different extents due to the substitution of the photoacidic compounds both in the electronic ground and excited states. The steric effect becomes more prominent on pK_a, ΔpK_a, and pK_a^* (see Figure 3d in main text) in comparison to the 0–0 transition gap (Figure 2d). These contrasting experimental plus modeling results highlight the different substituent effects on the properties of the same molecule (i.e., intramolecular) and chemical reaction involving different molecules (e.g., intermolecular proton transfer).

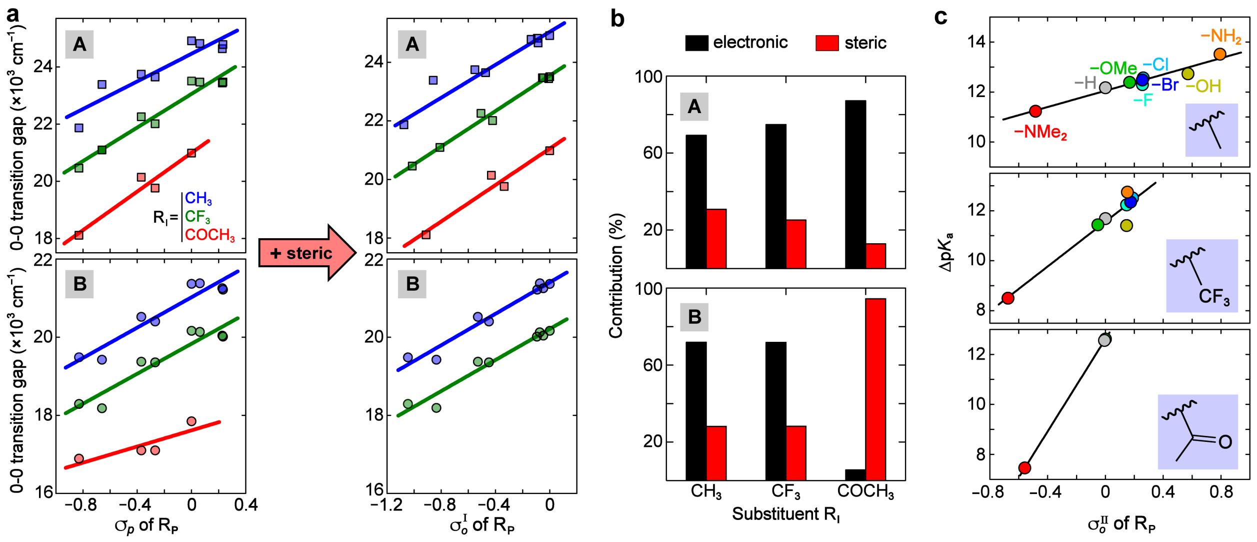

Figure S20. Correlations between optical properties and substituent parameters for the unlocked (nonfluorescent) photoacids in water. (a) The experimentally determined 0-0 transition energy gap values for the acid (–OH, labeled as A in the gray box; data points shown as squares in top panels) and conjugate base (–O$^-$, labeled as B in the gray box; data points shown as circles in bottom panels) are plotted against Hammett parameters σ_p of R_P (left panels) and σ_o^{I} of R_P (i.e., plus steric effects, right panels). The three R_I substituents of $–CH_3$, $–CF_3$, and $–COCH_3$ are colored in blue, green, and red, respectively. The experimental values can be found in Table S3 above. The corresponding linear relationship for the R_P series with each R_I substituent is denoted by the color-coded straight line (i.e., a positive correlation). The conjugate base form of the $R_I = –COCH_3$ series yields a drastically different slope (Supplementary Table S6) and is not shown in bottom right panel. (b) Histograms of electronic (black) and steric (red) contributions (sum to 100%) to the 0–0 transition gaps with three different R_I substituents for the A form (top) and B form (bottom panel). The retrieved coefficients can be found in Table S6. (c) Correlations between ΔpK_a and the newly defined *ortho* parameter σ_o^{II} with electronic, steric, and intermolecular interactions of the substituents included (see Equation 2 and related discussions in main text, with the retrieved coefficients listed in Table S7). The resultant pK_a^* values and plots can be found in Figure 3c in main text (using the measured pK_a values in Figure 3a).

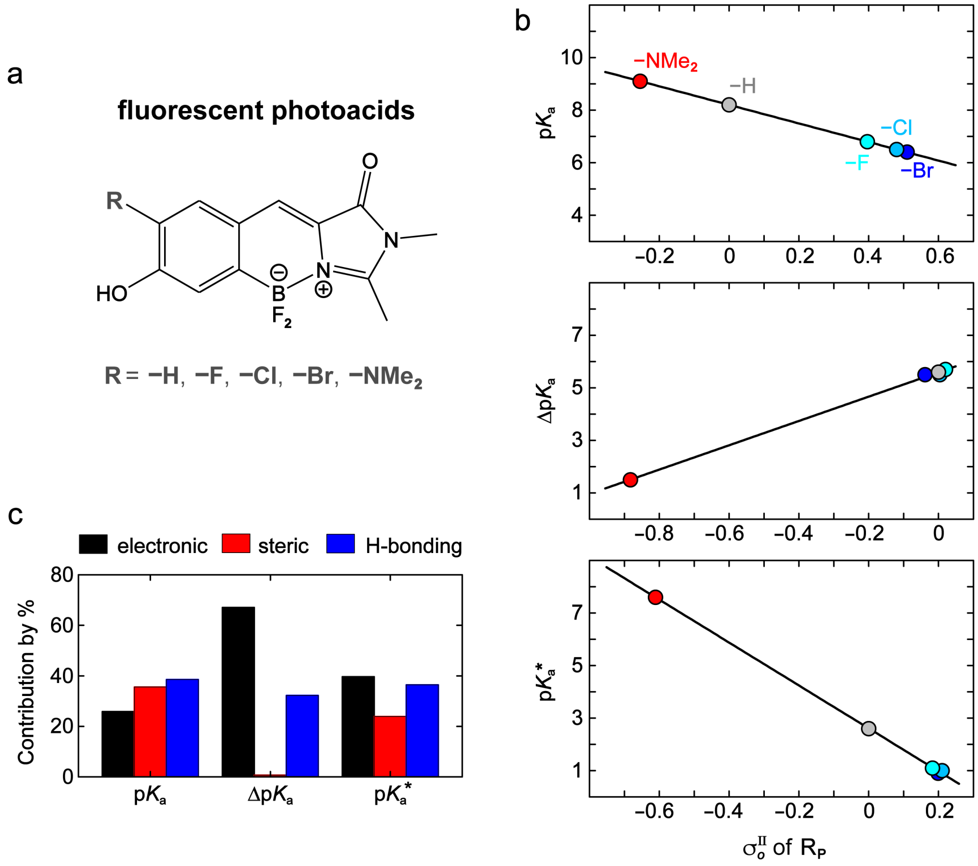

Figure S21. Correlations between optical properties and substituent parameters for the locked (fluorescent) photoacids in water. (a) Chemical structures of the five singly substituted locked photoacids: L0F, L1F, L1Cl, L1Br, and L1NMe2. (b) Correlations between pK_a, ΔpKa, pK_a^* (Table S9) and the newly defined *ortho* parameter σ_o^{II} with electronic, steric, and intermolecular H-bonding interactions of the substituents included (see Equation 2 in main text). (c) Electronic (black), steric (red), and intermolecular H-bonding (blue) contributions (sum to 100%) to the changes of pK_a, ΔpKa, pK_a^*. The retrieved coefficients can be found in Table S8 below.

Like the unlocked compounds, the pK_a values of the locked compounds exhibit a good linear correlation with the electron-withdrawing, steric, and H-bonding capability of the substituent. Using 0–0 transition gaps to compute ΔpK_a and pK_a^*, we again obtained their linear correlations to three parameters of the substituent (see Table S5 above and Equation 2 in main text). Their ESPT rate constants (obtained by transient absorption data, Figures S22 and S23) also follow the linear BEP relationship (Figure 4c). These results substantiate our multivariable linear regression analysis of acidity/photoacidity-substituent relationships and the model that ESPT driving force for fluorescent photoacids can be estimated by the conventionally used free energy difference.

Table S5. Electronic, steric, and hydrophobic parameters for multivariable linear analysis.

substituent	Hammett (σ_p)[a]	Charton (V)[b]	hydrophobicity (π)[c]
–H	0	0	0
–F	0.062	0.27	0.14
–Cl	0.227	0.55	0.72
–Br	0.232	0.65	0.86
–OMe	–0.268	0.36	–0.02
–OH	–0.37	0.32	–0.67
$–NH_2$	–0.66	0.35	–1.23
$–NMe_2$	–0.83	0.43	0.18

[a] The Hammett parameters were taken from references [43,49].

[b] The Charton steric parameters were taken from references [48,49].

[c] The hydrophobicity parameters were taken from references [45,49].

Table S6. Multivariable linear regression for the 0-0 transition energy gaps of the unlocked *p*-HBDI derivatives.

Property	Coeff.	$-CH_3$			$-CF_3$			$-COCH_3$		
		value[b]	*p*-value	R^2	value[b]	*p*-value	R^2	value[b]	*p*-value[c]	R^2
E_{0-0}^{A} **(A form)** acetonitrile[a]	ρ	4.68	0.0025	0.92	5.17	0.0012	0.94	1.35	–	1.00
	s	–1.74	0.2835		–2.63	0.1114		–1.82	–	
	$\Delta E_{0-0,H}$	25.50	2.03×10^{-6}		24.73	1.64×10^{-6}		22.03	–	
E_{0-0}^{B} **(B form)** acetonitrile[a]	ρ	2.04	9.93×10^{-6}	0.99	1.77	0.0002	0.98	1.99	–	1.00
	s	–0.67	0.0112		–0.81	0.0488		0.28	–	
	$\Delta E_{0-0,H}$	20.56	6.15×10^{-10}		19.45	1.08×10^{-8}		17.18	–	
E_{0-0}^{A} **(A form)** water	ρ	2.52	5.51×10^{-4}	0.93	3.02	8.54×10^{-6}	0.99	3.11	0.2924	0.94
	s	–1.43	0.0833		–1.30	0.0120		–0.59	0.8679	
	$\Delta E_{0-0,H}$	25.01	3.76×10^{-9}		23.55	1.71×10^{-10}		21.06	0.0153	
E_{0-0}^{B} **(B form)** water	ρ	2.01	0.0001	0.96	1.99	0.0002	0.95	0.09	0.0672	0.99
	s	–1.00	0.0457		–1.00	0.0579		–2.05	0.0923	
	$\Delta E_{0-0,H}$	21.40	4.98×10^{-10}		20.21	9.42×10^{-10}		17.84	0.0019	
E_{0-0}^{A} **(A form)** water[d]	ρ	3.15	8.34×10^{-5}	0.99	3.14	0.0002	0.99	2.42	–	1.00
	s	–0.66	0.1162		–1.16	0.0470		–1.64	–	
	p	–0.60	0.0092		–0.11	0.5251		–0.87	–	
	$\Delta E_{0-0,H}$	24.85	4.42×10^{-9}		23.52	1.25×10^{-8}		20.99	–	

E_{0-0}^{B} **(B form)** water[d]	ρ	1.81	0.0017	0.97	1.78	0.0025	0.97	0.17	–	1.00
	s	–1.25	0.0400		–1.26	0.0489		–1.94	–	
	p	0.19	0.2949		0.20	0.3030		0.09	–	
	$\Delta E_{0-0,H}$	21.45	1.98×10^{-8}		20.26	3.35×10^{-8}		17.85	–	

[a] The R_P substituent –OH is removed for this multivariable regression due to its H-bonding interaction with the solvent acetonitrile (see Figure 2c right panels for visualization of the linear fit with respect to the newly defined parameter $\sigma_o^{\mathrm{I}} = \sigma_p + \frac{s}{\rho}V$. The inclusion of this specific –OH substituent worsens the linearity of the overall fit. The magnitudes of the retrieved ρ and s coefficients (see Equation 1 in main text), weighted according to the numerical span of the corresponding substituent parameters (see Table S5), were used to compute the contribution percentages of σ_p (electronic) and V (steric effects), respectively, to the energy term. These contributions sum to 100% in each case.

[b] In the unit of 10^3 cm^{-1}.

[c] The p-value is not available for the $–COCH_3$ series due to the limited number of data points (three data points against two variables in acetonitrile after removal of the R_P substituent –OH; four data points against three variables in water) for both **A** and **B** forms.

[d] The linear regression for the 0-0 transition energy gaps of **A** and **B** forms in water was performed with three variables, one more than the two rows above that involve two variables. Overall the small p coefficients indicate that the intermolecular contributions to the 0-0 transition energy gaps are minor (the corresponding fits are thus not shown).

Table S7. Multivariable linear regression for pK_a, ΔpK_a, and pK_a^* of the unlocked *p*-HBDI derivatives in water.

Property	Coeff.	$-CH_3$			$-CF_3$			$-COCH_3$		
		value	*p*-value	R^2	value	*p*-value	R^2	value	*p*-value [a]	R^2
pK_a [b]	ρ	–1.78	0.0109	0.81	–1.57	0.0067	0.84	–2.81	0.2226	0.88
	s	–1.70	0.1266		–1.31	0.1309		–4.09	0.2716	
	$pK_{a, H}$	7.98	5.92×10^{-6}		7.06	3.19×10^{-6}		7.25	0.0296	
pK_a [b]	ρ	–2.55	0.0032	0.94	–2.17	0.0024	0.94	–2.35	–	1.00
	s	–2.65	0.0190		–2.05	0.0201		–3.39	–	
	p	0.73	0.0498		0.57	0.0515		0.58	–	
	$pK_{a, H}$	8.17	7.20×10^{-6}		7.22	4.56×10^{-6}		7.30	–	
ΔpK_a [c]	ρ	1.63	0.0030	0.95	4.49	0.0022	0.93	9.24	–	1.00
	s	1.89	0.0123		2.56	0.0804		6.62	–	
	p	–1.39	0.0011		–2.23	0.0059		–1.86	–	
	$\Delta pK_{a, H}$	12.05	2.36×10^{-7}		11.56	1.12×10^{-5}		12.61	–	
pK_a^* [c]	ρ	–4.18	0.0001	0.98	–6.66	0.0010	0.95	–11.59	–	1.00
	s	–4.54	0.0008		–4.61	0.0249		–10.00	–	
	p	2.12	0.0004		2.80	0.0049		2.43	–	
	$pK_{a, H}^*$	–3.88	3.66×10^{-5}		–4.35	0.0011		–5.31	–	
pK_a^* [d]	ρ	–5.35	0.0013	0.94	–5.03	0.0011	0.96	–7.08	–	1.00
	s	–3.88	0.0284		–2.26	0.0930		–4.01	–	
	p	2.38	0.0056		1.22	0.0353		2.59	–	

	$pK_{a,\,H}^{*}$	1.05	0.0795		0.39	0.3800		0.72	–	

[a] The p-value is not available for the –$COCH_3$ series when all three variables (electronic, steric, and hydrophobicity) are included due to the limited number of data points for the fits.

[b] The two-variable linear regression for pK_a is based on Equation 2 in main text without the hydrophobicity term. The three-variable linear regression for pK_a is based on Equation 2 in main text with the hydrophobicity term included.

[c] Calculated with the revised transition energy ($E_{FC}^{A} + \Delta E_{0-0}$), where $\Delta E_{0-0} = E_{0-0}^{A} - E_{0-0}^{B}$ and E_{FC}^{A} is the Franck-Condon relaxation energy (i.e., excess energy above the zero-point energy of S_1 state upon photoexcitation, see the schematic in Figure 3b). This key energy term, which was omitted, allows for a more accurate estimate of ESPT driving force for the prediction of ESPT occurrence/rate (see Section S5.2).

[d] Calculated with the conventional transition energy $\Delta E_{0-0} = E_{0-0}^{A} - E_{0-0}^{B}$. Note that the Franck-Condon relaxation energy term was not included in this conventional treatment that has been widely adopted in the field (see Section S5.2 for details).

Table S8. Multivariable linear regression for pK_a, ΔpK_a, and pK_a^* of the locked photoacids[a] in water.

Property	Coeff.	value	*p*-value	R^2
pK_a	ρ	–3.54	0.0018	1.00
	s	–6.22	0.0033	
	p	3.56	0.0044	
	$\Delta pK_{a,H}$	8.20	0.0003	
ΔpK_a[b]	ρ	4.63	0.0454	0.99
	s	0.06	0.9650	
	p	–1.50	0.3226	
	$\Delta pK_{a,H}$	5.59	0.0160	
pK_a^*[b]	ρ	–8.16	0.0250	0.99
	s	–6.28	0.1058	
	p	5.06	0.1009	
	$pK_{a,H}^*$	2.61	0.0332	

[a] The chemical structures of the five singly substituted (including –H, –F, –Cl, –Br, $-NMe_2$ as the R_P substituent) locked photoacids are shown in Figure 4a or Figure S21a. They are termed as L0F, L1F, L1Cl, L1Br, and $L1NMe_2$, respectively, with the acidity values listed in Table S9 below.

[b] Calculated with the transition energy $\Delta E_{0-0} = E_{0-0}^{\mathrm{A}} - E_{0-0}^{\mathrm{B}}$, which is the conventionally used free energy difference (see Table S9).

S7. ESPT kinetics and Bell-Evans-Polanyi principle

S7.1 Spectroscopic measurement by transient absorption

We used femtosecond transient absorption (fs-TA) spectroscopy in this work to measure the ESPT dynamics for various photoacids in water. Our homebuilt fs-TA setup is based on the same 1-kHz repetition rate laser source for FSRS (see Section S3.1). The 400 nm pump pulse (for photoexcitation) is generated by second harmonic generation (SHG) of the 800 nm fundamental pulse, which is then compressed by a prism pair (06SB10, transmission range of 185 nm to 2.1 μm, Newport, Inc.). The probe pulse is shared with FSRS which is supercontinuum white light (SCWL) generated by focusing a small portion of the fundamental pulse onto a 2-mm-pathlength quartz cuvette filled with deionized water, followed by temporal compression using a chirped mirror pair DCM-12, 400−700 nm, and DCM-9, 450−950 nm (Laser Quantum, Inc.) for photoacids of L*n*F series and **7a** (R_P = –NH_2 and R_I = –CH_3, or *m*-NH_2-*p*-HBDI), respectively. The cross-correlation time for these measurements was estimated to be ~100 fs. For L*n*F series of photoacids in H_2O and D_2O, polarizations of the pump and probe beams were set at the magic angle (54.7°) configuration to eliminate anisotropic contributions.[40,50] For the photoacid *m*-NH_2-*p*-HBDI, we used parallel polarizations for the two incident beams because of the ultrashort ESPT time of the photoacid (~120 fs) and hence the dynamics would not be affected by the rotational diffusion/anisotropy dynamics, which typically occur on the tens-to-hundreds of picoseconds (ps) timescales for *p*-HBDI derivatives of similar sizes.[2,17] The detection time window for these fs-TA measurements was typically from –2 to 900 ps with customized increments except for L0F in pD = 3 solution, whose maximal temporal delay was set to 3.6 nanosecond (ns) using a quadruple-pathlength delay stage to capture the slow ESPT dynamics. The probe pulse dispersion for the 3.6 ns experiment was not corrected. The sample concentrations of L*n*F series were best achieved in the OD range of 0.1–0.3 per mm due to their very low solubility in water, and higher-than-usual pump powers of 0.4–0.5 mW were used to compensate for the signal intensity due to low sample concentrations. The protonated neutral photoacid *m*-NH_2-*p*-HBDI has slightly better solubility in water and thereby a decent OD of 0.3–0.5 per mm and a pump power of 0.2–0.3 mW were used.

S7.2 Global analysis and ESPT dynamics

The ESPT rate constants were obtained from fs-TA spectra by global analysis using Glotaran.[51] Under the sequential and parallel kinetic schemes, evolution-associated difference spectra (EADS)

and decay-associated difference spectra (DADS) were retrieved with their characteristic lifetimes. The L*n*F series of photoacids are best described by three components (Figures S22 and S23). The first EADS (black trace) shows a blue SE band between ca. 450–500 nm and was assigned to the protonated form (A^*). The associated lifetimes of 580–970 fs that match the solvent reorganization time of water[52,53] and insensitivity to deuteration (670–1.4 ps) suggest that A^* undergoes solvation, presumably re-establishing the H-bonding network, prior to ESPT. In particular, the apparent intermediate form with 0.58–1.4 ps lifetimes by global analysis (Figure S22) likely results from the so-called "contact-ion-pair (CIP)" mixed with solvent reorganization, which sets up proper H-bonding between the photoacid and solvent for ESPT because the dipole moment of the photoacid changes in the excited state due to photoexcitation. This is why the time constant matches typical solvation (longitudinal relaxation) time of water.[52] We have previously studied this problem in a series of works[2,17] and it has also been extensively reported in literature.[54] This two-step ESPT dynamics is known as the Eigen-Weller model (i.e., CIP formation, followed by the diffusion-controlled proton dissociation) which has well described many photoacid systems and seems to be a good approximation for our current systems as well. Both processes are driven by the intrinsic photoacidity computed from the 0-0 transition energies (Section S4.2 and Table S4), which has been traditionally used and is corroborated by the linear relationships in Figure 4c (main text).

The second EADS (red trace) exhibits a red-shifted SE band due to the energy state stabilization by solvation and the kinetic isotopic effect (~1.6–2.2) confirms its assignment to an intermediate state that undergoes an actual ESPT step, which has been the experimental validation (Table S9). This intermediate may be A^*- or B^*-like (see Figure S22), likely depending on the chromophore photoacidity; this is because the SE peak position gets closer to the B^* SE peak (blue EADS) as the photoacidity increases from L0F to L3F (see top to bottom panels in Figures S22 and S23 middle columns). The last EADS (blue trace) showing a consistent SE peak wavelength with B^* fluorescence peak is assigned to B^*, which decays on the ns timescales along with the high FQY. Steady-state electronic spectra of several reported photoacids are presented (Figure S26) for more insightful comparisons with the GFP-family of photoacidic chromophores.

The unlocked photoacid *m*-NH_2-*p*-HBDI (compound **7a**, see Table S1 and Figure 4c) has drastically different dynamics from the locked photoacid series. First, the ESPT reaction of **7a** is close to being barrierless (black to red EADS with a lifetime of 120 fs, Figure S25b bottom panels) and the solvation step as commonly considered essential for superphotoacids is absent. Notably,

the lifetime of A* retrieved by global analysis which characterizes the overall k_{nr} (equal to $k_{\mathrm{ESPT}} + k_{\mathrm{IC}}$ where k_{IC} is the rate constant of other nonradiative internal conversion pathways) is used to approximate the ESPT time constant considering that k_{ESPT} must be comparable (without the order-of-magnitude difference) to k_{IC} for observing the ESPT occurrence (Figure S15). Such an approximation is sufficient for the systematic linear regression analysis for several series of GFP chromophore derivatives in this work. Using Huppert's classifications of photoacids by the ESPT rate, **7a** belongs to regime IV defining photoacids with $\mathrm{p}K_\mathrm{a}^* < -7$.[54] However, the calculated $\mathrm{p}K_\mathrm{a}^*$ of **7a** using various methods (Table S4) are all significantly higher than –7, and only by using Stokes shift (as discussed in Section S5.2) can a $\mathrm{p}K_\mathrm{a}^*$ lower than –7 be yielded. This contradiction poses a new challenge to previous ESPT theories that were formulated only on the basis of limited samplings of photoacids with an underestimated driving force. Second, due to the tiny FQY, the dynamics following ESPT can be readily assigned to isomerization of B^* featuring the formation of an intermediate state[40,55] (blue EADS with a red-shifted SE band and an increased excited-state absorption band at shorter wavelengths) and a hot ground state (green EADS) with a peak wavelength typically to the red side of the original ground state absorption peak.[3,22,40] Furthermore, a mixing of the fluorescent and nonfluorescent mechanisms would occur when ESPT rate is similar or comparable to Franck-Condon relaxation rate, which is typically on hundreds of femtoseconds timescale (sub-ps to ps), while other nonradiative decay should not kinetically outcompete ESPT.

S7.3 Previous theories *versus* Bell-Evans-Polanyi principle: Implications on ESPT

Despite the extensive study for decades, there are still no analytical theories to date that can explain all aspects of ESPT. One relatively thorough ESPT model was proposed by Huppert and coworkers who applied Hynes's ground-state proton transfer theory and Marcus' semi-empirical bond energy bond order (BEBO) theory to describe the ESPT rate and its correlations to the thermodynamic driving force $\mathrm{p}K_\mathrm{a}^*$.[54,56] The photoacids are thus categorized into different regimes by the magnitudes of $\mathrm{p}K_\mathrm{a}^*$ and ESPT rate. Photoacids in Regime I are capable of ESPT to water while those in Regime II can transfer a proton to water and alcohols. The ESPT rate for both regimes can be described by nonadiabatic proton transfer theory originally proposed by Hynes. Huppert defined the $\mathrm{p}K_\mathrm{a}^*$ for photoacids in Regimes I and II to be ~0 and negative with up to a few units below zero, respectively. Regime III characterizes photoacids with $-6 < \mathrm{p}K_\mathrm{a}^* < -4$ whose ESPT rate is subject to solvent reorientation time. The adiabatic proton transfer rate can be adopted to

describe the kinetic behavior for photoacids in this regime. The last Regime IV describes superphotoacids with pK_a^* < –7 which are believed to reach the rate limit of ESPT around 100 fs. This limit has been explained by Huppert using the empirical BEBO theory in which the solvent orientational motions are not needed any more when the driving force is large enough (essentially barrierless). Due to the extremely fast ESPT rate and low pK_a^*, very few reported photoacids fall into this category and QCy9 has been considered as the strongest photoacid to date.[57-61] Huppert's comprehensive insights of photoacid strengths and thermodynamics-kinetics relationships well account for the ESPT behaviors for most reported photoacids. However, some recent experimental studies have posed considerable challenges. For example, Meech et al. studied the difluorinated *p*-HBDI (denoted as 2F in this work) by time-resolved fluorescence up-conversion method with a sub-50 fs time resolution and showed that the ESPT of 2F in water is ultrafast with a time constant of ~50 fs.[62] The pK_a^* of 2F is calculated to be –4 by Förster equation. According to Huppert's classification, 2F belongs to Regime III at most and its ESPT rate should be limited by solvation, which, however, contradicts the experimentally observed ~50 fs ESPT time.

In this work, we did not directly implement Hynes' proton transfer theory or Marcus' BEBO proton transfer theory to correlate pK_a^* to ESPT rate constant (k_{ESPT}) for the LnF series of photoacids. This is because these two theories yield nonlinear relationships between pK_a^* and the logarithm of ESPT rate constant, which directly contradicts our experimental observation for these fluorescent photoacids (Figure 4c). In Hynes' proton transfer theory, the reaction barrier $\Delta G^{\#}$ is given by the same expression as nonadiabatic electron transfer:

$$\Delta G^{\#} = \left(1 + \frac{\Delta G_0}{4\Delta G_0^{\#}}\right)^2 \Delta G_0^{\#} \quad \text{(S9)}$$

where $\Delta G_0^{\#}$ is the intrinsic barrier for a symmetric proton transfer when the driving force, the free energy change ΔG_0, is zero. With p$K_a^* = \frac{\Delta G_0}{RT\ln 10}$ and $\log(k_{\mathrm{ESPT}}) \sim -\Delta G^{\#}$, one can obtain a quadratic relationship:

$$\log(k_{\mathrm{ESPT}}) \sim -\left(1 + \frac{RT\ln 10}{4\Delta G_0^{\#}} \mathrm{p}K_a^*\right)^2 \Delta G_0^{\#} \quad \text{(S10)}$$

In Marcus' BEBO theory, the reaction barrier is given by:

$$\Delta G^{\#} = \frac{\Delta G_0}{2} + \Delta G_0^{\#} + \frac{\Delta G_0^{\#}}{\ln 2} \ln\left(\cosh\left(\frac{\Delta G_0 \ln 2}{2\Delta G_0^{\#}}\right)\right) \quad \text{(S11)}$$

The thermodynamics-kinetics relationship will then follow:

$$\log(k_{\mathrm{ESPT}}) \sim -\frac{RT\ln 10}{2}\mathrm{p}K_{\mathrm{a}}^{*} - \frac{\Delta G_0^{\#}}{\ln 2}\ln\left(\cosh\left(\frac{RT\ln 2 \cdot \ln 10}{2\Delta G_0^{\#}}\mathrm{p}K_{\mathrm{a}}^{*}\right)\right) \quad \text{(S12)}$$

The LnF series of photoacids demonstrate a linear numerical relationship between $\log(k_{\mathrm{ESPT}})$ and pK_a^* (see data plots in Figure 4c, Class 1) and thus better fit the Bell-Evans-Polanyi (BEP) principle:

$$E_{\mathrm{a}} = E_0 + \alpha \cdot \Delta H \quad \text{(S13)}$$

$$\log(k_{\mathrm{ESPT}}) = \log(k_0) - \alpha \cdot \mathrm{p}K_{\mathrm{a}}^{*} \quad \text{(S14)}$$

The E_0 here is the same as $\Delta G_0^{\#}$ that denotes the intrinsic barrier for the proton transfer reaction when the driving force (we approximate the reaction enthalpy ΔH as the Gibbs free energy ΔG_0 assuming the minimal contribution of entropy) is zero. It is important to note that all three models for ESPT are best discussed within the same family of photoacids whose structures do not drastically differ (e.g., the GFP-chromophore-derived LnF series of fluorescent photoacids, or the unlocked p-HBDI derivatives as essentially nonfluorescent photoacids). As such, $\Delta G_0^{\#}$ or E_0 and α can be held constant in the modeling of thermodynamics-kinetics relationships using Equations S10, S12, and S14. This prerequisite is valid as seen by the conspicuous deviation of other photoacids (i.e., QCy9, 2F, NH_2, and other dim compounds; **Class 2**) from the linear trend of LnF series/L1Cl/L1Br of photoacids (**Class 1**) in the same GFP chromophore family (Figure 4c in main text). Nevertheless, BEP principle seems to govern ESPT for both nonfluorescent and fluorescent photoacids despite their class-specific slopes and intercepts in the experimental linear fits.

Importantly, the better match with BEP principle rings an alarm bell about the aforementioned ESPT knowledge translated from Hynes' and Marcus' proton transfer theories, which ideally apply to ground-state proton transfer reactions but exhibit clear limitations in characterizing/predicting ESPT properties (i.e., in the electronic excited state). This important outcome is somewhat expected because BEP is a generalized principle and has demonstrated successes in various types of chemical reactions beyond proton transfer.[63-65] The other two theories, however, have not been fully validated by extensive experimental proton transfer works besides Huppert's initial efforts.[54,56] In fact, the recent combined experimental and computational reports on ESPT reaction have shown that, for the same family of photoacids or excited-state intramolecular proton transfer (ESIPT)-capable molecules, the thermodynamic driving force (ΔG, computed) and kinetics (experimental) mainly follow the BEP principle.[66,67]

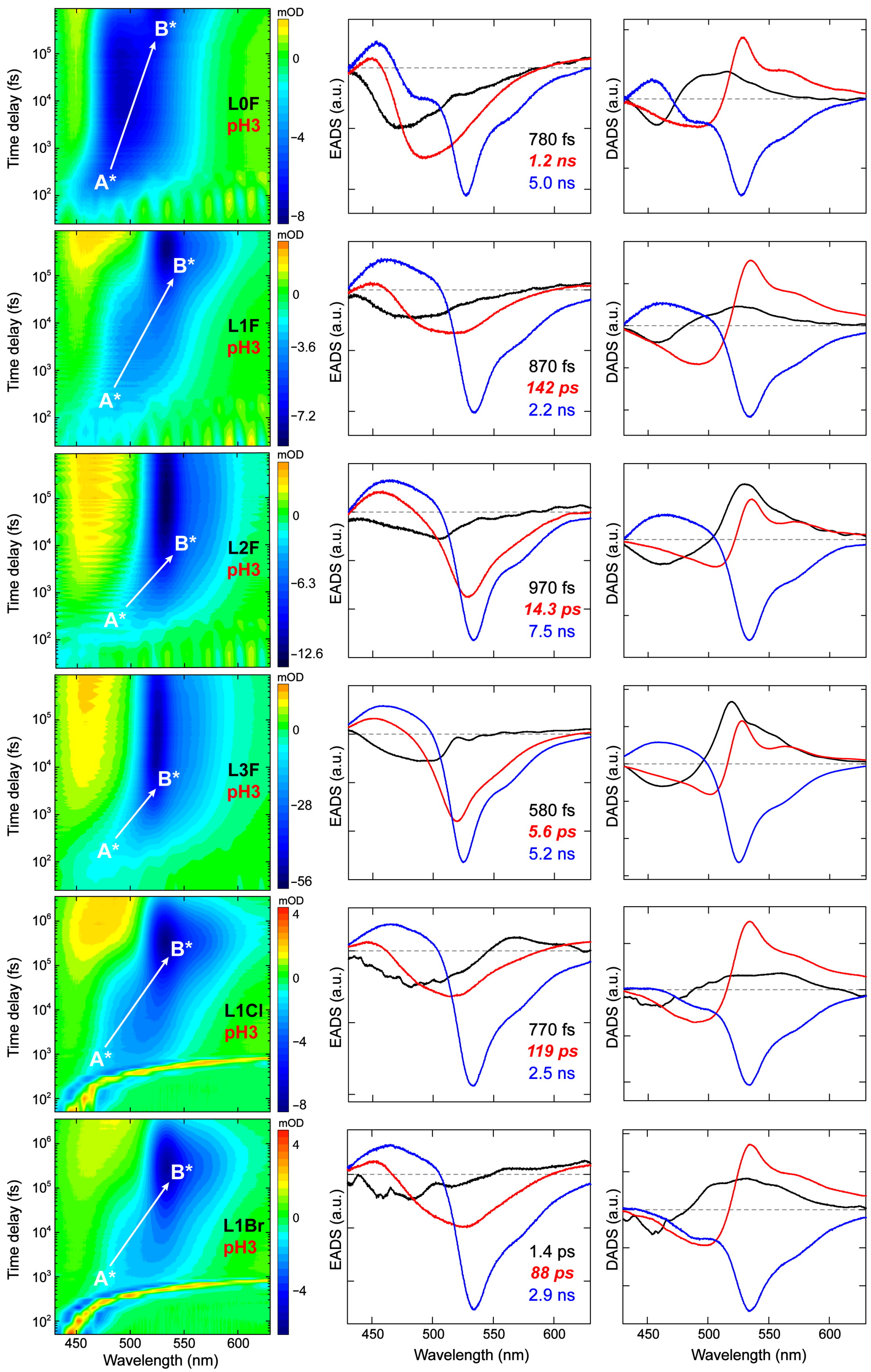

L0F
pH3
780 fs
1.2 ns
5.0 ns
L1F
pH3
870 fs
142 ps
2.2 ns
L2F
pH3
970 fs
14.3 ps
7.5 ns
L3F
pH3
580 fs
5.6 ps
5.2 ns
L1Cl
pH3
770 fs
119 ps
2.5 ns
L1Br
pH3
1.4 ps
88 ps
2.9 ns
Time delay (fs)
Wavelength (nm)
EADS (a.u.)
DADS (a.u.)
mOD

Figure S22. Fs-TA spectra and global analysis for the fluorescent (locked) photoacids in pH 3 aqueous solutions (H_2O). All sample solutions were excited at 400 nm. (Left column) The ESPT process is illustrated by the SE band shift from the protonated (A*) to deprotonated (B*) forms in the excited state with a white tilted arrow. Global analysis (right two columns) yields three EADS/DADS corresponding to the solvation (black), ESPT (red), and B* decay (blue), respectively, of the photoexcited chromophores L0F, L1F, L2F, L3F, L1Cl, and L1Br (from top to bottom panels). The retrieved best-fit lifetimes are color-coded and listed in the insets of middle panels (see Section S7.2 for more details and the necessary/reasonable approximations). Zero ΔOD is depicted by the gray dashed line.

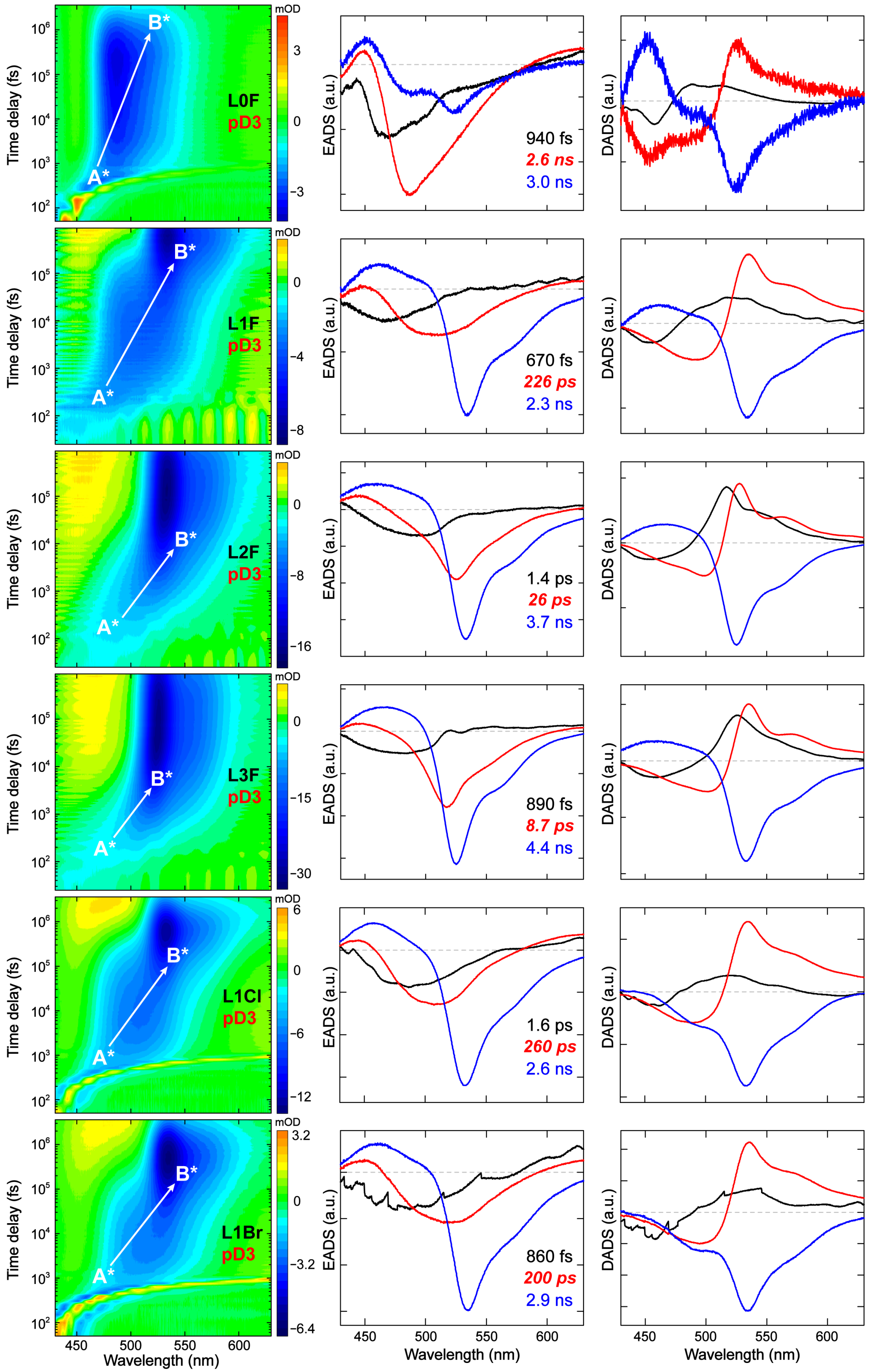
L0F
pD3
940 fs
2.6 ns
3.0 ns
L1F
pD3
670 fs
226 ps
2.3 ns
L2F
pD3
1.4 ps
26 ps
3.7 ns
L3F
pD3
890 fs
8.7 ps
4.4 ns
L1Cl
pD3
1.6 ps
260 ps
2.6 ns
L1Br
pD3
860 fs
200 ps
2.9 ns
A*
B*
mOD
Time delay (fs)
EADS (a.u.)
DADS (a.u.)
Wavelength (nm)

Figure S23. Fs-TA spectra and global analysis for the fluorescent (locked) photoacids in pD 3 aqueous solutions (D_2O). All sample solutions were excited at 400 nm. (Left column) The ESPT process is illustrated by the SE band shift from the protonated (A*) to deprotonated (B*) forms in the excited state with a tilted white arrow. Global analysis (right two columns) yields three EADS/DADS corresponding to the solvation (black), ESPT (red), and B* decay (blue), respectively, of the photoexcited chromophores L0F, L1F, L2F, L3F, L1Cl, and L1Br (from top to bottom panels). The retrieved best-fit lifetimes are color-coded and listed in the insets of middle panels (see Section S7.2 for more details and the necessary/reasonable approximations). Zero ΔOD is depicted by the gray dashed line.

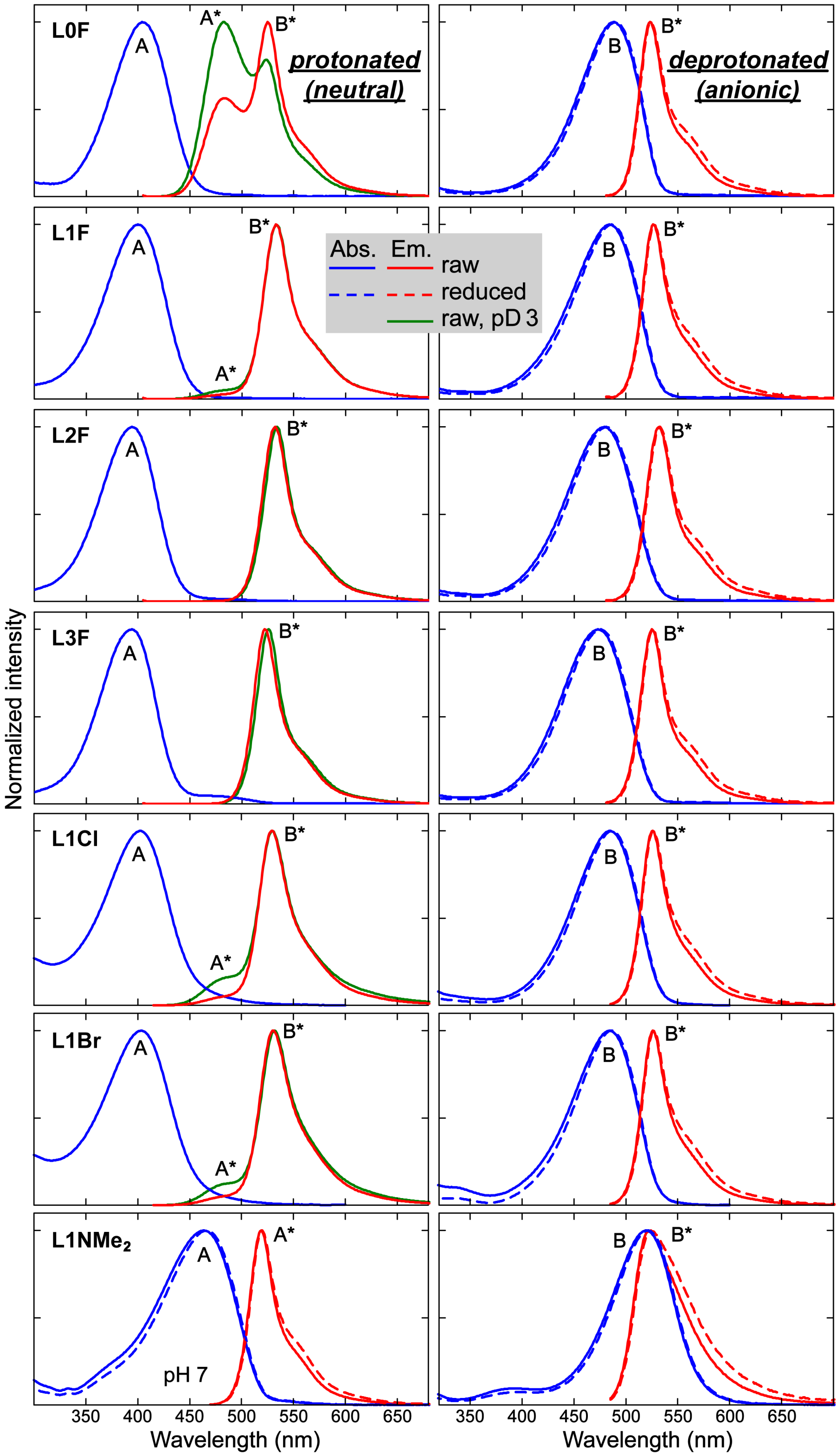

Figure S24. Electronic absorption and emission spectra of the fluorescent (locked) photoacids in H_2O and D_2O. The original absorption (blue) and emission (red) spectra are in solid lines. The

absorption and emission spectra in dashed lines are the reduced spectra to determine 0–0 transition energy gap for the deprotonated/anionic form (see Section S4.2 and Table S9). All the molecules are protonated at pH 3 except L1NMe$_2$ which is protonated at pH 7 (left bottom panel) and deprotonated at pH 11–12 (right bottom panel) in aqueous solution. The emission bands from the protonated and deprotonated forms are denoted as A* and B*, respectively, of the photoexcited chromophores. The emission spectra in pD 3 aqueous solution are shown in green solid lines in the left column.

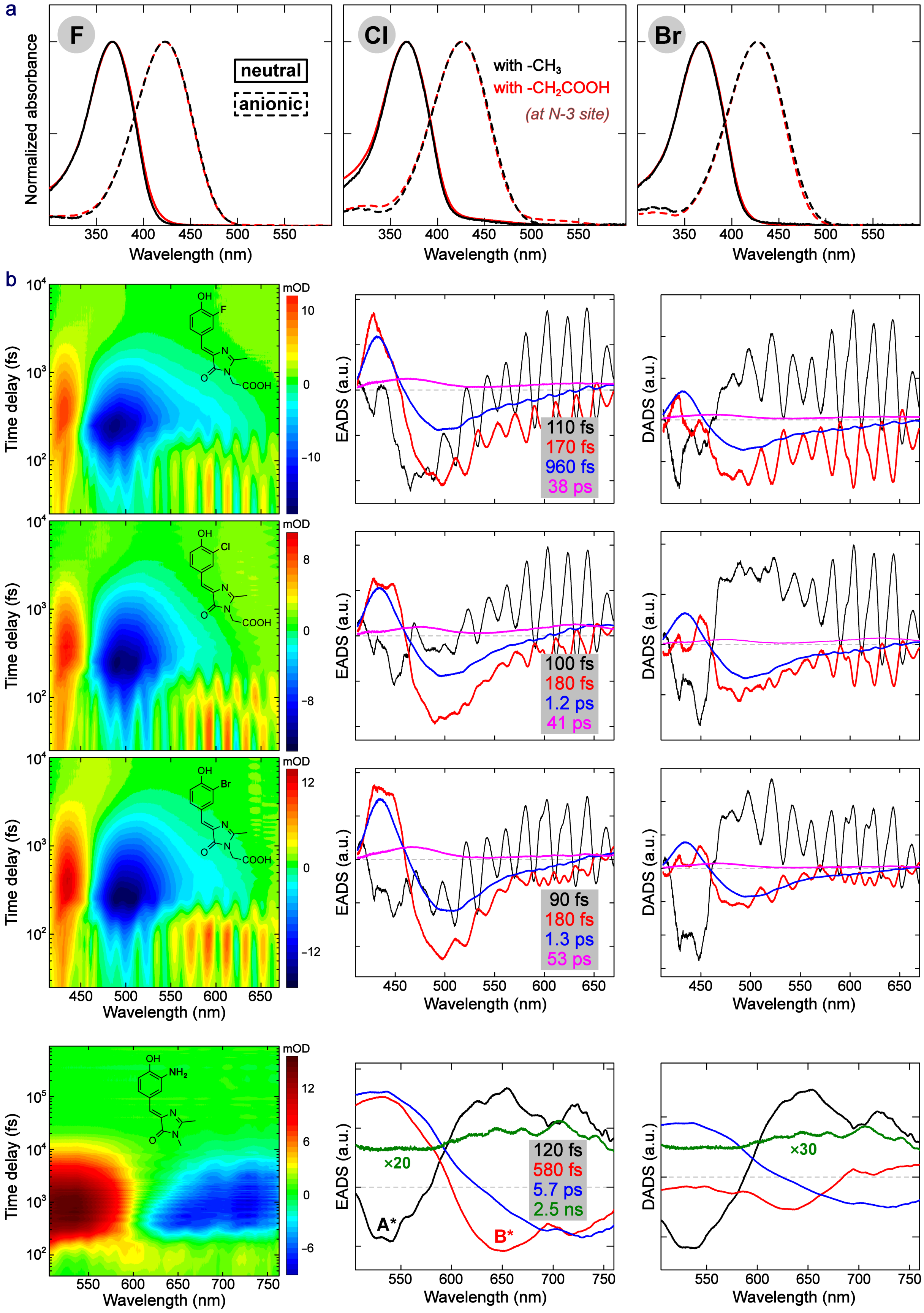
a
F
Cl
Br
neutral
anionic
with -CH3
with -CH2COOH
(at N-3 site)
Normalized absorbance
Wavelength (nm)
b
Time delay (fs)
mOD
EADS (a.u.)
DADS (a.u.)
110 fs
170 fs
960 fs
38 ps
100 fs
180 fs
1.2 ps
41 ps
90 fs
180 fs
1.3 ps
53 ps
120 fs
580 fs
5.7 ps
2.5 ns
×20
×30
A*
B*

Figure S25. Fs-TA spectra and global analysis for the nonfluorescent (unlocked) photoacids in aqueous solution. (a) Normalized electronic absorption spectra of the neutral (solid) and anionic (dashed lines) halogenated (R_P = –F, –Cl, –Br) unlocked photoacids in $–CH_3$ series without (black) and with (red) $–CH_2COOH$ group added to the I-ring's N-3 site that is adjacent to the I-ring C=O group. (b) 2D-contour plots of fs-TA spectra with the photoacid chemical structures shown in the insets of left column. The vertical axis (time delay in fs unit) is displayed in the logarithmic scale. (Middle/right columns) Global analysis yields four EADS/DADS corresponding to the ESPT (black), B* (red), intermediate state (blue), and hot ground state (green, multiplied as shown for easy visualization), respectively, of the photoexcited chromophores (see Section S7.2 for more details and the necessary/reasonable approximations). Note that the bottom panels present data on the compound **7a** without the $–CH_2COOH$ group addition. A* and B* represent the protonated and deprotonated chromophores after photoexcitation. Zero ΔOD is depicted by the gray dashed line. The samples were all excited at 400 nm.

Notably, there is an even faster component within the instrument response time of our optical setup (~90 fs) which mainly fits the coherent artefact, so we did not show this component (i.e., with <90 fs lifetime) in Figure S25b (see above) to better present the unlocked chromophore dynamics that are important. The coherent artefact has been mainly taken care of by this very first component (within the instrument response time) and does not corrupt the later A* components shown above for the protonated chromophore in the excited state. We obtained very good fitting quality of global analysis at multiple probe wavelengths with significant intensities across the detection spectral window, which justifies the accuracy and reliability in both the retrieved time constants and spectral profiles. Importantly, the coherent artefact-caused intensity variation for the A* EADS (see black traces in Figure S25b right panels above) is mainly located in the redder-wavelength region above ~550 nm, which has much less interference with the more intense SE and blue ESA bands (below 550 nm) that are more characteristic of the A* and B* species through the ESPT reaction. In particular, the SE at ~500 nm in fs-TA spectra is not A* but B* because the wavelength matches the steady-state B* fluorescence at ~500 nm (Figure S12). A* fluorescence peak is at ~450 nm, which matches the blue SE peak at ~450 nm in the first EADS that we assigned to A*. The wavelengths of unlocked and locked molecules cannot be directly compared because the BF_2-locking redshifts the absorption and emission wavelengths (Figure S12 vs. Figure S24).

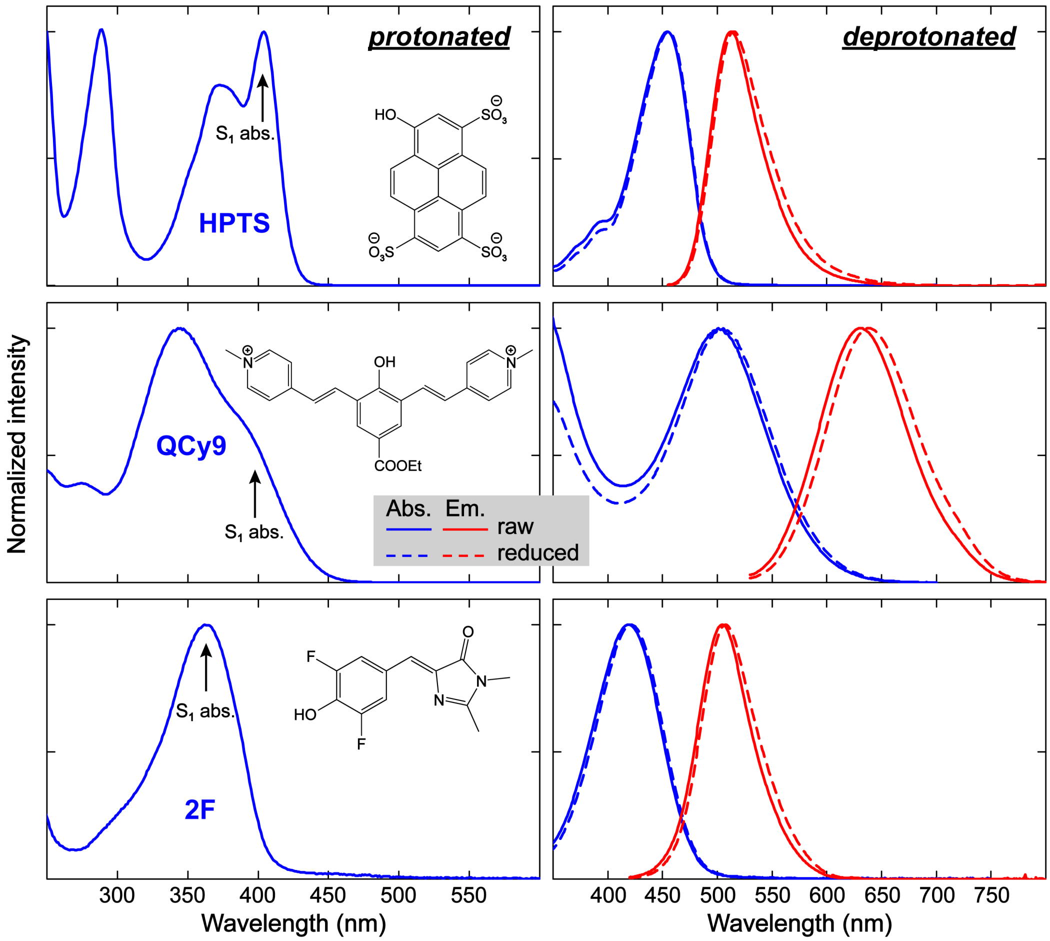

Figure S26. Electronic absorption and emission spectra of some other reported photoacids. (Left column) The structures of these reported photoacids are shown as insets. The original absorption (blue) and emission (red) spectra are displayed in solid lines. (Right column) The absorption and emission spectra in the color-coded dashed lines are reduced spectra to determine the 0-0 transition gap for the deprotonated chromophore form (see Section S4.2). The first singlet excited state (S_1) of the protonated chromophores (top, HPTS; middle, QCy9; bottom, 2F) is indicated by the arrow in each panel of left column.

Considering the typical ns/ps timescale of radiative/nonradiative decay (Section S7.2), a crude estimate of the FQY threshold for regular organic chromophores is on the order of ~10^{-3}. Note that the unlocked photoacids of this work have FQYs on the order of ~10^{-4} or lower for the protonated form (see main text). If a medium FQY is considered on the order of 10^{-2} to 10^{-1}, our models would predict a conventional ESPT scheme with the driving force determined by the energy difference of two energy minima of the acid and conjugate base in the excited state (Figure 3b).

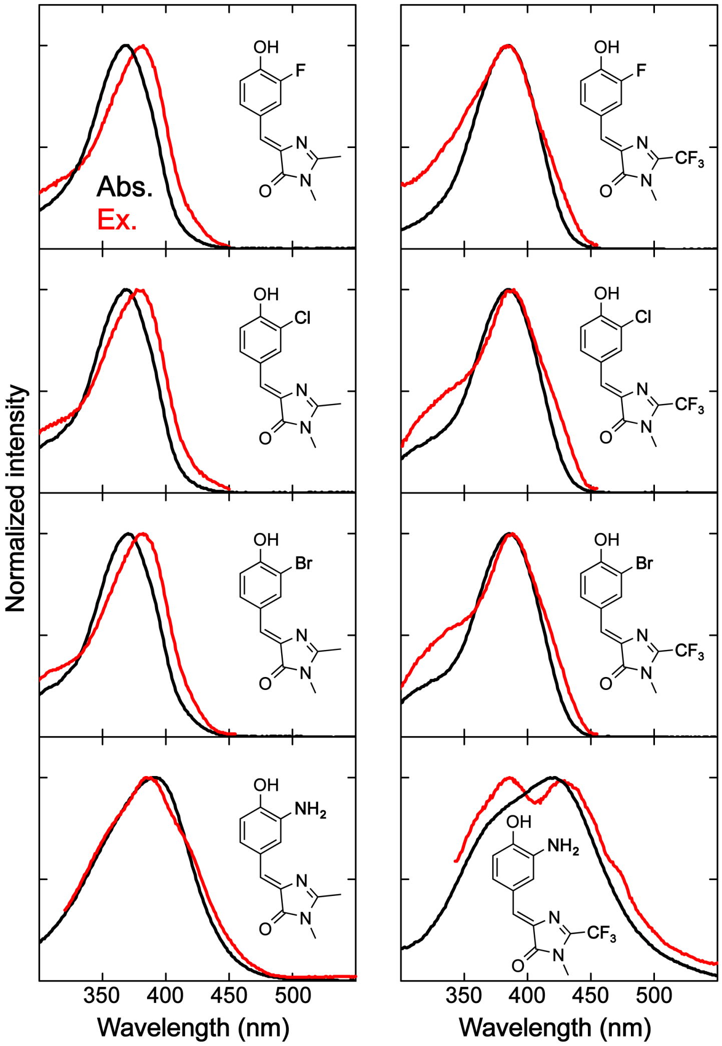

Figure S27. Comparison of the electronic absorption and excitation spectra of the nonfluorescent photoacids in methanol. The structures of the (left column) –CH_3 series and (right column) –CF_3 series of photoacids are shown as insets. The absorption (black) and excitation (red) spectra are normalized for a better profile comparison. The difference between the normalized absorption and excitation spectral profiles suggests that the red-absorbing region is in general more fluorescent (i.e., featuring a slower nonradiative decay rate by ring twists or internal conversion from the excited state) than the blue-absorbing region. Note that ESPT is inhibited in methanol, hence the main internal conversion pathway from the excited state "cleanly" involves the ring twists of these unlocked nonfluorescent photoacids (in their protonated form) derived from the GFP chromophore.

Notably, a bluer (higher-energy) light may lead to more ring twists while accelerate ESPT for unlocked compounds. The exact ESPT rate is thus challenging to determine due to this interplay.

Table S9. Acidities and kinetics of the locked fluorescent photoacids.

Comp.	protonated (A)			deprotonated (B)			pK_a [a]	ΔpK_a [b]	pK_a^* [b]	τ_{ESPT} / ps [c] (pH 3/pD 3)
	λ_{abs} / nm	λ_{em} / nm	E^{A}_{0-0} / cm^{-1}	λ_{abs} / nm	λ_{em} / nm	E^{B}_{0-0} / cm^{-1}				
L0F	404	483	22153	488	523	19474	8.2	5.6 (11.1)	2.6 (–2.9)	1200/2600
L1F	400	480	22153 [e]	484	526	19436	6.8	5.7 (11.7)	1.1 (–4.9)	142/226
L2F	394	– [d]	22153 [e]	479	532	19380	5.4	5.8 (12.6)	–0.4 (–7.2)	14.3/26
L3F	393.5	– [d]	22153 [e]	473	525	19608	4.6	5.3 (12.2)	–0.7 (–7.6)	5.6/8.7
L1Cl	402.5	483	22072	485	526	19445	6.5	5.5	1.0	119/260
L1Br	403	479	22059	484	526	19445	6.4	5.5	0.9	88/200
L1NMe$_2$	464	519	19836	520	523	19109	9.1	1.5	7.6	–/– [f]

[a] The pK_a values of L0F and L1NMe$_2$ were reported,[7] while those of L1F/L2F/L3F/L1Cl/L1Br were measured systematically and presented in Figure S19 above.

[b] Calculated with conventional transition energy ΔE_{0-0} (i.e., $E^{A}_{0-0} - E^{B}_{0-0}$). The values calculated with the revised transition energy of $E^{A}_{FC} + \Delta E_{0-0}$ are listed in the parentheses, which yield much smaller pK_a^* values that contradict the observed ESPT kinetics for these locked (fluorescent) photoacids with rather long ESPT time constants (see the retrieved values in Figures S22 and S23, tabulated in the last column herein, as well as main text for discussions). Note that $pK_a^* = pK_a - \Delta pK_a$ (by definition) as shown in Section S5.2 above.

[c] There is a clear correlation between pK_a^* and τ_{ESPT}: the lower the pK_a^*, the smaller the τ_{ESPT} (i.e., a faster ESPT reaction).[22,41,54,67]

[d] Not available due to the efficient ESPT reaction in water. See Figure S24 above for the experimental steady-state electronic spectra.

[e] The 0-0 transition energy for L1F/L2F/L3F is approximated as that of L0F due to their similarities.[7,41]

[f] ESPT does not occur for this compound in water,[7] consistent with its high pK_a^*.

Table S10. Acidities and kinetics of several nonfluorescent and reported photoacids in aqueous solution.

Photoacid	protonated	deprotonated			pK_a	ΔpK_a	pK_a^*	τ_{ESPT} / ps
	λ_{abs} / nm	λ_{abs} / nm	λ_{em} / nm	E^{B}_{0-0} / cm^{-1}				
F [a]	367	423	497.5	21381	6.9	12.3	–5.4	0.11
Cl [a]	367	425.5	498	21254	6.7	12.6	–5.9	0.10
Br [a]	368	427	498	21218	6.6	12.5	–5.9	0.09
NH_2 [a]	386.5	454	650	19425	8.1	13.5	–5.4	0.12
2F [b]	364	419	504	21322	5.7	12.9	–7.2	0.05
QCy9 [b]	388	500	631	17331	4.3	17.7	–13.4	0.04
HPTS [b]	404	455	512	20619	7.5	6.9 [c]	0.6 [c]	90

[a] The chromophore chemical structures can be found in Figure S25b insets. The electronic absorption and emission spectra of F/Cl/Br-substituted chromophores (with $-CH_2COOH$ at the N-3 position) are nearly identical to their counterparts without $-CH_2COOH$ at the N-3 position (Figure S25a). The values are nearly identical to those of the unlocked compounds with $-CH_3$ at the N-3 position (Table S3).

[b] The pK_a values and ESPT time constants for these photoacids were taken from the literature reports.[41,57,61,62,68,69] The corresponding steady-state electronic spectra can be found in Figure S26 above.

[c] The ΔpK_a and pK_a^* values were calculated with the conventional transition energy ΔE_{0-0} (i.e., $E^{A}_{0-0} - E^{B}_{0-0}$) for this highly fluorescent photoacid in **Class 1** (see Figure 4c,d in main text). For the other photoacids in this table which are nonfluorescent, the ΔpK_a and pK_a^* values were calculated with the revised transition energy: $E^{A}_{FC} + \Delta E_{0-0} = E^{A}_{ex} - E^{B}_{0-0}$, where $\Delta E_{0-0} = E^{A}_{0-0} - E^{B}_{0-0}$. The latter two terms, E^{A}_{ex} and E^{B}_{0-0}, can be readily obtained from steady-state electronic spectra for these essentially nonfluorescent photoacids in **Class 2** as demonstrated above.

S8. References

1. Kojima, S. et al. Fluorescent properties of model chromophores of tyrosine-66 substituted mutants of *Aequorea* green fluorescent protein (GEP). *Tetra. Lett.* **39**, 5239-5242 (1998).
2. Chen, C. et al. Unveiling structural motions of a highly fluorescent superphotoacid by locking and fluorinating the GFP chromophore in solution. *J. Phys. Chem. Lett.* **8**, 5921-5928 (2017).
3. Boulanger, S. A. et al. Shedding light on ultrafast ring-twisting pathways of halogenated GFP chromophores from the excited to ground state. *Phys. Chem. Chem. Phys.* **23**, 14636-14648 (2021).
4. Lincke, K. et al. On the absorption of the phenolate chromophore in the green fluorescent protein—role of individual interactions. *Chem. Commun.* **46**, 734-736 (2010).
5. Lee, C.-Y. et al. Facile synthesis of 4-arylidene-5-imidazolinones as synthetic analogs of fluorescent protein chromophore. *Tetrahedron* **68**, 5898-5907 (2012).
6. Chen, C. et al. Designing redder and brighter fluorophores by synergistic tuning of ground and excited states. *Chem. Commun.* **55**, 2537-2540 (2019).
7. Boulanger, S. A. et al. Excited-state dynamics of a meta-dimethylamino locked GFP chromophore as a fluorescence turn-on water sensor. *Photochem. Photobiol.* **98**, 311-324 (2022).
8. Yampolsky, I. V., Balashova, T. A. & Lukyanov, K. A. Synthesis and spectral and chemical properties of the yellow fluorescent protein zFP538 chromophore. *Biochemistry* **48**, 8077-8082 (2009).
9. Chen, C. et al. Developing bright green fluorescent protein (GFP)-like fluorogens for live-cell imaging with nonpolar protein−chromophore interactions. *Chem. Eur. J.* **27**, 8946-8950 (2021).
10. Chen, C. & Fang, C. Devising efficient red-shifting strategies for bioimaging: A generalizable donor-acceptor fluorophore prototype. *Chem. Asian J.* **15**, 1514-1523 (2020).
11. Chen, C., Zhang, H., Zhang, J., Ai, H.-w. & Fang, C. Structural origin and rational development of bright red noncanonical variants of green fluorescent protein. *Phys. Chem. Chem. Phys.* **25**, 15624-15634 (2023).
12. Lin, C.-Y., Romei, M. G., Oltrogge, L. M., Mathews, I. I. & Boxer, S. G. Unified model for photophysical and electro-optical properties of green fluorescent proteins. *J. Am. Chem. Soc.* **141**, 15250-15265 (2019).
13. Romei, M. G., Lin, C.-Y., Mathews, I. I. & Boxer, S. G. Electrostatic control of photoisomerization pathways in proteins. *Science* **367**, 76-79 (2020).

14. Kim, E., Koh, M., Lim, B. J. & Park, S. B. Emission wavelength prediction of a full-color-tunable fluorescent core skeleton, 9-aryl-1,2-dihydropyrrolo[3,4-*b*]indolizin-3-one. *J. Am. Chem. Soc.* **133**, 6642-6649 (2011).
15. Hashimoto, N. et al. Synthesis and photophysical properties of λ5-phosphinines as a tunable fluorophore. *J. Am. Chem. Soc.* **140**, 2046-2049 (2018).
16. Zhu, L., Liu, W. & Fang, C. A versatile femtosecond stimulated Raman spectroscopy setup with tunable pulses in the visible to near infrared. *App. Phys. Lett.* **105**, 041106 (2014).
17. Chen, C. et al. Photoinduced proton transfer of GFP-inspired fluorescent superphotoacids: Principles and design. *J. Phys. Chem. B* **123**, 3804-3821 (2019).
18. Fang, C. & Tang, L. Mapping structural dynamics of proteins with femtosecond stimulated Raman spectroscopy. *Annu. Rev. Phys. Chem.* **71**, 239-265 (2020).
19. Dietze, D. R. & Mathies, R. A. Femtosecond stimulated Raman spectroscopy. *ChemPhysChem* **17**, 1224–1251 (2016).
20. Fang, C., Tang, L., Oscar, B. G. & Chen, C. Capturing structural snapshots during photochemical reactions with ultrafast Raman spectroscopy: From materials transformation to biosensor responses. *J. Phys. Chem. Lett.* **9**, 3253–3263 (2018).
21. Batignani, G., Ferrante, C., Fumero, G., Martinati, M. & Scopigno, T. Femtosecond stimulated Raman spectroscopy. *Nat. Rev. Methods Primers* **4**, 34 (2024).
22. Fang, C., Tang, L. & Chen, C. Unveiling coupled electronic and vibrational motions of chromophores in condensed phases. *J. Chem. Phys.* **151**, 200901 (2019).
23. Frontiera, R. R., Shim, S. & Mathies, R. A. Origin of negative and dispersive features in anti-Stokes and resonance femtosecond stimulated Raman spectroscopy. *J. Chem. Phys.* **129**, 064507 (2008).
24. Umapathy, S., Mallick, B. & Lakshmanna, A. Mode-dependent dispersion in Raman line shapes: Observation and implications from ultrafast Raman loss spectroscopy. *J. Chem. Phys.* **133**, 024505 (2010).
25. Chen, C., Zhu, L.-d. & Fang, C. Femtosecond stimulated Raman line shapes: Dependence on resonance conditions of pump and probe pulses. *Chin. J. Chem. Phys.* **31**, 492-502 (2018).
26. Hart, Stephanie M., Silva, W. R. & Frontiera, R. R. Femtosecond stimulated Raman evidence for charge-transfer character in pentacene singlet fission. *Chem. Sci.* **9**, 1242-1250 (2018).

27. Liu, W. et al. Tracking ultrafast vibrational cooling during excited-state proton transfer reaction with anti-Stokes and Stokes femtosecond stimulated Raman spectroscopy. *J. Phys. Chem. Lett.* **8**, 997-1003 (2017).
28. Tang, L. et al. Excited state structural evolution of a GFP single-site mutant tracked by tunable femtosecond-stimulated Raman spectroscopy. *Molecules* **23**, 2226 (2018).
29. Wang, Z. et al. Mapping the complete photocycle that powers a large Stokes shift red fluorescent protein. *Angew. Chem. Int. Ed.* **62**, e202212209 (2023).
30. Kochendoerfer, G. G., Lin, S. W., Sakmar, T. P. & Mathies, R. A. How color visual pigments are tuned. *Trends Biochem. Sci.* **24**, 300-305 (1999).
31. Callender, R. & Honig, B. Resonance Raman studies of visual pigments. *Annu. Rev. Biophys. Bioeng.* **6**, 33-55 (1977).
32. Rimai, L., Heyde, M. E. & Gill, D. Vibrational spectra of some carotenoids and related linear polyenes. Raman spectroscopic study. *J. Am. Chem. Soc.* **95**, 4493-4501 (1973).
33. Schaffer, H. E., Chance, R. R., Silbey, R. J., Knoll, K. & Schrock, R. R. Conjugation length dependence of Raman scattering in a series of linear polyenes: Implications for polyacetylene. *J. Chem. Phys.* **94**, 4161-4170 (1991).
34. Creemers, T. M. H., Lock, A. J., Subramaniam, V., Jovin, T. M. & Völker, S. Photophysics and optical switching in green fluorescent protein mutants. *Proc. Natl. Acad. Sci. U.S.A.* **97**, 2974-2978 (2000).
35. Bonsma, S. et al. Green and red fluorescent proteins: Photo- and thermally induced dynamics probed by site-selective spectroscopy and hole burning. *ChemPhysChem* **6**, 838-849 (2005).
36. Parson, W. W. *Modern optical spectroscopy: With exercises and examples from biophysics and biochemistry*. (Springer Science & Business Media, 2007).
37. Fünfschilling, J., Glatz, D. & Zschokke-Gränacher, I. Hole-burning spectroscopy as a tool to eliminate inhomogeneous broadening. *J. Lumin.* **36**, 85-92 (1986).
38. Völker, S. Hole-burning spectroscopy. *Annu. Rev. Phys. Chem.* **40**, 499-530 (1989).
39. Mandal, D., Tahara, T. & Meech, S. R. Excited-state dynamics in the green fluorescent protein chromophore. *J. Phys. Chem. B* **108**, 1102-1108 (2004).
40. Kumpulainen, T., Lang, B., Rosspeintner, A. & Vauthey, E. Ultrafast elementary photochemical processes of organic molecules in liquid solution. *Chem. Rev.* **117**, 10826-10939 (2017).

41. Chen, C. et al. Ultrafast excited-state proton transfer dynamics in dihalogenated non-fluorescent and fluorescent GFP chromophores. *J. Chem. Phys.* **152**, 021101 (2020).
42. Unger, S. & Hansch, C. Quantitative models of steric effects. *Prog. Phys. Org. Chem.* **12**, 91-118 (1976).
43. Hansch, C., Leo, A. & Taft, R. W. A survey of Hammett substituent constants and resonance and field parameters. *Chem. Rev.* **91**, 165-195 (1991).
44. Fujita, T. & Nishioka, T. The analysis of the ortho effect. *Prog. Phys. Org. Chem* **12**, 49-89 (1976).
45. Leo, A., Hansch, C. & Elkins, D. Partition coefficients and their uses. *Chem. Rev.* **71**, 525-616 (1971).
46. Cherkasov, A. et al. QSAR modeling: Where have you been? Where are you going to? *J. Med. Chem.* **57**, 4977-5010 (2014).
47. Karelson, M., Lobanov, V. S. & Katritzky, A. R. Quantum-chemical descriptors in QSAR/QSPR studies. *Chem. Rev.* **96**, 1027-1044 (1996).
48. Charton, M. & Motoc, L. *Steric effects in drug design*. (Springer-Verlag Berlin, 1983).
49. Hansch, C. & Leo, A. *Substituent constants for correlation analysis in chemistry and biology*. (Wiley, 1979).
50. Krueger, T. D. et al. Illuminating excited-state intramolecular proton transfer of a fungi-derived red pigment for sustainable functional materials. *J. Phys. Chem. C* **126**, 459-477 (2022).
51. Snellenburg, J., J., Laptenok, S., Seger, R., Mullen, K., M. & Van Stokkum, I., H.M. Glotaran: A Java-based graphical user interface for the R package TIMP. *J. Stat. Softw.* **49**, 1-22 (2012).
52. Agmon, N., Huppert, D., Masad, A. & Pines, E. Excited-state proton-transfer to methanol water mixtures. *J. Phys. Chem.* **95**, 10407-10413 (1991).
53. Han, F., Liu, W. & Fang, C. Excited-state proton transfer of photoexcited pyranine in water observed by femtosecond stimulated Raman spectroscopy. *Chem. Phys.* **422**, 204-219 (2013).
54. Simkovitch, R., Shomer, S., Gepshtein, R. & Huppert, D. How fast can a proton-transfer reaction be beyond the solvent-control limit? *J. Phys. Chem. B* **119**, 2253-2262 (2015).
55. Liu, J., Chen, C., Sokolov, A. I., Baranov, M. S. & Fang, C. Substituent effects in the cationic green fluorescent protein chromophore: Ultrafast excited-state proton transfer or twisting? *ChemPhotoChem* **8**, e202400037 (2024).

56. Spies, C. et al. Solvent dependence of excited-state proton transfer from pyranine-derived photoacids. *Phys. Chem. Chem. Phys.* **16**, 9104-9114 (2014).
57. Simkovitch, R., Karton-Lifshin, N., Shomer, S., Shabat, D. & Huppert, D. Ultrafast excited-state proton transfer to the solvent occurs on a hundred-femtosecond time-scale. *J. Phys. Chem. A* **117**, 3405-3413 (2013).
58. Simkovitch, R. et al. Comprehensive study of ultrafast excited-state proton transfer in water and D_2O providing the missing $RO^{-}\cdots H+$ ion-pair fingerprint. *J. Phys. Chem. A* **118**, 4425-4443 (2014).
59. Simkovitch, R., Shomer, S., Gepshtein, R., Shabat, D. & Huppert, D. Excited-state proton transfer from quinone-cyanine 9 to protic polar-solvent mixtures. *J. Phys. Chem. A* **118**, 1832-1840 (2014).
60. Raucci, U., Chiariello, M. G. & Rega, N. Modeling excited-state proton transfer to solvent: A dynamics study of a super photoacid with a hybrid implicit/explicit solvent model. *J. Chem. Theory Comput.* **16**, 7033-7043 (2020).
61. Lee, C. et al. Excited state proton transfer of quinone cyanine 9: Implications on the origin of super-photoacidity. *ChemPhotoChem* **5**, 245-252 (2021).
62. Laptenok, S. P. et al. Photoacid behaviour in a fluorinated green fluorescent protein chromophore: Ultrafast formation of anion and zwitterion states. *Chem. Sci.* **7**, 5747-5752 (2016).
63. Mayr, H. & Ofial, A. R. The reactivity–selectivity principle: An imperishable myth in organic chemistry. *Angew. Chem. Int. Ed.* **45**, 1844-1854 (2006).
64. Mandal, D. & Shaik, S. Interplay of tunneling, two-state reactivity, and Bell–Evans–Polanyi effects in C–H activation by nonheme Fe(IV)O oxidants. *J. Am. Chem. Soc.* **138**, 2094-2097 (2016).
65. Chen, Y., Chang, K. H., Meng, F. Y., Tseng, S. M. & Chou, P. T. Broadening the horizon of the Bell–Evans–Polanyi principle towards optically triggered structure planarization. *Angew. Chem. Int. Ed.* **60**, 7205-7212 (2021).
66. Liu, Z.-Y. et al. Correlation among hydrogen bond, excited-state intramolecular proton-transfer kinetics and thermodynamics for −OH type proton-donor molecules. *J. Phys. Chem. C* **122**, 21833-21840 (2018).
67. Liu, Z.-Y., Wei, Y.-C. & Chou, P.-T. Correlation between kinetics and thermodynamics for excited-state intramolecular proton transfer reactions. *J. Phys. Chem. A* **125**, 6611-6620 (2021).

68. Liu, W. et al. Panoramic portrait of primary molecular events preceding excited state proton transfer in water. *Chem. Sci.* **7**, 5484-5494 (2016).
69. Tran-Thi, T. H., Gustavsson, T., Prayer, C., Pommeret, S. & Hynes, J. T. Primary ultrafast events preceding the photoinduced proton transfer from pyranine to water. *Chem. Phys. Lett.* **329**, 421-430 (2000).

S9. Appendix (^{1}H and ^{13}C NMR spectra of new compounds)

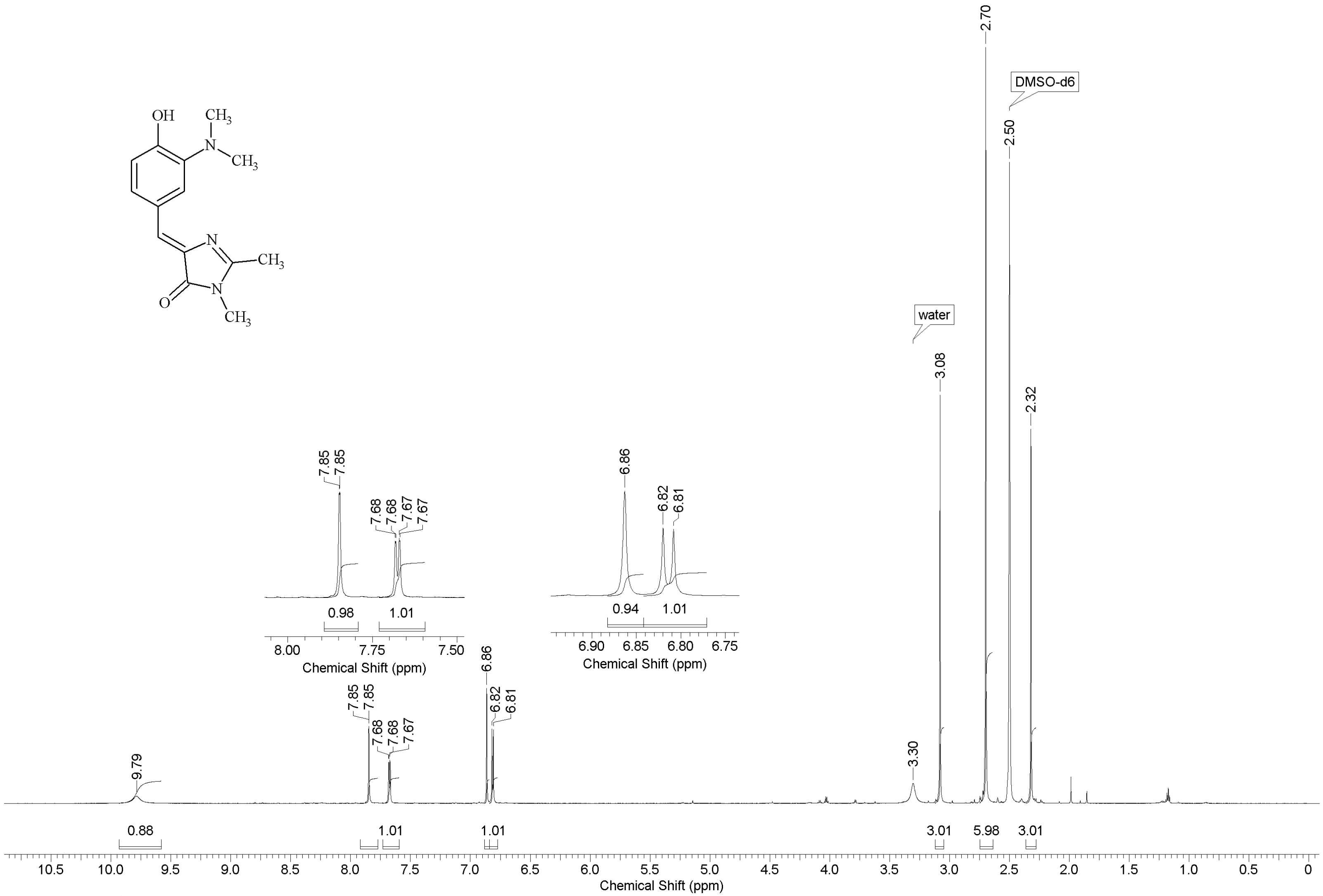

Appendix S1. ^{1}H NMR spectrum of (*Z*)-5-(3-(dimethylamino)-4-hydroxybenzylidene)-2,3-dimethyl-3,5-dihydro-4*H*-imidazol-4-one **(8a)**

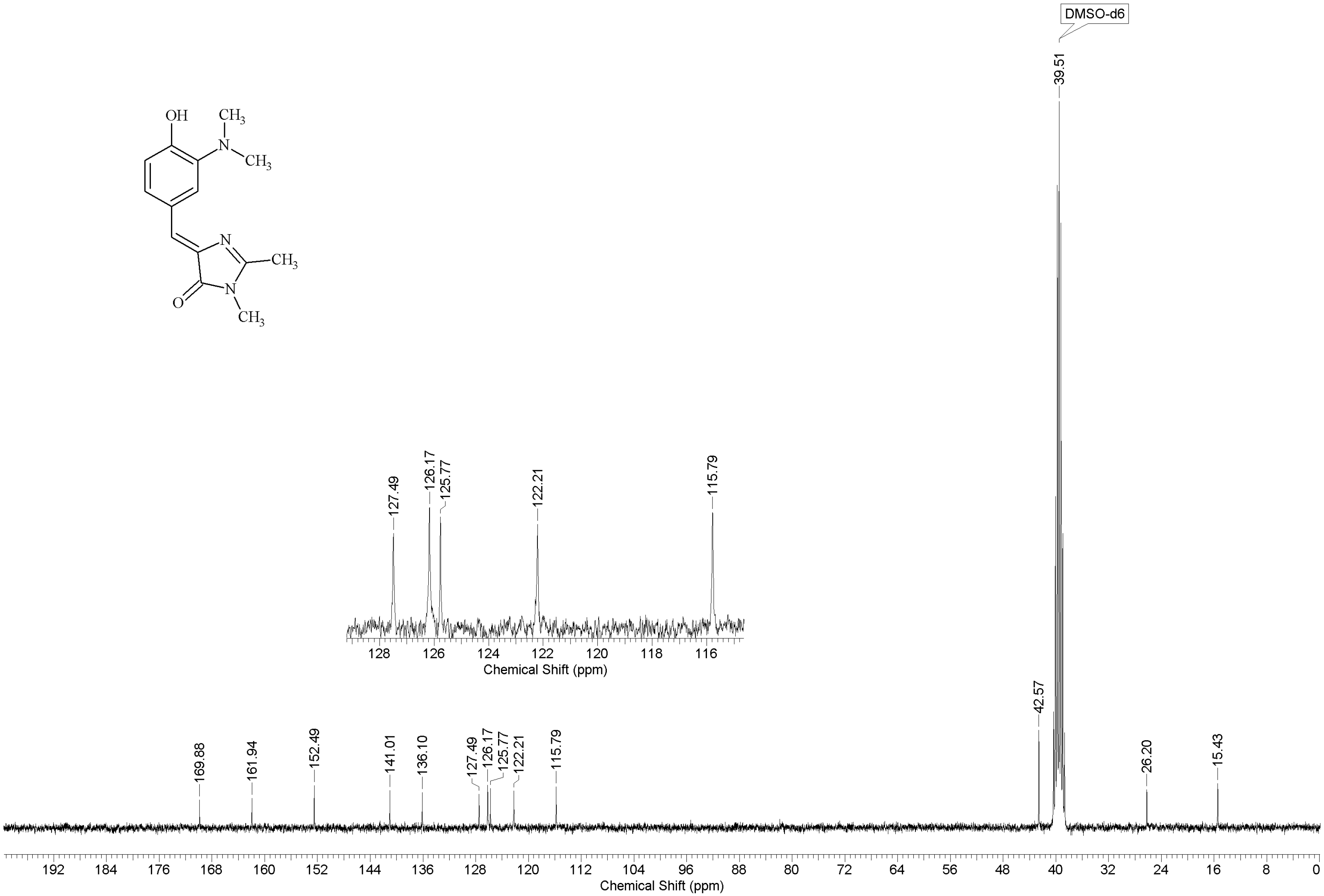

Appendix S2. ^{13}C NMR spectrum of (*Z*)-5-(3-(dimethylamino)-4-hydroxybenzylidene)-2,3-dimethyl-3,5-dihydro-4*H*-imidazol-4-one **(8a)**

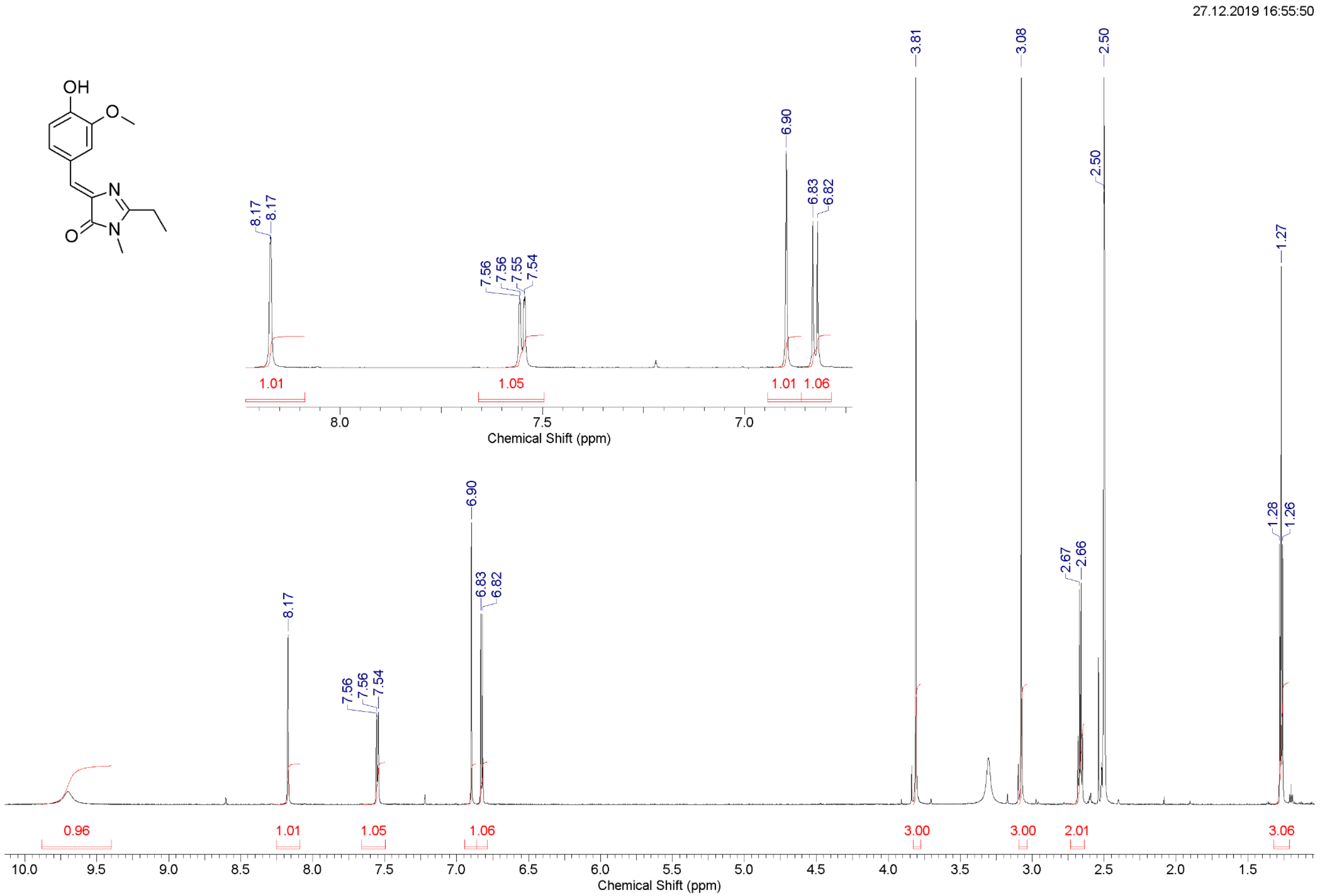

Appendix S3. ^{1}H NMR spectrum of (*Z*)-5-(4-hydroxy-3-methoxybenzylidene)-2-ethyl-3-methyl-3,5-dihydro-4*H*-imidazol-4-one

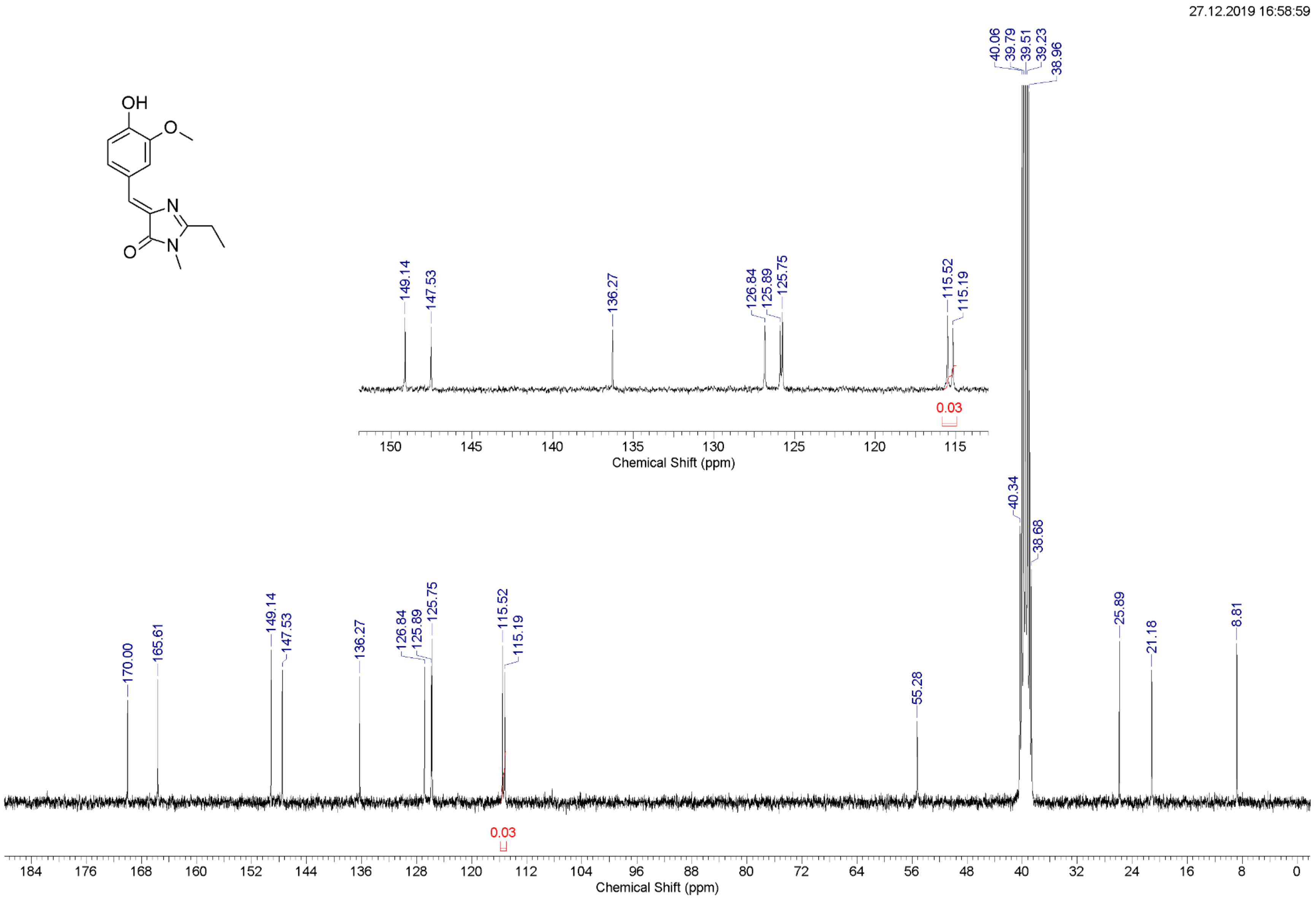

Appendix S4. ^{13}C NMR spectrum of (*Z*)-5-(4-hydroxy-3-methoxybenzylidene)-2-ethyl-3-methyl-3,5-dihydro-4*H*-imidazol-4-one

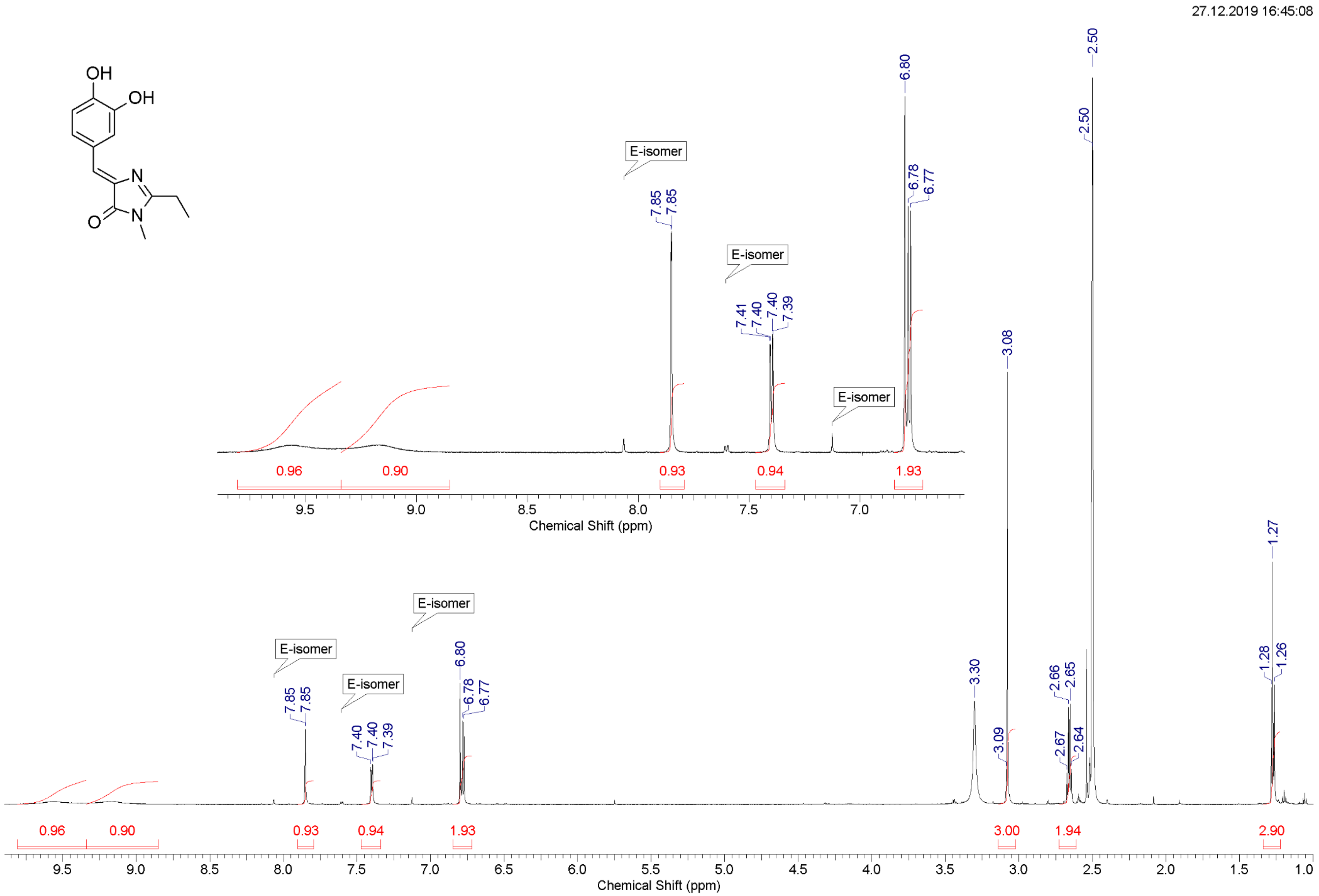

Appendix S5. ^{1}H NMR spectrum of (*Z*)-5-(3,4-dihydroxybenzylidene)-2-ethyl-3-methyl-3,5-dihydro-4*H*-imidazol-4-one

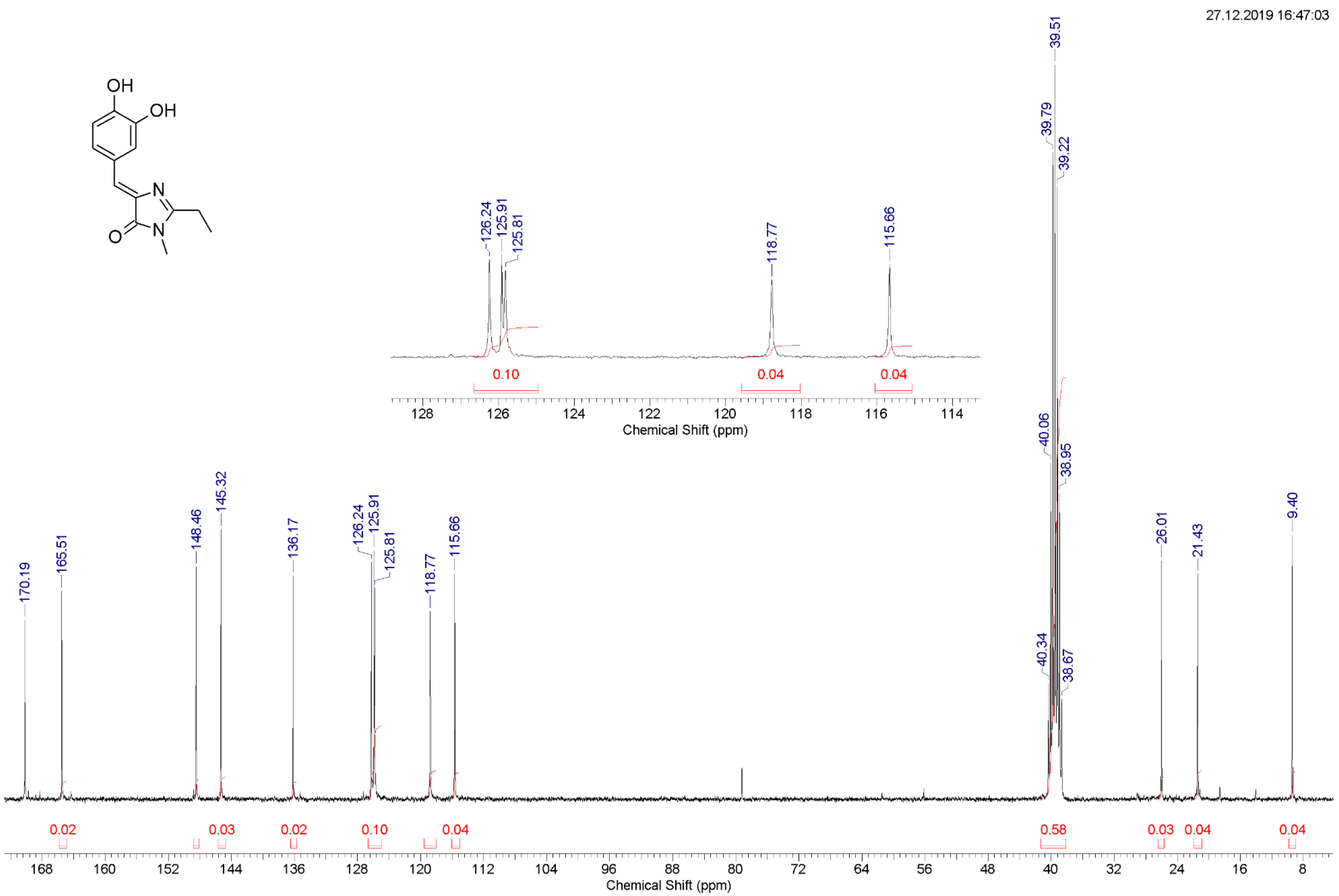

Appendix S6. ^{13}C NMR spectrum of (*Z*)-5-(3,4-dihydroxybenzylidene)-2-ethyl-3-methyl-3,5-dihydro-4*H*-imidazol-4-one

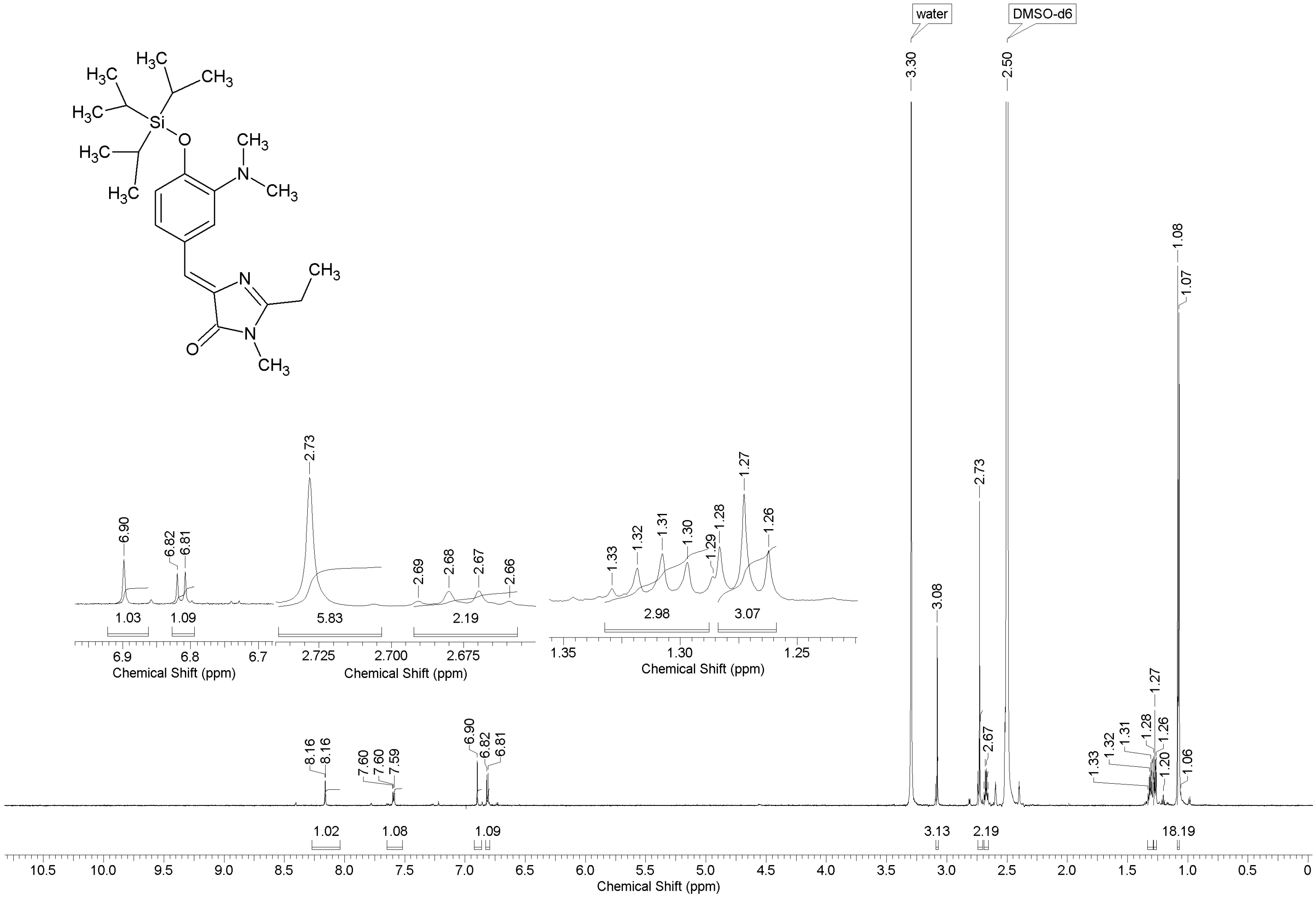

Appendix S7. ¹H NMR spectrum of (*Z*)-5-(3-(dimethylamino)-4-((triisopropylsilyl)oxy)benzylidene)-2-ethyl-3-methyl-3,5-dihydro-4*H*-imidazol-4-one

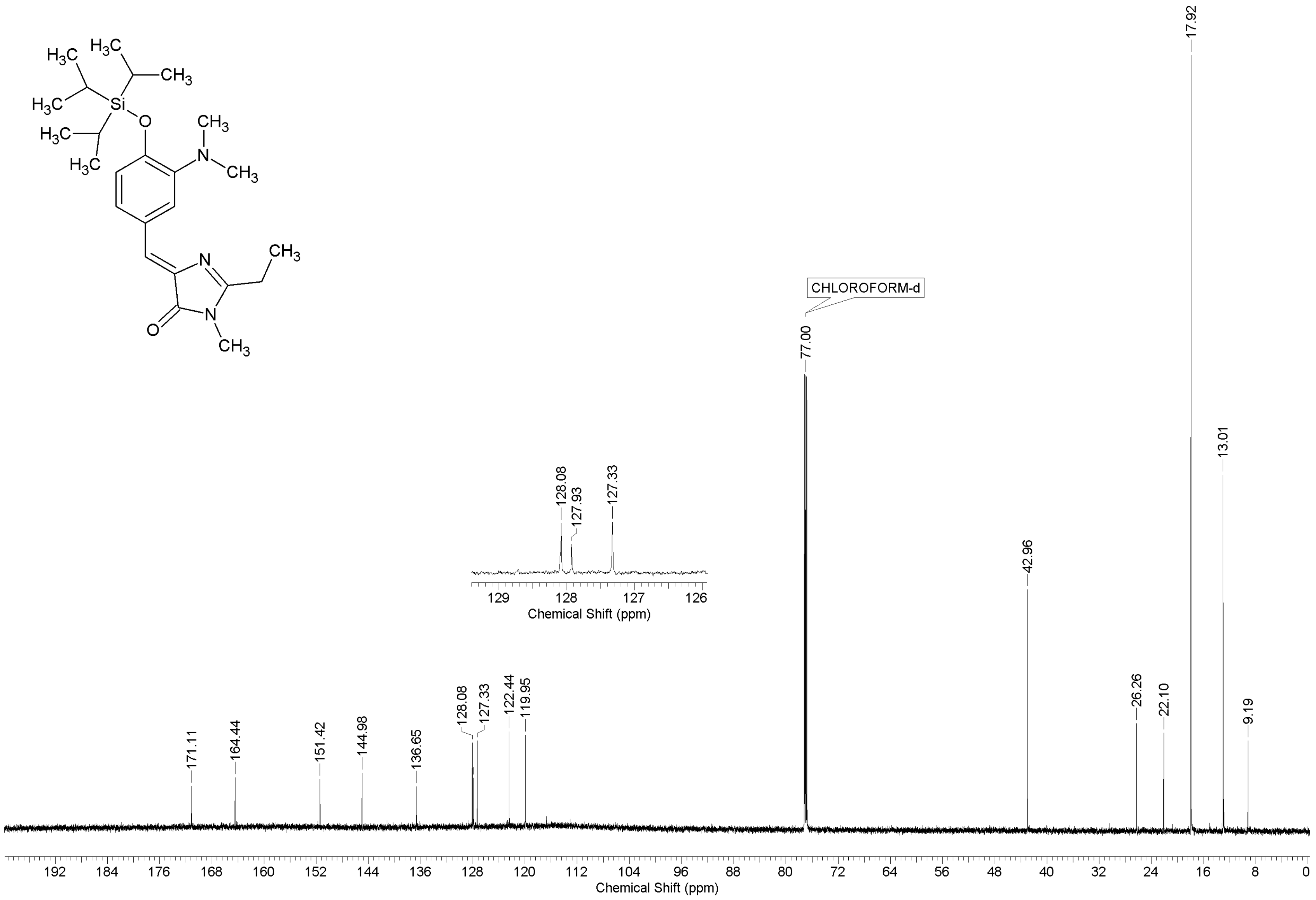

Appendix S8. ^{13}C NMR spectrum of (*Z*)-5-(3-(dimethylamino)-4-((triisopropylsilyl)oxy)benzylidene)-2-ethyl-3-methyl-3,5-dihydro-4*H*-imidazol-4-one

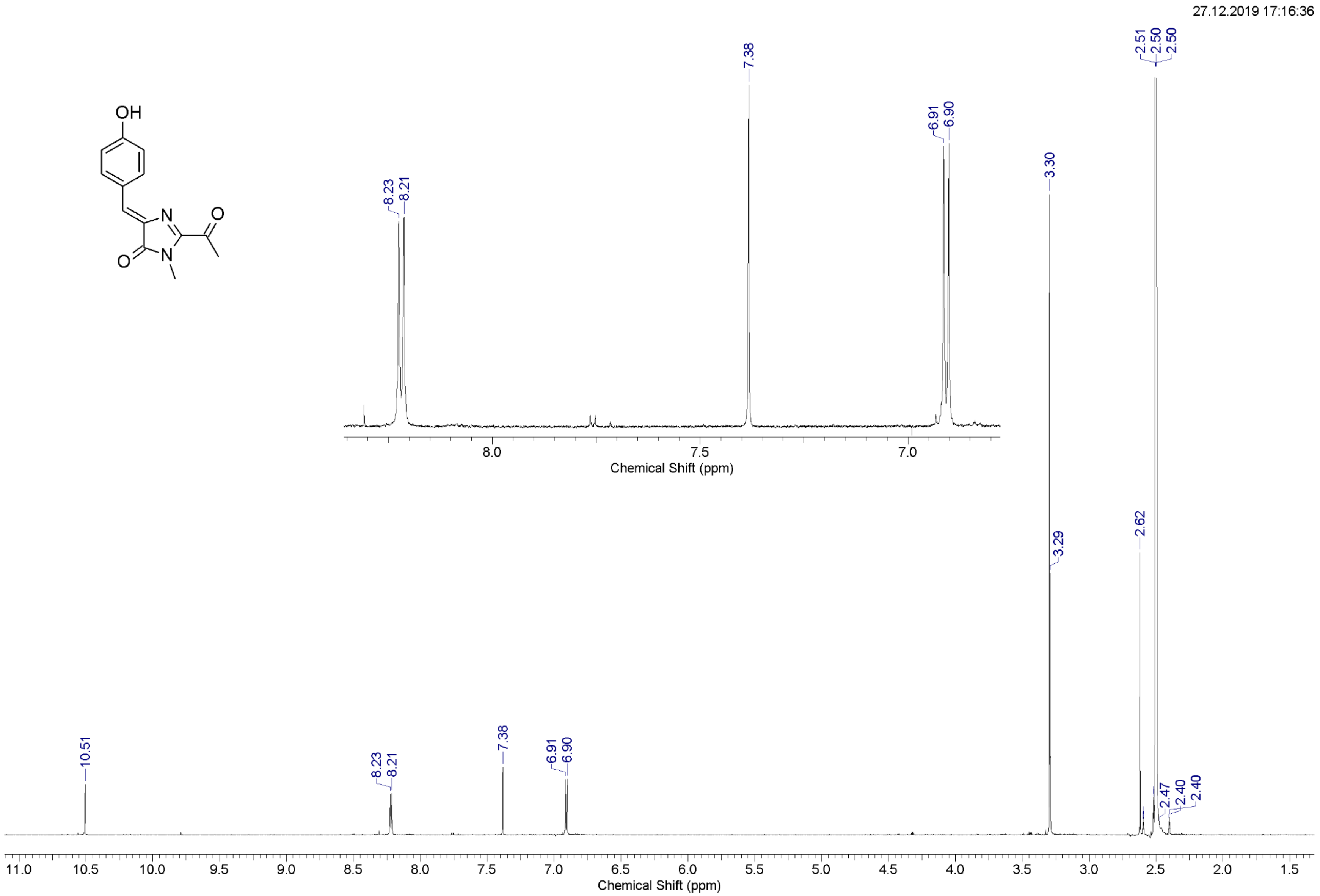

Appendix S9. ^{1}H NMR spectrum of (*Z*)-5-(4-hydroxybenzylidene)-2-acetyl-3-methyl-3,5-dihydro-4*H*-imidazol-4-one **(1c)**

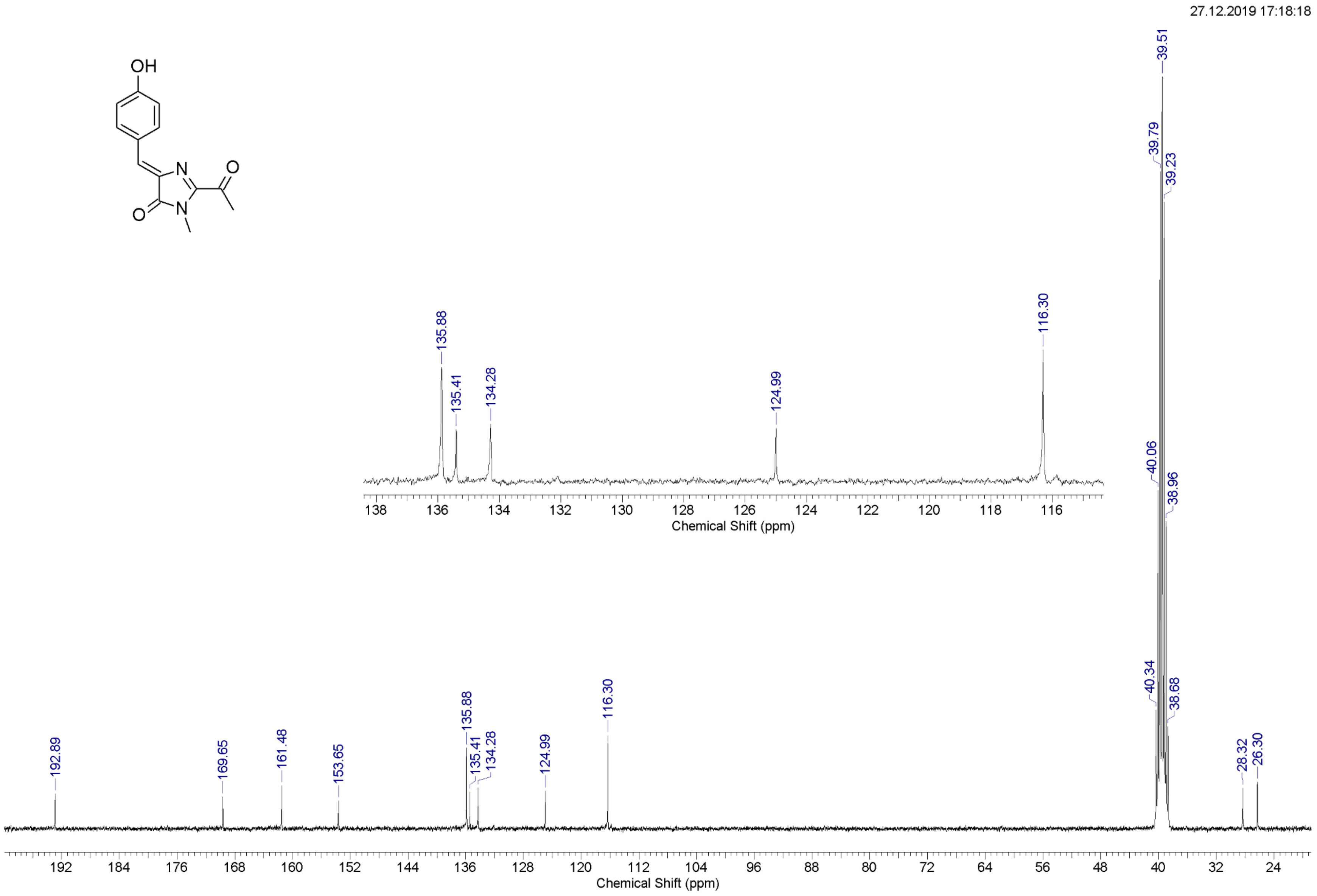

Appendix S10. ^{13}C NMR spectrum of (*Z*)-5-(4-hydroxybenzylidene)-2-acetyl-3-methyl-3,5-dihydro-4*H*-imidazol-4-one **(1c)**

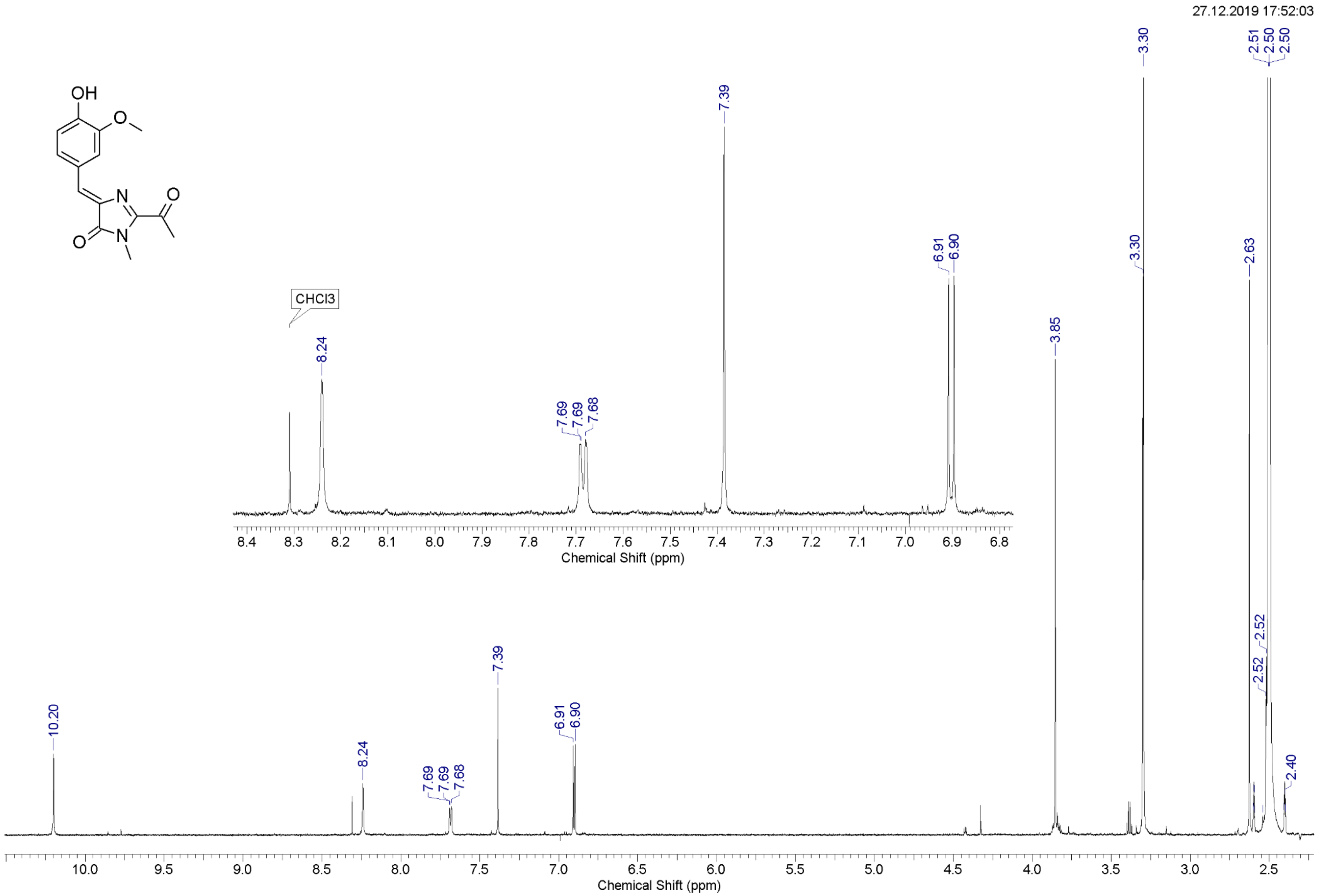

Appendix S11. ^{1}H NMR spectrum of (*Z*)-5-(4-hydroxy-3-methoxybenzylidene)-2-acetyl-3-methyl-3,5-dihydro-4*H*-imidazol-4-one **(5c)**

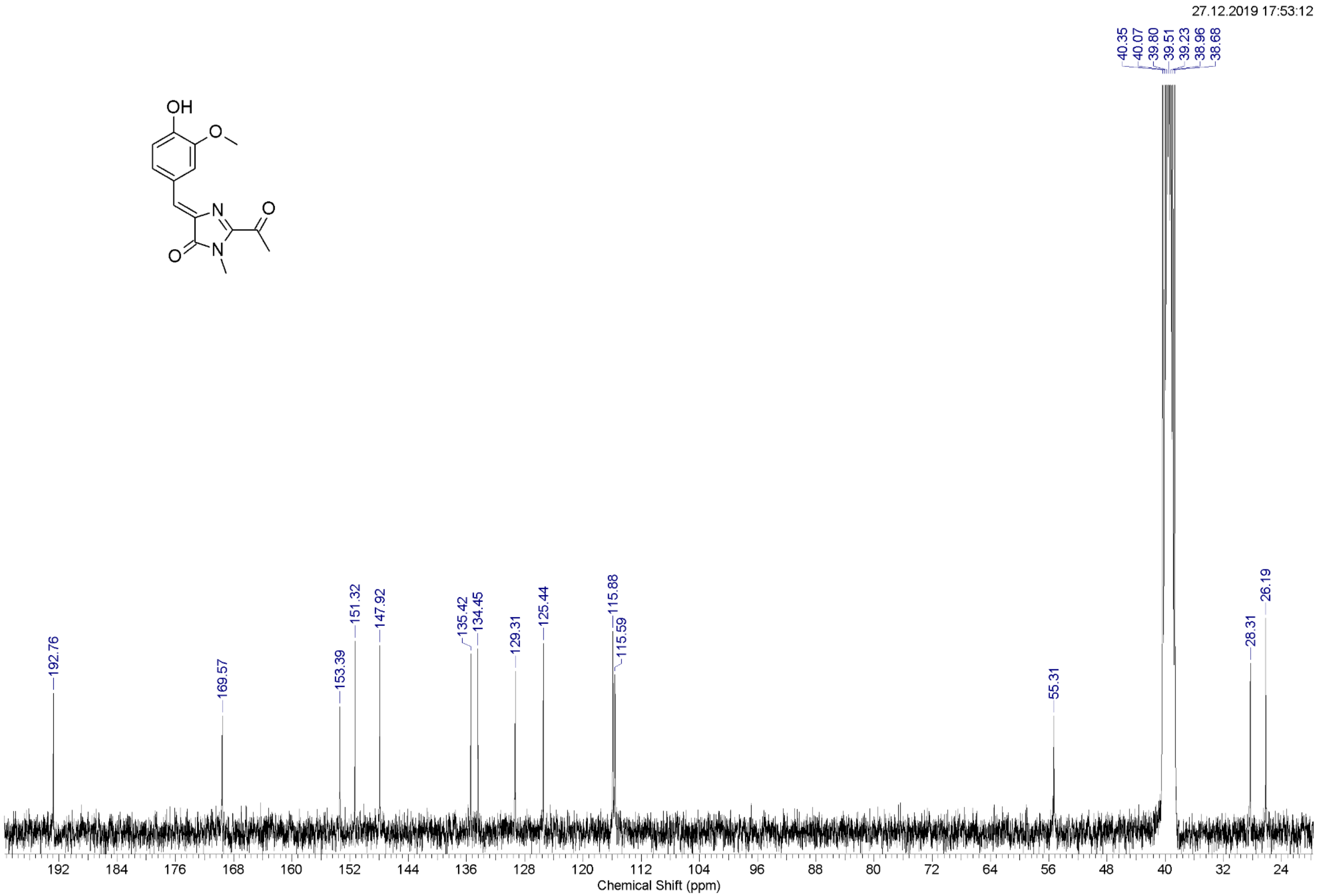

Appendix S12. ^{13}C NMR spectrum of (*Z*)-5-(4-hydroxy-3-methoxybenzylidene)-2-acetyl-3-methyl-3,5-dihydro-4*H*-imidazol-4-one **(5c)**

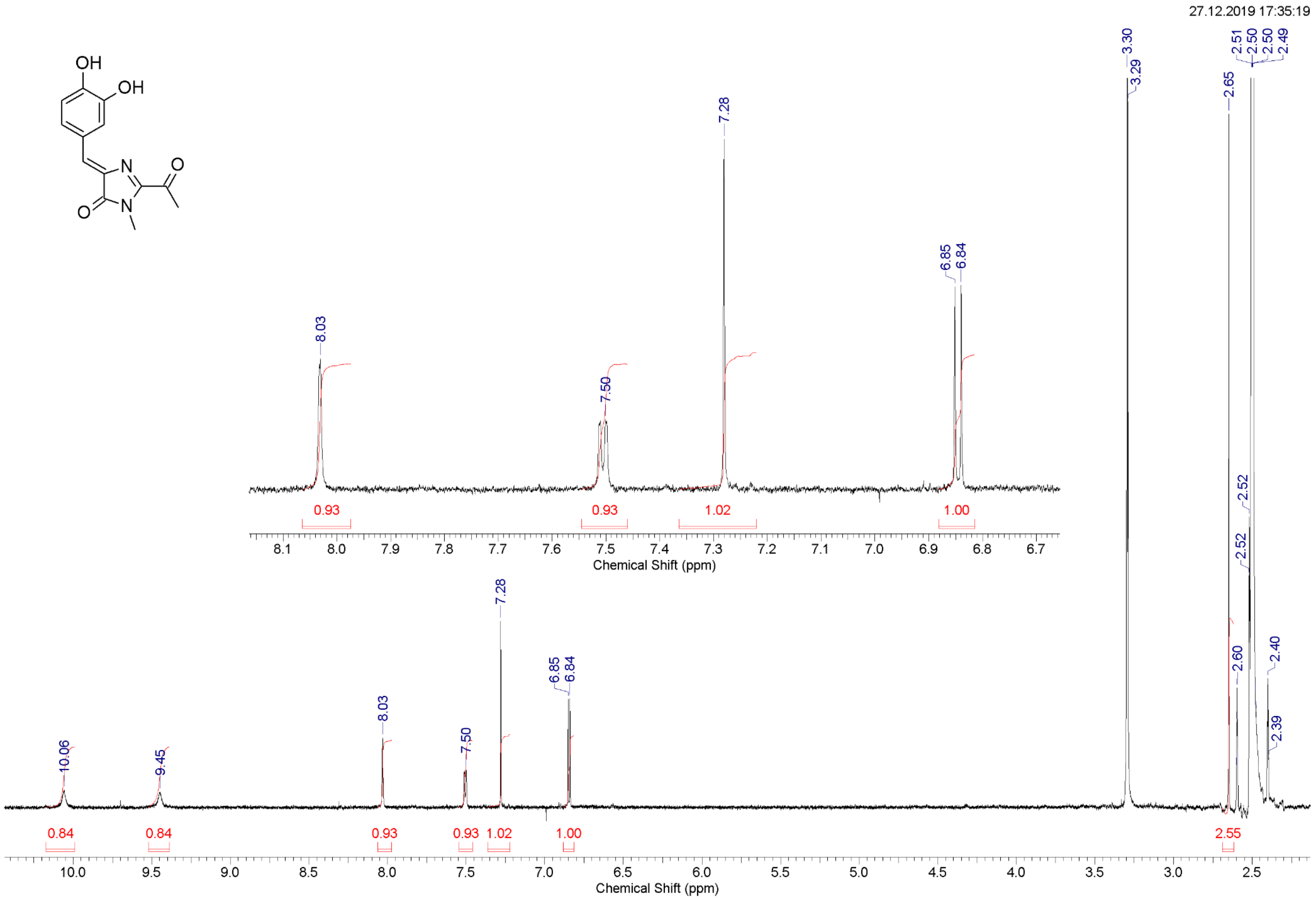

Appendix S13. ^{1}H NMR spectrum of (*Z*)-5-(3,4-dihydroxybenzylidene)-2-acetyl-3-methyl-3,5-dihydro-4*H*-imidazol-4-one **(6c)**

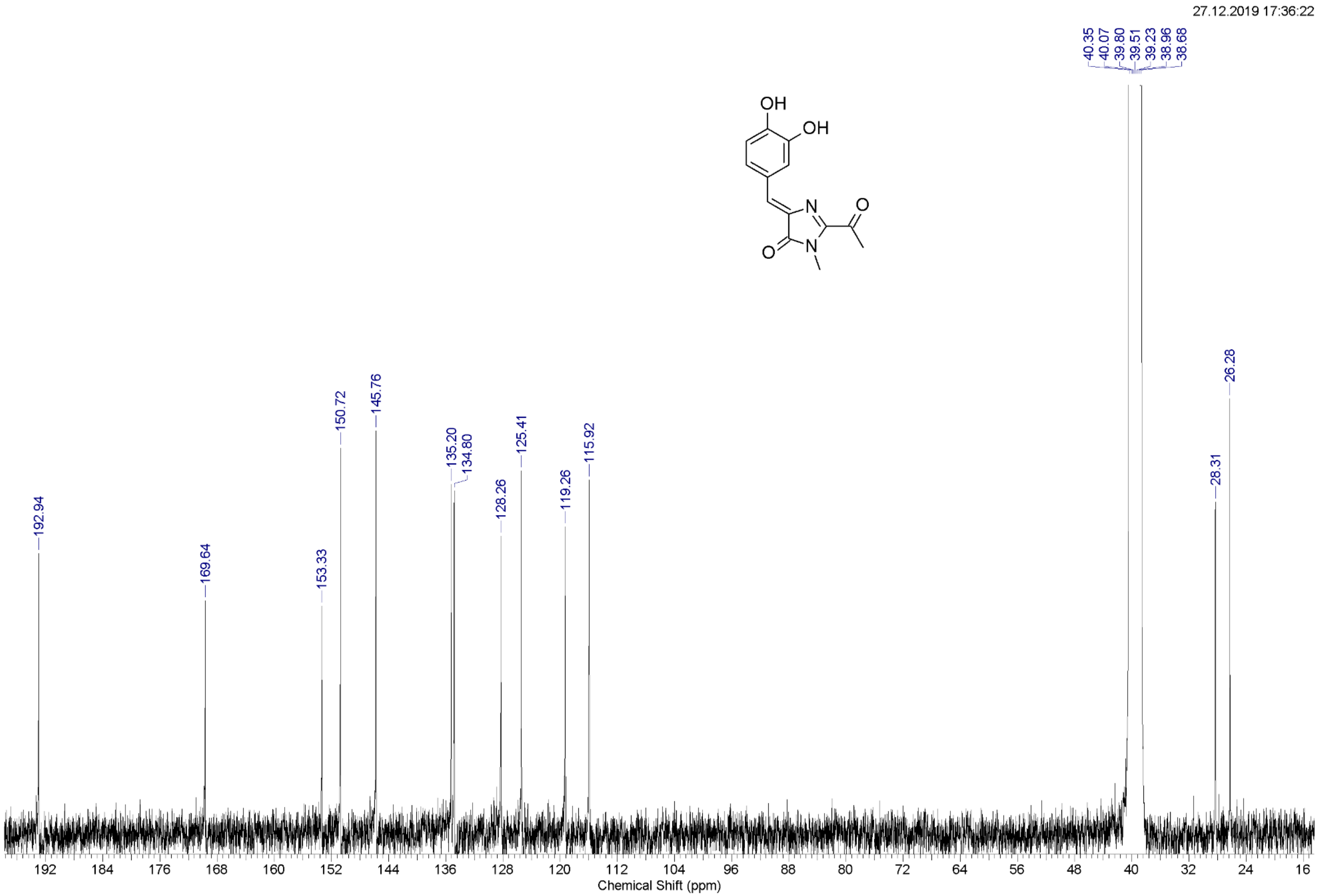

Appendix S14. ^{13}C NMR spectrum of (*Z*)-5-(3,4-dihydroxybenzylidene)-2-acetyl-3-methyl-3,5-dihydro-4*H*-imidazol-4-one **(6c)**

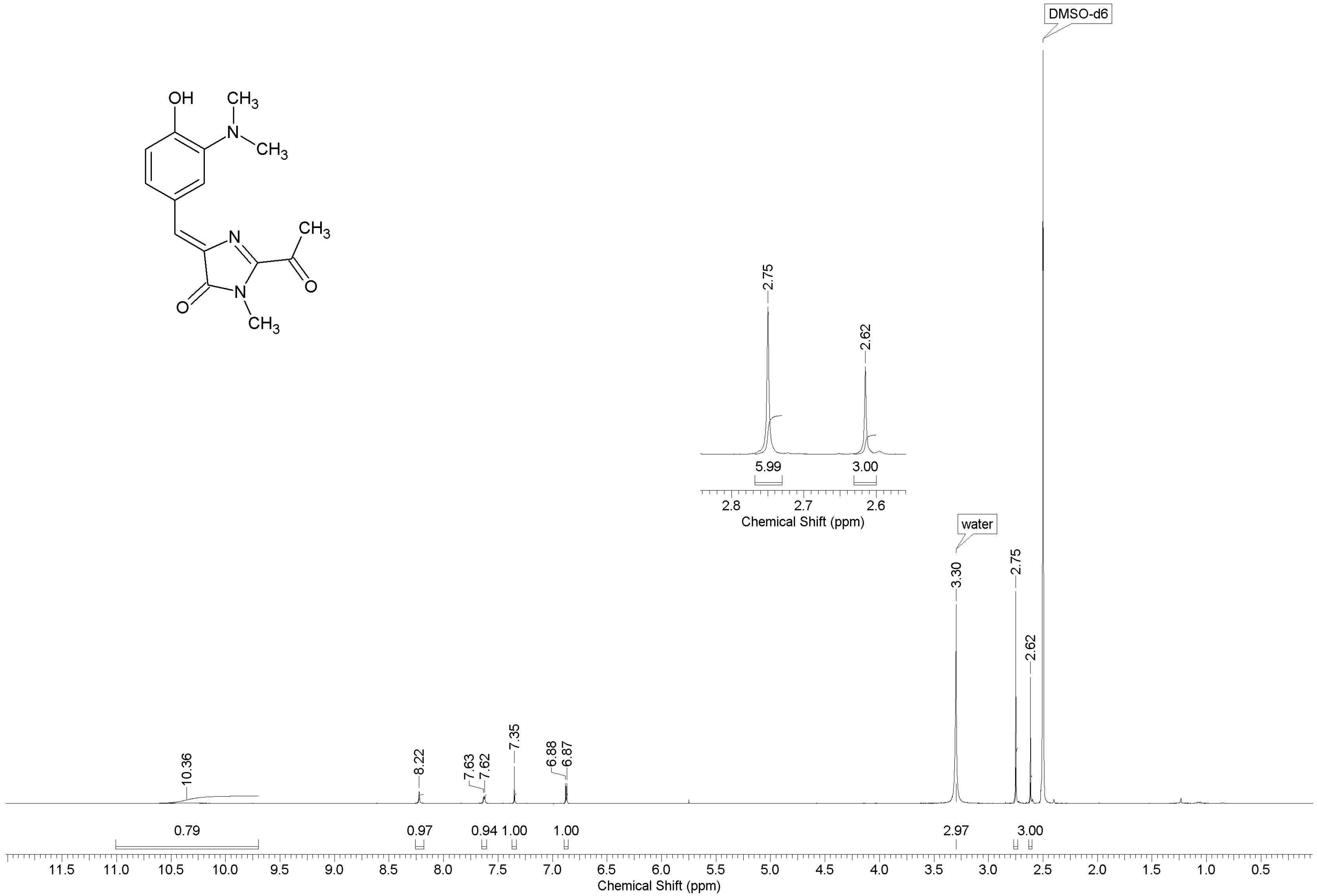

Appendix S15. ^{1}H NMR spectrum of (*Z*)-5-(3-(dimethylamino)-4-hydroxybenzylidene)-2-acetyl-3-methyl-3,5-dihydro-4*H*-imidazol-4-one **(8c)**

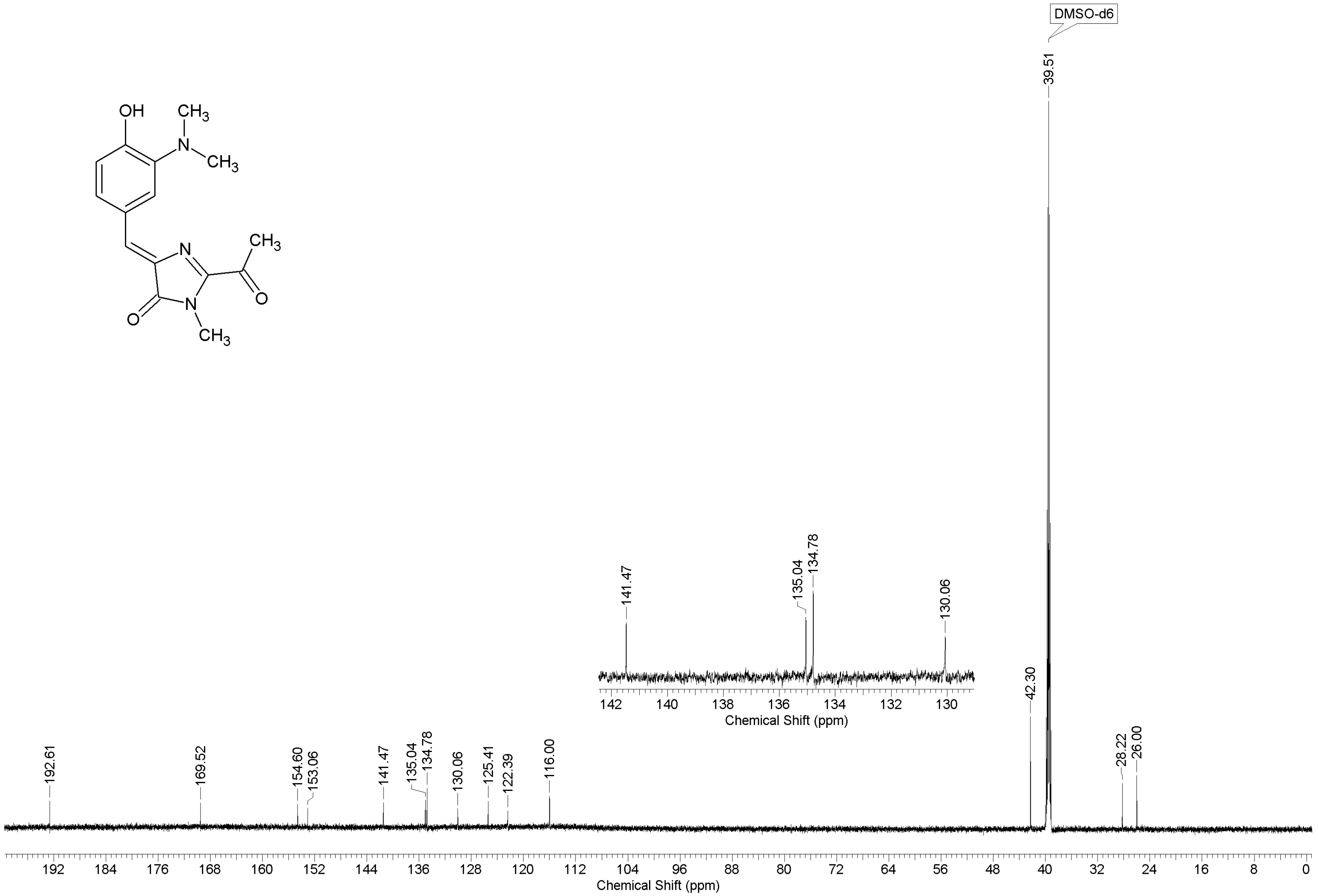

Appendix S16. ^{13}C NMR spectrum of (*Z*)-5-(3-(dimethylamino)-4-hydroxybenzylidene)-2-acetyl-3-methyl-3,5-dihydro-4*H*-imidazol-4-one **(8c)**

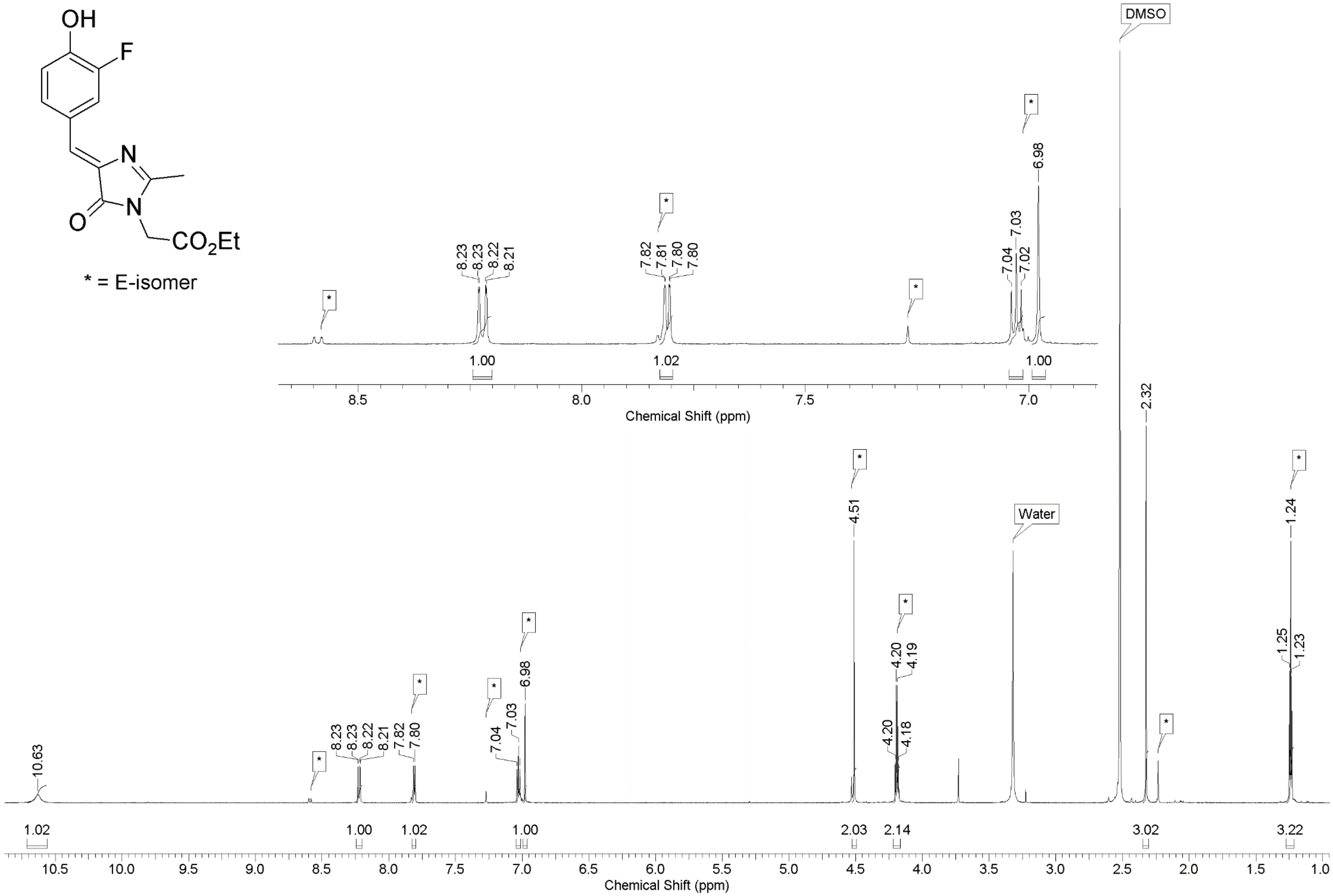

Appendix S17. ^{1}H NMR spectrum of (*Z*)-ethyl-2-(5-(3-fluoro-4-hydroxybenzylidene)-2-methyl-3,5-dihydro-4*H*-imidazol-4-one-3-yl)acetate

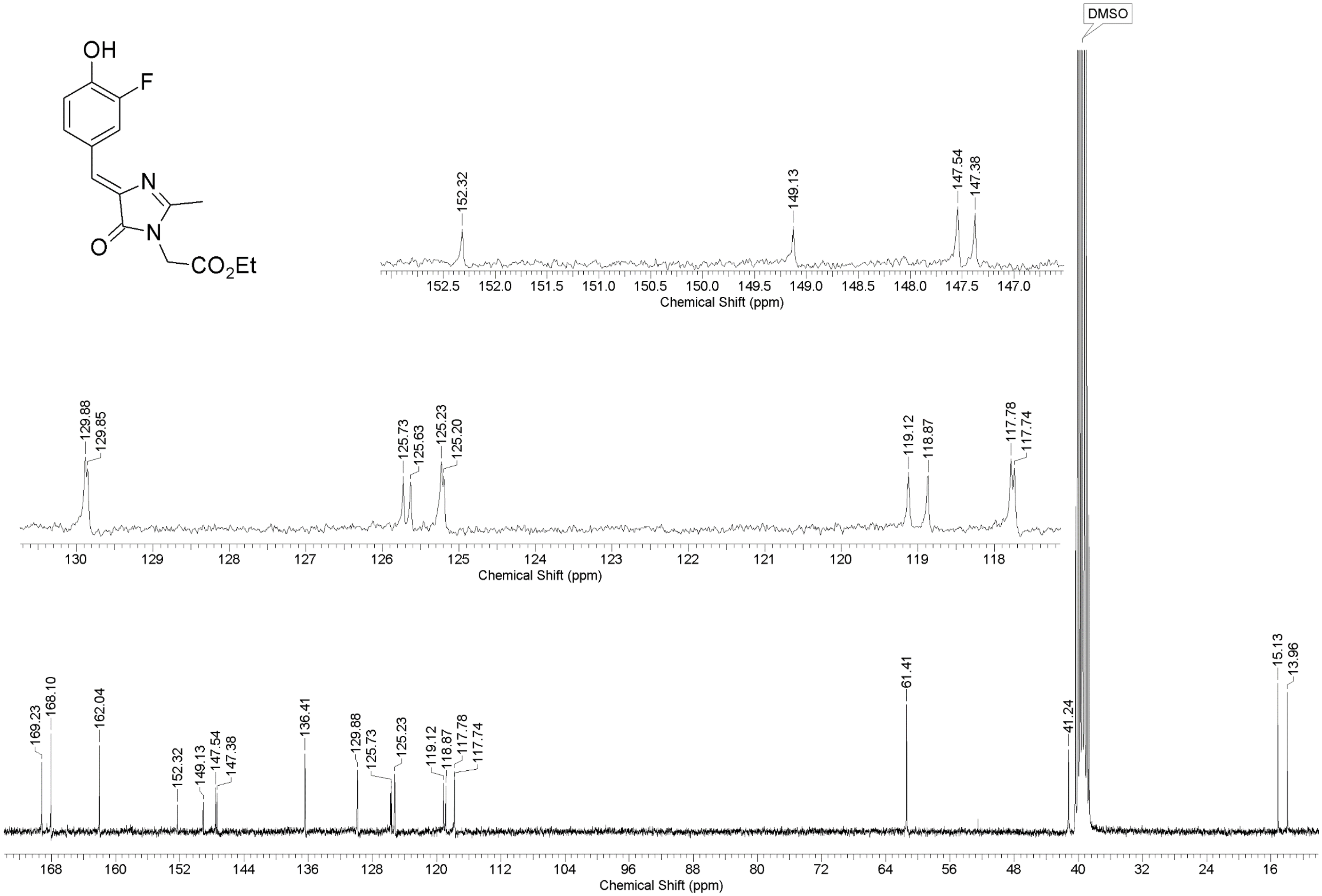

Appendix S18. ^{13}C NMR spectrum of (*Z*)-ethyl-2-(5-(3-fluoro-4-hydroxybenzylidene)-2-methyl-3,5-dihydro-4*H*-imidazol-4-one-3-yl)acetate

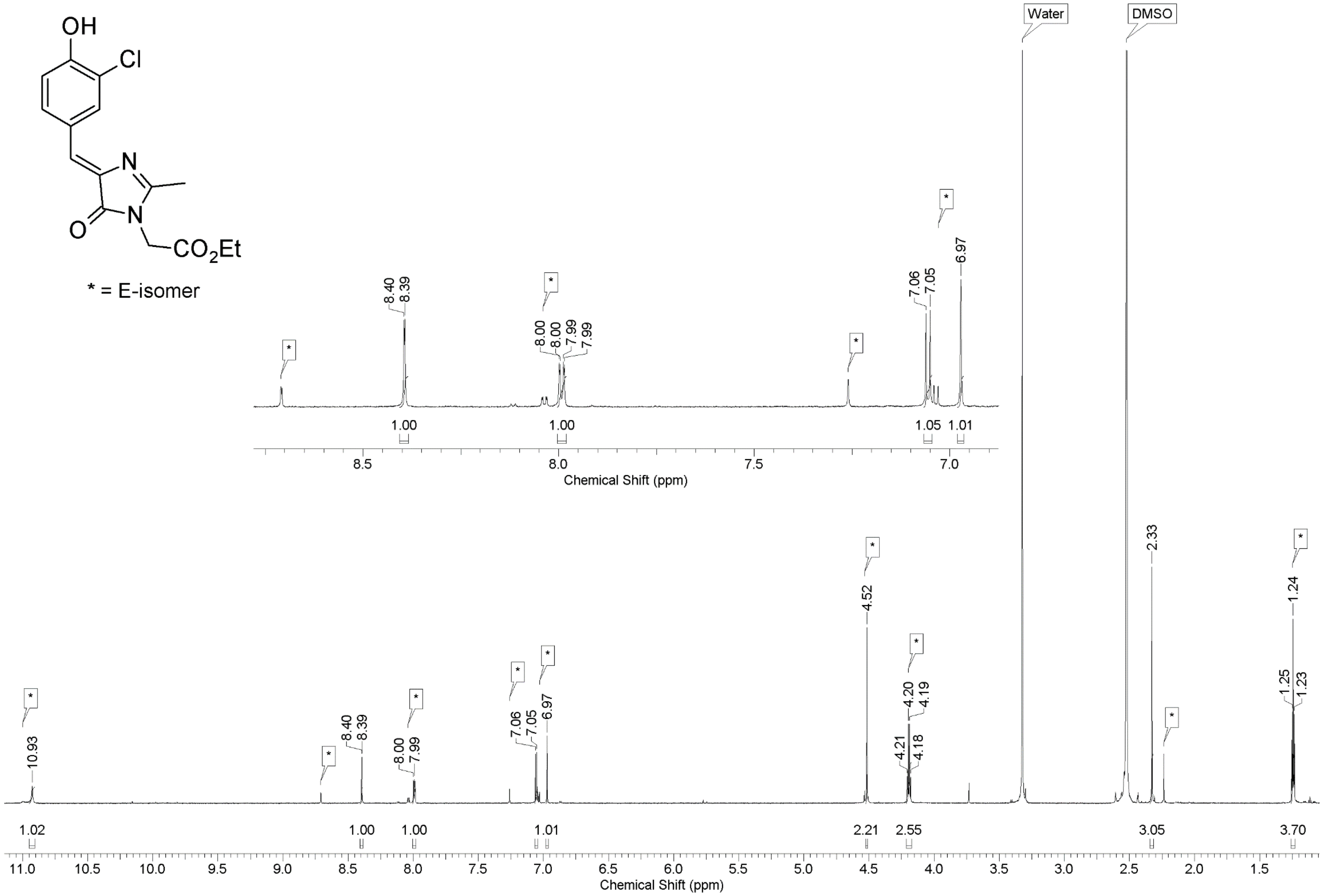

Appendix S19. ^{1}H NMR spectrum of (*Z*)-ethyl-2-(5-(3-chloro-4-hydroxybenzylidene)-2-methyl-3,5-dihydro-4*H*-imidazol-4-one-3-yl)acetate

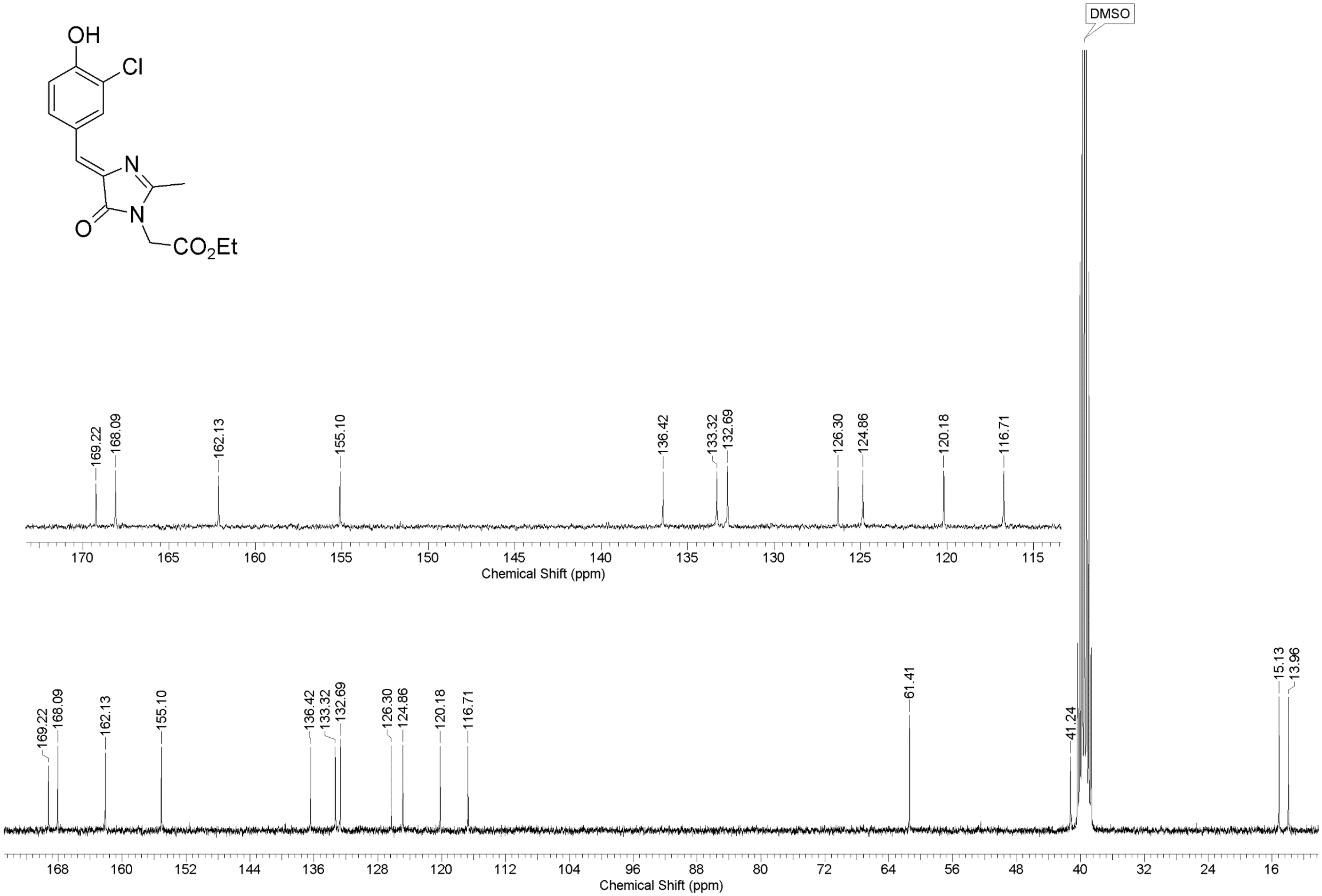

Appendix S20. ^{13}C NMR spectrum of (*Z*)-ethyl-2-(5-(3-chloro-4-hydroxybenzylidene)-2-methyl-3,5-dihydro-4*H*-imidazol-4-one-3-yl)acetate

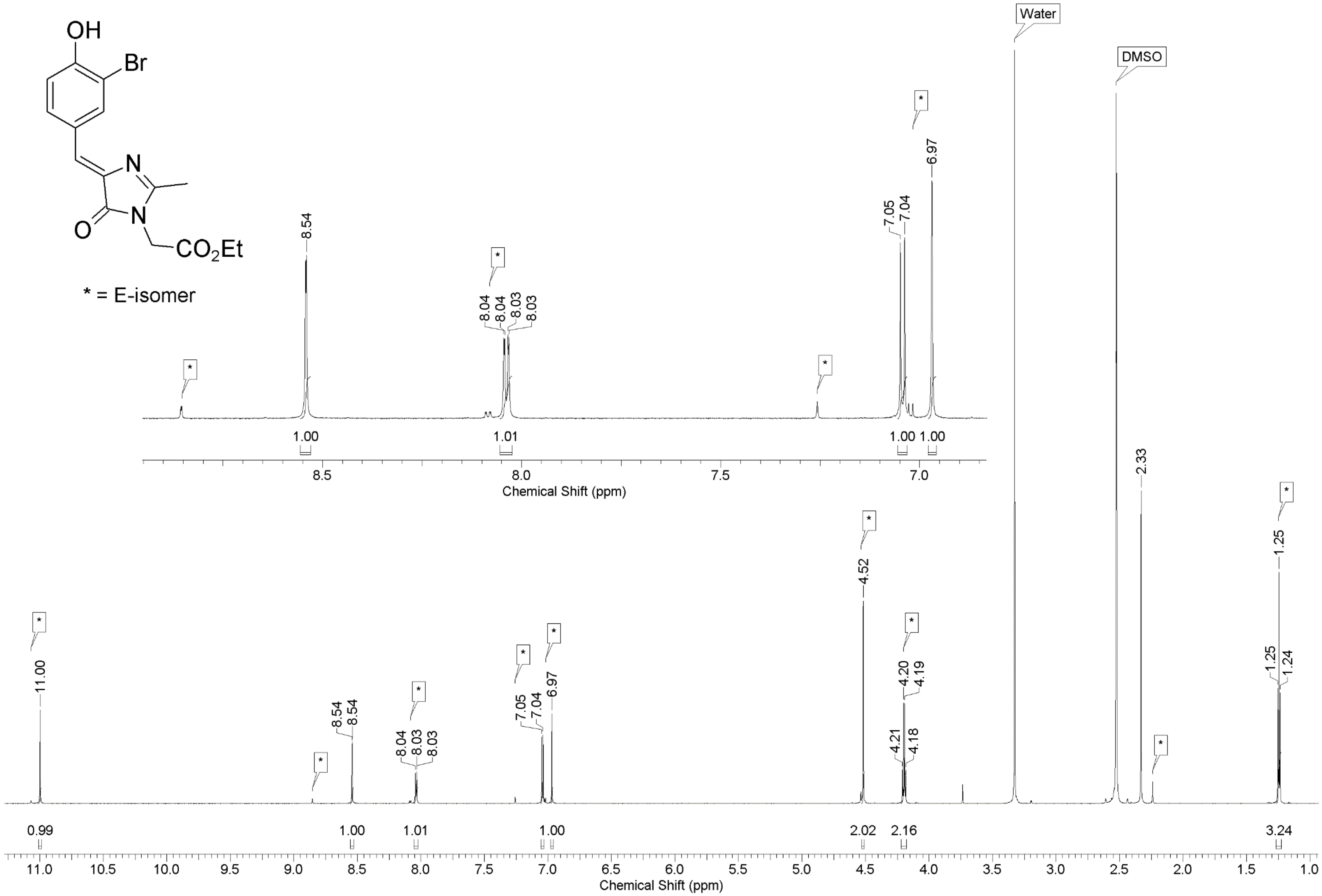

Appendix S21. ^{1}H NMR spectrum of (*Z*)-ethyl-2-(5-(3-bromo-4-hydroxybenzylidene)-2-methyl-3,5-dihydro-4*H*-imidazol-4-one-3-yl)acetate

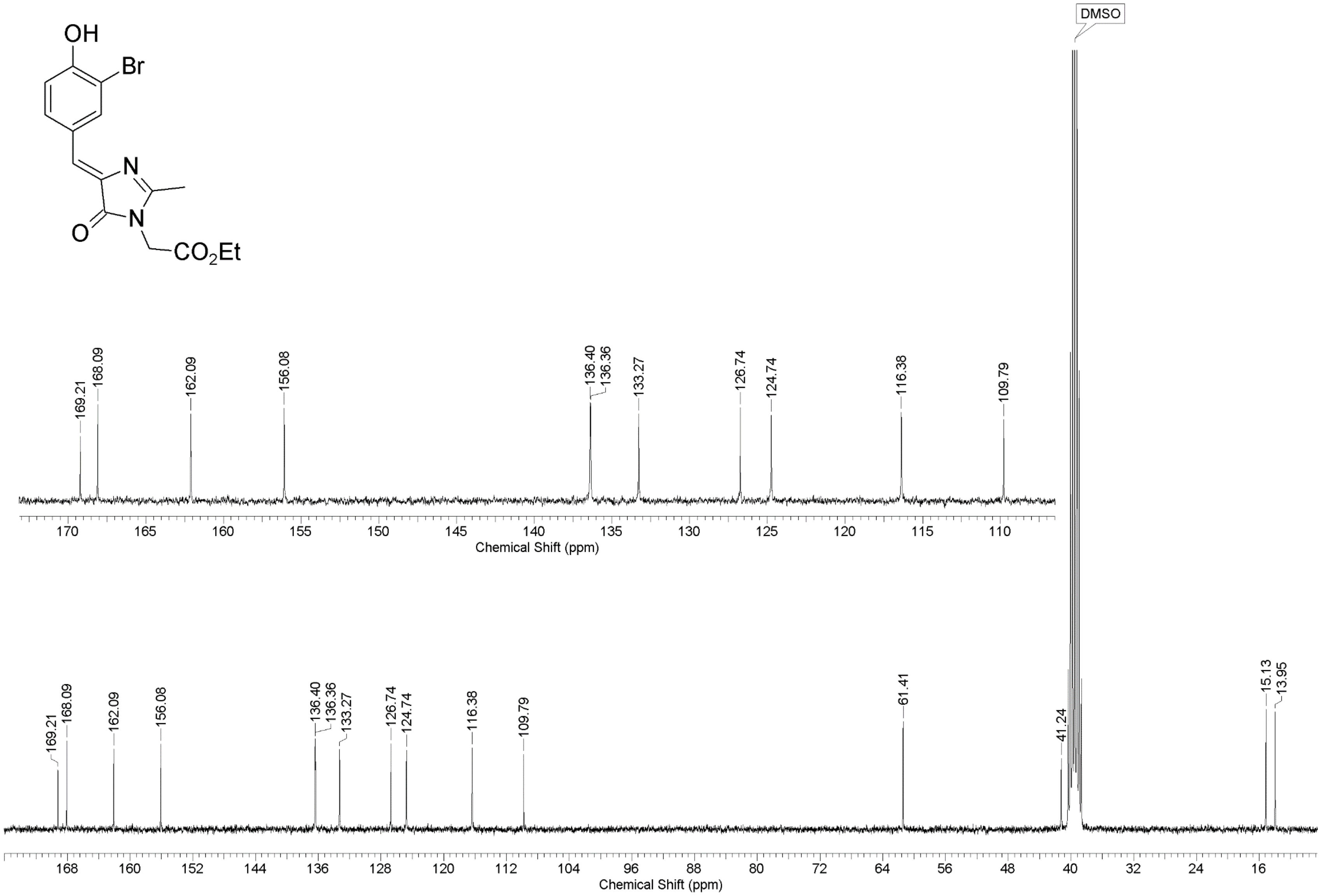

Appendix S22. ^{13}C NMR spectrum of (*Z*)-ethyl-2-(5-(3-bromo-4-hydroxybenzylidene)-2-methyl-3,5-dihydro-4*H*-imidazol-4-one-3-yl)acetate

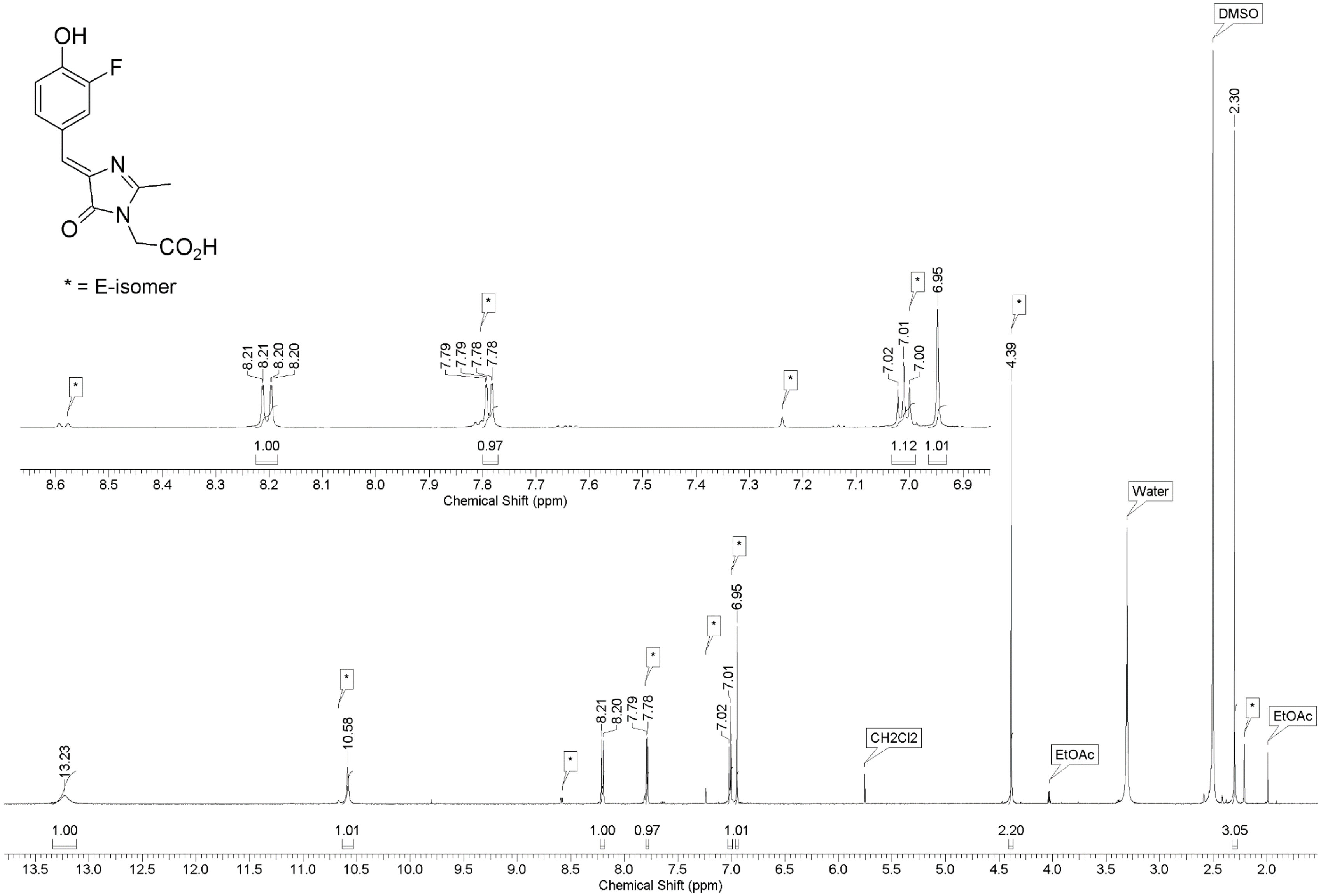

Appendix S23. ^{1}H NMR spectrum of (*Z*)-2-(5-(3-fluoro-4-hydroxybenzylidene)-2-methyl-3,5-dihydro-4*H*-imidazol-4-one-3-yl)acetic acid

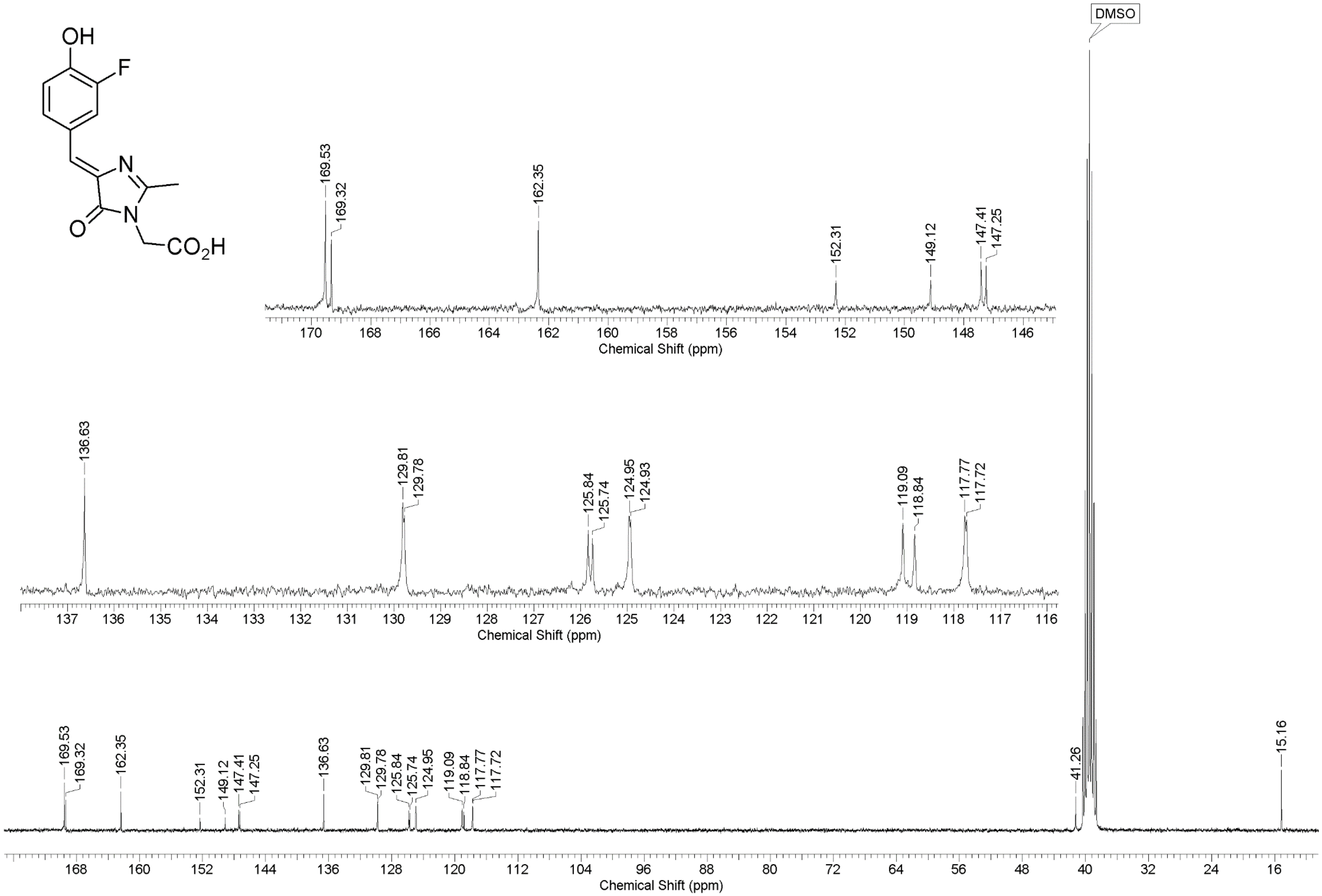

Appendix S24. ^{13}C NMR spectrum of (*Z*)-2-(5-(3-fluoro-4-hydroxybenzylidene)-2-methyl-3,5-dihydro-4*H*-imidazol-4-one-3-yl)acetic acid

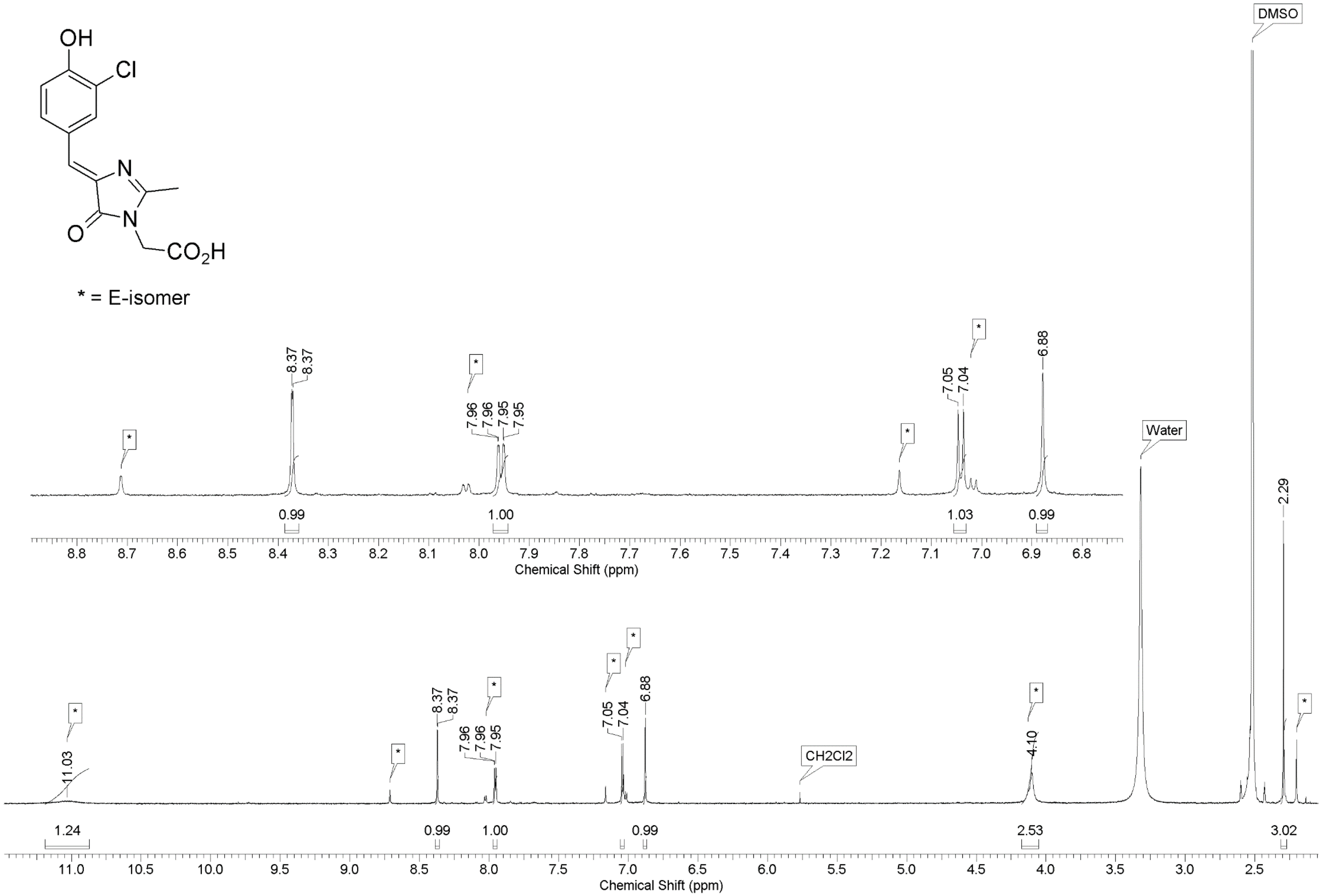

Appendix S25. ^{1}H NMR spectrum of (*Z*)-2-(5-(3-chloro-4-hydroxybenzylidene)-2-methyl-3,5-dihydro-4*H*-imidazol-4-one-3-yl)acetic acid

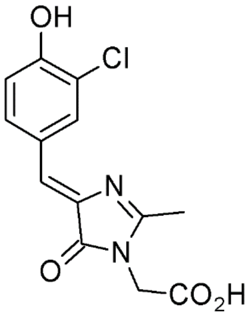

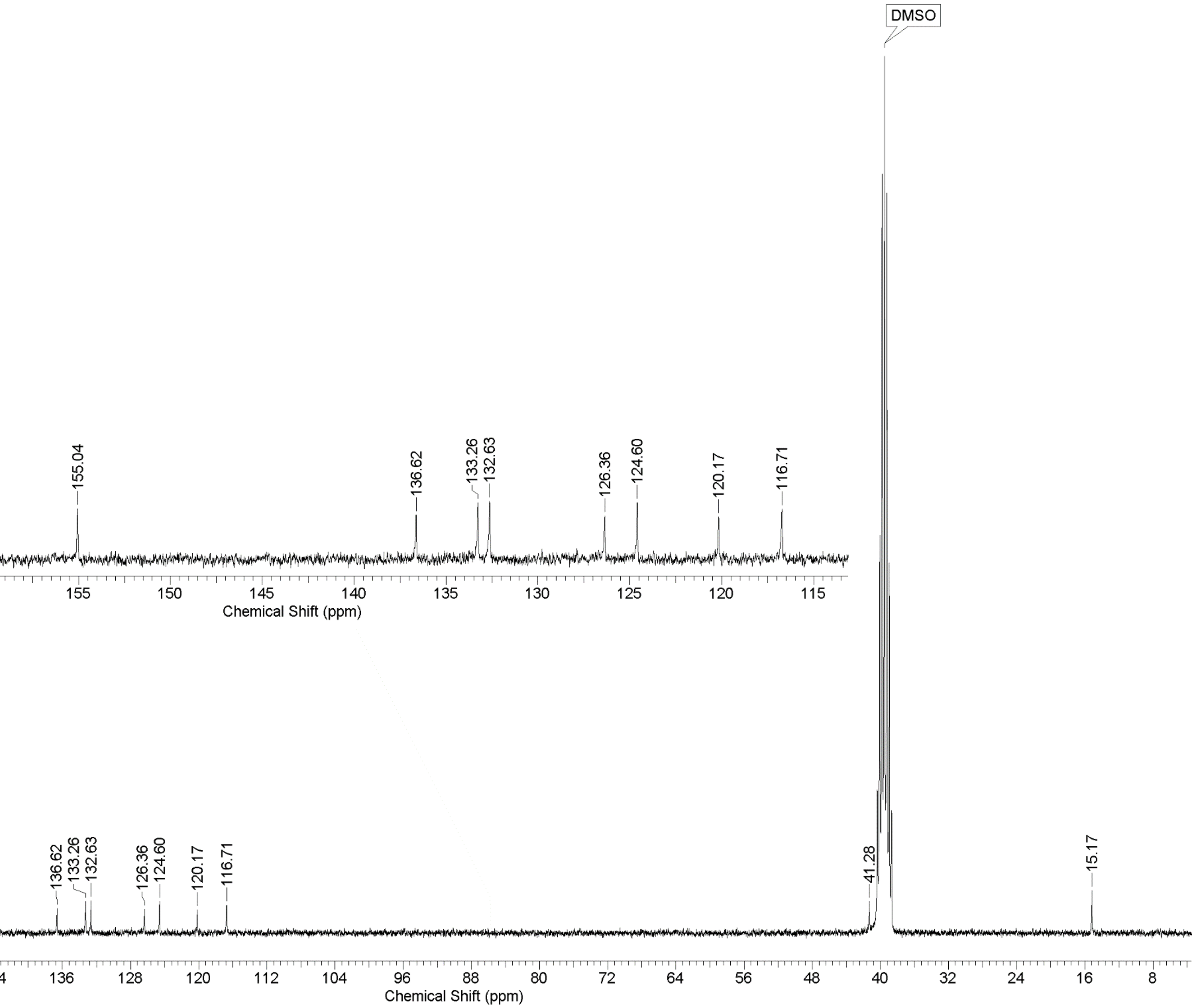

Appendix S26. ^{13}C NMR spectrum of (*Z*)-2-(5-(3-chloro-4-hydroxybenzylidene)-2-methyl-3,5-dihydro-4*H*-imidazol-4-one-3-yl)acetic acid

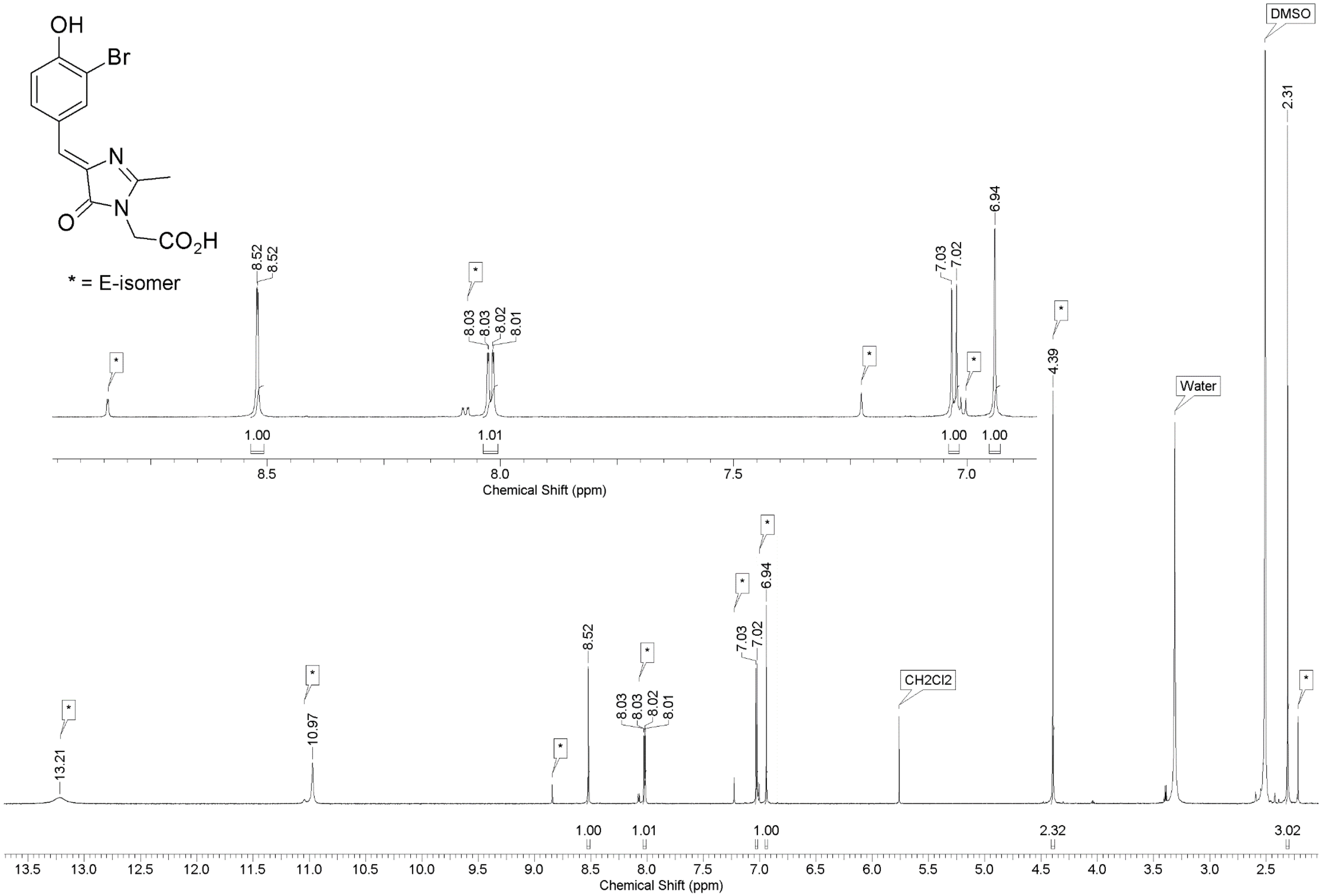

Appendix S27. ^{1}H NMR spectrum of (*Z*)-2-(5-(3-bromo-4-hydroxybenzylidene)-2-methyl-3,5-dihydro-4*H*-imidazol-4-one-3-yl)acetic acid

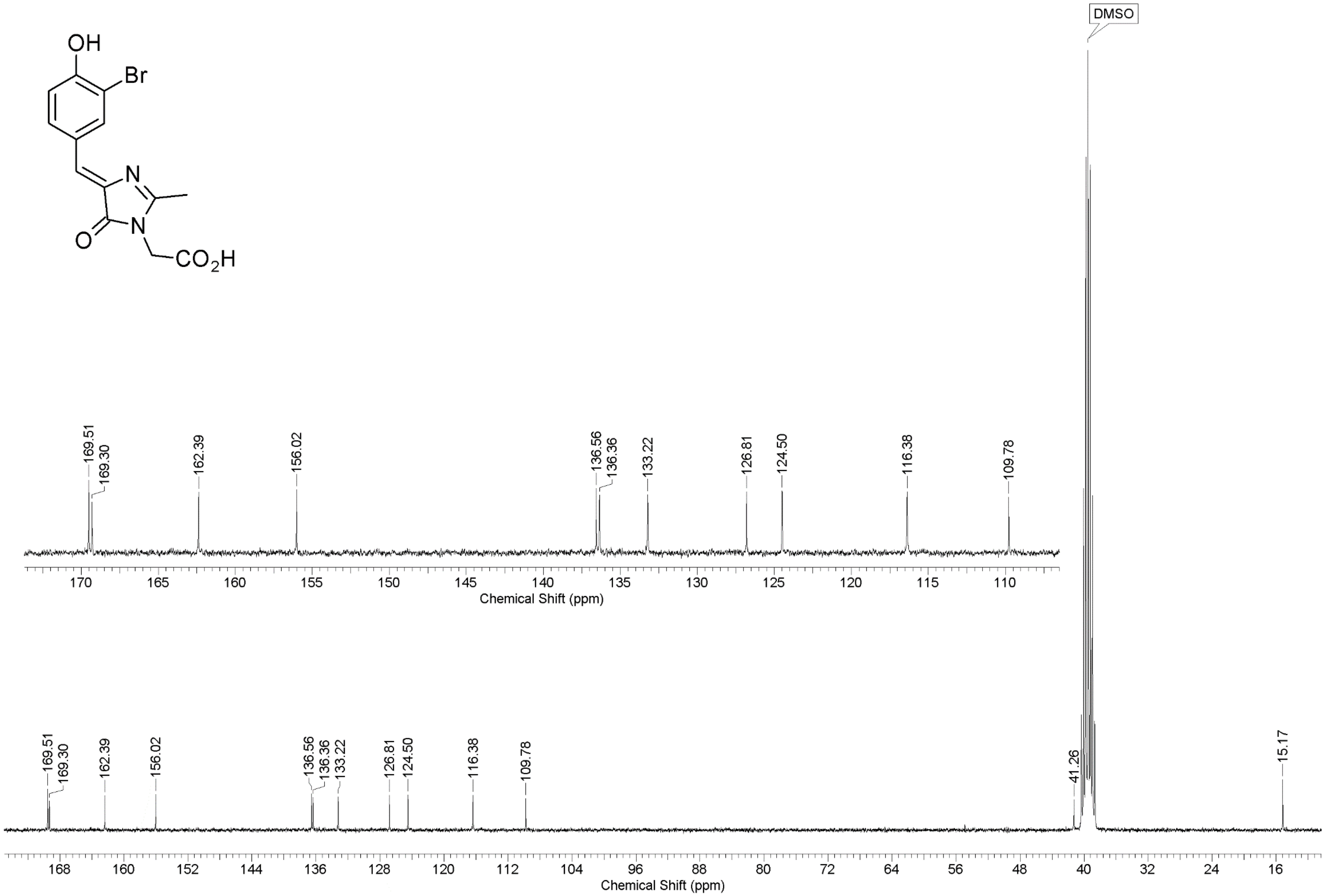

Appendix S28. ^{13}C NMR spectrum of (*Z*)-2-(5-(3-bromo-4-hydroxybenzylidene)-2-methyl-3,5-dihydro-4*H*-imidazol-4-one-3-yl)acetic acid

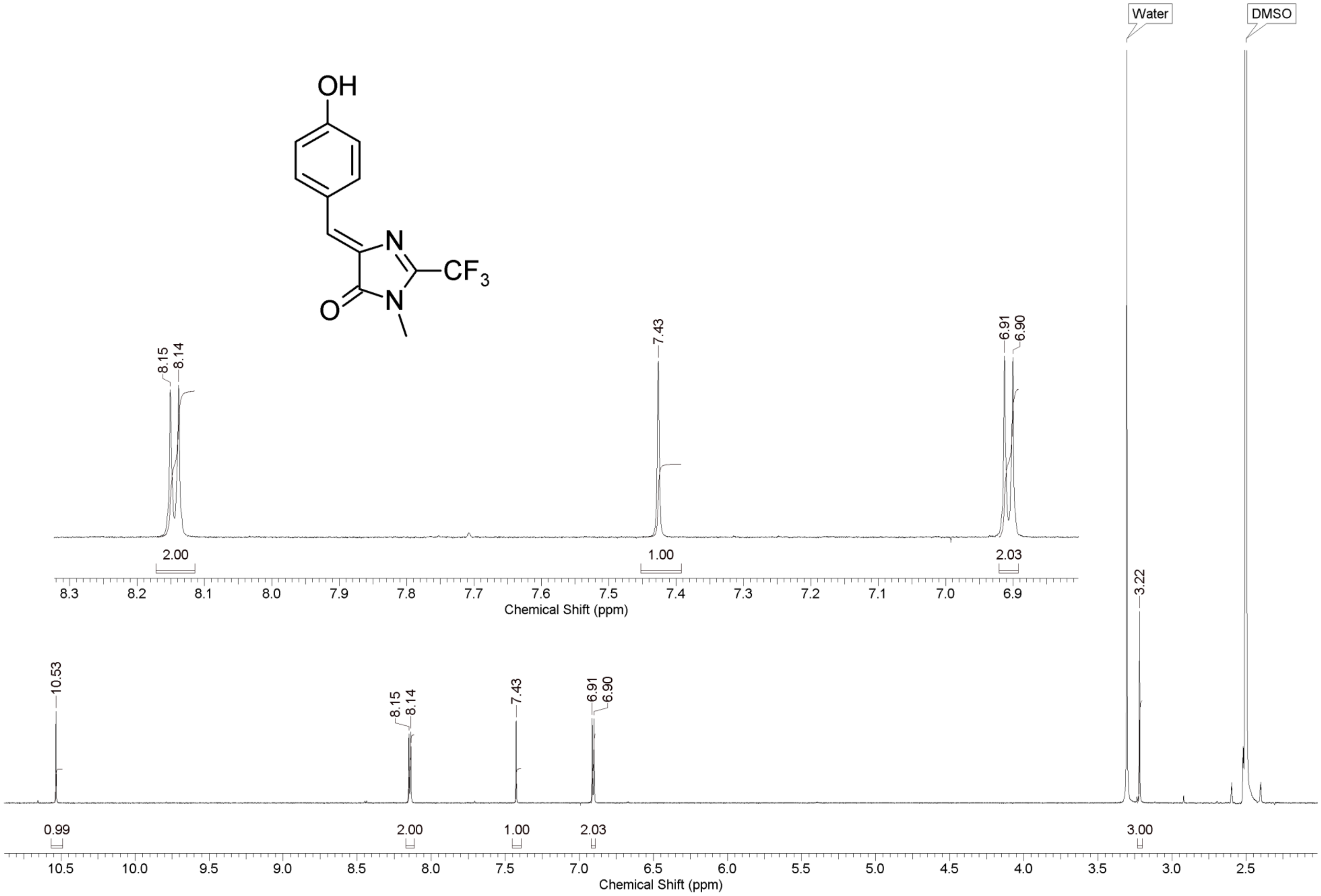

Appendix S29. ^{1}H NMR spectrum of (*Z*)-5-(4-hydroxybenzylidene)-3-methyl-2-(trifluoromethyl)-3,5-dihydro-4*H*-imidazol-4-one **(1b)**

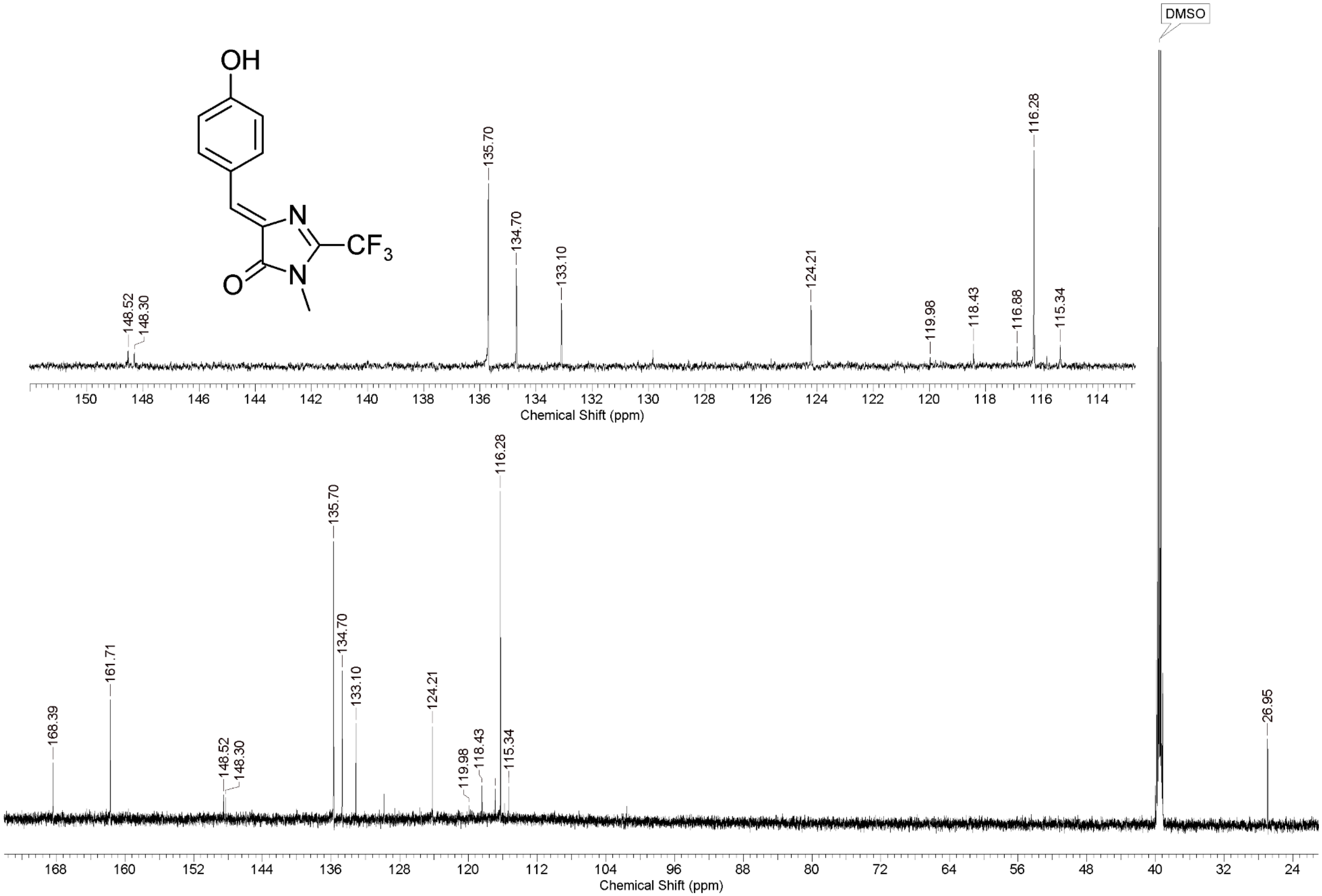

Appendix S30. ^{13}C NMR spectrum of (*Z*)-5-(4-hydroxybenzylidene)-3-methyl-2-(trifluoromethyl)-3,5-dihydro-4*H*-imidazol-4-one **(1b)**

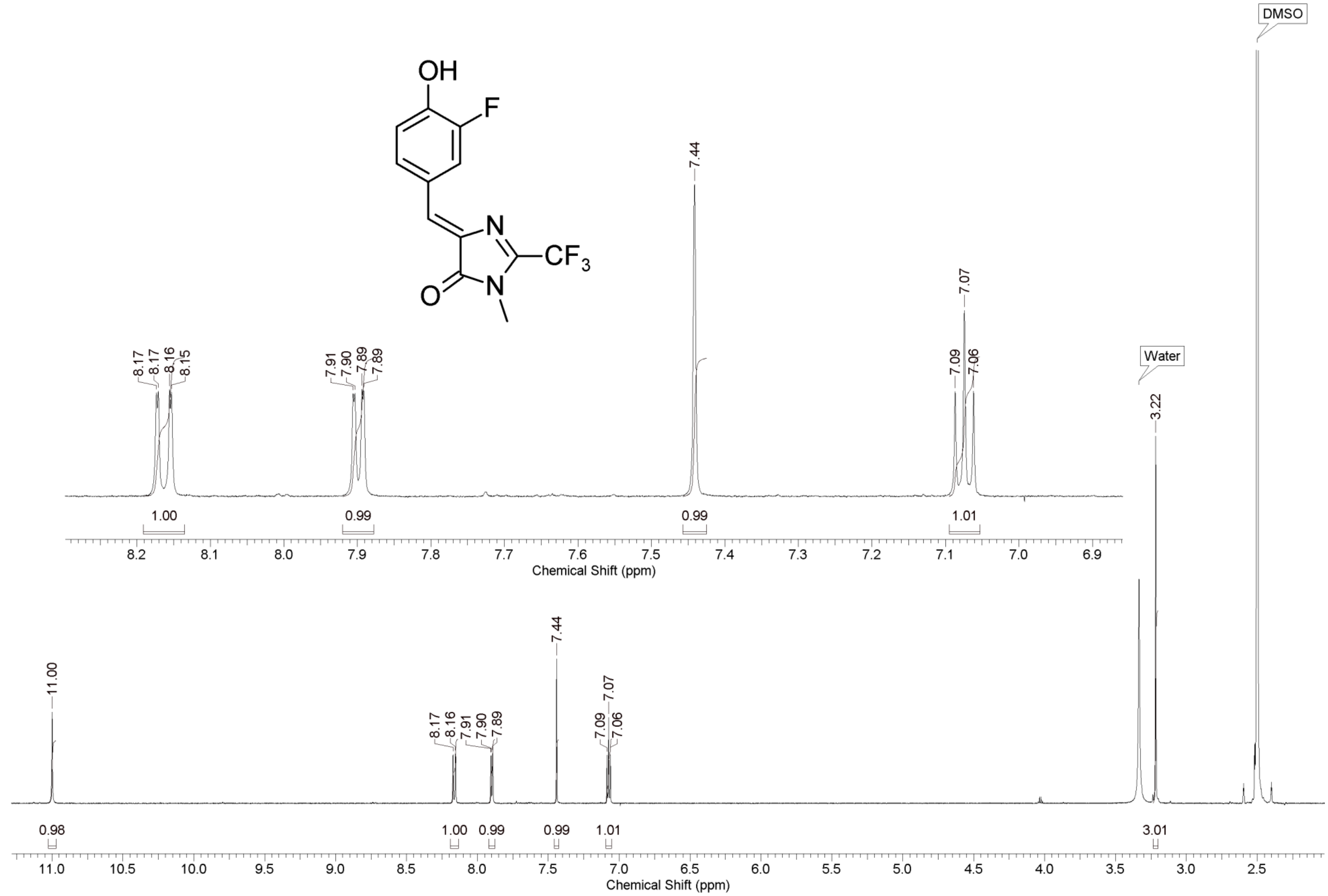

Appendix S31. ^{1}H NMR spectrum of (*Z*)-5-(3-fluoro-4-hydroxybenzylidene)-3-methyl-2-(trifluoromethyl)-3,5-dihydro-4*H*-imidazol-4-one **(2b)**

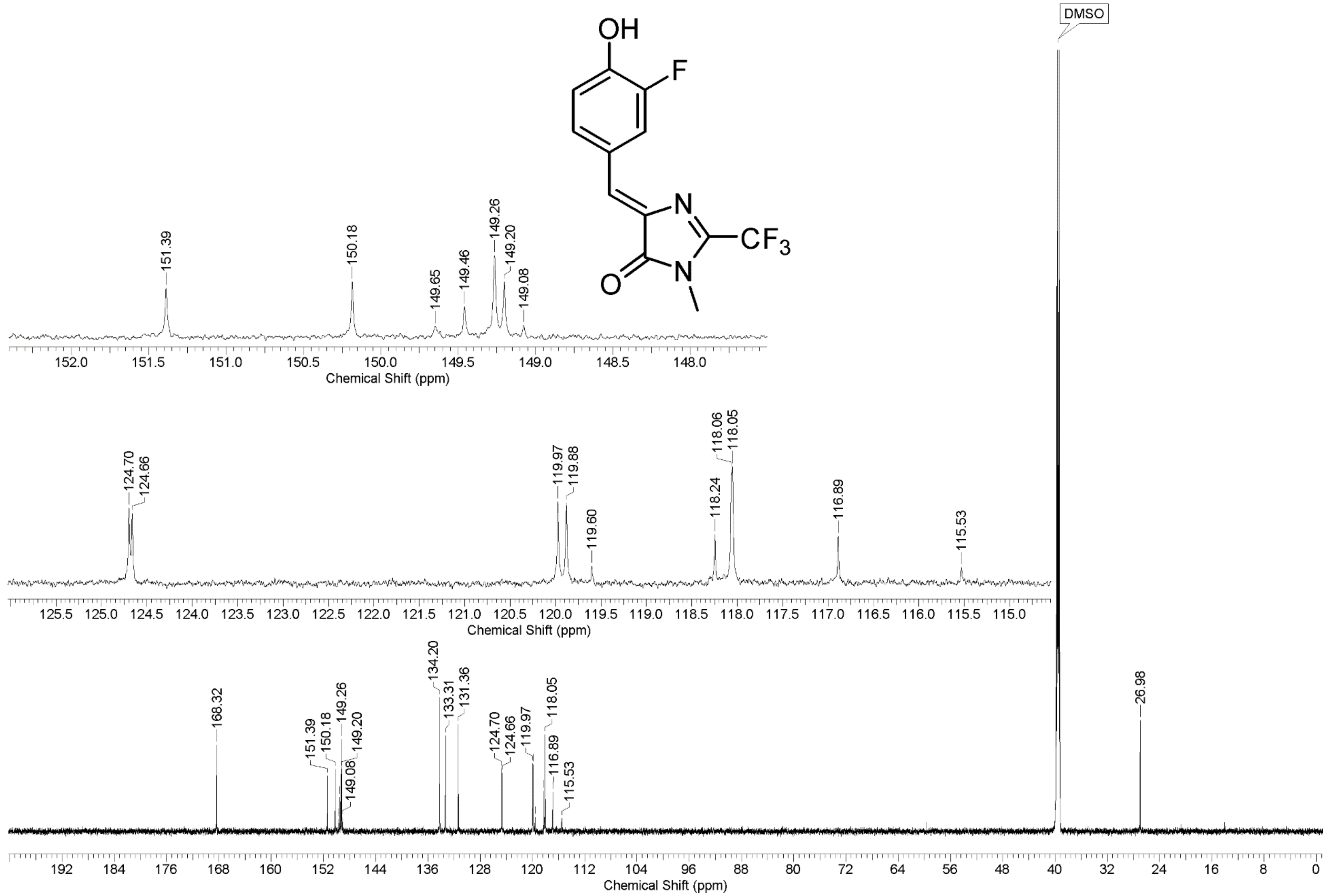

Appendix S32. ^{13}C NMR spectrum of (*Z*)-5-(3-fluoro-4-hydroxybenzylidene)-3-methyl-2-(trifluoromethyl)-3,5-dihydro-4*H*-imidazol-4-one **(2b)**

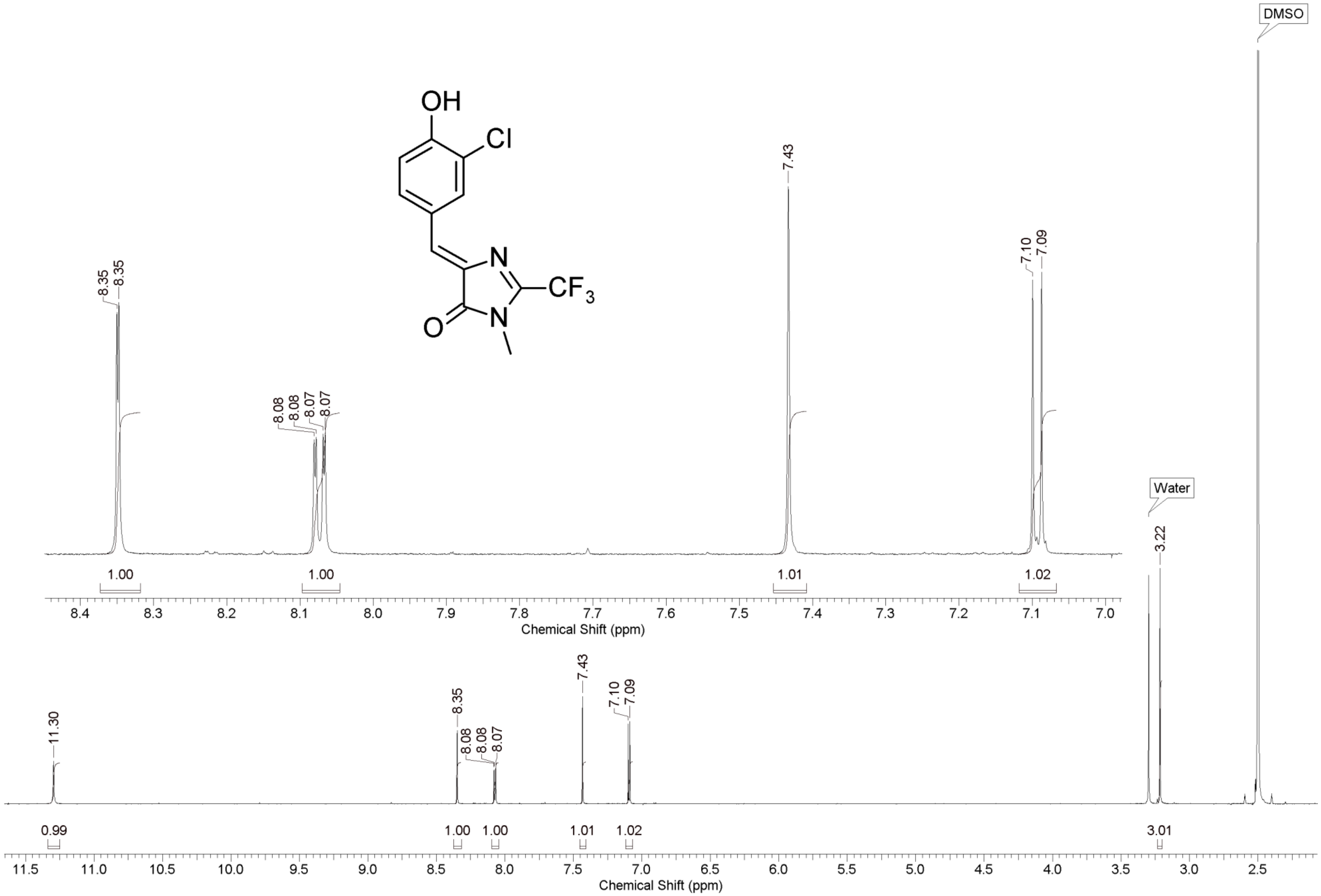

Appendix S33. ^{1}H NMR spectrum of (*Z*)-5-(3-chloro-4-hydroxybenzylidene)-3-methyl-2-(trifluoromethyl)-3,5-dihydro-4*H*-imidazol-4-one **(3b)**

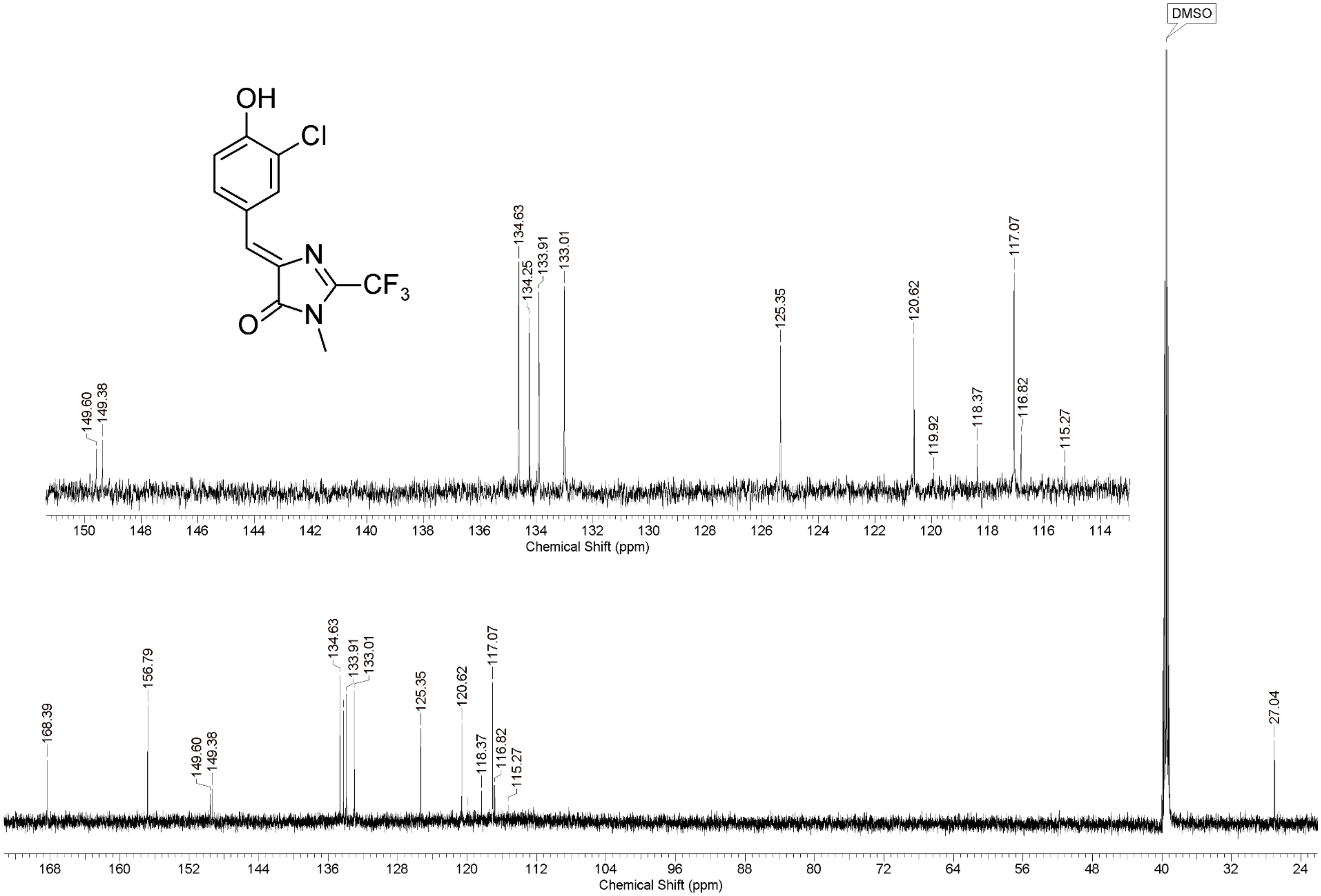

Appendix S34. ^{13}C NMR spectrum of (*Z*)-5-(3-chloro-4-hydroxybenzylidene)-3-methyl-2-(trifluoromethyl)-3,5-dihydro-4*H*-imidazol-4-one **(3b)**

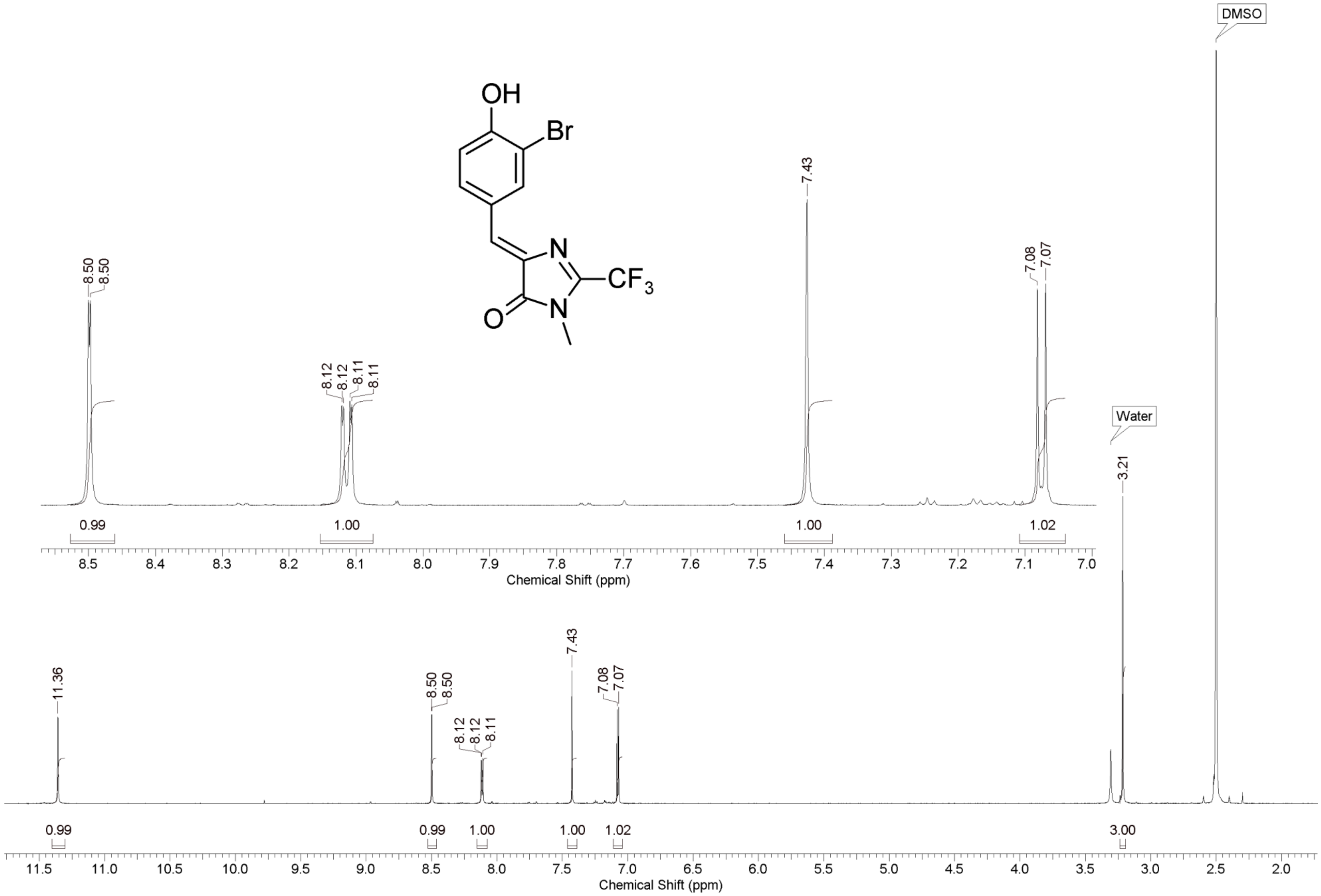

Appendix S35. ^{1}H NMR spectrum of (*Z*)-5-(3-bromo-4-hydroxybenzylidene)-3-methyl-2-(trifluoromethyl)-3,5-dihydro-4*H*-imidazol-4-one **(4b)**

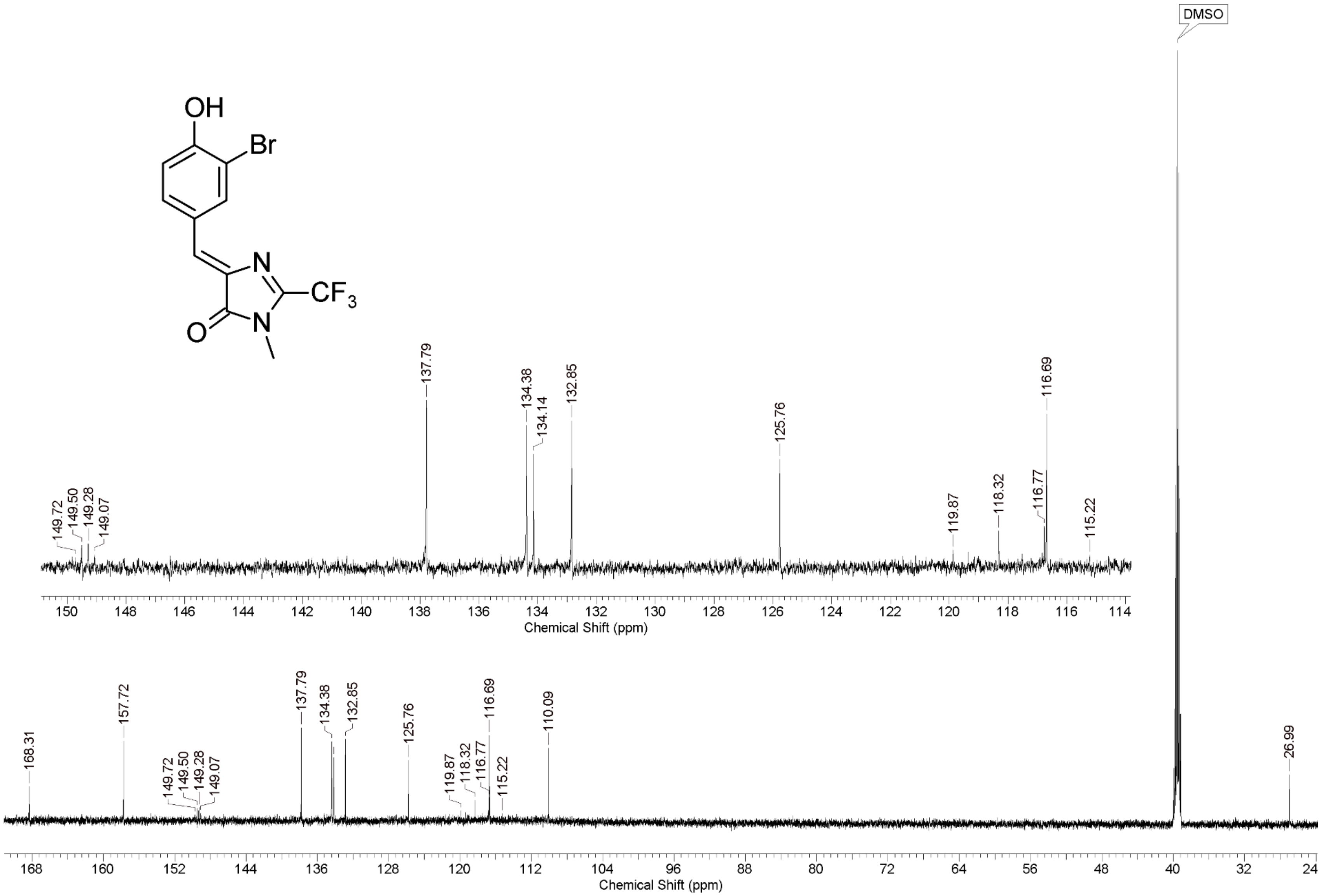

Appendix S36. ^{13}C NMR spectrum of (*Z*)-5-(3-bromo-4-hydroxybenzylidene)-3-methyl-2-(trifluoromethyl)-3,5-dihydro-4*H*-imidazol-4-one **(4b)**

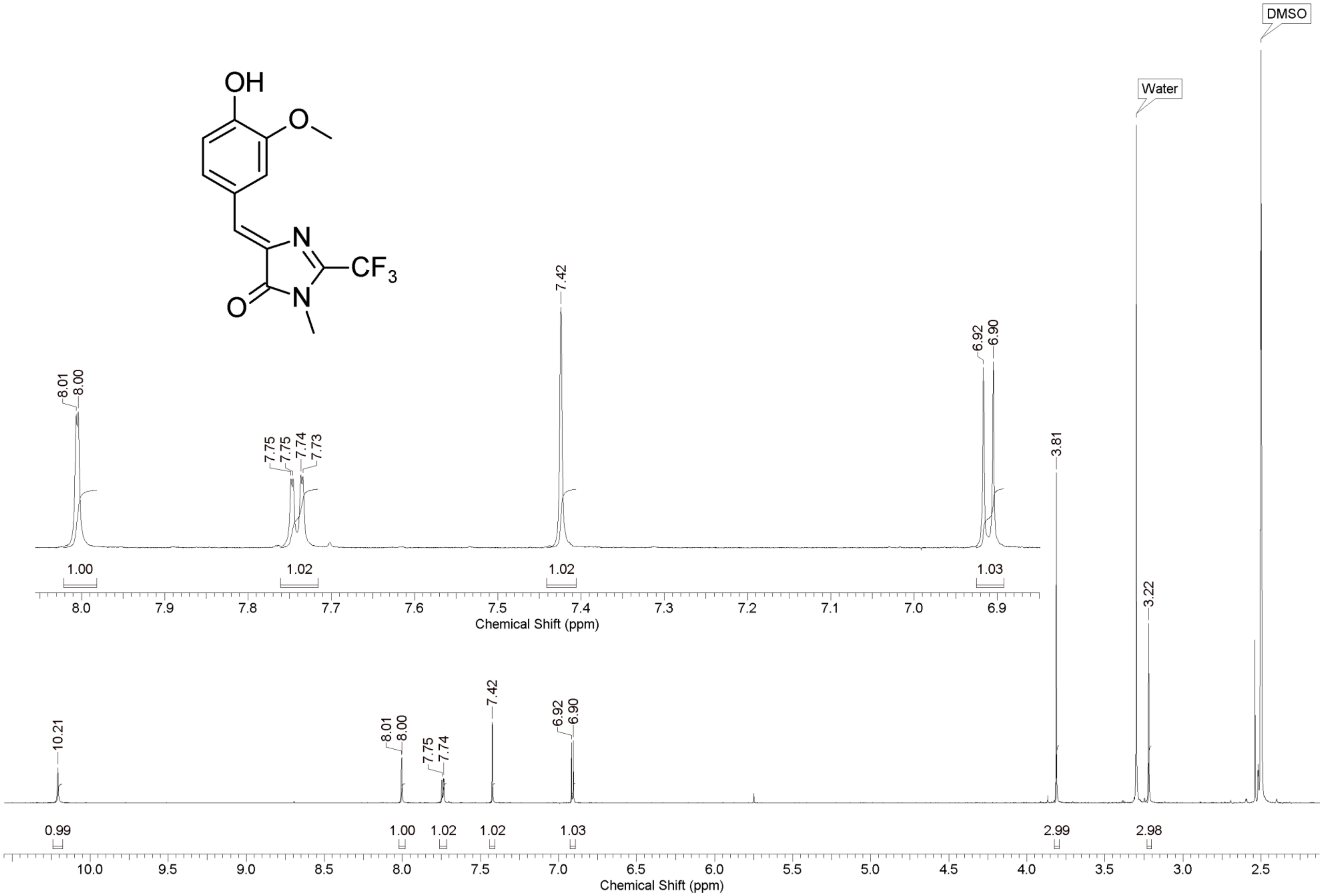

Appendix S37. ^{1}H NMR spectrum of (*Z*)-5-(4-hydroxy-3-methoxybenzylidene)-3-methyl-2-(trifluoromethyl)-3,5-dihydro-4*H*-imidazol-4-one **(5b)**

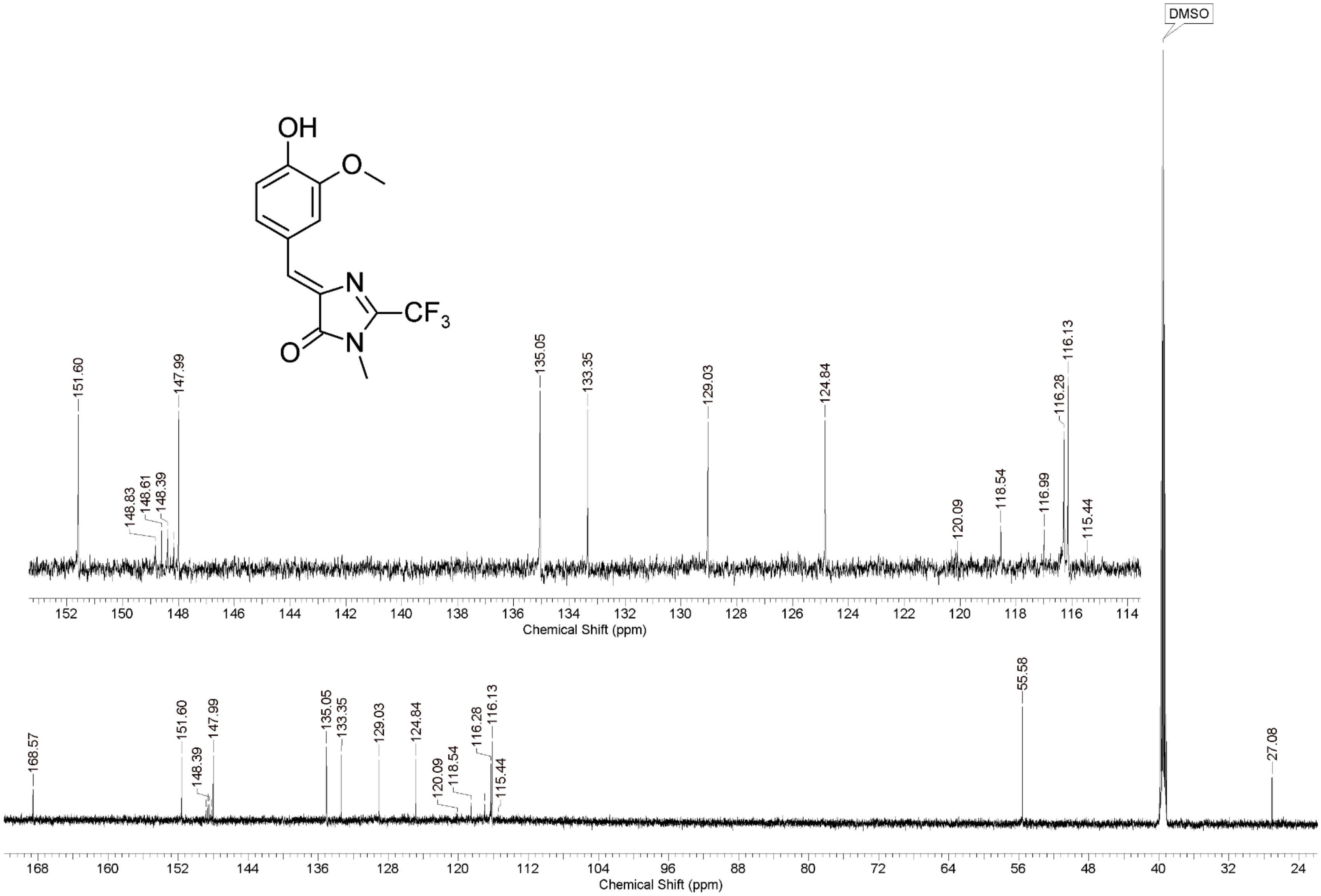

Appendix S38. ^{13}C NMR spectrum of (*Z*)-5-(4-hydroxy-3-methoxybenzylidene)-3-methyl-2-(trifluoromethyl)-3,5-dihydro-4*H*-imidazol-4-one **(5b)**

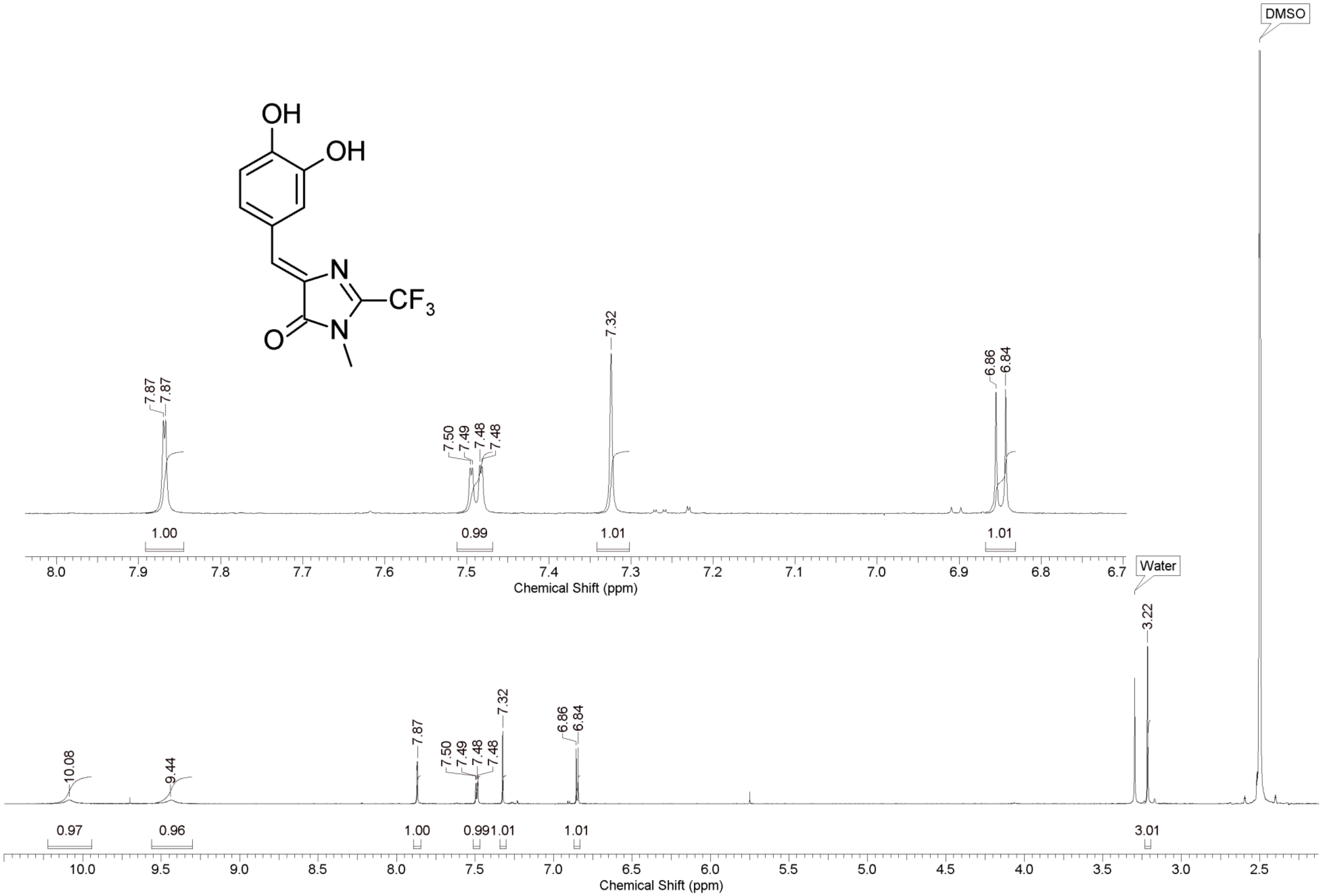

Appendix S39. ^{1}H NMR spectrum of (*Z*)-5-(3,4-dihydroxybenzylidene)-3-methyl-2-(trifluoromethyl)-3,5-dihydro-4*H*-imidazol-4-one **(6b)**

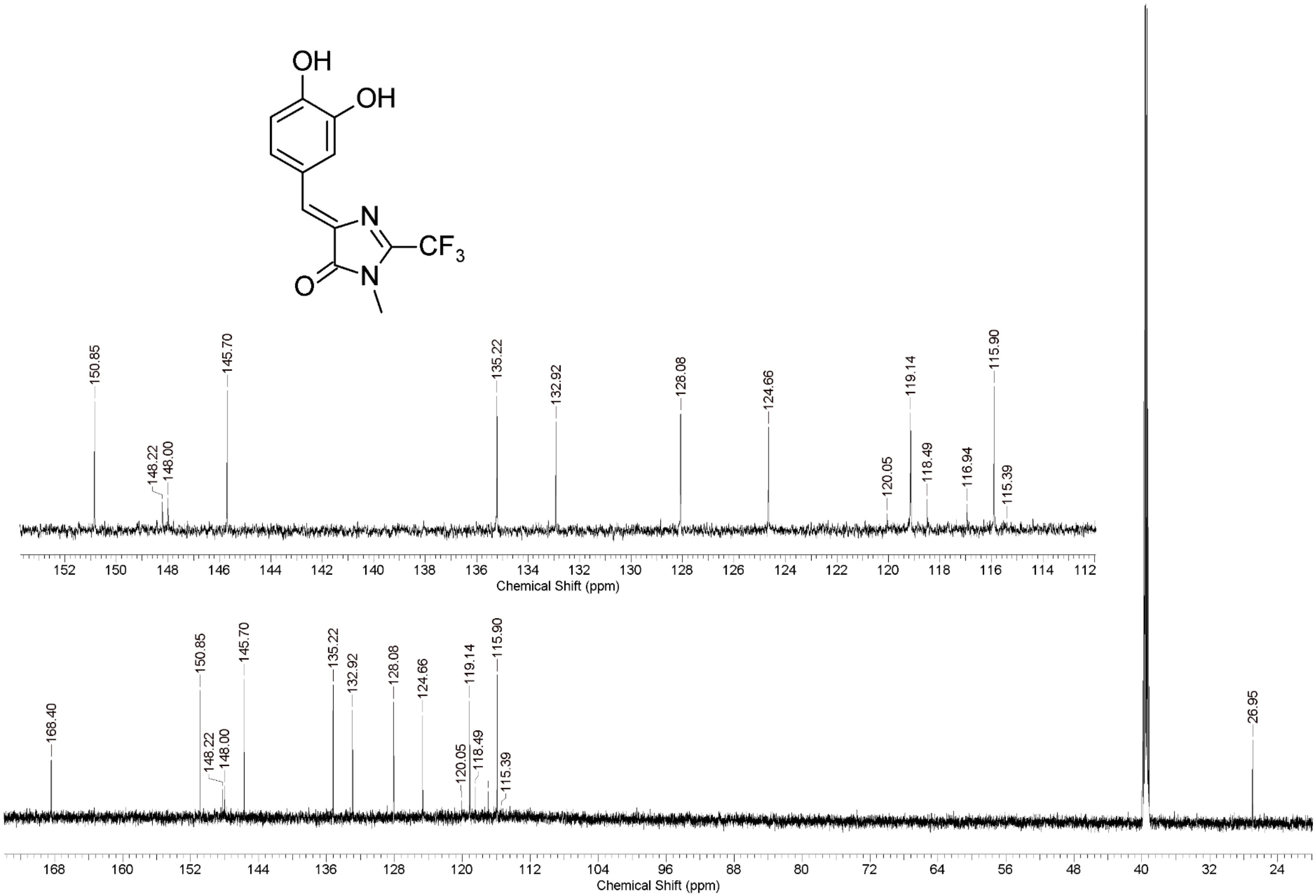

Appendix S40. ^{13}C NMR spectrum of (*Z*)-5-(3,4-dihydroxybenzylidene)-3-methyl-2-(trifluoromethyl)-3,5-dihydro-4*H*-imidazol-4-one **(6b)**

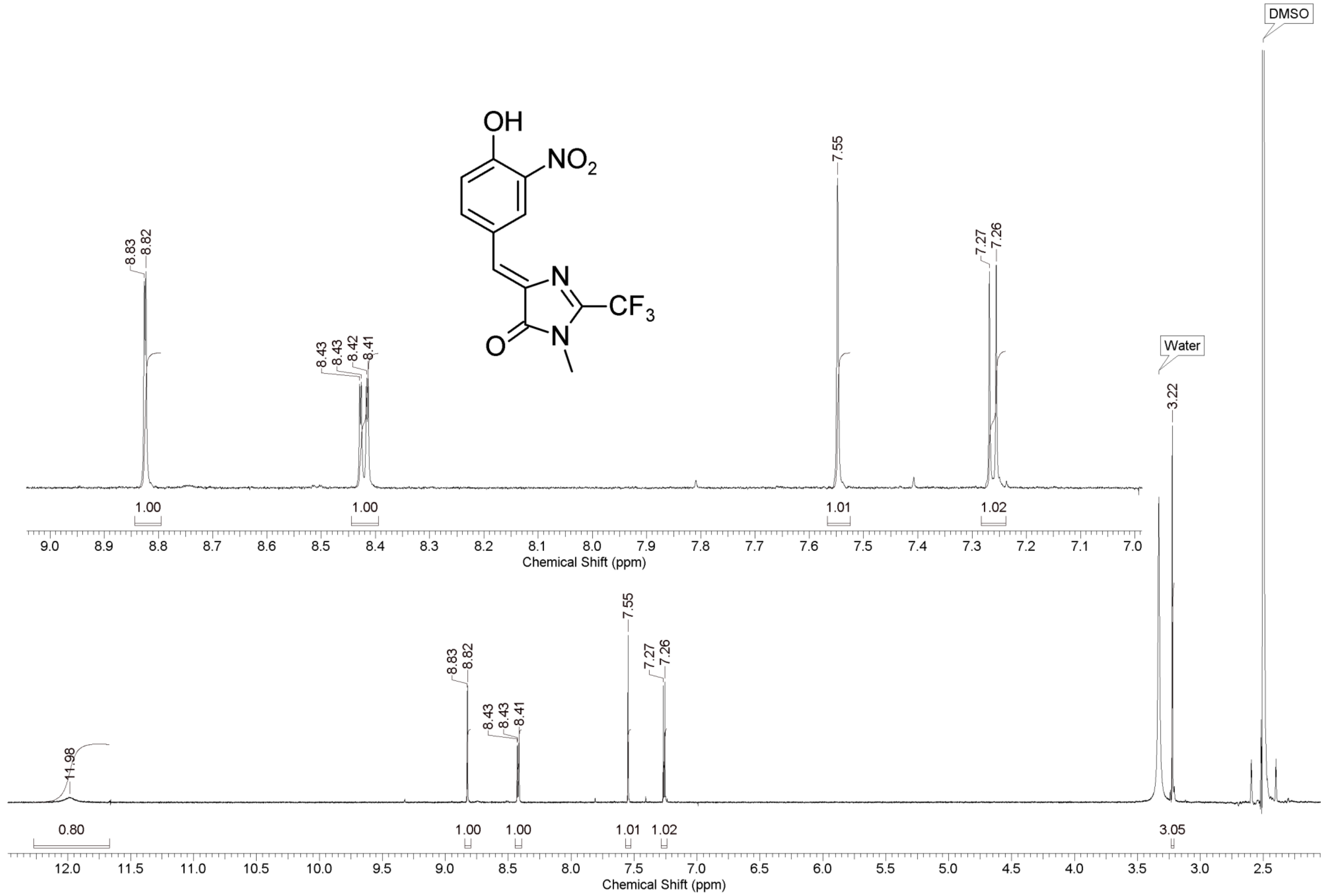

Appendix S41. ^{1}H NMR spectrum of (*Z*)-5-(4-hydroxy-3-nitrobenzylidene)-3-methyl-2-(trifluoromethyl)-3,5-dihydro-4*H*-imidazol-4-one

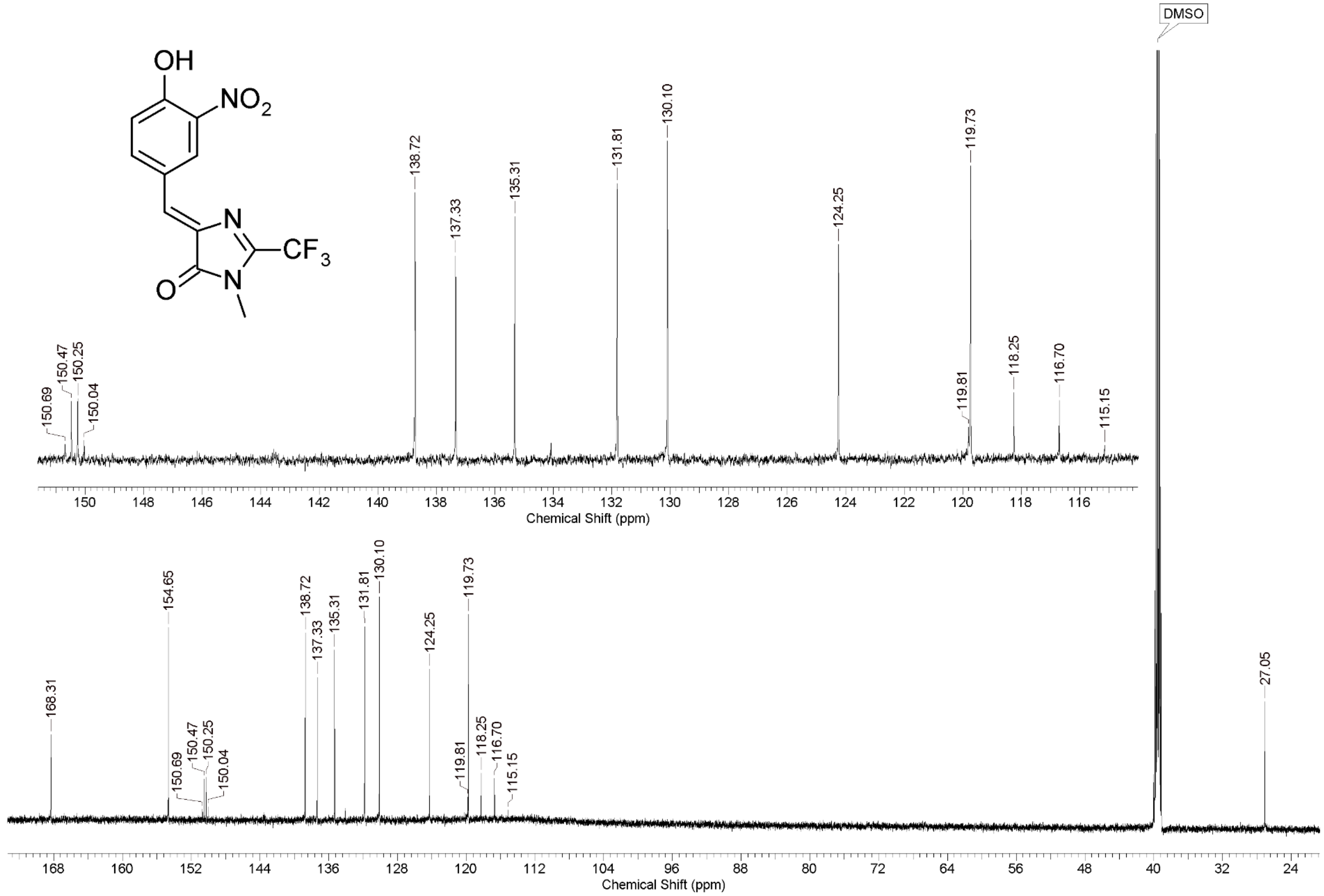

Appendix S42. ^{13}C NMR spectrum of (*Z*)-5-(4-hydroxy-3-nitrobenzylidene)-3-methyl-2-(trifluoromethyl)-3,5-dihydro-4*H*-imidazol-4-one

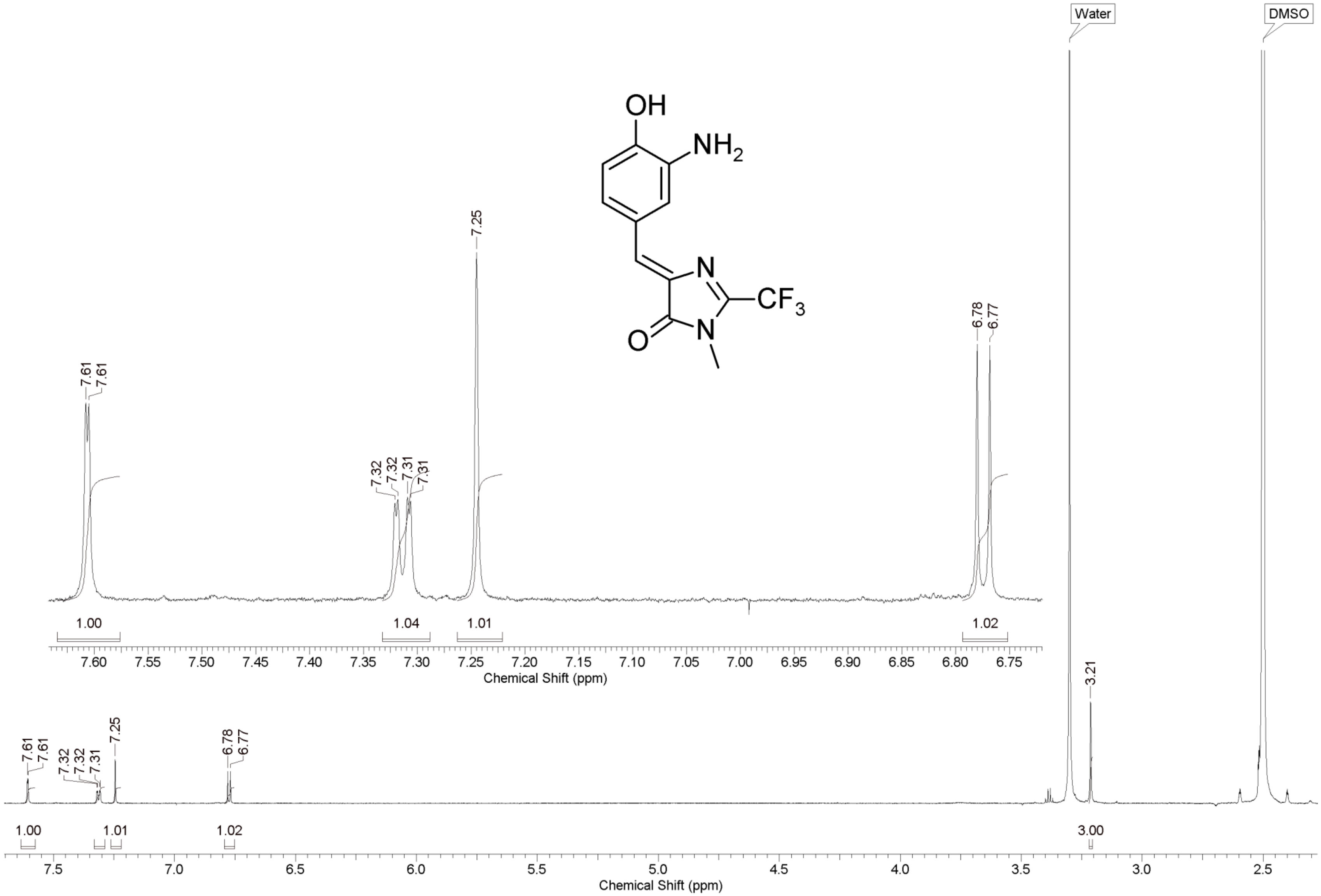

Appendix S43. ^{1}H NMR spectrum of (*Z*)-5-(4-hydroxy-3-aminobenzylidene)-3-methyl-2-(trifluoromethyl)-3,5-dihydro-4*H*-imidazol-4-one **(7b)**

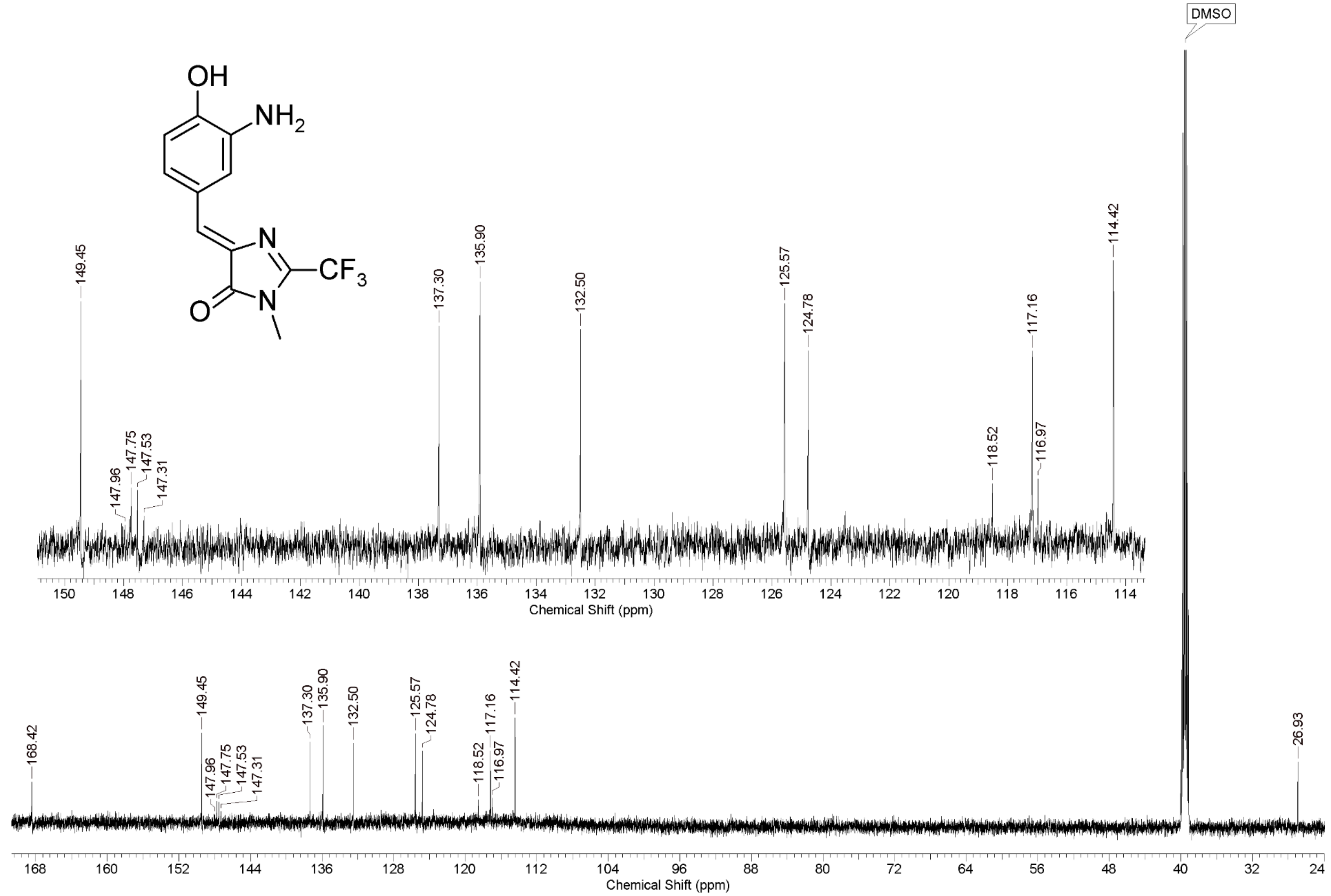

Appendix S44. ^{13}C NMR spectrum of (*Z*)-5-(4-hydroxy-3-aminobenzylidene)-3-methyl-2-(trifluoromethyl)-3,5-dihydro-4*H*-imidazol-4-one **(7b)**

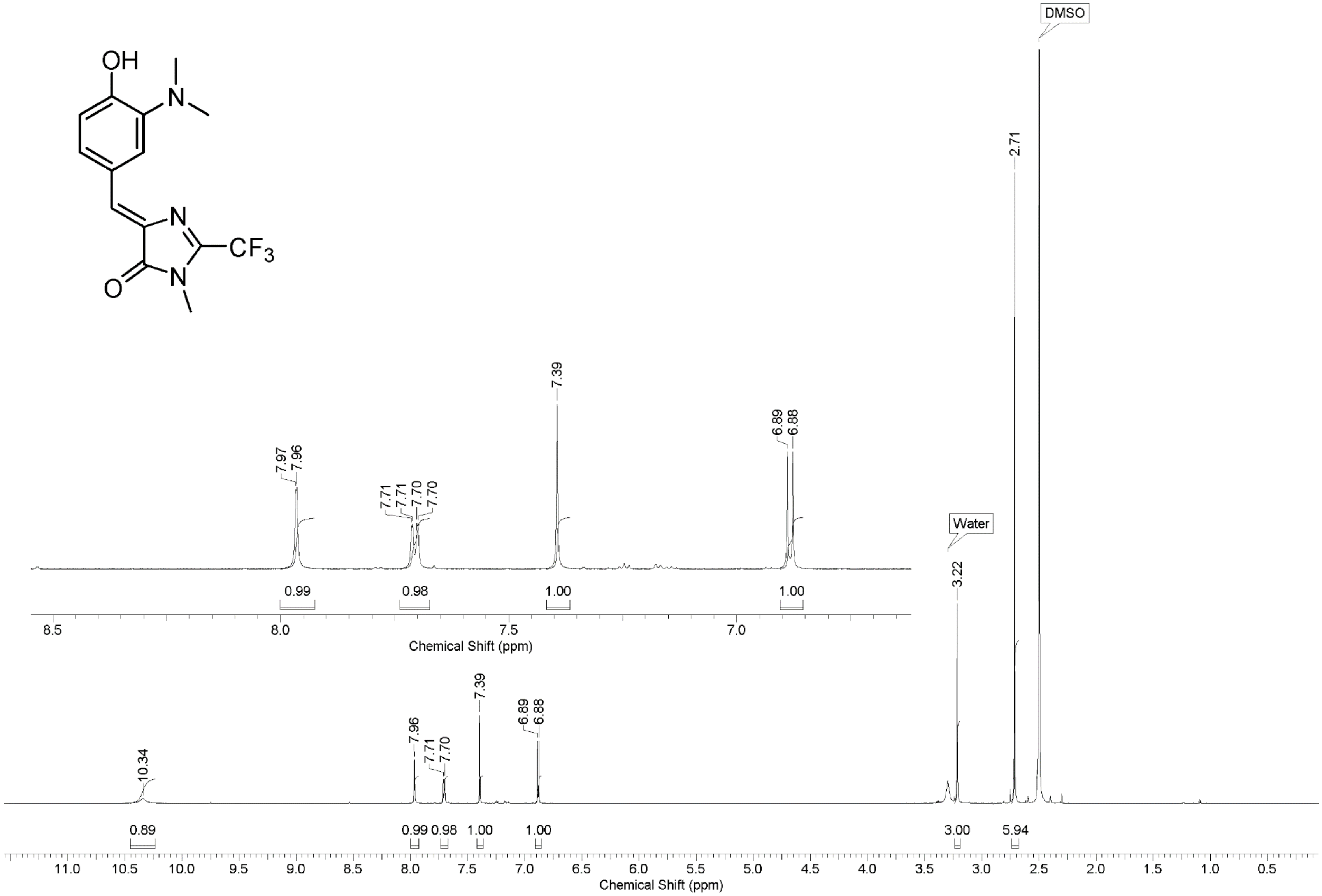

Appendix S45. ^{1}H NMR spectrum of (*Z*)-5-(3-(dimethylamino)-4-hydroxybenzylidene)-3-methyl-2-(trifluoromethyl)-3,5-dihydro-4*H*-imidazol-4-one **(8b)**

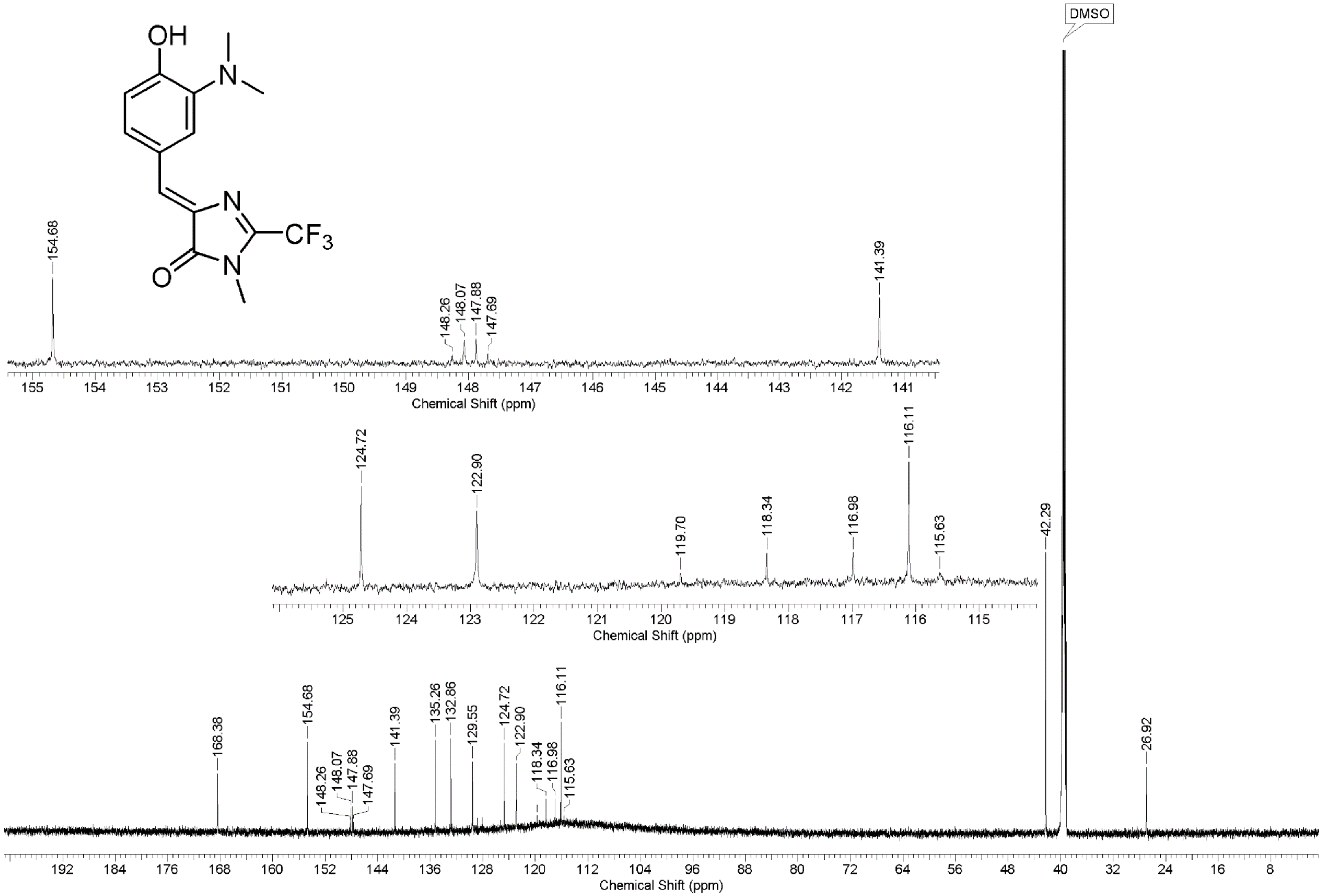

Appendix S46. ^{13}C NMR spectrum of (*Z*)-5-(3-(dimethylamino)-4-hydroxybenzylidene)-3-methyl-2-(trifluoromethyl)-3,5-dihydro-4*H*-imidazol-4-one **(8b)**

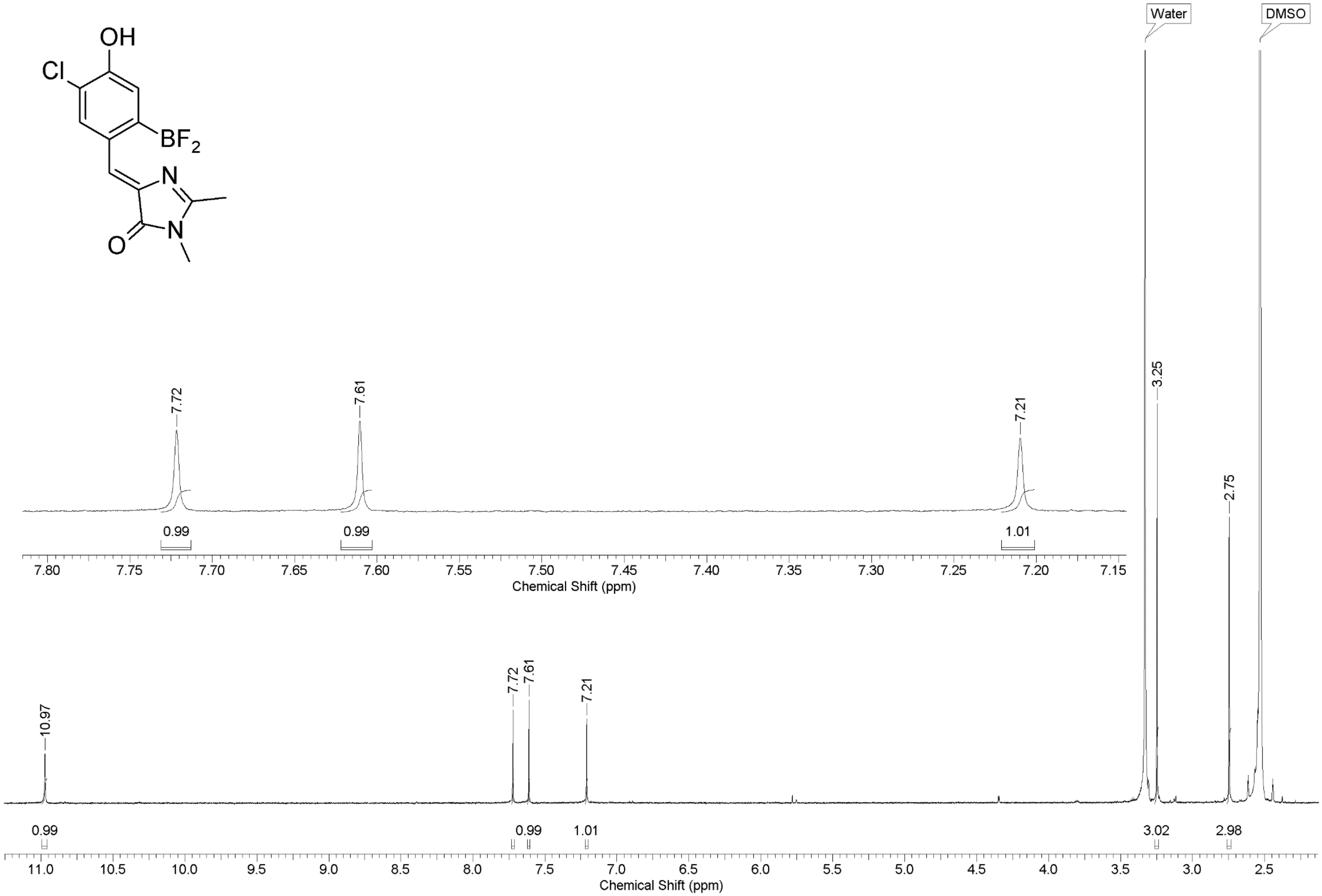

Appendix S47. ^{1}H NMR spectrum of (*Z*)-5-(5-chloro-2-(difluoroboranyl)-4-hydroxybenzylidene)-2,3-dimethyl-3,5-dihydro-4*H*-imidazol-4-one

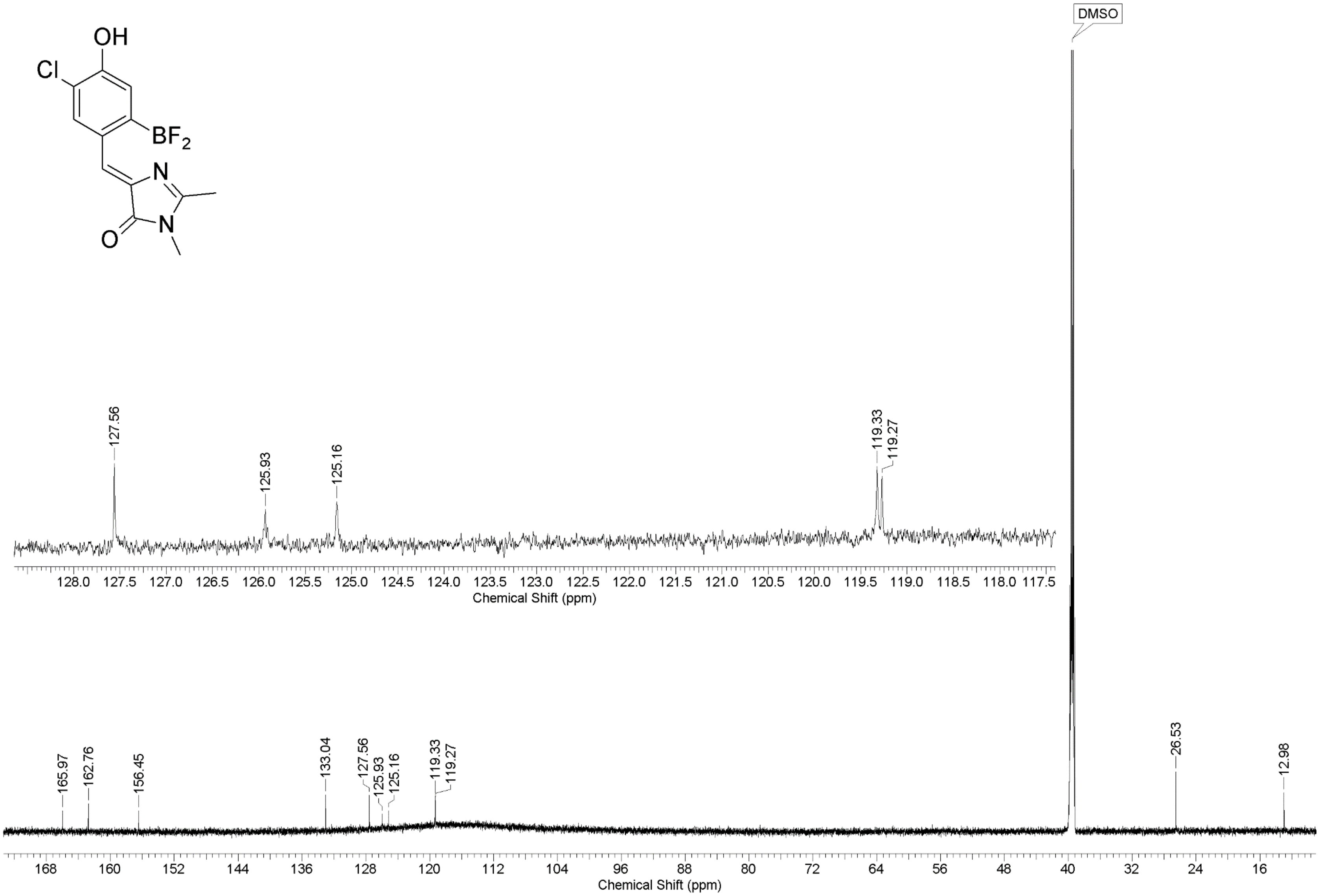

Appendix S48. ^{13}C NMR spectrum of (*Z*)-5-(5-chloro-2-(difluoroboranyl)-4-hydroxybenzylidene)-2,3-dimethyl-3,5-dihydro-4*H*-imidazol-4-one

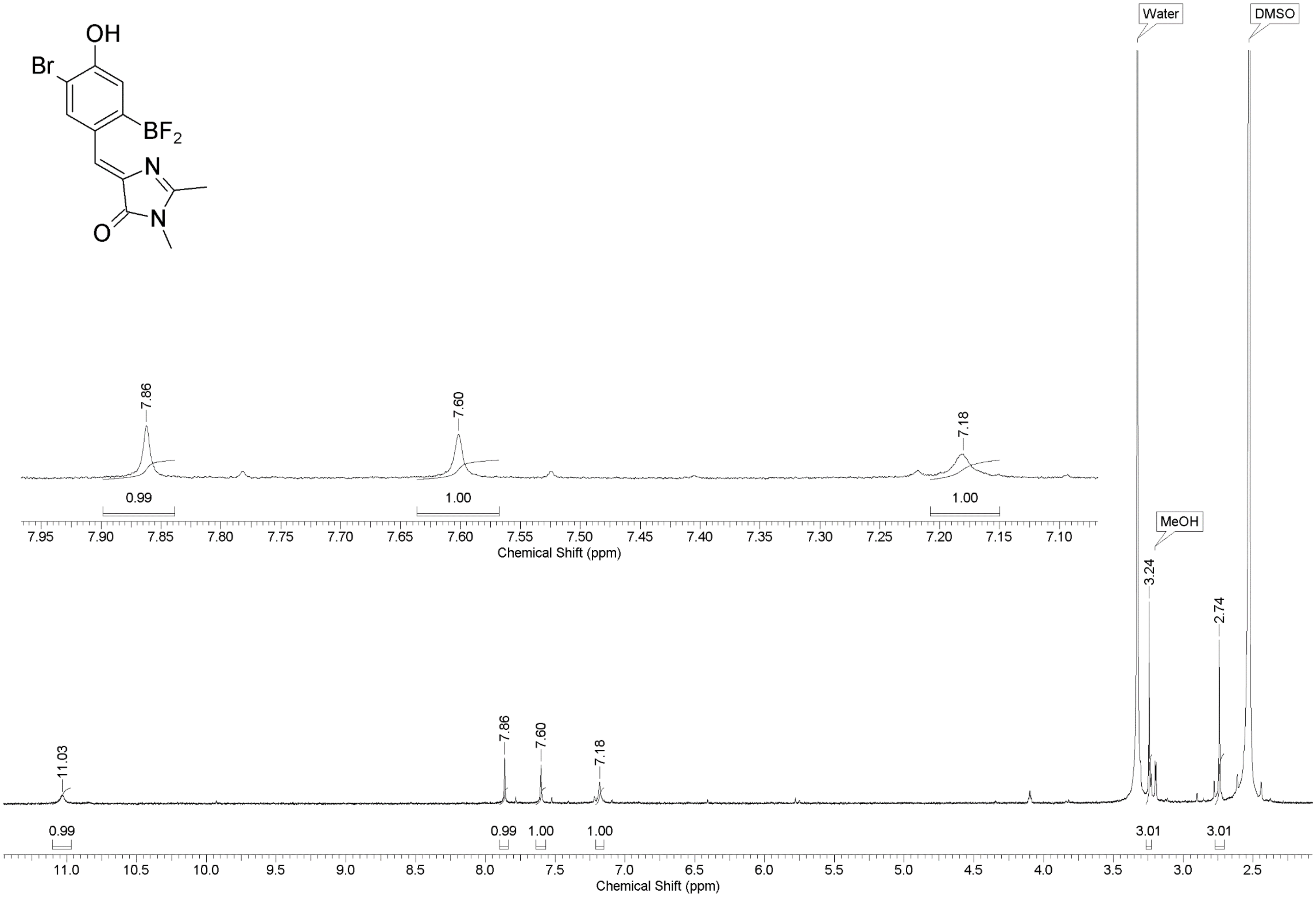

Appendix S49. ^{1}H NMR spectrum of (*Z*)-5-(5-bromo-2-(difluoroboranyl)-4-hydroxybenzylidene)-2,3-dimethyl-3,5-dihydro-4*H*-imidazol-4-one

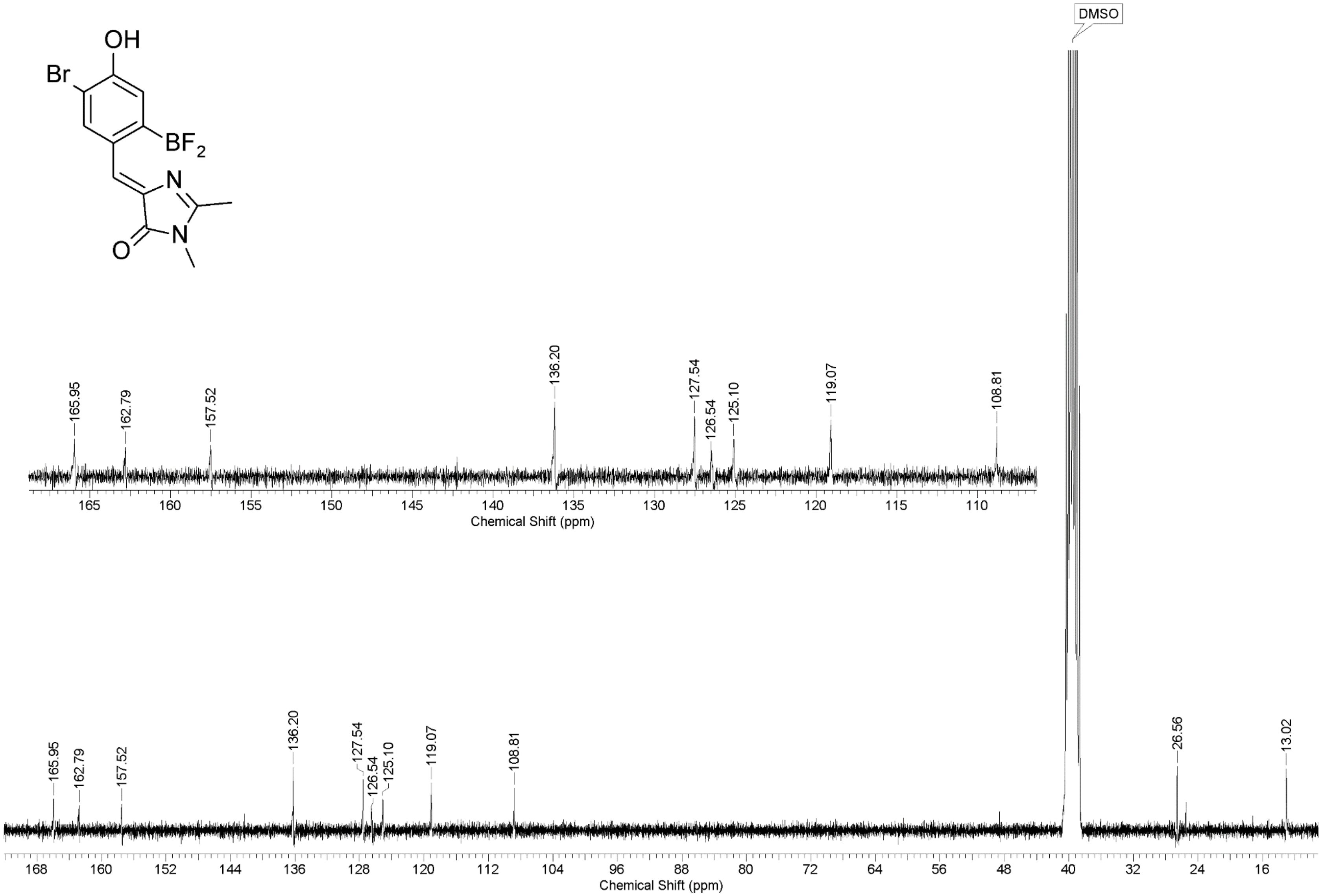

Appendix S50. ^{13}C NMR spectrum of (*Z*)-5-(5-bromo-2-(difluoroboranyl)-4-hydroxybenzylidene)-2,3-dimethyl-3,5-dihydro-4*H*-imidazol-4-one
